# A Mild One-Pot Reduction of Phosphine(V) Oxides Affording Phosphines(III) and Their Metal Catalysts

**DOI:** 10.1021/acs.organomet.0c00788

**Published:** 2021-03-05

**Authors:** Łukasz Kapuśniak, Philipp N. Plessow, Damian Trzybiński, Krzysztof Woźniak, Peter Hofmann, Phillip Iain Jolly

**Affiliations:** † Biological and Chemical Research Centre, Faculty of Chemistry, University of Warsaw, Żwirki i Wigury Street 101, 02-089 Warsaw, Poland; ‡ Institute of Catalysis Research and Technology, Karlsruhe Institute of Technology, Hermann-von-Helmholtz-Platz 1, D-76344 Eggenstein-Leopoldshafen, Germany; § Organisch-Chemisches Institut, Heidelberg University, Im Neuenheimer Feld 270, 69120 Heidelberg, Germany; ∥ Catalysis Research Laboratory (CaRLa), Im Neuenheimer Feld 584, 69120 Heidelberg, Germany

## Abstract

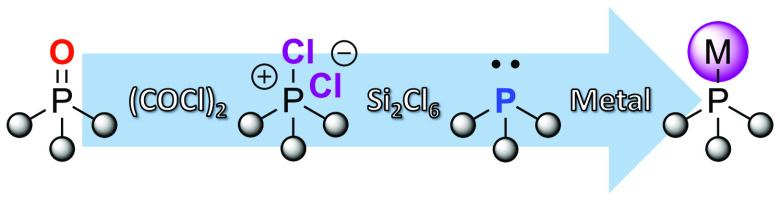

The metal-free reduction of a range of phosphine(V) oxides employing oxalyl chloride as an activating agent and hexachlorodisilane as reducing reagent has been achieved under mild reaction conditions. The method was successfully applied to the reduction of industrial waste byproduct triphenylphosphine(V) oxide, closing the phosphorus cycle to cleanly regenerate triphenylphosphine(III). Mechanistic studies and quantum chemical calculations support the attack of the dissociated chloride anion of intermediated phosphonium salt at the silicon of the disilane as the rate-limiting step for deprotection. The exquisite purity of the resultant phosphine(III) ligands after the simple removal of volatiles under reduced pressure circumvents laborious purification prior to metalation and has permitted the facile formation of important transition metal catalysts.

## Introduction

### Applications of Phosphine(III) Ligands and Synthesis

Phosphines and their derivatives are of significant importance to both academic and industrial chemistry. In particular, within organic chemistry phosphine(III) compounds have a distinguished history, mediating classical transformations such as the Appel,^1^ Mitsunobu,^2^ and Wittig^3,4^ reactions. Additionally, the ready modulation of electronic and steric properties of phosphine(III) has made them excellent ligands for the formation of well-defined transition metal complexes,^5^ although recalcitrant phosphine(V) oxides arise, when phosphine(III) compounds are employed as labile ligands^6^ or the metal complexes are simply decomposed, in the presence of a suitable oxidant.^7^ Arguably, the stoichiometric formation of phosphine(V) oxide waste from the above-named organic reactions presents an even greater issue, especially on the industrial scale,^3,4^ as the conversion of P^(V)^=O to the P^(III)^ oxidation state is nontrivial (*vide infra*).

### Direct Reduction of Phosphine(V) Oxide

**Scheme 1:**
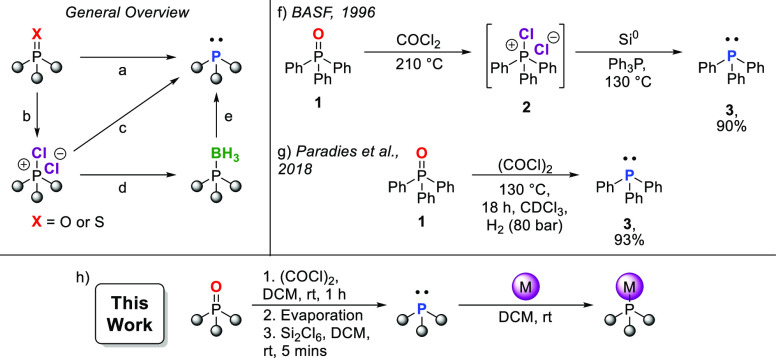
Phosphine Synthesis: Background and This Work Left: (a) direct reduction of P^(V)^=O or P^(V)^=S affording P^(III)^; (b) conversion of P^(V)^=O or P^(V)^=S to activated phosphonium salt; (c) reduction of activated phosphonium salt to P^(III)^; (d) conversion of activated phosphonium salt to phosphine–borane; (e) deprotection of phosphine–borane affording P^(III)^. Right-top: (f) BASFs conversion of Ph_3_PO to Ph_3_P using phosgene and silicon. Right-bottom: (g) Paradies et al. recent conversion of Ph_3_PO to Ph_3_P using oxalyl chloride and pressurized hydrogen. Center-bottom: (h) this work.

Given the significance of phosphine(III) compounds, a variety of anaerobic syntheses have been reported.^8,9^ However, the sensitivity of phosphine(III) to oxidation (requiring only minutes to hours) has led to the widespread use of “protected” phosphines,^10^ such as phosphine–borane adducts^11,12^ and phosphine(V) sulfides^13,14^ but predominantly phosphine(V) oxides.^15−17^ These precursors tolerate the reaction conditions necessary to construct more complex architectures^18^ although the protection must be removed in the penultimate^12,19^ or final^20,21^ step of the ligand synthesis. Thus, much attention has been focused on the conversion of P^(V)^=O to P^(III)^^15,16^ (Scheme 1a), including the use of silanes and siloxanes such as HSiCl_3_,^22−25^ HSiCl_3_/Ph_3_P,^26^ Si_2_Cl_6_,^24,27^ Si_2_Me_6_ with CsF/TBAF,^28^ HSi(OEt)_3_/Ti(O-*i*-Pr)_4_,^29^ PhSiH_3_,^30−32^ 1,1,3,3-tetramethyldisiloxane (TMDS) with CuX_2_,^33^ polymethylhydrosiloxane (PMHS),^34,35^ 1,3-diphenyldisiloxane (DPDS),^36^ and (EtO)_2_MeSiH/(RO)_2_P(O)OH;^37^ aluminum hydrides such as LiAlH_4_,^38,39^ LiAlH_4_/CeCl_3_,^40^ AlH_3_,^41^ and HAl(*i*-Bu)_2_;^42^ low-valent metals such as SmI_2_/HMPA (hexamethylphosphoramide)^43^ or Cp_2_TiCl_2_/Mg;^44^ hydrocarbon/activated carbon;^45^ and electrochemical reduction.^46−48^ A mild iodine-catalyzed reduction of phosphine(V) oxides employing a sacrificial electron-rich phosphine was developed by Laven and Kullberg,^49^ while Li et al.^50^ employed less expensive phosphite, although in both cases P^(V)^=O-containing contaminants must be removed from the final products. Thus, disadvantages of these procedures include harsh reaction conditions, toxic and/or highly reactive, potentially explosive reducing agents, narrow scope or undesirable side reactions, *e.g.*, C–P,^51,52^ C–O,^52^ or P–N^53−56^ bond cleavage, and laborious column chromatography to purify the desired phosphine(III).

### Reduction of Activated Chlorophosphonium Salts

The inherent stability of the P^(V)^=O has compelled others to explore sequential activation reduction methods, *i.e.*, the conversion of the phosphine(V) oxide to more reactive chlorophosphonium salts (CPS) and subsequent reduction (Scheme 1b,c). Horner, Hoffmann, and Beck first published the reduction of chlorotriphenylphosphonium chloride (Ph_3_PCl_2_) in 1958,^57^ with both LiAlH_4_ and sodium. The following year a sequential activation and deprotection was published, converting triphenylphosphine(V) oxide (Ph_3_PO) first to activated CPS, Ph_3_PCl_2_, before it was reduced to triphenylphosphine (Ph_3_P) with sodium metal.^58^ Being readily afforded *via* inexpensive chlorinating reagents,^59^ CPSs have also been reduced with aluminum/metal salts,^60^ alkali metals,^57,58^ LiAlH_4_,^57,61,62^ thiols/Et_3_N,^63^ activated carbon,^45^ Hantzsch ester/Et_3_N,^64^ electrochemically,^46−48,65,66^ elemental aluminum^67,68^ or silicon,^69^ and hydrogenolysis,^70^ which may be catalyzed by frustrated Lewis pairs (FLPs).^71,72^ Harsh metal bases and Grignard reagents have even been used to deprotect certain CPSs.^73^ Alternatively, CPS can be converted to phosphine–boranes by either NaBH_4_^74,75^ or LiBH_4_,^76−79^ although ultimately the borane “protecting group” itself requires removal (Scheme 1b,d,e).

### Motivation to Develop a New Facile Reduction of Phosphine(V) Oxides

**Scheme 2:**
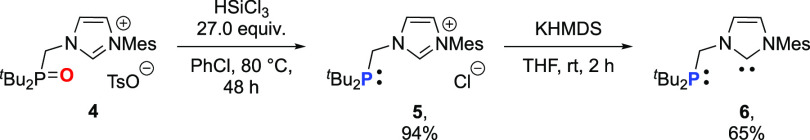
Problematic Reduction of NHCP Precursor Synthesis of NHCP **6***via* the challenging reduction of phosphine(V) oxide in azolium salt **4** to phosphine(III) **5**.

Our interest in phosphine(V) oxides reduction originates from our desire to explore bulky *N*-phosphinomethyl-functionalized *N*-heterocyclic carbene ligands (NHCPs)^80,81^ as potential ligands for new olefin metathesis catalyst (Scheme 2).^19^ Progress has been severely hampered due to difficulties accessing azolium salt **5**, with the problematic reduction of **4** being achievable only with a large excess of trichlorosilane (27.0 equiv) in anhydrous degassed chlorobenzene at elevated temperature over 2 days.^19^ As well as the lengthy reaction time, we experienced some reproducibility issues, with the unsuccessful reduction being accompanied by the decomposition of the precious azolinium **4**, previously obtained *via* a multistep synthesis.^19^ In light of this, a simple procedure for the conversion of **4** to **5** would be a great advantage. Such a process might also permit access to other challenging phosphine(III) and metal catalysts as well as permitting the recovery of the valuable phosphine(III) ligands: “closing the phosphorus cycle” is of increasing importance due to environmental and availability concerns.^82−84^ Herein, we report a new activation/deprotection of phosphine(V) oxides without the use of harsh reaction conditions, metals, or sacrificial phosphanes. Intermediate CPSs are directly converted to desired phosphines by reaction with hexachlorodisilane. Mechanistic details have been elucidated by experimentation and supported by computation. The “one-pot” procedure affords excellent yields of pure phosphine(III) ligands that can be telescoped into formation of transition metal catalysts without the prior need for silica gel chromatography.

## Results and Discussion

### Reduction of Activated CPSs with Disilane

In 1996, BASF reported the generation of tetrachlorosilane (SiCl_4_) when the CPS, Ph_3_PCl_2_ (**2**), was heated with elemental silicon at 185 °C.^69^ Not wanting to expose our ligand precursor to such harsh reaction conditions, we hypothesized that hexachlorodisilane might serve as a suitable surrogate for elemental silicon and similarly generate 2 equiv of SiCl_4_ on reactions with a CPS. The abundant industrial byproduct Ph_3_PO (**1**) appeared to be the ideal test substrate,^3,4^ and was easily converted to activated Ph_3_PCl_2_ (**2**) with inexpensive oxalyl chloride.^59^ Gratifyingly on reaction with 1.1 equiv of hexachlorodisilane (Si_2_Cl_6_) at room temperature, both ^1^H NMR and ^31^P NMR indicated the immediate, clean, and complete formation of Ph_3_P (**3**), with ^29^Si NMR showing only the formation of tetrachlorosilane, SiCl_4_ (δ = −18.8 ppm). Motivated by the ability of Si_2_Cl_6_ to reduce **2**, we chose to explore other disilanes (Table 1, entries 2–10): 1,1,2,2-tetrachloro-1,2-dimethyldisilane (Si_2_Me_2_Cl_4_), hexamethyldisilane (Si_2_Me_6_), and hexaphenyldisilane (Si_2_Ph_6_), which might generate the corresponding attractive byproducts trichloromethylsilane (MeSiCl_3_), trimethylsilyl chloride (Me_3_SiCl), or triphenylsilyl chloride (Ph_3_SiCl). However, the more electron-rich and sterically hindered disilanes generated the desired phosphines in either lower yield, over extended reaction times or not at all. For instance, the addition of a single electron-donating methyl group to each of the silicon atoms in Si_2_Me_2_Cl_4_ drastically decreased the rate of reaction, with only a 28% conversion to **3** after 24 h, eventually reaching completion after 144 h. In contrast, the reaction with Si_2_Cl_6_ was complete in under 5 min.^a^ No reaction was observed for even more electron-rich and sterically shielded Si_2_Ph_6_ or Si_2_Me_6_.

**Table 1:**
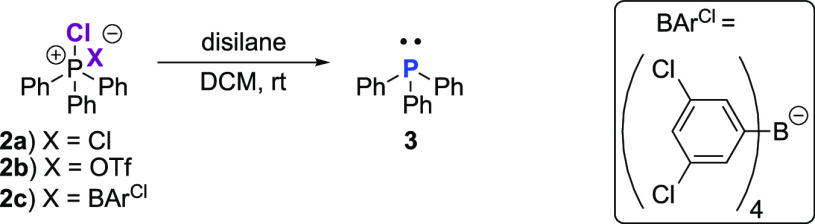
Reaction of Phosphonium Salts with Disilanes

entry	CPS **2a**–**c**, X =	disilane	equiv	time	conv to **3** [%]^a^
1	Cl	Si_2_Cl_6_	1.1	5 min	100
2	Cl	Si_2_Me_2_Cl_4_	1.1	5 min	0
3	Cl	Si_2_Me_2_Cl_4_	1.1	1 day	28
4	Cl	Si_2_Me_2_Cl_4_	1.1	2 days	55
5	Cl	Si_2_Me_2_Cl_4_	1.1	3 days	72
6	Cl	Si_2_Me_2_Cl_4_	1.1	4 days	78
7	Cl	Si_2_Me_2_Cl_4_	1.1	5 days	83
8	Cl	Si_2_Me_2_Cl_4_	1.1	6 days	100
9	Cl	Si_2_Me_6_	1.0	1 day	0
10	Cl	Si_2_Ph_6_	1.0	1 day	0
11	OTf	Si_2_Cl_6_	1.1	10 min	7
12	OTf	Si_2_Cl_6_	1.1	1 day	80
13	OTf	Si_2_Cl_6_	1.1	2 days	100
14	BAr^Cl^	Si_2_Cl_6_	4	2 days	0

a Conversion judged by ^31^P NMR of **2a**–**c** relative to **3**.

### Scope of the New Procedure

With Si_2_Cl_6_ proving to be the reductant of choice, we expanded the application of the procedure to other phosphine(III) compounds.^b^ Aliphatic tricyclohexylphosphine (**7**) was afforded in 97% yield, in contrast to the recently reported hydrogenation at 130 °C, which notably afforded none of the desired phosphine(III) complexes.^71^ Cyclic alkene 2-phospholene oxide was also converted to P^(III)^ 2-phospholene (**8**)^77^ (98%) without the reduction or isomerization of the C=C bond. Reduction of phosphinamides without the P–N bond scission is particularly challenging;^53−56^ while Gilheany et al. synthesized “protected” aminophosphine–borane adducts from CPSs in excellent yields,^75^ we were able to furnish the free aminophosphine **9** directly (89%). The dimethylamino group in DavePhos **11** (93%) was also tolerated well, with fellow Buchwald ligand CyJohnPhos **10** being cleanly afforded in 95% yield. Chiral phosphines^8^ are still of great significance, and we chose to explore binaphthyl systems as the CPSs of P-chirogenic phosphines are known to racemize.^85^ The oxides of chiral phosphepines permit structure elaboration,^86^ and our new method rapidly afforded (*S*)-Ph-BINEPINE (**12**)^87^ (96% yield). (*R*)-MeO-MOP (**13**)^88^ was also readily synthesized (99%). It is of note that the direct reaction of MeO–MOP oxide with Si_2_Cl_6_ in acetonitrile led exclusively to scission of the C–O bond without reduction of P^(V)^=O,^52^ highlighting the divergence in the reactivity of the activated P^(V)^Cl_2_ compared to recalcitrant P^(V)^=O. Moreover, we observed no racemization in the case of either **12** or **13**.

**Figure 1:**
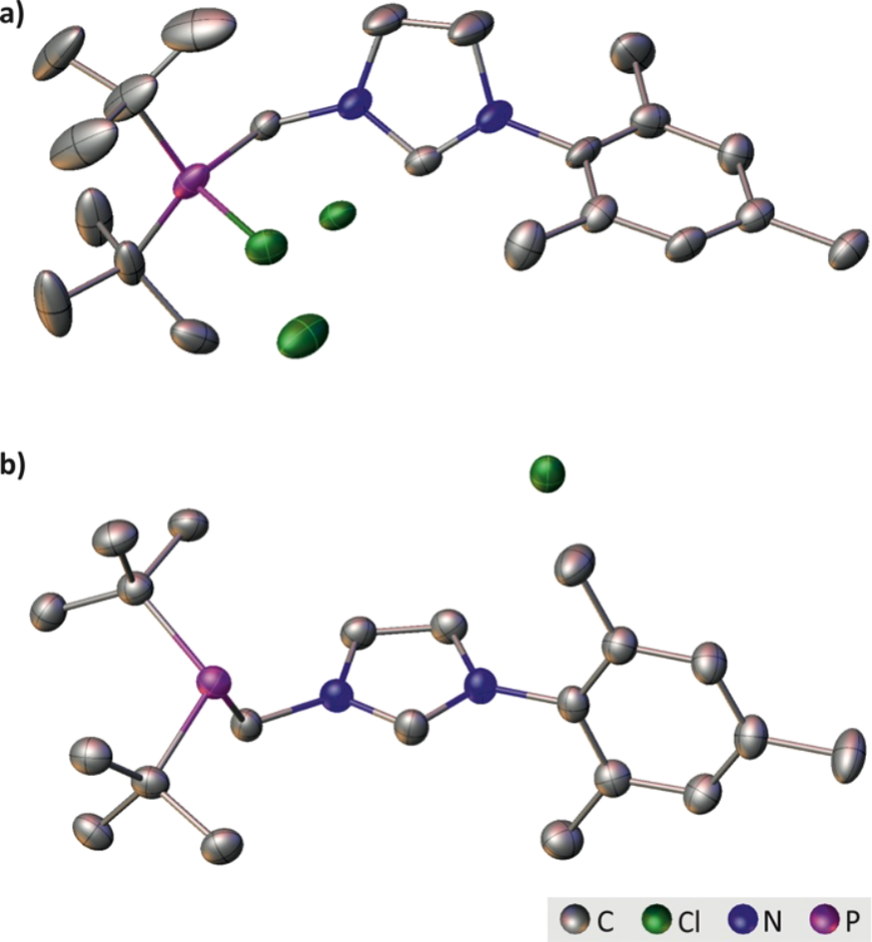
Graphical representation of molecular structure, where (a) CPS **15** and (B) azolium **5**. Displacement ellipsoids are drawn at the 50% probability level. The H atoms, the HCl molecule (CPS **15**), and the ionic pair “B” (azolium **5**) were omitted for clarity.

Having established the optimal conditions for the generation of a range of phosphine(III) compounds, we turned our attention back to azolinium **5**. The reaction of **4** with excess oxalyl chloride yielded a new chlorophosphonium bearing azolinium salt **15** (after removal of 4-toluenesulfonyl chloride produced by chlorination of the 4-toluenesulfonate; see the Supporting Information) which was readily transformed to the desired azolium **5** with hexachlorodisilane (1.5 equiv). The identity of both salts **5** and **15** was established by single-crystal X-ray diffraction analysis. Crystals suitable for this purpose were obtained by layering methylene chloride with hexane and storing at −30 °C. The salts crystallize in the monoclinic *P*2_1_/*c* (CPS **15**) and *P*2_1_/*n* (azolium **5**) space group, respectively. Graphical representation of molecular structure of both compounds is shown in Figure 1. The tetravalent phosphorus atom effectively means each molecule of CPS **15** has two dissociated chloride counteranions: one for each of the cationic phosphonium and the azolinium constituent parts. Interestingly, the asymmetric unit of the crystal lattice of **15** also contained a molecule of hydrochloride (Figure S2).^c^ The additional chloride counterion has important implications for the deprotection of **15**, which thus requires 1.5 equiv of hexachlorodisilane to fully convert the CPS to P^(III)^**5**: presumably, the extra Cl^–^ counterion of the imidazolium moiety also reacts with Si_2_Cl_6_ (*vide infra*). Finally, mesityl-substituted **5** could be facilely synthesized in an excellent 94% yield, without implementing harsh reaction conditions. In addition, we further demonstrated the usefulness of the new procedure at generating phosphine-bearing azolium salts with the synthesis of the 2,6-diisopropylphenyl analogue **14**, in a comparable 92% yield. More details concerning the crystal structure of CPS **15** and azolium **5** can be found in the Supporting Information (Figures S58–S66).

**Table 2:**
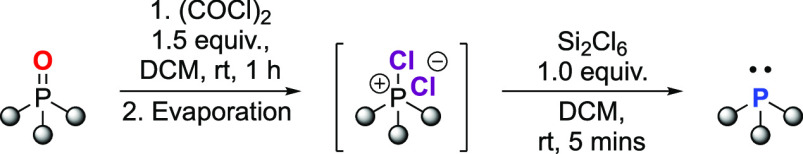

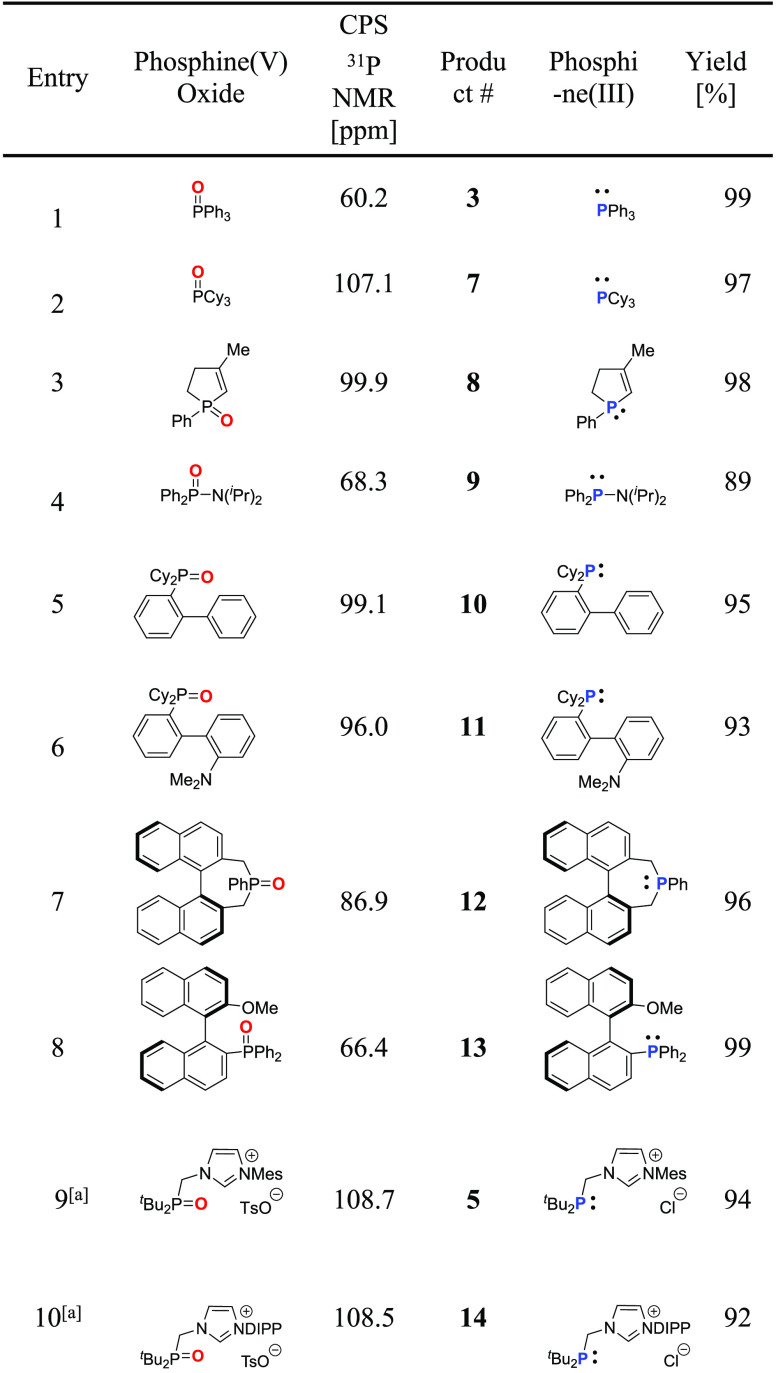
Conversion of Phosphine(V) Oxides to Phosphine(III) Ligands *via* CPS Intermediates

a The azolium salts were reacted with 5.0 equiv (COCl)_2_. The resultant CPS was separated from TsCl and then reacted with 1.5–1.6 equiv of Si_2_Cl_6_.

### Experimental and Computational Mechanism Studies

**Scheme 3:**
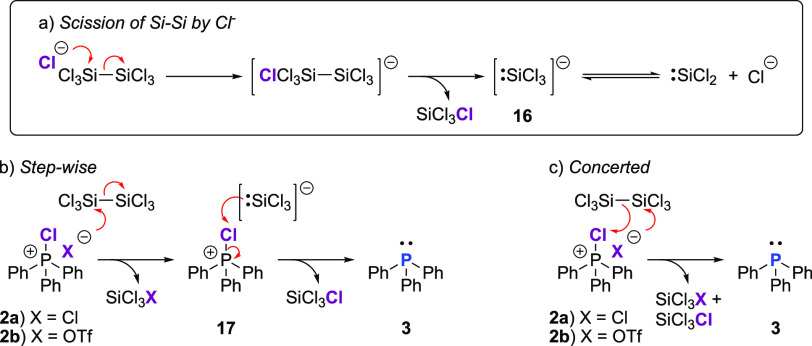
Reaction Mechanism of Si_2_Cl_6_ with Dissociated Chloride Anions Known formation of anion [:SiCl_3_]– from Si_2_Cl_6_. Stepwise reaction mechanism (bottom left). Concerted mechanism (bottom right).

CPSs in methylene chloride form a cationic phosphonium with a noncoordinated anionic chloride counteranion,^89−93^ while it has been demonstrated that Cl^–^ (e.g., from ammonium salts) leads to scission of the Si–Si bond in Si_2_Cl_6_ (Scheme 3).^94−99^ This lead us to surmise that the reaction is initiated by the attack of chloride anion at silicon of Si_2_Cl_6_ generating an equivalent tetrachlorosilane (SiCl_4_) and a reactive transient trichlorosilanide anion [:SiCl_3_]^−^ which then abstracts the remaining phosphorus bound chloride from intermediated **17** to generate the second and final equivalent of SiCl_4_.

**Figure 2:**
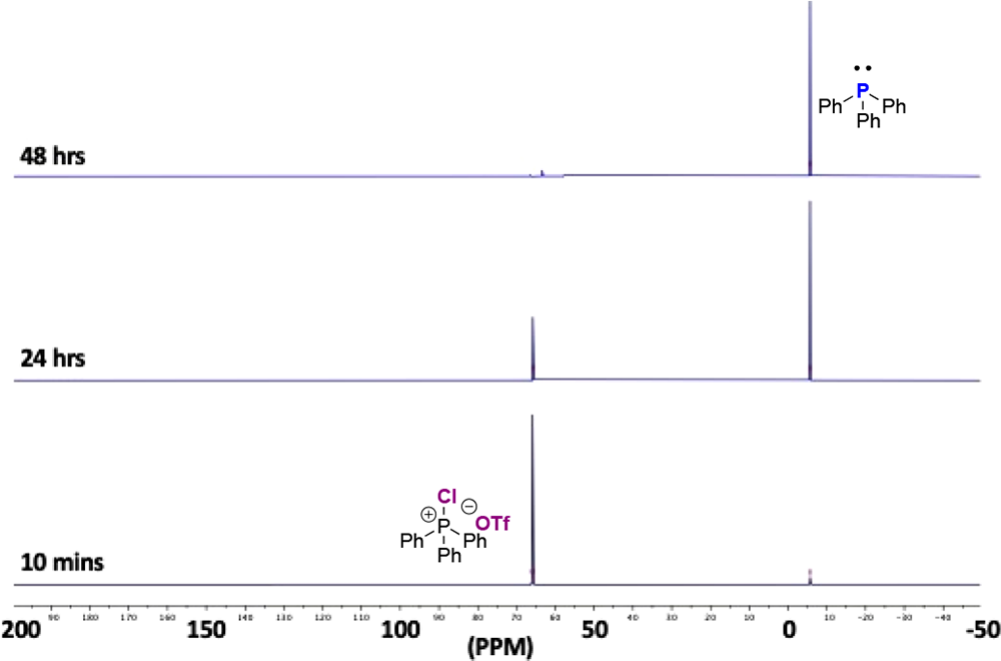
^31^P NMR (CD_2_Cl_2_) of Ph_3_PClOTf (**2b**) + Si_2_Cl_6_ → PPh_3_ (**3**). Reaction times = 10 min (bottom), 24 h (center), and 48 h (top).

To explore this mechanistic proposal, chlorotriphenylphosphonium triflate (Ph_3_PClOTf) **2b** was synthesized.^100^ The triflate anion is a superb nucleofuge, being a far more stable leaving group than chloride;^101^ therefore, the dissociated triflate ion (TfO^–^) of **2b** would be expected to react much slower with hexachlorodisilane than Cl^–^ of **2a**. Indeed, after reaction for 10 min, ^31^P NMR indicated **5b** had generated only 7% Ph_3_P **6**, progressing to 80% and 100% after 24 and 48 h, respectively (Table 1, entries 11–13; Figure 2), significantly slower than the dichloride analogue **2a** which appears to react instantly. As with **2a**, ^29^Si NMR analysis of the reaction mixture of monotriflate **2b** with Si_2_Cl_6_ showed the generated of SiCl_4_ (singlet at δ = −18.8 ppm) but in addition a singlet at δ = −38.2 ppm. ^13^C NMR spectra showed a quartet at δ = 118 ppm (*J* = 320 Hz) and ^19^F NMR a singlet at δ = −75.6 ppm; these signals are tentatively attributed to trichlorosilyl triflate, SiCl_3_OTf (see the Supporting Information). Finally, CPS **2c** bearing the non-nucleophilic tetrakis(3,5-dichlorophenyl)borate anion, [BAr^Cl^]^−^, was mixed with Si_2_Cl_6_ in methylene chloride. As anticipated, no triphenylphosphine **3** was formed, even with an excess of Si_2_Cl_6_, demonstrating that the reaction is initiated by the attack of a dissociated anion at silicon.

To gain further insight, quantum-chemical calculations employing the TURBOMOLE program were performed to study the thermodynamics and kinetics of the reaction. By use of the harmonic oscillator and rigid rotator approximation with a reference pressure of 1 bar, Gibbs free energies are given at the PBE0-D3/def2-TZVPP//PBE-D3/dhf-SV(P) level of theory.^102−109^ Our calculations show that the disproportionation of CPS into free phosphine with liberation of chlorine is uphill in free energy by 94 kJ/mol; similarly, formation of (unstabilized):SiCl_2_ by disproportionation of Si_2_Cl_6_ is also expected to be very unfavorable, Δ*G* = 107 kJ/mol. However, the formation of the free phosphine with Si_2_Cl_6_ releasing two SiCl_4_ molecules is thermodynamically favorable, Δ*G* = −246 kJ/mol (Scheme 3b,c).

**Table 3:**
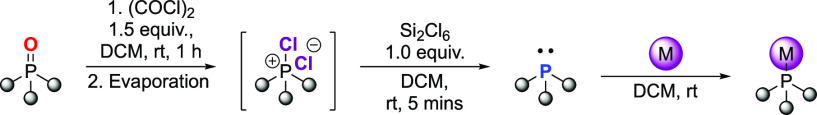

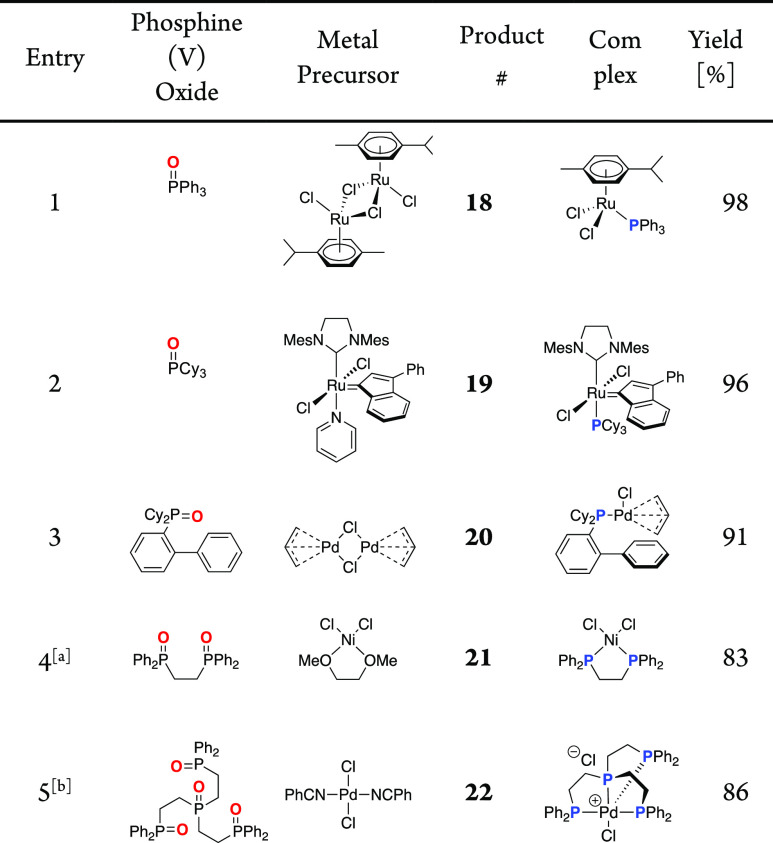
Conversion of Phosphine(V) Oxides to Their Corresponding Phosphine(III) Ligands and Metal Complexes

a Activated with 3.0 equiv of (COCl)_2_, deprotected with 2.1 equiv of Si_2_Cl_6_.

b Activated with 6.0 equiv of (COCl)_2_, deprotected with 4.1 equiv of Si_2_Cl_6_.

### A Telescoped Synthesis of Metal Complexes from Their Corresponding Phosphine(V) Oxides

With the new method of generating phosphine(III) ligands with high yield and purity in hand, we attempted to telescope^110^ the procedure for the synthesis of organometallic catalysts. As such, after deprotection and removal of SiCl_4_ by evaporation, “intermediate” phosphine(III) compounds were filtered through Celite and then reacted with a suitable metal precursor to yield a selection of prominent phosphine-bearing catalysts. The resultant monodentate triphenylphosphine, tricyclohexylphosphine, and CyJohnPhos were reacted with the dichloro(*p*-cymene)ruthenium(II) dimer, Umicore M31, and (η^3^-allyl)palladium(II) dichloride to afford the versatile dichloro(*p*-cymene)(triphenylphosphine)ruthenium(II) catalyst, **18**,^111^ olefin metathesis catalyst Umicore M2 (Grubbs catalyst M202), **19**,^112^ and the palladium Buchwald complex, CyJohnPhos(η^3^-allyl)PdCl, **20**,^113^ respectively, in excellent yields (91–98%). Moreover, the oxides of multidentate ligands where similarly reduced and successfully metalated, thus affording bidentate nickel **21**(114) and tetradentate palladium complexes **22**(115) in good yields of 83% and 86%, respectively.

## Conclusions

We have developed a simple mild one-pot activation/deprotection procedure in which phosphine(V) oxides are converted to their corresponding phosphine(III) ligands cleanly and efficiently at ambient temperature without the use of metals or the need for silica gel chromatography. The reduction of activated CPS **2** was investigated with a range of disilanes, and Si_2_Cl_6_ was demonstrated to be the best reductant. A reaction mechanism for the transformation has been elucidated through experimentation and supported by computation calculations, with the reduction being initiated by attack of the CPS’s dissociated chloride anion at the silicon of hexachlorodisilane. The new method was successfully applied to a range of aryl and alkyl phosphines, including state-of-the-art ligands, and found to be compatible with alkene, ether, and amine function groups. Challenging phosphine-bearing azolium salts were readily furnished. Furthermore, the high purity of resultant phosphine(III) compounds allowed the procedure to be telescoped for the formation of some prominent transition metal catalysts. We believe this research will facilitate the synthesis of both known and novel new phosphine(III) ligands as well as their corresponding complexes, while the catalytic use, reuse, or recycling of valuable phosphine(III)-based reagents is of importance for sustainability and is likely to be of only greater significance as increased demands or restrictions are placed upon finite phosphorus resources.^82−84^

## Supporting Information Available

Experimental procedures and characterization data (PDF)

## Accession Codes

CCDC 2023530–2023531 contain the supplementary crystallographic data for this paper. These data can be obtained free of charge *via*www.ccdc.cam.ac.uk/data_request/cif, or by emailing data_request@ccdc.cam.ac.uk, or by contacting The Cambridge Crystallographic Data Centre, 12 Union Road, Cambridge CB2 1EZ, UK; fax: +44 1223 336033.

## Footnotes

a We believe the deprotection occurs immediately, although 5 min had elapsed between addition of Si_2_Cl_6_ and acquisition of NMR data.

b In the case of Ph_3_P=O **1**, we were able perform a one-pot process without removal of excess oxalyl chloride *in vacou*: as little as 1.01 equiv of oxalyl chloride was reacted with **1** in dry degassed methylene chloride before 1.04 equiv of Si_2_Cl_6_ was added to the intermediate CPS **2**, thus completely converting **1** to Ph_3_P **3***in situ*. However, for expediency we decided use 1.5 equiv of oxalyl chloride and then strip the excess chlorinating reagent and solvent *in vacuo* before the CPS salt was once again dissolved in dry methylene chloride and deprotected with 1.04–1.10 equiv of Si_2_Cl_6_ (see the Supporting Information for details). It should be noted that residual oxalyl chloride appears to react vigorously with hexachlorodisilane leading to discoloration of phosphine(III) and even undesired byproducts.

c The molecule of HCl is likely to arise from oxalyl chloride.

## References

[ref1] Wang Z. Appel Reaction. In Comprehensive Organic Name Reactions and Reagents; American Cancer Society: 2010; pp 95–99.

[ref2] Fletcher S. The Mitsunobu Reaction in the 21 St Century. Org. Chem. Front. 2015, 2 (6), 739–752. 10.1039/C5QO00016E.

[ref3] Pommer H. The Wittig Reaction in Industrial Practice. Angew. Chem., Int. Ed. Engl. 1977, 16 (7), 423–429. 10.1002/anie.197704233.

[ref4] Eggersdorfer M.; Laudert D.; Létinois U.; McClymont T.; Medlock J.; Netscher T.; Bonrath W. One Hundred Years of Vitamins—A Success Story of the Natural Sciences. Angew. Chem., Int. Ed. 2012, 51 (52), 12960–12990. 10.1002/anie.201205886. 23208776

[ref5] Crabtree R. H. The Organometallic Chemistry of the Transition Metals, 6th ed.; Wiley: Hoboken, NJ, 2014.

[ref6] Ton S. J.; Fogg D. E. The Impact of Oxygen on Leading and Emerging Ru-Carbene Catalysts for Olefin Metathesis: An Unanticipated Correlation Between Robustness and Metathesis Activity. ACS Catal. 2019, 9 (12), 11329–11334. 10.1021/acscatal.9b03285.

[ref7] Borguet Y.; Sauvage X.; Zaragoza G.; Demonceau A.; Delaude L. Synthesis and Catalytic Evaluation in Olefin Metathesis of a Second-Generation Homobimetallic Ruthenium–Arene Complex Bearing a Vinylidene Ligand. Organometallics 2011, 30 (10), 2730–2738. 10.1021/om2001074.

[ref8] Fernández-Pérez H.; Etayo P.; Panossian A.; Vidal-Ferran A. Phosphine–Phosphinite and Phosphine–Phosphite Ligands: Preparation and Applications in Asymmetric Catalysis. Chem. Rev. 2011, 111 (3), 2119–2176. 10.1021/cr100244e. 21250669

[ref9] Musina E. I.; Balueva A. S.; Karasik A. A. Phosphines: Preparation, Reactivity and Applications. Organophosphorus Chemistry 2019, 48, 1–63. 10.1039/9781788016988-00001.

[ref10] Pietrusiewicz K. M.; Zablocka M. Preparation of Scalemic P-Chiral Phosphines and Their Derivatives. Chem. Rev. 1994, 94 (5), 1375–1411. 10.1021/cr00029a009.

[ref11] Imamoto T.; Kusumoto T.; Suzuki N.; Sato K. Phosphine Oxides and Lithium Aluminum Hydride-Sodium Borohydride-Cerium(III) Chloride: Synthesis and Reactions of Phosphine-Boranes. J. Am. Chem. Soc. 1985, 107 (18), 5301–5303. 10.1021/ja00304a061.

[ref12] Brill M.; Kühnel E.; Scriban C.; Rominger F.; Hofmann P. A Short and Modular Synthesis of Bulky and Electron-Rich N -Phosphinomethyl-Functionalised N-Heterocyclic Carbene Complexes. Dalton Trans. 2013, 42 (36), 12861–12864. 10.1039/c3dt51777b. 23903289

[ref13] Maier L. Organische Phosphorverbindungen XVI. Reduktion von Phosphinsulfiden Zu Den Entsprechenden Dreiwertigen Phosphorverbindungen. Helv. Chim. Acta 1964, 47 (8), 2137–2140. 10.1002/hlca.19640470806.

[ref14] Omelanczuk J.; Mikolajczyk M. Optically Active Trivalent Phosphorus Compounds. 2. Reactivity of Alkylthio- and Alkylselenophosphonium Salts. The First Stereospecific Synthesis of a Chiral Phosphinite. J. Am. Chem. Soc. 1979, 101 (24), 7292–7295. 10.1021/ja00518a026.

[ref15] Hérault D.; Hanh Nguyen D.; Nuel D.; Buono G. Reduction of Secondary and Tertiary Phosphine Oxides to Phosphines. Chem. Soc. Rev. 2015, 44 (8), 2508–2528. 10.1039/C4CS00311J. 25714261

[ref16] Kovacs T.; Keglevich G. The Reduction of Tertiary Phosphine Oxides by Silanes. Curr. Org. Chem. 2017, 21 (7), 569–585. 10.2174/1385272821666161108121532.

[ref17] Chrzanowski J.; Krasowska D.; Drabowicz J. Synthesis of Optically Active Tertiary Phosphine Oxides: A Historical Overview and the Latest Advances. Heteroat. Chem. 2018, 29 (5–6), e21476 10.1002/hc.21476.

[ref18] Corey E. J.; Chen Z.; Tanoury G. J. A New and Highly Enantioselective Synthetic Route to P-Chiral Phosphines and Diphosphines. J. Am. Chem. Soc. 1993, 115 (23), 11000–11001. 10.1021/ja00076a072.

[ref19] Salem H.; Schmitt M.; Herrlich (née Blumbach) U.; Kühnel E.; Brill M.; Nägele P.; Bogado A. L.; Rominger F.; Hofmann P. Bulky N-Phosphinomethyl-Functionalized N-Heterocyclic Carbene Chelate Ligands: Synthesis, Molecular Geometry, Electronic Structure, and Their Ruthenium Alkylidene Complexes. Organometallics 2013, 32 (1), 29–46. 10.1021/om300487r.

[ref20] Muci A. R.; Campos K. R.; Evans D. A. Enantioselective Deprotonation as a Vehicle for the Asymmetric Synthesis of C2-Symmetric P-Chiral Diphosphines. J. Am. Chem. Soc. 1995, 117 (35), 9075–9076. 10.1021/ja00140a028.

[ref21] Qiu L.; Kwong F. Y.; Wu J.; Lam W. H.; Chan S.; Yu W.-Y.; Li Y.-M.; Guo R.; Zhou Z.; Chan A. S. C. A New Class of Versatile Chiral-Bridged Atropisomeric Diphosphine Ligands: Remarkably Efficient Ligand Syntheses and Their Applications in Highly Enantioselective Hydrogenation Reactions. J. Am. Chem. Soc. 2006, 128 (17), 5955–5965. 10.1021/ja0602694. 16637664

[ref22] Fritzsche H.; Hasserodt U.; Korte F. Reduktion organischer Verbindungen des fünfwertigen Phosphors zu Phosphinen, II. Reduktion tertiärer Phosphinoxyde zu tertiären Phosphinen mit Trichlorsilan. Chem. Ber. 1965, 98 (1), 171–174. 10.1002/cber.19650980122.

[ref23] Horner L.; Balzer W. D. Phosphororganische verbindungen IXL zum sterischen verlauf der desoxygenierung von tertiären phosphinoxyden zu tertiären phosphinen mit trichlorsilan. Tetrahedron Lett. 1965, 6 (17), 1157–1162. 10.1016/S0040-4039(01)83990-3.

[ref24] Naumann K.; Zon G.; Mislow K. Use of Hexachlorodisilane as a Reducing Agent. Stereospecific Deoxygenation of Acyclic Phosphine Oxides. J. Am. Chem. Soc. 1969, 91 (25), 7012–7023. 10.1021/ja01053a021.

[ref25] Krenske E. H. Theoretical Investigation of the Mechanisms and Stereoselectivities of Reductions of Acyclic Phosphine Oxides and Sulfides by Chlorosilanes. J. Org. Chem. 2012, 77 (8), 3969–3977. 10.1021/jo300346g. 22497492

[ref26] Wu H.-C.; Yu J.-Q.; Spencer J. B. Stereospecific Deoxygenation of Phosphine Oxides with Retention of Configuration Using Triphenylphosphine or Triethyl Phosphite as an Oxygen Acceptor. Org. Lett. 2004, 6 (25), 4675–4678. 10.1021/ol048227c. 15575658

[ref27] Krenske E. H. Reductions of Phosphine Oxides and Sulfides by Perchlorosilanes: Evidence for the Involvement of Donor-Stabilized Dichlorosilylene | The Journal of Organic Chemistry. J. Org. Chem. 2012, 77 (1), 1–4. 10.1021/jo202370x. 22148631

[ref28] Gevorgyan A.; Mkrtchyan S.; Grigoryan T.; Iaroshenko V. O. Disilanes as Oxygen Scavengers and Surrogates of Hydrosilanes Suitable for Selective Reduction of Nitroarenes, Phosphine Oxides and Other Valuable Substrates. Org. Chem. Front. 2017, 4 (12), 2437–2444. 10.1039/C7QO00566K.

[ref29] Coumbe T.; Lawrence N. J.; Muhammad F. Titanium (IV) Catalysis in the Reduction of Phosphine Oxides. Tetrahedron Lett. 1994, 35 (4), 625–628. 10.1016/S0040-4039(00)75855-2.

[ref30] Marsi K. L. Stereochemistry of Some Reactions of Phospholane Derivatives. J. Am. Chem. Soc. 1969, 91 (17), 4724–4729. 10.1021/ja01045a025.

[ref31] Marsi K. L. Phenylsilane Reduction of Phosphine Oxides with Complete Stereospecificity. J. Org. Chem. 1974, 39 (2), 265–267. 10.1021/jo00916a041.

[ref32] Schirmer M.-L.; Jopp S.; Holz J.; Spannenberg A.; Werner T. Organocatalyzed Reduction of Tertiary Phosphine Oxides. Adv. Synth. Catal. 2016, 358 (1), 26–29. 10.1002/adsc.201500762.

[ref33] Li Y.; Das S.; Zhou S.; Junge K.; Beller M. General and Selective Copper-Catalyzed Reduction of Tertiary and Secondary Phosphine Oxides: Convenient Synthesis of Phosphines. J. Am. Chem. Soc. 2012, 134 (23), 9727–9732. 10.1021/ja301764m. 22480270

[ref34] Fritzsche H.; Hasserodt U.; Korte F. Reduktion organischer Verbindungen des fünfwertigen Phosphors zu Phosphinen, I. Reduktion tertiärer Phosphinoxyde zu tertiären Phosphinen mit Silanen. Chem. Ber. 1964, 97 (7), 1988–1993. 10.1002/cber.19640970729.

[ref35] Nicolas E.; Guerriero A.; Lyaskovskyy V.; Peruzzini M.; Lammertsma K.; Gonsalvi L.; Slootweg J. C. Metal-Free Reduction of Phosphine Oxides Using Polymethylhydrosiloxane. Inorganics 2016, 4 (4), 34 10.3390/inorganics4040034.

[ref36] Buonomo J. A.; Eiden C. G.; Aldrich C. C. Chemoselective Reductions of Phosphine Oxides by 1,3-Diphenyl-Disiloxane. Chem. - Eur. J. 2017, 23 (58), 14434–14438. 10.1002/chem.201703875. 28840623 PMC5647249

[ref37] Li Y.; Lu L.-Q.; Das S.; Pisiewicz S.; Junge K.; Beller M. Highly Chemoselective Metal-Free Reduction of Phosphine Oxides to Phosphines. J. Am. Chem. Soc. 2012, 134 (44), 18325–18329. 10.1021/ja3069165. 23062083

[ref38] Hein F.; Issleib K.; Rabold H. Über die Reduktion von tertiären Phosphinoxyden bzw. -sulfiden mit Lithium- bzw. Calciumalanat zu den entsprechenden Phosphinen. Z. Anorg. Allg. Chem. 1956, 287 (4–6), 208–213. 10.1002/zaac.19562870406.

[ref39] Henson P. D.; Naumann K.; Mislow K. Stereomutation of Phosphine Oxides by Lithium Aluminum Hydride. J. Am. Chem. Soc. 1969, 91 (20), 5645–5646. 10.1021/ja01048a041.

[ref40] Imamoto T.; Takeyama T.; Kusumoto T. Facile Reduction of Organic Halides and Phosphine Oxides with LiAlH4–CeCl3. Chem. Lett. 1985, 14 (10), 1491–1492. 10.1246/cl.1985.1491.

[ref41] Griffin S.; Heath L.; Wyatt P. Alane — A Novel Way to Reduce Phosphine Oxides. Tetrahedron Lett. 1998, 39 (24), 4405–4406. 10.1016/S0040-4039(98)00748-5.

[ref42] Busacca C. A.; Raju R.; Grinberg N.; Haddad N.; James-Jones P.; Lee H.; Lorenz J. C.; Saha A.; Senanayake C. H. Reduction of Tertiary Phosphine Oxides with DIBAL-H. J. Org. Chem. 2008, 73 (4), 1524–1531. 10.1021/jo7024064. 18197688

[ref43] Handa Y.; Inanaga J.; Yamaguchi M. Rapid and Mild Deoxygenation of Organoheteroatom Oxides with the Efficient Electron Transfer System SmI 2 – Tetrahydrofuran–Hexamethylphosphoric Triamide. J. Chem. Soc., Chem. Commun. 1989, 0 (5), 298–299. 10.1039/C39890000298.

[ref44] Mathey F.; Maillet R. Reduction Des Oxydes de Phosphines Par Le Systeme Cp2TiCl2-Mg. Tetrahedron Lett. 1980, 21 (26), 2525–2526. 10.1016/0040-4039(80)80117-1.

[ref45] Dockner T. Reduction and Hydrogenation with the System Hydrocarbon/Carbon [New Synthetic Methods (70)]. Angew. Chem., Int. Ed. Engl. 1988, 27 (5), 679–682. 10.1002/anie.198806791.

[ref46] Elias J. S.; Costentin C.; Nocera D. G. Direct Electrochemical P(V) to P(III) Reduction of Phosphine Oxide Facilitated by Triaryl Borates. J. Am. Chem. Soc. 2018, 140 (42), 13711–13718. 10.1021/jacs.8b07149. 30278122

[ref47] Chakraborty B.; Menezes P. W.; Driess M. Beyond CO_2_ Reduction: Vistas on Electrochemical Reduction of Heavy Non-Metal Oxides with Very Strong E—O Bonds (E = Si, P, S). J. Am. Chem. Soc. 2020, 142 (35), 14772–14788. 10.1021/jacs.0c05862. 32786773

[ref48] Manabe S.; Wong C. M.; Sevov C. S. Direct and Scalable Electroreduction of Triphenylphosphine Oxide to Triphenylphosphine. J. Am. Chem. Soc. 2020, 142 (6), 3024–3031. 10.1021/jacs.9b12112. 31948233

[ref49] Laven G.; Kullberg M. A Process for the Reduction of a Tertiary Phosphine Oxide to the Corresponding Tertiary Phosphine in the Presence of a Catalyst and Use of a Tertiary Phosphine for Reducing a Tertiary Phosphine Oxide in the Presence of a Catalyst. WO2011123037 (A1), October 6, 2011.

[ref50] Li P.; Wischert R.; Métivier P. Mild Reduction of Phosphine Oxides with Phosphites To Access Phosphines. Angew. Chem., Int. Ed. 2017, 56 (50), 15989–15992. 10.1002/anie.201709519. 28994175

[ref51] Quin L. D.; Rao N. S. 1-Phenyl-Cis-3a,7a-Dihydrophosphindole and Its Properties. J. Org. Chem. 1983, 48 (21), 3754–3759. 10.1021/jo00169a029.

[ref52] Higham L. J.; Clarke E. F.; Müller-Bunz H.; Gilheany D. G. P-Chirogenic Phosphines. MOP/DiPAMP Hybrids, Their Oxide Crystal Structures, Reduction Studies and Alternative Syntheses. J. Organomet. Chem. 2005, 690 (1), 211–219. 10.1016/j.jorganchem.2004.09.015.

[ref53] Henson P. D.; Ockrymiek S. B.; Markham R. E. Reductive Cleavage of Phosphinanilides with Lithium Aluminum Hydride. J. Org. Chem. 1974, 39 (15), 2296–2298. 10.1021/jo00929a039.

[ref54] Quin L. D.; Keglevich G. Stereochemistry of the Reaction of Oxygen Nucleophiles with a Phosphinous Chloride in the 7-Phosphanorbornene Series. J. Chem. Soc., Perkin Trans. 2 1986, 2 (7), 1029–1034. 10.1039/p29860001029.

[ref55] Szewczyk J.; Quin L. D. Stereochemical Studies with Aminophosphines and Related Compounds Having the 7-Phosphanorbornene Structure. J. Org. Chem. 1987, 52 (7), 1190–1196. 10.1021/jo00383a003.

[ref56] Kuroboshi M.; Kita T.; Aono A.; Katagiri T.; Kikuchi S.; Yamane S.; Kawakubo H.; Tanaka H. Reduction of Phosphine Oxides to the Corresponding Phosphine Derivatives in Mg/Me3SiCl/DMI System. Tetrahedron Lett. 2015, 56 (7), 918–920. 10.1016/j.tetlet.2014.12.132.

[ref57] Horner L.; Hoffmann H.; Beck P. Phosphororganische Verbindungen, XVI. Wege zur Darstellung primärer, sekundärer und tertiärer Phosphine. Chem. Ber. 1958, 91 (8), 1583–1588. 10.1002/cber.19580910803.

[ref58] Horner L.; Beck P.; Hoffmann H. Phosphororganische Verbindungen, XIX. Reduktion von Phosphorverbindungen mit Alkalimetallen. Chem. Ber. 1959, 92 (9), 2088–2094. 10.1002/cber.19590920920.

[ref59] Rajendran K. V.; Kennedy L.; O’Connor C. T.; Bergin E.; Gilheany D. G. Systematic Survey of Positive Chlorine Sources in the Asymmetric Appel Reaction: Oxalyl Chloride as a New Phosphine Activator. Tetrahedron Lett. 2013, 54 (51), 7009–7012. 10.1016/j.tetlet.2013.10.044.

[ref60] Yano T.; Hoshino M.; Kuroboshi M.; Tanaka H. A Practical One-Pot Transformation of Triphenylphosphine Oxide to Triphenylphosphine by Reduction of in Situ Generated Triphenylphosphine Dichloride. Synlett 2010, 2010 (5), 801–803. 10.1055/s-0029-1219194.

[ref61] Byrne P. A.; Rajendran K. V.; Muldoon J.; Gilheany D. G. A Convenient and Mild Chromatography-Free Method for the Purification of the Products of Wittig and Appel Reactions. Org. Biomol. Chem. 2012, 10 (17), 3531–3537. 10.1039/c2ob07074j. 22437367

[ref62] Carr D. J.; Kudavalli J. S.; Dunne K. S.; Müller-Bunz H.; Gilheany D. G. Synthesis of 2,3-Dihydro-1-Phenylbenzo[b]Phosphole (1-Phenylphosphindane) and Its Use as a Mechanistic Test in the Asymmetric Appel Reaction: Decisive Evidence against Involvement of Pseudorotation in the Stereoselecting Step. J. Org. Chem. 2013, 78 (20), 10500–10505. 10.1021/jo401318g. 24079276

[ref63] Masaki M.; Fukui K. Reaction of Tertiary Phosphine Dichlorides with Thiols in the Presence of Triethylamine. a Convenient Method for the Reduction of Phosphine Oxides to Phosphines. Chem. Lett. 1977, 6 (2), 151–152. 10.1246/cl.1977.151.

[ref64] Zhang T.-X.; Zhang W.-X.; Luo M.-M. Metal-Free Reduction of Tertiary Phosphine Oxides with Hantzsch Ester. Chin. Chem. Lett. 2014, 25 (1), 176–178. 10.1016/j.cclet.2013.09.007.

[ref65] Yano T.; Kuroboshi M.; Tanaka H. Electroreduction of Triphenylphosphine Dichloride and the Efficient One-Pot Reductive Conversion of Phosphine Oxide to Triphenylphosphine. Tetrahedron Lett. 2010, 51 (4), 698–701. 10.1016/j.tetlet.2009.11.115.

[ref66] Kuroboshi M.; Yano T.; Kamenoue S.; Kawakubo H.; Tanaka H. Electroreduction of Tetra-Coordinate Phosphonium Derivatives; One-Pot Transformation of Triphenylphosphine Oxide into Triphenylphosphine. Tetrahedron 2011, 67 (32), 5825–5831. 10.1016/j.tet.2011.05.044.

[ref67] Bassler P.; Hammes P.; Hermeling D.; Hugo R.; Lechtken P.; Siegel H. Preparation of Triphenylphosphine. US5527966A, June 18, 1996.

[ref68] Hugo R.; Bassler P. D.; Hammes P. D.; Hermeling D. D.; Lechtken P. D.; Siegel H. D. Process for the Preparation of Triphenyl Phosphine. EP0638580B1, December 30, 1998.

[ref69] Wettling T. D. Process for the Preparation of Tertiary Phosphines. EP0548682B1, November 6, 1996.

[ref70] Masaki M.; Kakeya N. Hydrogenolysis of Trisubstituted Dichlorophosphoranes—A New Method for Deoxygenation of Oxophosphoranes. Angew. Chem., Int. Ed. Engl. 1977, 16 (8), 552–553. 10.1002/anie.197705522.

[ref71] Stepen A. J.; Bursch M.; Grimme S.; Stephan D. W.; Paradies J. Electrophilic Phosphonium Cation-Mediated Phosphane Oxide Reduction Using Oxalyl Chloride and Hydrogen. Angew. Chem., Int. Ed. 2018, 57 (46), 15253–15256. 10.1002/anie.201809275. 30230149

[ref72] Zhu H.; Qu Z.-W.; Grimme S. Reduction of Phosphine Oxide by Using Chlorination Reagents and Dihydrogen: DFT Mechanistic Insights. Chem. - Eur. J. 2019, 25 (18), 4670–4672. 10.1002/chem.201900379. 30758069

[ref73] Vetter A. C.; Nikitin K.; Gilheany D. G. Long Sought Synthesis of Quaternary Phosphonium Salts from Phosphine Oxides: Inverse Reactivity Approach. Chem. Commun. 2018, 54 (46), 5843–5846. 10.1039/C8CC02173B. 29714382

[ref74] Rajendran K. V.; Gilheany D. G. Simple Unprecedented Conversion of Phosphine Oxides and Sulfides to Phosphine Boranes Using Sodium Borohydride. Chem. Commun. 2012, 48 (6), 817–819. 10.1039/C1CC14856G. 22022704

[ref75] Kenny N. P.; Rajendran K. V.; Jennings E. V.; Gilheany D. G. Cleavage of P=O in the Presence of P–N: Aminophosphine Oxide Reduction with In Situ Boronation of the PIII Product. Chem. - Eur. J. 2013, 19 (42), 14210–14214. 10.1002/chem.201302907. 24027002

[ref76] Al Sulaimi S. S.; Rajendran K. V.; Gilheany D. G. Lithium Borohydride for Achiral and Stereospecific Reductive Boronation at Phosphorus: Lack of Electronic Effects on Stereoselective Formation of Alkoxyphosphonium Salts. Eur. J. Org. Chem. 2015, 2015 (27), 5959–5965. 10.1002/ejoc.201500521.

[ref77] Herbay R.; Bagi P.; Fogassy E.; Keglevich G. Preparation of P-Heterocyclic Phosphine Boranes and Optically Active Phosphine Oxides *via* Phosphonium Salts. Phosphorus, Sulfur Silicon Relat. Elem. 2016, 191 (11–12), 1656–1657. 10.1080/10426507.2016.1225060.

[ref78] Herbay R.; Bagi P.; Mucsi Z.; Mátravölgyi B.; Drahos L.; Fogassy E.; Keglevich G. A Novel Preparation of Chlorophospholenium Chlorides and Their Application in the Synthesis of Phospholene Boranes. Tetrahedron Lett. 2017, 58 (5), 458–461. 10.1016/j.tetlet.2016.12.059.

[ref79] Bagi P.; Herbay R.; Ábrányi-Balogh P.; Mátravölgyi B.; Fogassy E.; Keglevich G. Dynamic Kinetic Resolution of 1-Substituted-3-Methyl-3-Phospholene Oxides *via* the Formation of Diastereomeric Alkoxyphospholenium Salts. Tetrahedron 2018, 74 (40), 5850–5857. 10.1016/j.tet.2018.07.058.

[ref80] Weskamp T.; Böhm V. P. W.; Herrmann W. A. Combining N-Heterocyclic Carbenes and Phosphines: Improved Palladium(II) Catalysts for Aryl Coupling Reactions. J. Organomet. Chem. 1999, 585 (2), 348–352. 10.1016/S0022-328X(99)00237-5.

[ref81] Hofmann P.; Brill M. NHCP Ligands for Catalysis. In Molecular Catalysts; John Wiley & Sons, Ltd.: 2014; pp 207–234.

[ref82] Withers P. J. A.; Elser J. J.; Hilton J.; Ohtake H.; Schipper W. J.; van Dijk K. C. Greening the Global Phosphorus Cycle: How Green Chemistry Can Help Achieve Planetary P Sustainability. Green Chem. 2015, 17 (4), 2087–2099. 10.1039/C4GC02445A.

[ref83] Withers P. J. A. Closing the Phosphorus Cycle. Nat. Sustain. 2019, 2 (11), 1001–1002. 10.1038/s41893-019-0428-6.

[ref84] Keijer T.; Bakker V.; Slootweg J. C. Circular Chemistry to Enable a Circular Economy. Nat. Chem. 2019, 11 (3), 190–195. 10.1038/s41557-019-0226-9. 30792512

[ref85] Nikitin K.; Jennings E. V.; Al Sulaimi S.; Ortin Y.; Gilheany D. G. Dynamic Cross-Exchange in Halophosphonium Species: Direct Observation of Stereochemical Inversion in the Course of an SN2 Process. Angew. Chem., Int. Ed. 2018, 57 (6), 1480–1484. 10.1002/anie.201708649. 29149539

[ref86] Enthaler S.; Erre G.; Junge K.; Michalik D.; Spannenberg A.; Marras F.; Gladiali S.; Beller M. Enantioselective Rhodium-Catalyzed Hydrogenation of Enol Carbamates in the Presence of Monodentate Phosphines. Tetrahedron: Asymmetry 2007, 18 (11), 1288–1298. 10.1016/j.tetasy.2007.06.001.

[ref87] Kalek M.; Fu G. C. Phosphine-Catalyzed Doubly Stereoconvergent γ-Additions of Racemic Heterocycles to Racemic Allenoates: The Catalytic Enantioselective Synthesis of Protected α,α-Disubstituted α-Amino Acid Derivatives. J. Am. Chem. Soc. 2015, 137 (29), 9438–9442. 10.1021/jacs.5b05528. 26192217 PMC4577964

[ref88] Zeng Q.; Zeng H.; Yang Z. New Route for Synthesis of MeO-MOP. Synth. Commun. 2011, 41 (23), 3556–3560. 10.1080/00397911.2010.519095.

[ref89] Al-Juboori M. A. H. A.; Gates P. N.; Muir A. S. Ionic–Molecular Isomerism in Chlorophenylphosphoranes Ph n PCl 5 – n (1 ⩽ n ⩽ 3). J. Chem. Soc., Chem. Commun. 1991, 0 (18), 1270–1271. 10.1039/C39910001270.

[ref90] Godfrey S. M.; McAuliffe C. A.; Sheffield J. M. Structural Dependence of the Reagent Ph3PCl2 on the Nature of the Solvent, Both in the Solid State and in Solution; X-Ray Crystal Structure of Trigonal Bipyramidal Ph3PCl2, the First Structurally Characterised Five-Coordinate R3PCl2 Compound. Chem. Commun. 1998, 8, 921–922. 10.1039/a800820e.

[ref91] Godfrey S. M.; Hinchliffe A.; Mkadmh A. Ab Initio Studies on the Reagent Ph3PCl2. J. Mol. Struct.: THEOCHEM 2005, 719 (1), 85–88. 10.1016/j.theochem.2005.01.027.

[ref92] Nikitin K.; Müller-Bunz H.; Gilheany D. Direct Evidence of a Multicentre Halogen Bond: Unexpected Contraction of the P–XXX–P Fragment in Triphenylphosphine Dihalides. Chem. Commun. 2013, 49 (14), 1434–1436. 10.1039/c2cc38363b. 23322232

[ref93] Vetter A. C.; Nikitin K.; Gilheany D. G. Exploring an Umpolung Strategy for Quaternization of Phosphorus. Phosphorus, Sulfur Silicon Relat. Elem. 2019, 194 (4–6), 339–342. 10.1080/10426507.2018.1541242.

[ref94] Wilkins C. J. 682. The Reactions of Hexachlorodisilane with Ammonium Halides and Trimethylamine Hydrohalides. J. Chem. Soc. 1953, 3409–3412. 10.1039/jr9530003409.

[ref95] Cooper G. D.; Gilbert A. R. Cleavage and Disproportionation of Polychlorodisilanes, Trichloromethylchlorosilanes and Hexachlorodisiloxane by Amines and Ammonium Salts. J. Am. Chem. Soc. 1960, 82 (19), 5042–5044. 10.1021/ja01504a008.

[ref96] Tillmann J.; Meyer L.; Schweizer J. I.; Bolte M.; Lerner H.-W.; Wagner M.; Holthausen M. C. Chloride-Induced Aufbau of Perchlorinated Cyclohexasilanes from Si2Cl6: A Mechanistic Scenario. Chem. - Eur. J. 2014, 20 (30), 9234–9239. 10.1002/chem.201402655. 24771318

[ref97] Teichmann J.; Bursch M.; Köstler B.; Bolte M.; Lerner H.-W.; Grimme S.; Wagner M. Trapping Experiments on a Trichlorosilanide Anion: A Key Intermediate of Halogenosilane Chemistry. Inorg. Chem. 2017, 56 (15), 8683–8688. 10.1021/acs.inorgchem.7b00216. 28318239

[ref98] Georg I.; Teichmann J.; Bursch M.; Tillmann J.; Endeward B.; Bolte M.; Lerner H.-W.; Grimme S.; Wagner M. Exhaustively Trichlorosilylated C1 and C2 Building Blocks: Beyond the Müller–Rochow Direct Process. J. Am. Chem. Soc. 2018, 140 (30), 9696–9708. 10.1021/jacs.8b05950. 29985607

[ref99] Teichmann J.; Wagner M. Silicon Chemistry in Zero to Three Dimensions: From Dichlorosilylene to Silafullerane. Chem. Commun. 2018, 54 (12), 1397–1412. 10.1039/C7CC09063C. 29359765

[ref100] Hwang S. J.; Powers D. C.; Maher A. G.; Nocera D. G. Tandem Redox Mediator/Ni(II) Trihalide Complex Photocycle for Hydrogen Evolution from HCl. Chem. Sci. 2015, 6 (2), 917–922. 10.1039/C4SC02357A. 29560177 PMC5811117

[ref101] Dhakal B.; Bohé L.; Crich D. Trifluoromethanesulfonate Anion as Nucleophile in Organic Chemistry. J. Org. Chem. 2017, 82 (18), 9263–9269. 10.1021/acs.joc.7b01850. 28858509 PMC5600715

[ref102] Schäfer A.; Horn H.; Ahlrichs R. Fully Optimized Contracted Gaussian Basis Sets for Atoms Li to Kr. J. Chem. Phys. 1992, 97 (4), 2571–2577. 10.1063/1.463096.

[ref103] Schäfer A.; Huber C.; Ahlrichs R. Fully Optimized Contracted Gaussian Basis Sets of Triple Zeta Valence Quality for Atoms Li to Kr. J. Chem. Phys. 1994, 100 (8), 5829–5835. 10.1063/1.467146.

[ref104] Eichkorn K.; Treutler O.; Öhm H.; Häser M.; Ahlrichs R. Auxiliary Basis Sets to Approximate Coulomb Potentials. Chem. Phys. Lett. 1995, 240 (4), 283–290. 10.1016/0009-2614(95)00621-A.

[ref105] Perdew J. P.; Burke K.; Ernzerhof M. Generalized Gradient Approximation Made Simple. Phys. Rev. Lett. 1996, 77 (18), 3865–3868. 10.1103/PhysRevLett.77.3865. 10062328

[ref106] Weigend F.; Häser M.; Patzelt H.; Ahlrichs R. RI-MP2: Optimized Auxiliary Basis Sets and Demonstration of Efficiency. Chem. Phys. Lett. 1998, 294 (1), 143–152. 10.1016/S0009-2614(98)00862-8.

[ref107] Weigend F.; Ahlrichs R. Balanced Basis Sets of Split Valence, Triple Zeta Valence and Quadruple Zeta Valence Quality for H to Rn: Design and Assessment of Accuracy. Phys. Chem. Chem. Phys. 2005, 7 (18), 3297–3305. 10.1039/b508541a. 16240044

[ref108] Weigend F. Accurate Coulomb-Fitting Basis Sets for H to Rn. Phys. Chem. Chem. Phys. 2006, 8 (9), 1057–1065. 10.1039/b515623h. 16633586

[ref109] Grimme S.; Antony J.; Ehrlich S.; Krieg H. A Consistent and Accurate Ab Initio Parametrization of Density Functional Dispersion Correction (DFT-D) for the 94 Elements H-Pu. J. Chem. Phys. 2010, 132 (15), 154104 10.1063/1.3382344. 20423165

[ref110] Hayashi Y. Pot Economy and One-Pot Synthesis. Chem. Sci. 2016, 7 (2), 866–880. 10.1039/C5SC02913A. 28791118 PMC5529999

[ref111] Fürstner A.; Liebl M.; Lehmann C. W.; Picquet M.; Kunz R.; Bruneau C.; Touchard D.; Dixneuf P. H. Cationic Ruthenium Allenylidene Complexes as Catalysts for Ring Closing Olefin Metathesis. Chem. - Eur. J. 2000, 6 (10), 1847–1857. 10.1002/(SICI)1521-3765(20000515)6:10<1847::AID-CHEM1847>3.0.CO;2-1. 10845645

[ref112] Boeda F.; Clavier H.; Nolan P. S. Ruthenium – Indenylidene Complexes: Powerful Tools for Metathesis Transformations. Chem. Commun. 2008, 0 (24), 2726–2740. 10.1039/b718287b. 18688294

[ref113] Kisanga P.; Widenhoefer R. A. Development, Synthetic Scope, and Mechanistic Studies of the Palladium-Catalyzed Cycloisomerization of Functionalized 1,6-Dienes in the Presence of Silane. J. Am. Chem. Soc. 2000, 122 (41), 10017–10026. 10.1021/ja001730+.

[ref114] Jarrett P. S.; Sadler P. J. Nickel(II) Bis(Phosphine) Complexes. Inorg. Chem. 1991, 30 (9), 2098–2104. 10.1021/ic00009a028.

[ref115] Aizawa S.; Iida T.; Funahashi S. Mechanistic Studies on Halo-Ligand Substitution of Five-Coordinate Trigonal-Bipyramidal Palladium(II) Complexes of Tris(2-(Diphenylphosphino)Ethyl)Phosphine with Trimethyl Phosphite in Chloroform at Various Temperatures and Pressures. Inorg. Chem. 1996, 35 (18), 5163–5167. 10.1021/ic9601596.

