# Supplementary material for: A Mild One-Pot Reduction of Phosphine(V) Oxides Affording Phosphines(III) and Their Metal Catalysts
Source: Organometallics. 2021 Mar 5;40(6):693–701. doi: 10.1021/acs.organomet.0c00788 (PMC8043083; doi:10.1021/acs.organomet.0c00788)
Supplement: Supplementary file 1 — om0c00788_si_001.pdf [file om0c00788_si_001.pdf]

Supplementary Information
A Mild One-pot Reduction of Phosphine(V) Oxides Affording Phosphines(III) and their Metal Catalysts

Łukasz Kapuśniak,[a] Philipp N. Plessow,[b] Damian Trzybiński,[a] Krzysztof Woźniak,[a] Peter Hofmann[c][d] and Phillip Iain Jolly*[a][d]

[a] *Biological and Chemical Research Centre, Faculty of Chemistry, University of Warsaw, Żwirki i Wigury Street 101, 02-089 Warsaw, Poland*

[b] *Institute of Catalysis Research and Technology, Karlsruhe Institute of Technology, Hermann-von-Helmholtz-Platz 1, D-76344 Eggenstein-Leopoldshafen, Germany*

[c] *Organisch-Chemisches Institut, Heidelberg University, Im Neuenheimer Feld 270, 69120 Heidelberg, Germany*

[d] *Catalysis Research Laboratory (CaRLa), Im Neuenheimer Feld 584, 69120 Heidelberg, Germany*

Page: **Content:**

S3 **General Considerations:**

S4 **Table, S1:** showing a compounds origin or reference for its literature synthesis

S5 **Preparation of chlorotriphenyl-λ^4-phosphanes:**

S5 Dichlorotriphenyl-λ^4-phosphane, **2a**:[1]

S5 Chlorotriphenyl-λ^4-phosphane trifluoromethanesulfate, **2b**[2]

S6 Chlorotriphenyl-λ^4-phosphane tetrakis(3,5-dichlorophenyl)borate, **2c**

S6 **Disilane screening:**

S6 Reaction of dichlorotriphenyl-λ^4-phosphane, **2a** with Hexachlorodisilane:

S8 Reaction of dichlorotriphenyl-λ^4-phosphane, **2a** with 1,1,2,2-tetrachloro-1,2-dimethyldisilane.

S9 Reaction of dichlorotriphenyl-λ^4-phosphane, **2a** with hexamethyldisilane.

S10 Reaction of dichlorotriphenyl-λ^4-phosphane, **2a** with hexaphenyldisilane.

S11 Reaction of Hexachlorodisilane with chlorotriphenyl-λ^4-phosphane trifluoromethanesulfate, **2b**.

S14 Reaction of Hexachlorodisilane with chlorotriphenyl-λ^4-phosphane tetrakis(3,5-dichlorophenyl)borate, **2c**.

S15 **One-pot procedure for converting phosphine(V) oxides phosphine(III) *via* chlorophosphonium salts:**

S15 General Procedure 1: 1.05 -5.00 equivalents of oxalyl chloride

S16 General Procedure 1: 1.01-1.05 equivalents of oxalyl chloride

S16 Synthesis of Triphenylphosphine (*via* dichlorotriphenyl-λ^4-phosphane), **3**:[3]

S16 Synthesis of Tricyclohexylphosphine (*via* dichlorotricyclohexyl-λ^4-phosphane), **7**.

S17 Synthesis of 4-methyl-1-phenyl-2,3-dihydro-1H-phosphole[4] (*via* 1-chloro-4-methyl-1-phenyl-2,3-dihydro-1H-phosphol-1-ium chloride), **8.**[1]

S17 Synthesis of *N,N*-diisopropyl-1,1-diphenylphosphanamine[5] (*via* chloro(diisopropylamino)diphenyl-phosphonium chloride, **9:**[5]

S17 Synthesis of [1,1'-biphenyl]-2-yldicyclohexylphosphane, CyJohnPhos[3a] (*via* 1-Chloro-3-methyl-1-phenyl-2-phospholenium chloride), **10**.

S18 Synthesis of 2-Dicyclohexylphosphino-2′-(*N,N*-dimethylamino)biphenyl, (DavePhos)[6] (*via* 2'-(dichlorodicyclohexyl- λ^5-phosphaneyl)-*N,N*-dimethyl-[1,1'-biphenyl]-2-amine, **11**.

S18 Synthesis of (*S*)-Ph-BINEPINE[7] (*via* 4-chloro-4-phenyl-4,5-dihydro-3H-dinaphtho[2,1-c:1',2'-e]phosphepin-4-ium), **12**.

S19 Synthesis of (*R*)-(+)-2-(Diphenylphosphino)-2′-methoxy-1,1′-binaphthyl[8] (*via* 4-chloro-4-phenyl-4,5-dihydro-3H-dinaphtho[2,1-c:1',2'-e]phosphepin-4-ium chloride), **13**.

S19 Synthesis of 3-((di-*tert*-butylphosphoryl)methyl)-1-mesityl-1*H*-imidazol-3-ium 4-methylbenzenesulfonate, **4.**[9]

S19 Synthesis of 3-((di-*tert*-butyldichloro- λ^4-phosphaneyl)methyl)-1-mesityl-1*H*-imidazol-3-ium chloride, **15**.

S20 Synthesis of 3-((di-*tert*-butylphosphaneyl)methyl)-1-mesityl-1*H*-imidazol-3-ium chloride**, 5**.[9]

S20 Synthesis of 1-((di-*tert*-butylphosphoryl)methyl)-3-(2,6-diisopropylphenyl)-1*H*-imidazol-3-ium 4-methylbenzenesulfonate.

S21 Synthesis of mono(1-((di-*tert*-butylchloro-λ^4-phosphaneyl)methyl)-3-(2,6-diisopropylphenyl)-1*H*-imidazol-3-ium) dichloride.

S21 Synthesis of 1-((di-*tert*-butylphosphaneyl)methyl)-3-(2,6-diisopropylphenyl)-1*H*-imidazol-3-ium chloride, **14**.

S22 **A telescoped synthesis of metal complexes from their corresponding phosphine(V) oxides:**

S22 Synthesis of Dichloro(η^6-*p*-cymene)(triphenylphosphine)ruthenium(II), **18**:[10]

S23 Synthesis of Umicore M2 (Grubbs Catalyst® M202) - [1,3-Bis(2,4,6-trimethylphenyl)-2-imidazolidinylidene] dichloro(3-phenyl-1*H*-inden-1-ylidene)(tricyclohexylphosphine) ruthenium(II), **19**:[11]

S23 Synthesis of CyJohnPhos(η3-allyl)PdCl, **20**:[12]

S23 Synthesis of (dppe)$NiCl_2$, **21.**[13]

S24 Synthesis of [Pd(P4)Cl]Cl, **22.**[14]

S24 **Computation**

S25 **NMR Spectra:**

For phosphine(V) oxides, chlorophosphonium salt, phosphines(III) and metal complexes.

S58 **Synthetic Reference**

S58 **Single-crystal X-ray diffraction analysis report (CPS 15 and Azolium 5)**

S66 **X-ray Reference**

General Considerations:

Unless otherwise stated, all reactions were carried out under argon atmosphere in oven dried glassware (overnight, 135 °C) equipped with a magnetic stirrer on the Schlenk line or in the glovebox. Solvents were purified by Solvent Purification System, Mbraun MB-SPS-800. Infrared (IR) spectra were recorded on a Perkin-Elmer Spectrum One FTIR spectrometer with diamond ATR accessory, wave numbers are in cm ^{1}, following abbreviations are employed to describe signal intensity: broad (br), weak (w), moderate (m) and strong (s). Elemental Analyses (EA) were provided by the EA analytical laboratory at the Institute of Organic Chemistry, Polish Academy of Sciences (PAS). High Resolution Mass Spectra (HRMS) were provided by the Faculty of Chemistry University of Warsaw or analytical laboratory at the Institute of Organic Chemistry, PAS. NMR spectra were recorded on an Agilent 400-MR DD2 400 MHz spectrometer. NMR chemical shifts are reported in ppm downfield from solvent residual peak (7.26 and 77.16 ppm for ^{1}H and ^{13}C in $CDCl_3$, 5.32 and 54.00 ppm for ^{1}H and ^{13}C in CD_2Cl_2). Data are reported as follows: chemical shift, multiplicity (s: singlet, d: doublet, t: triplet, q: quartet, qui: quintuplet, m: multiplet), coupling constant (*J* in Hz) and integration. Deuterated solvents were purchased from Sigma-Aldrich and dried over CaH with stirring overnight under an inert atmosphere, before being freeze-thaw degassed, vacuum transferred to a pressure ampule and stored in the glovebox. ^{13}C NMR spectra were recorded at 100 MHz using broadband proton decoupling, while ^{31}P NMR and ^{19}F NMR spectra were recorded at162 MHz and 376 MHz respectively with chemicals shifts reported in ppm. Known phosphine(III) compounds where compared to authentic samples by ^{1}H and ^{31}P NMR. Other than **2**, all other chlorophosphonium salts (CPS) were deprotected immediately due to their reactive nature and potential to decompose, as such CPSs were characterised by NMR only as they rapidly underwent hydrolysis when was attempted HMRS leading to the following statement from Institute of Organic Chemistry, Polish Academy of Sciences (PAS) ‘P-Cl bond: we are not able to measure these structures, because it is enough that there are traces of water in the ion source and P-Cl immediately changes to P=O’.

Commercially available reagents were purchased from the suppliers listed in the **Resources table** and used without further purification. (S)-Ph-BINEPINE was generously donated by the Kalek Group (CENT, Univeristy of Warsaw), other non-commercial reagents were prepared accord to the literate procedures; phosphine(V) oxides where prepared from their commercial phosphines(III) in THF with hydrogen peroxide solution[15] and compared to the known literature examples. A fresh bottle of oxalyl chloride was store in an oven dried ampule and on the Schlenk line under inert atmosphere. Hexachlorodisilane was stored in the glovebox where it was added to the CPS in methylene chloride or else it was collected in a syringe and the sealed by sticking the needle tip in a septum, then transferring from the glovebox and injecting into the reaction vessel under inert atmosphere.

Table S1: showing a compounds origin or reference for its literature synthesis.

Triphenylphosphine (V) Oxide	Tricyclophosphine(V) Oxide	3-Methyl-1-phenyl-2-phospholene 1-Oxide	*N,N*-Diisopropyl-*P,P*-diphenylphosphinamide	CyJohn Phos
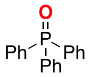	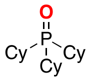	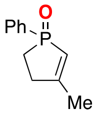	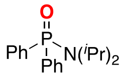	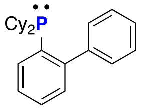
Sigma-Aldrich CAS: 791-28-6	TCI CAS: 13689-19-5	TCI CAS: 707-61-9	Sigma-Aldrich CAS: 131173-04-1	TCI CAS: 247940-06-3
DavePhos	(*S*)-Ph-BINEPINE	(*R*)-(+)-2-(Diphenylphosphino)-2′-methoxy-1,1′-binaphthyl	1,2-Bis(diphenylphosphino) ethane	Tris[2-(diphenylphosphino) ethyl] phosphine
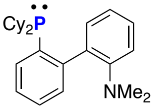	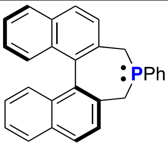	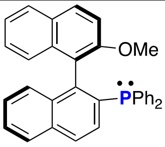	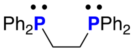	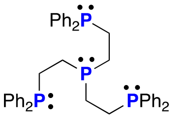
TCI CAS: 213697-53-1	Donated by the Kalek Group (Cent, University of Warsaw)	Sigma-Aldrich CAS: 145964-33-6	TCI: 1663-45-2	ACROS: 23582-03-8
di-tert-butyl(tosylmethyl)phosphine oxide	Sodium tetrakis(3,5-dichlorophenyl)borate	Dichloro(*p*-cymene)ruthenium(II) dimer	Umicore Grubbs Catalyst M310	Allylpalladium(II) chloride dimer
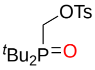	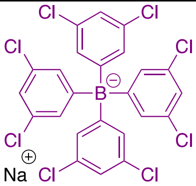	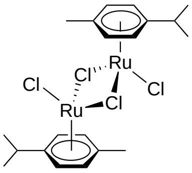	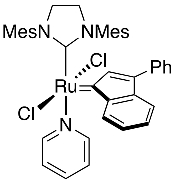	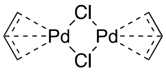
Prepared according to according to Hofmann et al.[9]	Prepared according to Serwatowski et al.[16]	Sigma-Aldrich CAS: 52462-29-0	Umicore CAS: 1031262-76-6	TCI CAS: 12012-95-2
Nickel(II) chloride ethylene glycol dimethyl ether complex	Bis(benzonitrile)palladium(II) Dichloride	Hexachlorodisilane	Oxalyl Chloride	Trimethylsilyl trifluoromethanesulfonate
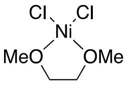	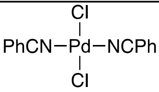	Si_2Cl_6	$(COCl)_2$	TMSOTf
Sigma-Alrich CAS: 29046-78-4	ABCR CAS: 14220-64-5	TCI CAS: 13465-77-5	Sigma-Aldrich CAS: 79-37-8	Sigma-Aldrich CAS: 27607-77-8

Preparation of chlorotriphenyl-λ^4-phosphanes:

Dichlorotriphenyl-λ^4-phosphane, 2a:[1]

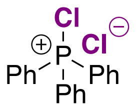

In a dry Schlenk flask triphenylphosphine(V) oxide (2.78 g, 10.0 mmol, 1.0 equiv.) was sealed under an atmosphere of argon with a septa before being dissolved in methylene chloride (20 mL) with stirring. A gentle flow of argon was established by piecing the septa with a needle attached to an oil bubbler. Oxalyl chloride (1.04 mL, 12.0 mmol, 1.2 equiv.) was added dropwise to the stirred solution from a syringe (**Caution**: this results in rapid evolution of carbon dioxide and carbon monoxide). The reaction was allowed to stir for 2 hours. The needle (connected to the bubbler) was removed and the tap of the Schlenk flask was closed before being attached to a solvent trap with rubber tubing. The trap was evacuated to $1x10^{-3}$ mbar and purged with argon, this was repeated a further two times, the trap was then placed under the active vacuum and submerged in dewar filled with nitrogen. The vessel was sealed under vacuum and transferred to the glovebox where the chlorophosphonium salt was dissolved in a small volume of methylene chloride and transferred to a vial, the solution was layered with large volume of hexane (3-5 times that of methylene chloride) and placed in the freezer at −30 °C. Upon complete crystallisation the supernatant was removed before the colourless crystals were dried under high vacuum, affording dichlorotriphenyl-λ5-phosphane (3.06 g, 9.18 mmol, 92% yield) which was stored in the glovebox freezer for future use: ^{1}H NMR (400 MHz, Methylene Chloride-d_2) δ = 8.01 – 7.94 (m, 3H), 7.85 – 7.77 (m, 12H); 101 MHz, Methylene Chloride-d_2) δ = 137.45 (d, *J*=3.1), 133.87 (d, *J*=13.0), 131.18 (d, *J*=15.1), 120.43 (d, *J*=96.7); ^{31}P NMR (162 MHz, Methylene Chloride-d_2) δ = 60.21.

Chlorotriphenyl-λ^4-phosphane trifluoromethanesulfate, 2b[2]

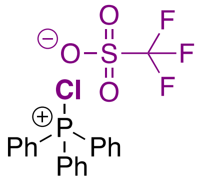

In the glovebox dichlorotriphenyl-λ^5-phosphane **5a** (333 mg, 1.0 mmol, 1.0 equiv.) was dissolved in methylene chloride (3 mL) with stirring. Trimethylsilyl trifluoromethanesulfonate (0.197 mL, 1.0 mmol, 1.0 equiv.) was added dropwise from a microsyringe and the reaction was allowed to stir. The reaction was monitored by ^{31}P NMR, confirming complete reaction before the solvents and TMSCl were evaporated *in vacuo*. The phosphonium salt was dissolved in a small volume of methylene chloride and the solution was layered with larger of hexane volume (3-5 times that of methylene chloride) then placed in the freezer at −30 °C. Upon complete crystallisation the supernatant was removed before the colourless crystals were dried under high vacuum, affording chlorotriphenyl-λ^4-phosphane trifluoromethanesulfate (391 mg, 0.88 mmol, 88% yield): ^{1}H NMR (400 MHz, Methylene Chloride-d_2) δ = 8.04 – 7.97 (m, 3H), 7.86 – 7.78 (m, 8H), 7.78 – 7.72 (m, 4H); ^{31}P NMR (162 MHz, Methylene Chloride-d2) δ = 65.9; ^{19}F NMR (376 MHz, Methylene Chloride-d2) δ = −78.9.

Chlorotriphenyl-λ^4-phosphane tetrakis(3,5-dichlorophenyl)borate, 2c

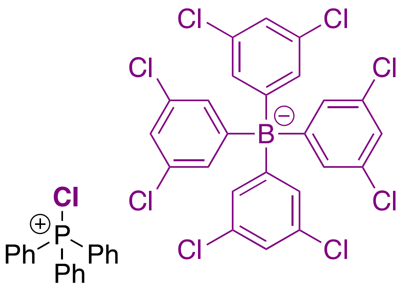

In the glovebox dichlorotriphenyl-λ^5-phosphane **5a** (67 mg, 0.2 mmol, 1.0 equiv.) was dissolved in methylene chloride (3 mL) with stirring. Sodium tetrakis(3,5-dichlorophenyl)borate (124 mg mL, 0.2 mmol, 1.0 equiv.) was added to the reaction was allowed to stir. The reaction was monitored by ^{31}P NMR before sodium chloride was filterd off by a pad of celite, washing with methylene chloride. The volume of methylene chloride was reduced before the solution was layered with larger of hexane volume then placed in the freezer at −30 °C. Upon complete crystallisation the supernatant was removed before the colourless crystals were dried under high vacuum, affording chlorotriphenyl-λ^4-phosphane tetrakis(3,5-dichlorophenyl)borate (140 mg, 0.16 mmol, 78% yield): ^{1}H NMR (400 MHz, Methylene Chloride-d2) δ = 7.97 – 7.91 (m, 3H), 7.75 – 7.69 (m, 6H), 7.68 – 7.61 (m, 6H), 7.02 (ddd $C_{24}H_{12}BCl_8O$, J=5.6, 2.8, 2.0, 9H), 6.94 (t, J=2.0, 3H); ^{13}C NMR (101 MHz, Methylene Chloride-d2) δ = 165.03 (q, *J* = 1.7 Hz), 137.94 (d, *J* = 3.3 Hz), 133.93 (d, *J* = 13.0 Hz), 133.45 (q, *J* = 1.5 Hz), 133.30 (q, *J* = 4.2 Hz), 131.27 (d, *J* = 14.6 Hz), 123.42, 118.81 (d, *J* = 93.8 Hz); ^{31}P NMR (162 MHz, Methylene Chloride-d2) δ = 65.8; ^{11}B NMR (128 MHz, Methylene Chloride-d2) δ = −7.0; HRMS (ESI) *m/z* [M-PClPh$_3$]$^-$ calcd for $C_{24}H_{12}BCl_8$: 590.8540. Found 590.8544; Elemental Analysis calculated for $C_{42}H_{27}BCl_9P$: C, 56.52; H, 3.05; Cl, 35.75 found: C, 56.49; H, 3.01; Cl, 35.78; IR (neat, cm^{-1}) ν = 3058 (w), 2967 (m); 2960 (m), 2902 (w), 1558 (m), 1542 (s), 1439 (m), 1368 (m), 1285 (w), 1112 (s), 1094 (m), 993 (w), 780 (m), 683 (m), 515 (m), 507 (m); Mp 188°C.

Disilane screening:

Reaction of dichlorotriphenyl-λ^4-phosphane, 2 with Hexachlorodisilane:

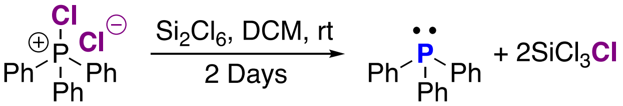

In the glovebox Ph_3PCl_2 (133 mg, 0.40 mmol, 1.0 equiv.) was dissolved in small vial with stirring in methylene chloride (1 mL). A few drops of TMS to the reaction mixture before hexachlorodisilane (76 µL, 0.44 mmol, 1.1 equiv.) was added from a Hamilton syringe. The reaction was sampled and diluted with *d*$_2$-methylene chloride before ^{31}P and ^{29}Si were acquired, demonstrating the clean complete consumption of CPS **2a** and clean formation of desired triphenylphosphine **3**; as well as the anticipated tetrachlorosilane, $SiCl_4$.

Figure S1: ^{31}P NMR: Ph_3PCl_2 reference (Top); reaction mixture immediately after addition of Si_2Cl_6 to Ph_3PCl_2 showing clean formation of Ph_3P (Bottom):

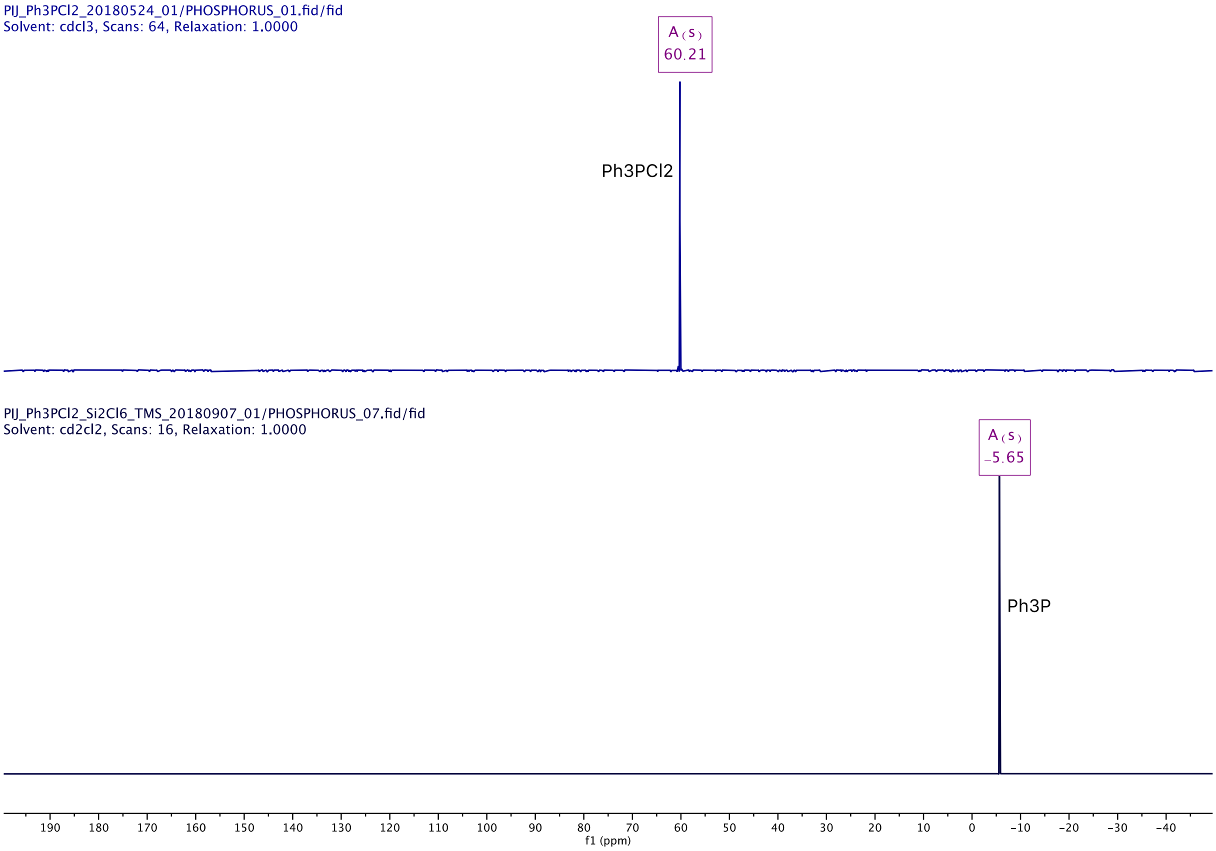

Figure, S2: ^{29}Si NMR with TMS internal standard: Si_2Cl_6 (Bottom) reactions mixture immediately after addition of Si_2Cl_6 to Ph_3PCl_2 showing formation of $SiCl_4$ (Top):

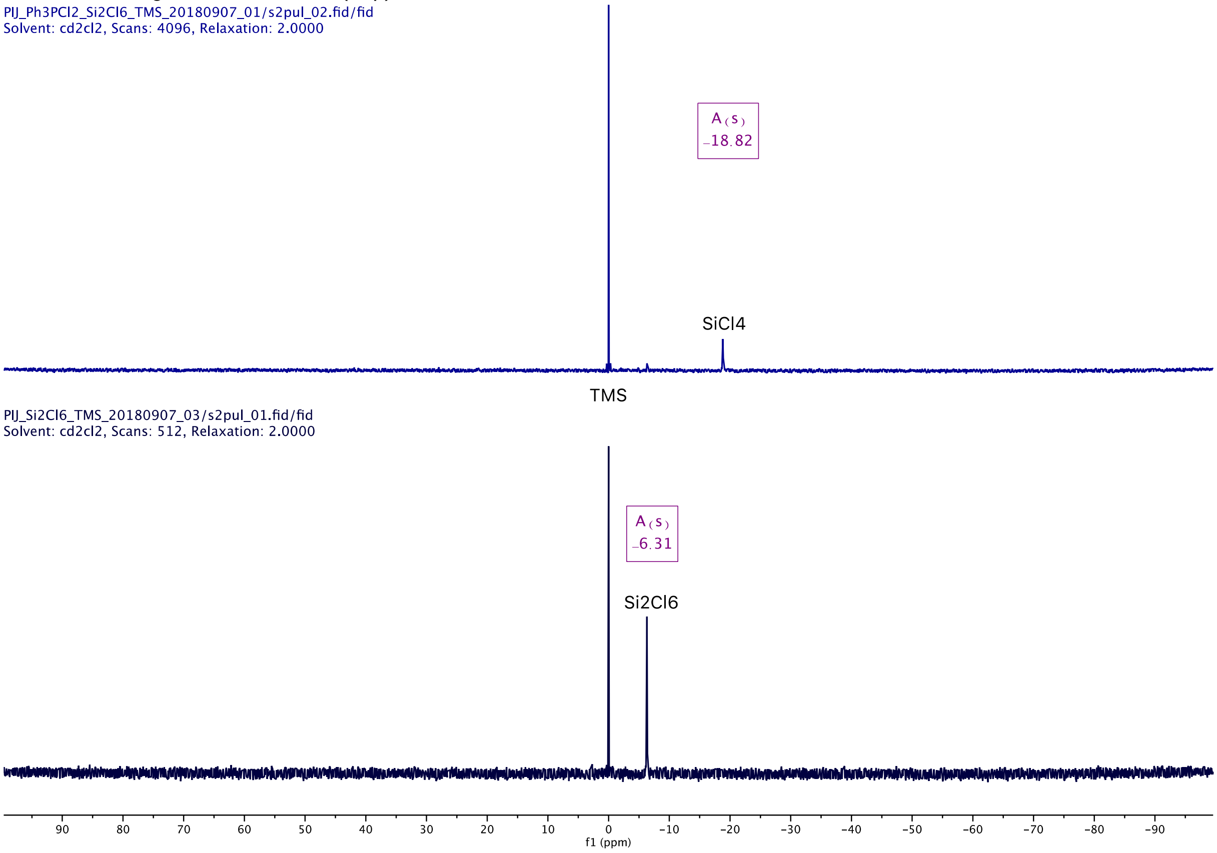

Reaction of dichlorotriphenyl-λ^4-phosphane, 2a with 1,1,2,2-tetrachloro-1,2-dimethyldisilane:

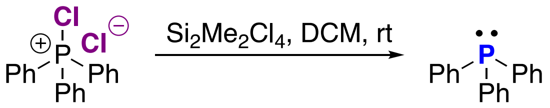

In the glovebox Ph_3PCl_2 (10 mg, 0.03 mmol, 1.0 equiv.) was dissolved in 0.7 mL *d*2-methylene chloride in an NMR tube, 1,1,2,2-tetrachloro-1,2-dimethyldisilane (5.93 µL, 0.033 mmol, 1.1 equiv.) was added from a Hamilton syringe. The tube was capped, shaken and sealed with parafilm before removing from the glovebox and examining ^{31}P NMR. No reaction was immediately observable so the reaction was periodically monitored over 144 hours. After 144 hours (6 days) ^{31}P NMR indicated the reaction was complete consumption CPS **2a** after this period.

Table S2: showing ^{31}P NMR intergrals and conversions, immediately after the addition of $Me_2Si_2Cl_4$ to **2a** then at 24 hour intervals up to 144 hours:

Hours	^{31}P NMR Integral Ph_3PCl_2	^{31}P NMR Integral Ph_3P	Conversion (%)
0	1.00	0.00	0.0
24	1.00	0.39	28.1
48	1.00	1.23	55.2
72	1.00	2.53	71.7
96	1.00	3.54	78.0
120	1.00	4.98	83.3
144	0.00	1.00	100.0

Figure S3: ^{31}P NMR immediately after addition of $Me_2Si_2Cl_4$ to **2a** then at 24 hour intervals up to 144 hours:

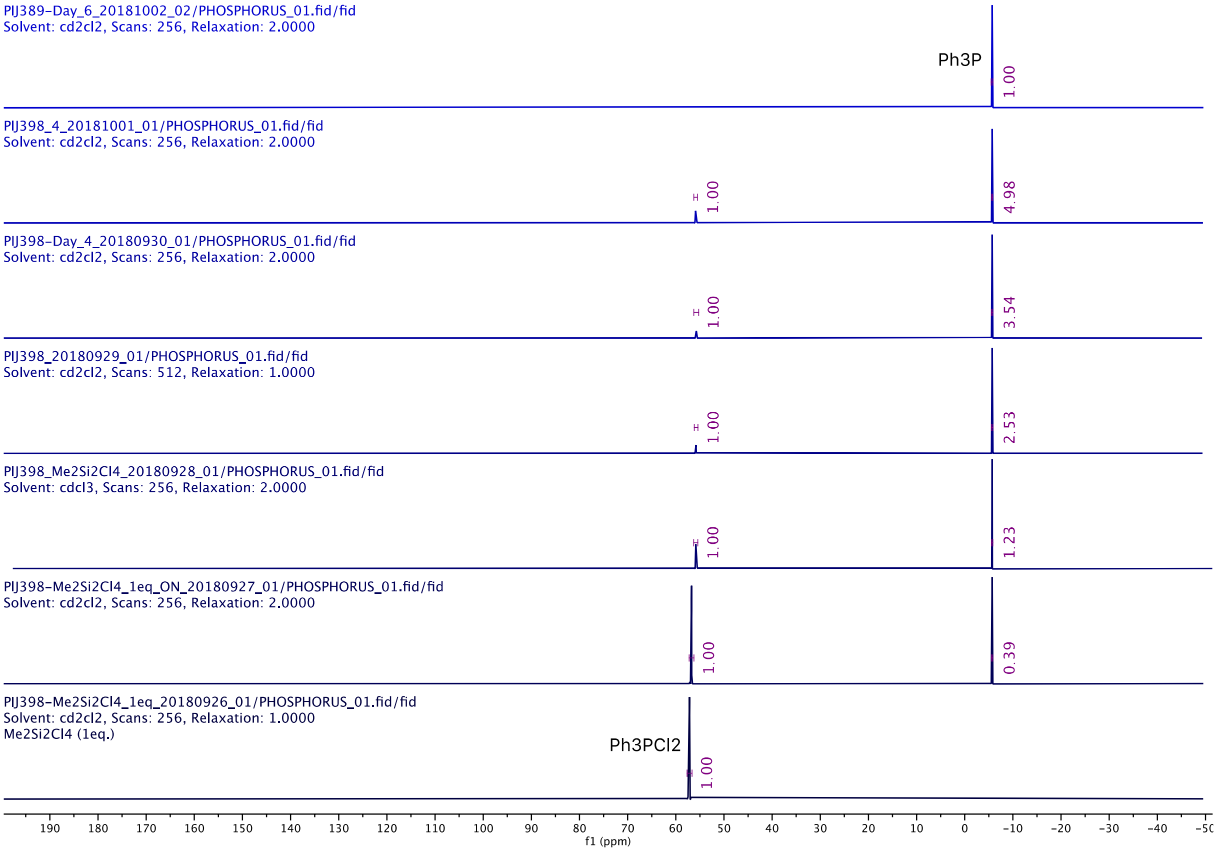

Reaction of dichlorotriphenyl-λ^4-phosphane, 2a with hexamethyldisilane:

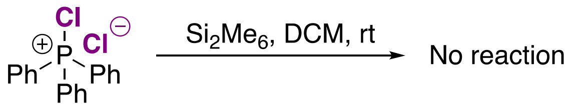

In the glovebox Ph_3PCl_2 (10 mg, 0.03 mmol, 1.0 equiv.) was dissolved in 0.7 mL *d*$_2$-methylene chloride in an NMR tube. Hexamethyldisilane (6.14 µL, 0.03 mmol, 1.0 equiv.) was added from a Hamilton syringe. The tube was capped, shaken and sealed with parafilm before removing from the glovebox and examining by ^1H and ^{31}P NMR, no reaction was observed (even after the addition of 4.0 further equivalents of hexamethyldisilane were add and monitored after 2 days).

Figure S4: ^1H NMR dichlorotriphenyl-λ^4-phosphane with hexamethyldisilane:

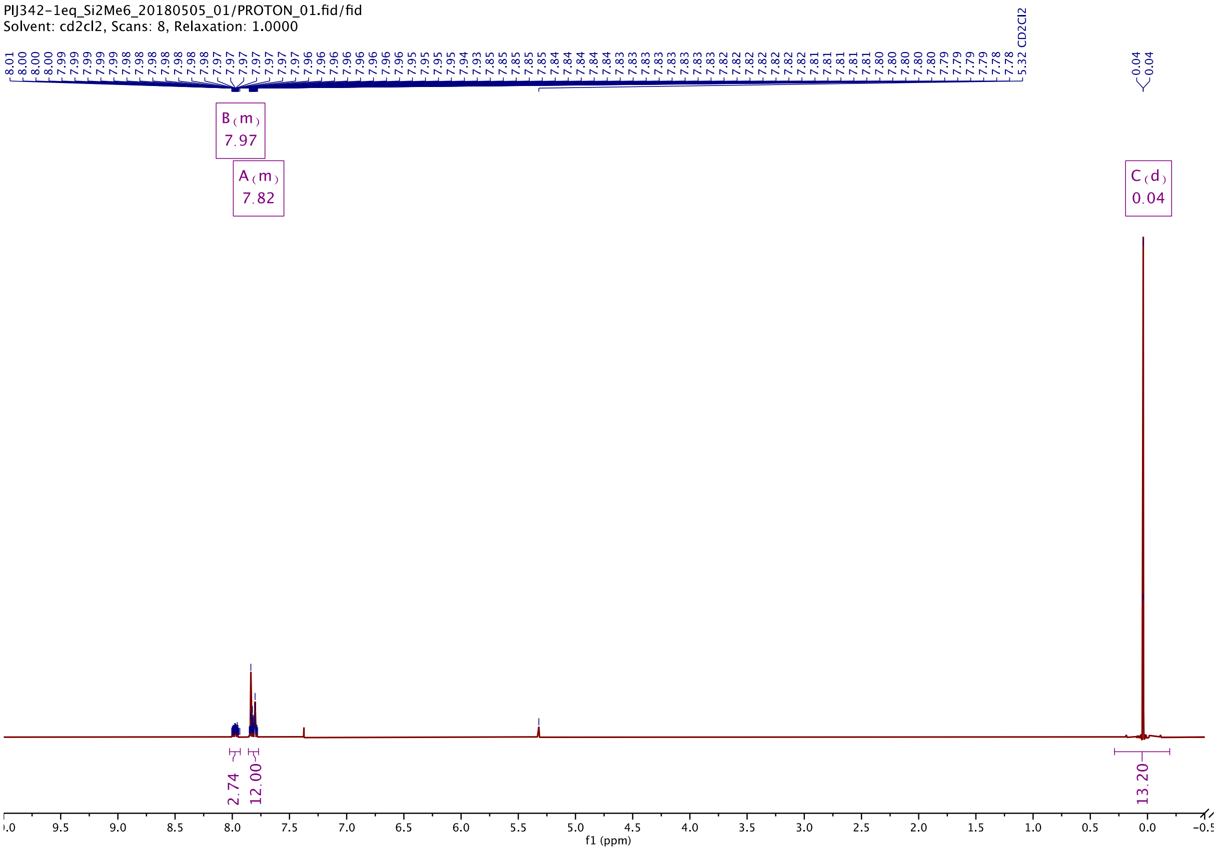

Figure S5: ^{31}P NMR dichlorotriphenyl-λ^4-phosphane with hexamethyldisilane showing only Ph_3PCl_2 start material:

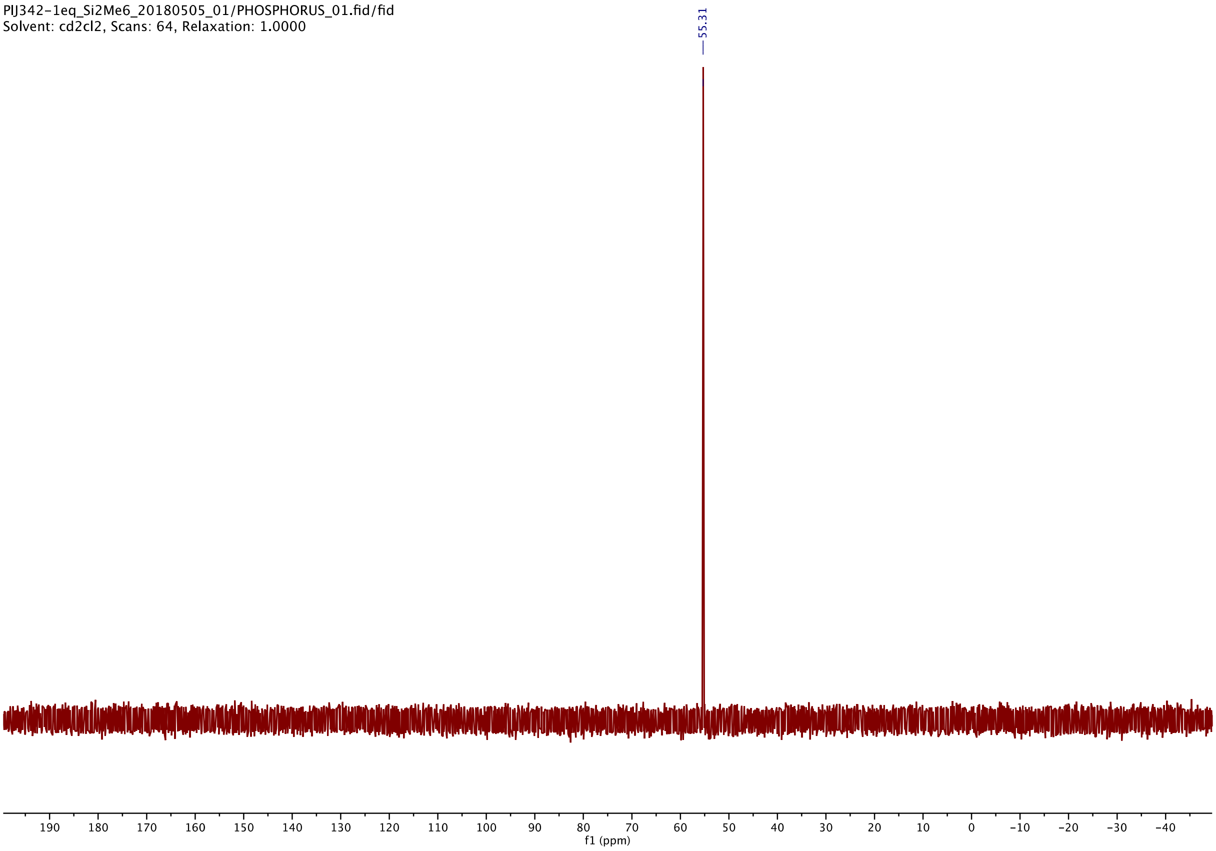

Reaction of dichlorotriphenyl-λ^4-phosphane, 2 with hexaphenyldisilane:

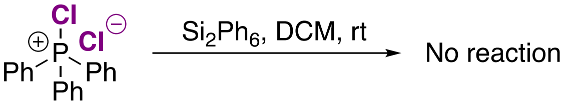

In the glovebox Ph_3PCl_2 (10 mg, 0.03 mmol, 1.0 equiv.) was dissolved in 0.7 mL d_2-methylene chloride in an NMR tube, hexaphenyldisilane (16 mg, 0.03 mmol, 1.0 equiv.) was added. The tube was capped, shaken and sealed with parafilm before removing from the glovebox and examining ^{31}P NMR, no reaction was observed.

Figure S6: ^{31}P NMR dichlorotriphenyl-λ^4-phosphane with hexaphenyldisilane showing only Ph_3PCl_2 start material:

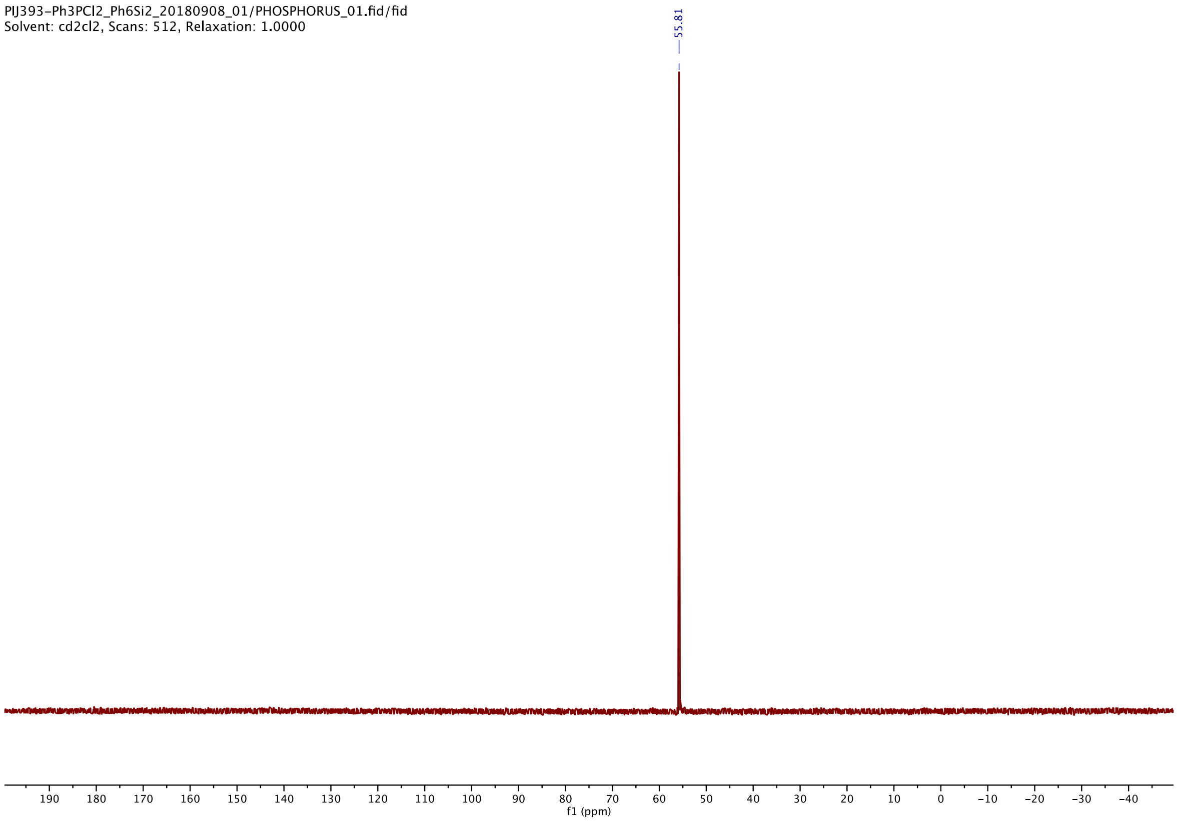

Reaction of Hexachlorodisilane with chlorotriphenyl-λ^4-phosphane trifluoromethanesulfate, 2b:

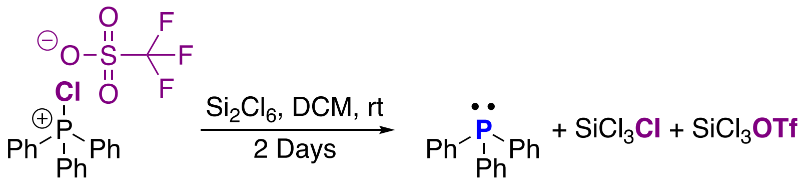

In the glovebox $Ph_3PClOTf$ (10 mg, 0.03 mmol, 1.0 equiv.) was dissolved in 0.7 mL d_2-methylene chloride in an NMR tube, hexachlorodisilane (6 µL, 0.033 mmol, 1.1 equiv.) was added. The tube was capped, shaken and sealed with parafilm before removing from the glovebox and examining by ^{31}P, ^{1}H, ^{19}F and ^{29}Si NMR, after 10 mins, 1 and 2 days. Reaction is complete after two days. However, a small unidentified impurity (less than 1%) is also represent after this period.

Table S3: showing ^{31}P NMR integrals and calculated conversions, immediately after the addtion of Si_2Cl_6 to $Ph_3PClOTf$, then at 1 and 2 days:

Day	^{31}P NMR Integral $Ph_3PClOTf$	^{31}P NMR Integral Ph_3P	Conversion (%)
0	1.00	0.07	6.5
1	1.00	4.05	80.2
2	0.00	1.00	100.0

Figure S7: ^{31}P NMR immediately after addition of Si_2Cl_6 to $Ph_3PClOTf$ (Bottom), then at 1 and 2 days (Middle and Top respectively):

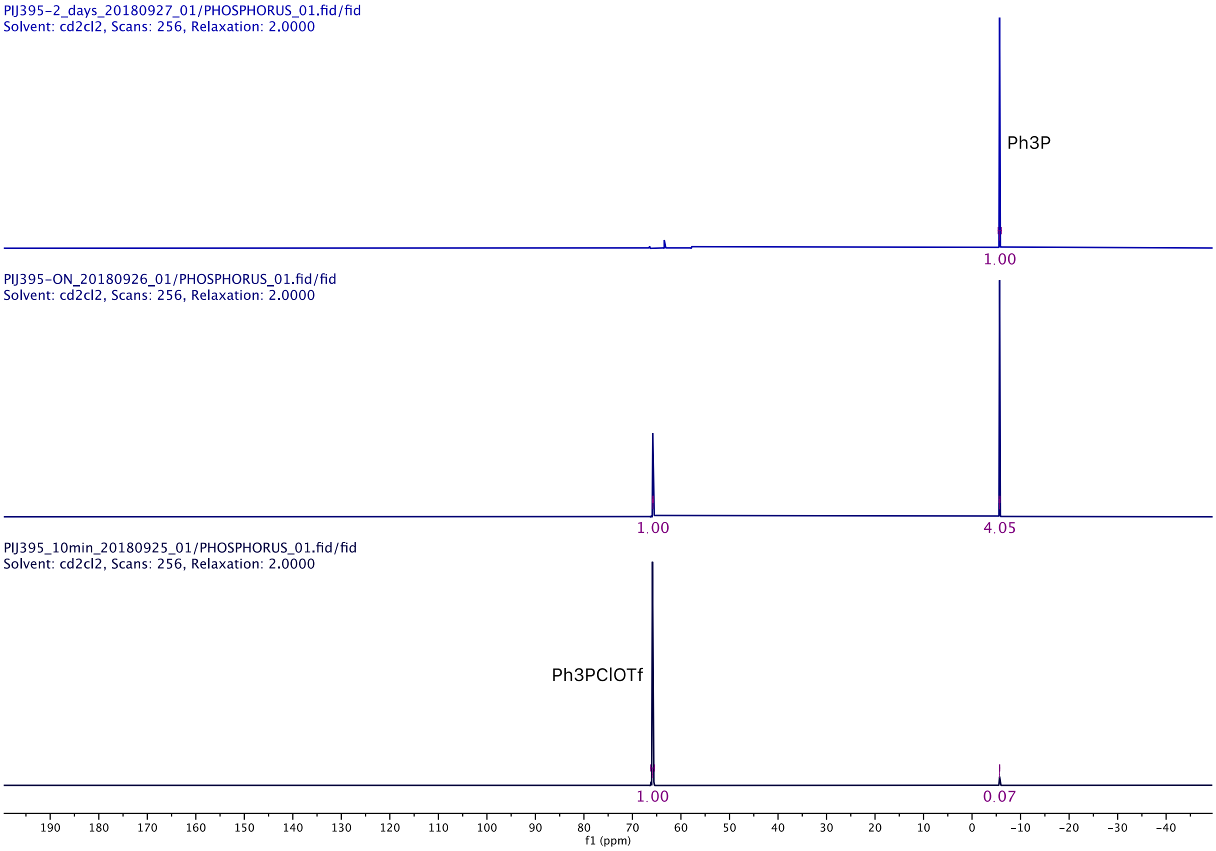

Figure S8: ^{1}H NMR immediately after addition of Si_2Cl_6 to $Ph_3PClOTf$ (Bottom), then at 1 and 2 days (Middle and Top respectively):

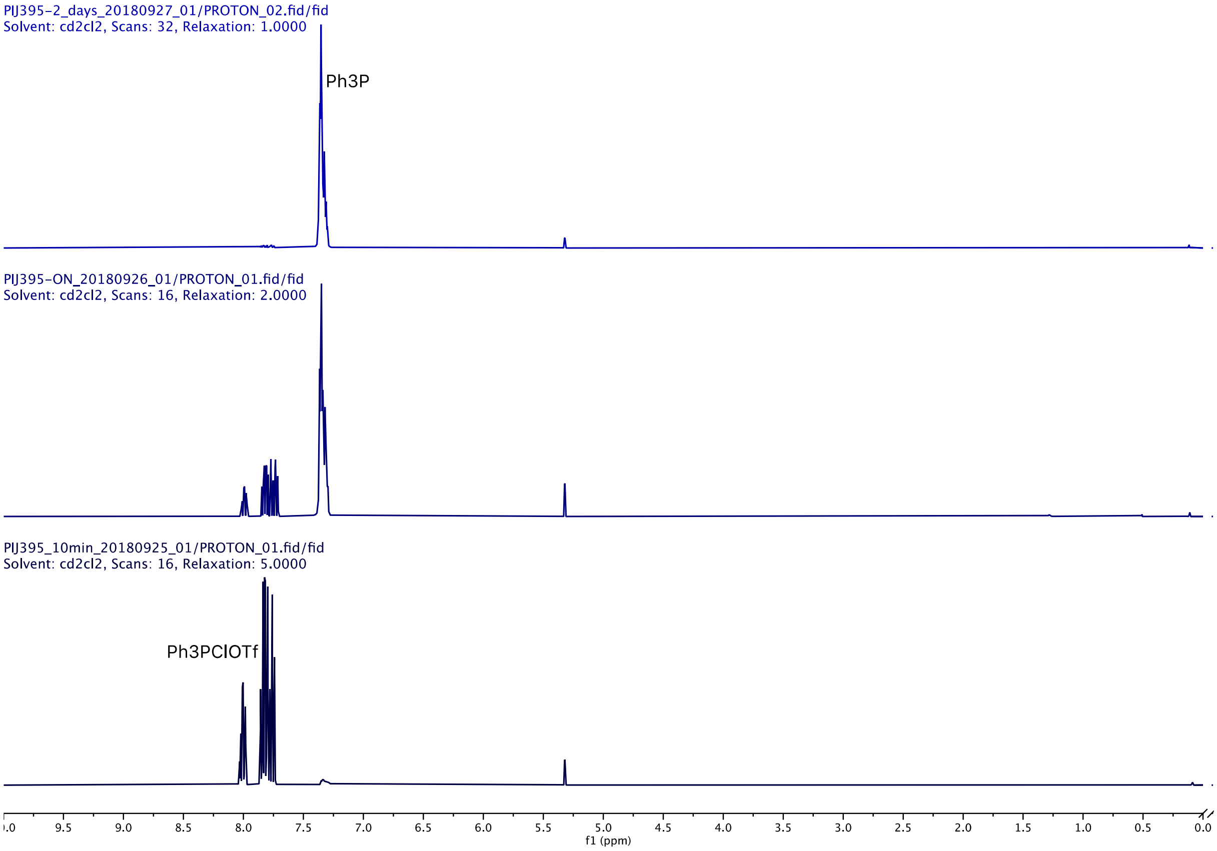

Figure S9: ^{19}F NMR immediately after addition of Si_2Cl_6 to $Ph_3PClOTf$ (Bottom), then at 1 and 2 days (Middle and Top respectively):

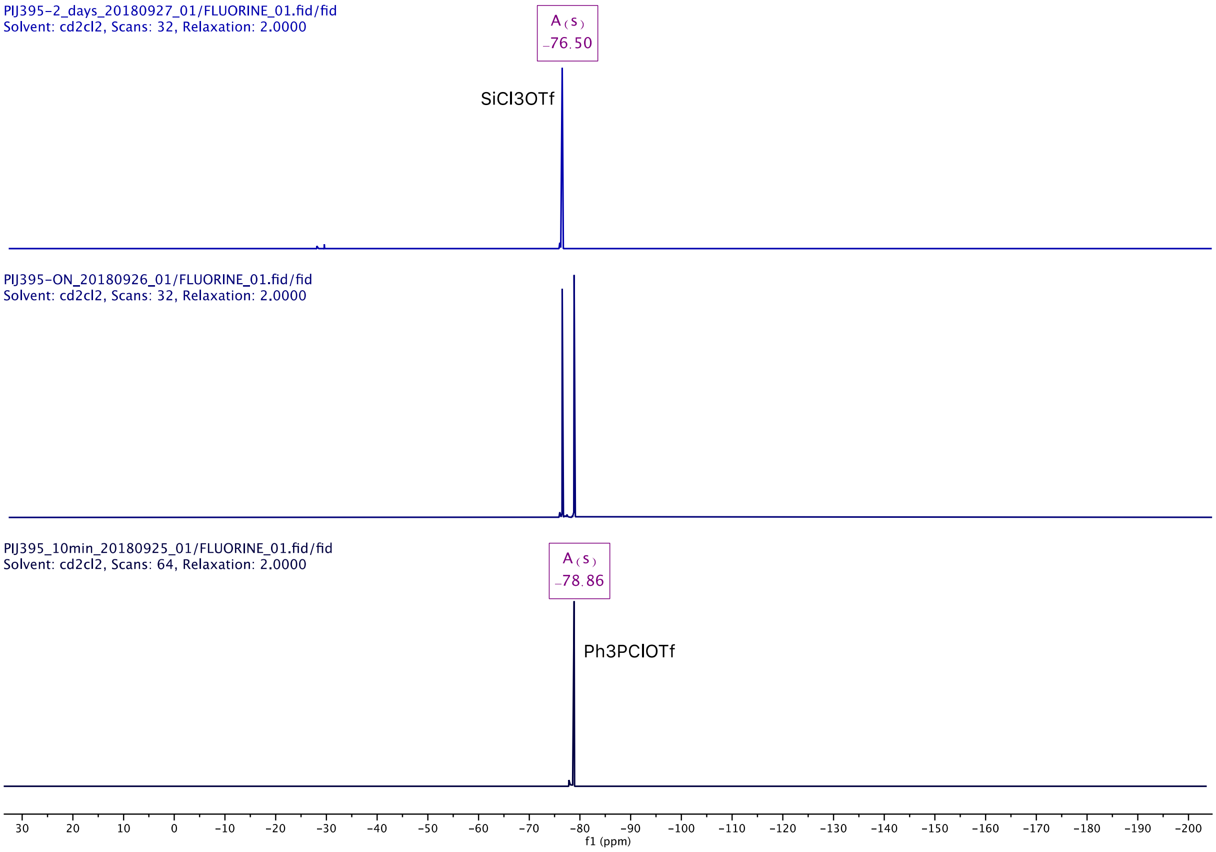

Figure S10: ^{29}Si NMR 2 days after addition of Si_2Cl_6 to $Ph_3PClOTf$ with TMS as internal standard:

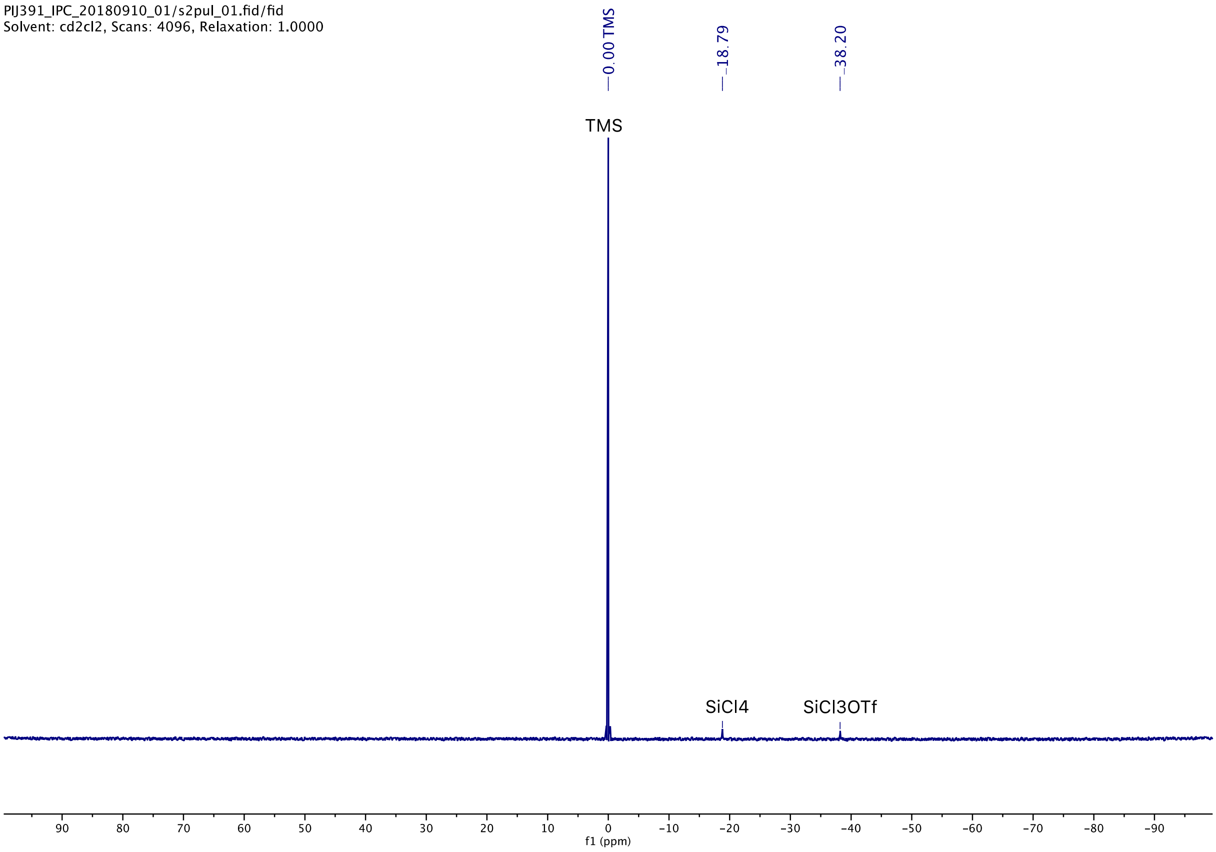

Reaction of Hexachlorodisilane with chlorotriphenyl-λ^4-phosphane tetrakis(3,5-dichlorophenyl)borate, 2c:

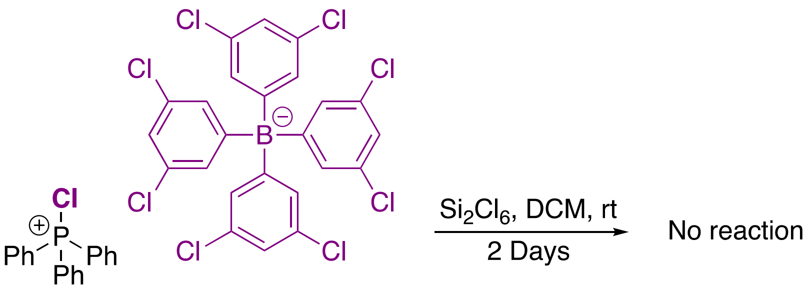

In the glovebox $Ph_3PClBAr^{Cl}$ (34.8 mg, 0.03 mmol, 1.0 equiv.) was dissolved in 0.7 mL *d*2-methylene chloride in an NMR tube, hexachlorodisilane (6 µL, 0.033 mmol, 1.1 equiv.) was added. The tube was capped, shaken and sealed with parafilm before removing from the glovebox and examining by ^{31}P and ^{1}H NMR, after 10 mins. No reaction was observed, additional hexachlorodisilane (21 µL, 0.12 mmol, 4.0 equiv.) was added before the reaction was analysis immediately by NMR and after two days, no triphenylphosphine formation was observed.

Figure S11: ^{31}P NMR immediately after addition of Si_2Cl_6 to $Ph_3PClBAr^{Cl}$, then at 1 and 2 days with excess Si_2Cl_6 added:

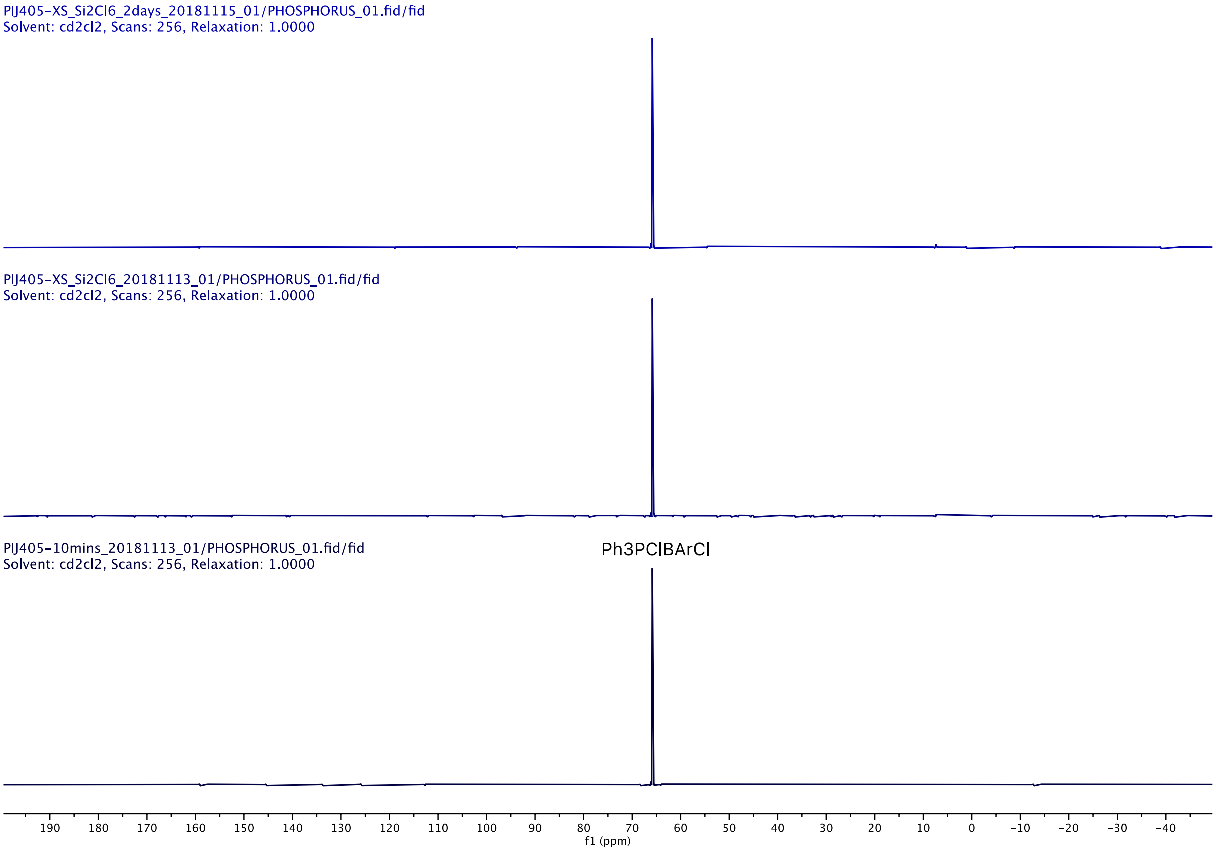

Figure S12: ^{1}H NMR immediately after addition of Si_2Cl_6 to $Ph_3PClBAr^{Cl}$, then at 1 and 2 days with excess Si_2Cl_6 added:

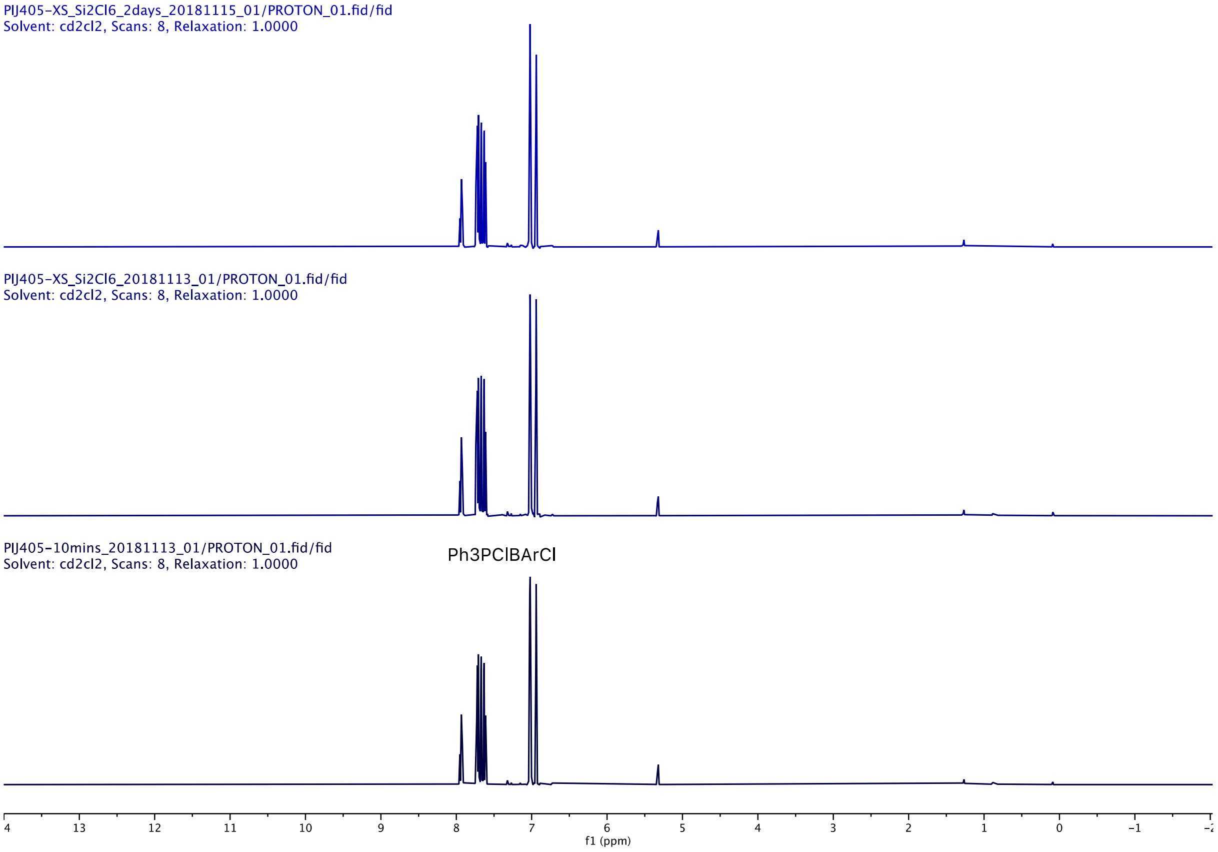

One-pot procedure for converting phosphine(V) oxides phosphine(III) *via* chlorophosphonium salts:

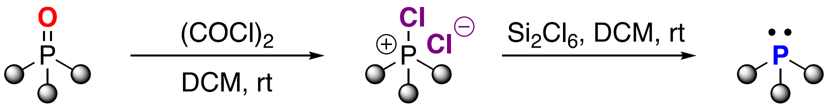

General Procedure 1 - In a dry Schlenk flask phosphine(V) oxide (1.0 equiv.) was sealed under an atmosphere of argon with a septa before being dissolved in dry degassed methylene chloride (3-10 mL per 1 mmol of start material) with stirring. A gentle flow of argon was established by piecing the septa with a needle attached to an oil bubbler. Oxalyl chloride (1.01-1.50 equiv. per phosphorus centre)* was added dropwise to the stirred solution from a glass microsyringe, resulting in evolution of carbon dioxide and carbon monoxide, the reaction was allowed to stir for 1 hour. As little as 1.01 equival. of oxalyl chloride may be used***** but for expedience we typically employed 1.5 equivalents in the following procedure:

1.05 - 5.0 equivalents of oxalyl chloride: The needle (connected to the bubbler) was removed and the tap of the Schlenk flask was closed before being attached to a solvent trap with rubber tubing. The trap/line was evacuated to $1x10^{-3}$ mbar and purged with argon, this was repeated a further two times, the trap was then placed under the active vacuum and submerged in dewar filled with nitrogen. All solvents were removed from the reaction vessel with stirring *in vacuo*. The Schlenk was then sealed under vacuum and transferred to glovebox (^{31}PNMR may be taken at to ensure complete activation). In the glovebox, the CPS was dissolved in methylene chloride (3-10 mL per 1 mmol of start material) then hexachlorodisilane (1.04 – 1.10 equiv. per phosphorus centre) was added dropwise with stirring, (^{31}PNMR may be taken at to ensure complete deprotection). The solvent was evaporated to dryness, the process was repeated twice more to ensure complete removal of residual Si_2Cl_6 or $SiCl_4$. The solid

was dissolved in methylene chloride and filtered through pipette packed with dry cotton and a small pad of celite into a tared flask. The solvent was removed *in vacuo* to afford spectroscopically pure phosphines(III) product crystalline solid.

1.01-1.05 equivalents of oxalyl chloride: When a small excess of oxalyl chloride***** was used hexachlorodisilane (1.04 – 1.10 equiv. per phosphorus centre) was added dropwise directly to reaction mixture (on the Schlenk line without prior evaporation) and allowed to stir for 5 minutes. The needle (connected to the bubbler) was removed and the tap of the Schlenk flask was closed before being attached to a solvent trap with rubber tubing. The trap/line was evacuated to $1x10^{-3}$ mbar and purged with argon, this was repeated a further two times, the trap was then placed under the active vacuum and submerged in dewar filled with nitrogen. All solvents were removed from the reaction vessel with stirring *in vacuo*. The Schlenk was then sealed under vacuum and transferred to glovebox (^{31}PNMR may be taken to ensure complete deprotection). In the glovebox, the phosphine(III) was dissolved in methylene chloride (3-10 mL per 1 mmol of start material) then the solvent was evaporated to dryness, the process was repeated (to ensure complete removal of residual Si_2Cl_6 or $SiCl_4$). The solid was dissolved in methylene chloride and filtered through pipette packed with dry cotton and a small pad of celite into a tared flask. The solvent was removed *in vacuo* to afford the desired phosphine(III) product as off-white crystalline solid.

*** Note:** Residual oxalyl chloride appeared to react with hexachlorodisilane vigorously and lead to discoloration of phosphine(III) and even undesired byproducts. Thus, when excess oxalyl chloride (> 1.05 equiv.) was employed all volatiles were first stripped for the intermediate CPS using the vacuum and trap. The solid CPS was then once again dissolved in dry degassed methyl chloride (3 mL) before hexachlorodisilane was added and worked up as per the above procedure.

Triphenylphosphine (*via* dichlorotriphenyl-λ^4-phosphane), 3:[3]

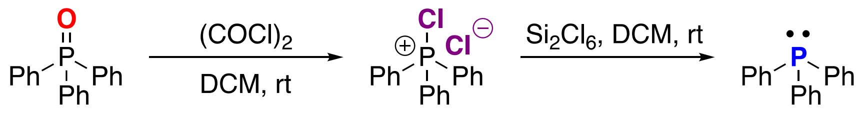

Dichlorotriphenyl-λ5-phosphane: ^{1}H NMR (400 MHz, Methylene Chloride-*d*$_2$) δ = 8.01 – 7.94 (m, 3H), 7.85 – 7.77 (m, 12H); 101 MHz, Methylene Chloride-*d*$_2$) δ = 137.45 (d, *J*=3.1), 133.87 (d, *J*=13.0), 131.18 (d, *J*=15.1), 120.43 (d, *J*=96.7); ^{31}P NMR (162 MHz, Methylene Chloride-*d*$_2$) δ = 60.21.

Triphenylphosphine(V) oxide (278 mg, 1.0 mmol, 1.0 equiv.) afforded triphenylphosphine (260 mg, 0.99 mmol, 99%) : ^{1}H NMR (400 MHz, Methylene Chloride-*d*$_2$) δ = 7.38 – 7.28 (m, 1H); ^{31}P NMR (162 MHz, Methylene Chloride-*d*$_2$) δ = -5.7.

Tricyclohexylphosphine (*via* dichlorotricyclohexyl-λ^4-phosphane), 7:

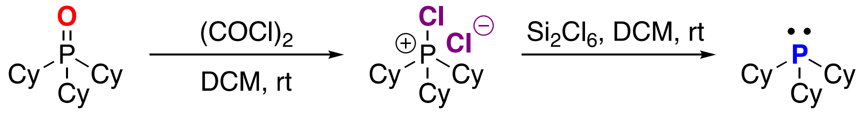

Dichlorotricyclohexyl-λ^4-phosphane: ^{1}H NMR (400 MHz, Chloroform-d) δ = 3.5 (s, 3H), 2.2 (s, 6H), 2.0 – 1.9 (m, 6H), 1.8 – 1.8 (m, 3H), 1.7 – 1.5 (m, 12H), 1.3 – 1.2 (m, 3H); δ = 53.43 (dt, *J*=54.5, 27.2), 35.84 (d, *J*=29.8), 26.58 (d, *J*=4.2), 25.89 (d, *J*=14.0), 25.05 (d, *J*=2.1); ^{31}P NMR (162 MHz, Methylene Chloride-*d*$_2$) δ = 107.2.

Tricyclophosphine oxide (88 mg, 0.25 mmol, 1.0 equiv.) afforded tricyclohexylphosphine (68 mg, 0.24 mmol, 97%):[3] ^{1}H NMR (400 MHz, Methylene Chloride-d_2) δ = 2.23 – 1.53 (m, 5H), 1.25 (qd, *J*=11.7, 10.9, 2.8, 4H); ^{31}P NMR (162 MHz, Methylene Chloride-d_2) δ = 10.4.

4-methyl-1-phenyl-2,3-dihydro-1H-phosphole[4] (*via* 1-chloro-4-methyl-1-phenyl-2,3-dihydro-1H-phosphol-1-ium chloride[1]), 8:

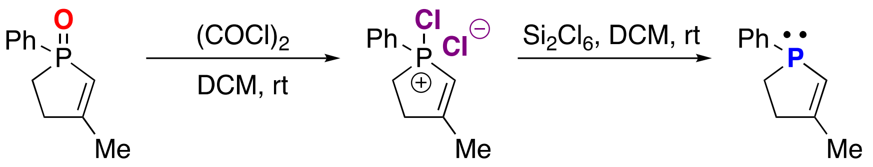

1-chloro-4-methyl-1-phenyl-2,3-dihydro-1H-phosphol-1-ium chloride: ^{1}H NMR (400 MHz, Methylene Chloride-d_2) δ = 8.29 – 8.21 (m, 2H), 7.79 – 7.73 (m, 1H), 7.68 – 7.60 (m, 2H), 6.73 (dp, *J*=32.4, 1.5, 1H), 3.70 – 3.62 (m, 3H), 3.45 – 3.35 (m, 3H), 2.30 (s, 3H); ^{31}P NMR (162 MHz, Methylene Chloride-d_2) δ = 99.94.

4-methyl-1-phenyl-2,3-dihydrophosphole 1-oxide (192 mg, 1 mmol, 1.0 equiv.) afforded 4-methyl-1-phenyl-2,3-dihydro-1H-phosphole (172 mg, 0.98 mmol, 98%): ^{1}H NMR (400 MHz, Methylene Chloride-d_2) δ = 7.44 – 7.39 (m, 1H), 7.33 – 7.26 (m, 2H), 5.82 – 5.68 (m, 1H), 2.75 – 2.64 (m, 1H), 2.54 – 2.41 (m, 1H), 2.32 – 2.17 (m, 1H), 1.98 (p, *J*=1.2, 3H), 1.91 – 1.81 (m, 1H); ^{31}P NMR (162 MHz, Methylene Chloride-d_2) δ = 3.47.

***N,N*-diisopropyl-1,1-diphenylphosphanamine[5] (*via* chloro(diisopropylamino)diphenyl-phosphonium chloride, 9:[5]**

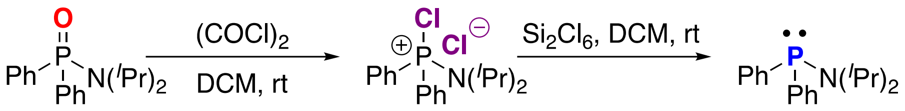

Chloro(diisopropylamino)diphenyl-phosphonium chloride: ^{1}H NMR (400 MHz, Chloroform-*d*) δ = 8.42 – 8.31 (m, 4H), 7.86 – 7.75 (m, 6H), 3.97 – 3.77 (m, 2H), 1.34 (d, *J*=6.9, 12H); ^{31}P NMR (162 MHz, Chloroform-d) δ = 68.3.

N,*N*-Diisopropyl-*P*,*P*-diphenylphosphinamide (301 mg, 1.0 mmol 1.0 equiv.) afforded *N,N*-diisopropyl-1,1-diphenylphosphanamine (255 mg, 0.89 mmol, 89%): ^{1}H NMR (400 MHz, Methylene Chloride-d_2) δ = 7.55 – 7.45 (m, 4H), 7.39 – 7.26 (m, 6H), 3.49 – 3.31 (m, 2H), 1.08 (d, *J*=6.7, 12H); ^{31}P NMR (162 MHz, Methylene Chloride-d2) δ = 37.1.

[1,1'-biphenyl]-2-yldicyclohexylphosphane, CyJohnPhos[3a] (*via* 1-Chloro-3-methyl-1-phenyl-2-phospholenium chloride), 10:

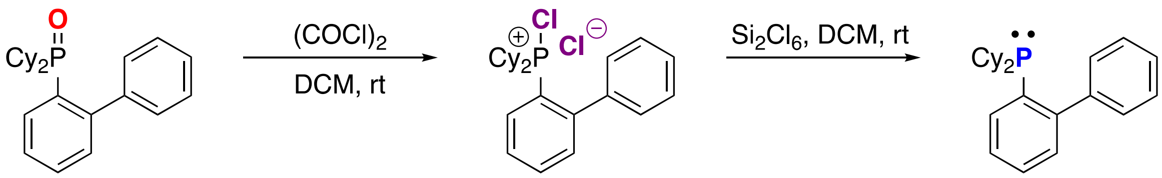

1-Chloro-3-methyl-1-phenyl-2-phospholenium chloride: ^{1}H NMR (400 MHz, Methylene Chloride-d_2) δ = 8.31 – 8.23 (m, 1H), 7.91 – 7.81 (m, 2H), 7.64 – 7.50 (m, 4H), 7.30 – 7.25 (m, 2H), 2.78 (ddt, *J*=14.2, 11.5, 2.7, 2H), 1.97 – 1.66 (m, 10H), 1.48 – 1.12 (m, 10H); ^{31}P NMR (162 MHz, Methylene Chloride-d_2) δ = 99.1.

[1,1'-biphenyl]-2-yldicyclohexylphosphine oxide (366 mg, 1.0 mmol, 1.0 equiv.) afforded [1,1'-biphenyl]-2-yldicyclohexylphosphane, CyJohnPhos (333 mg, 0.95 mmol, 95%): ^{1}H NMR (400 MHz, Methylene Chloride-d_2) δ = 7.64 – 7.58 (m, 1H), 7.40 – 7.23 (m, 7H), 1.83 (ttd, *J*=11.8, 3.1, 1.9, 2H), 1.76 – 1.47 (m, 10H), 1.26 – 0.93 (m, 10H); ^{31}P NMR (162 MHz, Methylene Chloride-d_2) δ = -13.40.

2-Dicyclohexylphosphino-2′-(*N,N*-dimethylamino)biphenyl, (DavePhos)[6] (*via* 2'-(dichlorodicyclohexyl- λ^5-phosphaneyl)-*N,N*-dimethyl-[1,1'-biphenyl]-2-amine, 11:

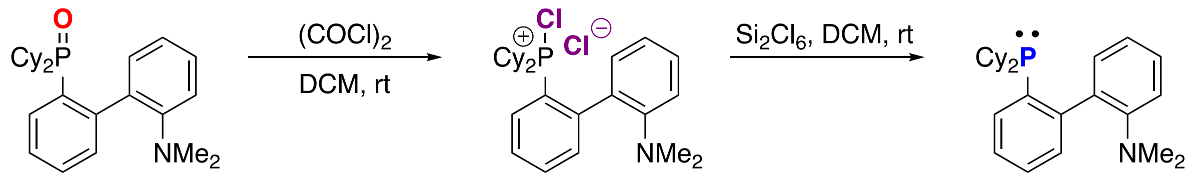

2'-(dichlorodicyclohexyl- λ^5-phosphaneyl)-N,N-dimethyl-[1,1'-biphenyl]-2-amine: ^{1}H NMR (400 MHz, Chloroform-*d*) δ = 9.02 (dd, *J*=15.1, 7.9, 1H), 7.94 – 7.78 (m, 2H), 7.51 – 7.40 (m, 2H), 7.40 – 7.28 (m, 1H), 7.15 (t, *J*=7.5, 1H), 7.06 (dd, *J*=7.5, 1.6, 1H), 4.49 (br. s, 1H), 3.84 (br. s, 1H), 2.61 (s, 6H), 2.28 – 0.87 (m, 20H).; ^{31}P NMR (162 MHz, Chloroform-*d*) δ = 96.0.

Dicyclohexyl(2'-(dimethylamino)-[1,1'-biphenyl]-2-yl)phosphine oxide afforded 2-dicyclohexylphosphino-2′-(*N,N*-dimethylamino)biphenyl, DavePhos (365 mg, 0.93 mmol, 93%): ^{1}H NMR (400 MHz, Methylene Chloride-d_2) δ = 7.61 – 7.53 (m, 1H), 7.39 (tdd, *J*=7.4, 1.5, 0.9, 1H), 7.33 (dd, *J*=7.5, 1.6, 1H), 7.33 – 7.23 (m, 2H), 7.06 – 6.92 (m, 3H), 2.43 (s, 6H), 2.01 (tdt, *J*=12.0, 6.1, 3.2, 1H), 1.89 – 0.73 (m, 21H)**;** ^{31}P NMR (162 MHz, Methylene Chloride-d_2) δ = -9.86.

(*S*)-Ph-BINEPINE[7] (*via* 4-chloro-4-phenyl-4,5-dihydro-3H-dinaphtho[2,1-c:1',2'-e]phosphepin-4-ium), 12:

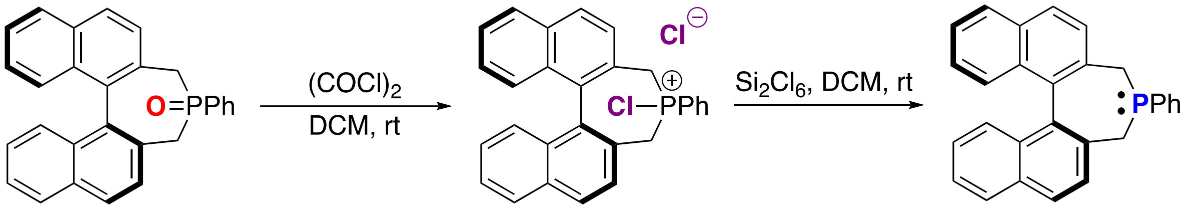

4-chloro-4-phenyl-4,5-dihydro-3H-dinaphtho[2,1-c:1',2'-e]phosphepin-4-ium: ^{1}H NMR (400 MHz, Methylene Chloride-d_2) δ = 8.76 – 8.66 (m, 2H), 8.03 (s, 4H), 8.00 – 7.96 (m, 2H), 7.81 – 7.74 (m, 1H), 7.73 – 7.65 (m, 2H), 7.59 – 7.51 (m, 2H), 7.33 – 7.25 (m, 2H), 7.15 (d, *J*=8.6, 2H), 6.07 (t, *J*=14.9, 2H), 3.74 (dd, *J*=15.5, 12.0, 2H); ^{31}P NMR (162 MHz, Methylene Chloride-d_2) δ = 86.7.

4-phenyl-3,5-dihydrodinaphtho[2,1-*c*:1',2'-*e*]phosphepine 4-oxide (101 mg, 0.25 mmol 1.0 equiv.) afforded 4-phenyl-4,5-dihydro-3H-dinaphtho[2,1-c:1',2'-e]phosphepine (93 mg, 0.24 mmol, 96%): ^{1}H NMR (400 MHz, Methylene Chloride-d_2) δ = 8.01 – 7.95 (m, 2H), 7.89 (dt, *J*=8.3, 1.1, 1H), 7.71 (dd, *J*=9.4, 8.4, 2H), 7.49 – 7.39 (m, 2H), 7.36 – 7.16 (m, 9H), 6.94 (d, *J*=8.3, 1H), 3.07 (dd, *J*=16.8, 11.5, 1H), 2.89 – 2.79 (m, 3H); ^{31}P NMR (162 MHz, Methylene Chloride-d_2) δ = 7.4.

(*R*)-(+)-2-(Diphenylphosphino)-2′-methoxy-1,1′-binaphthyl[8] (*via* 4-chloro-4-phenyl-4,5-dihydro-3H-dinaphtho[2,1-c:1',2'-e]phosphepin-4-ium chloride), 13:

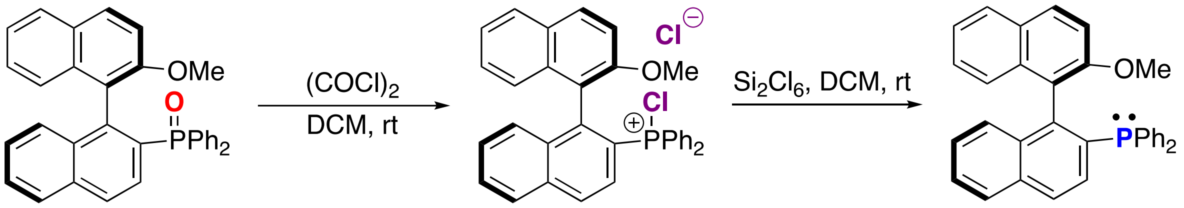

4-chloro-4-phenyl-4,5-dihydro-3H-dinaphtho[2,1-c:1',2'-e]phosphepin-4-ium chloride: ^{1}H NMR (400 MHz, Methylene Chloride-*d*2) δ = 8.44 (ddd, *J*=8.9, 3.7, 0.9, 1H), 8.19 (d, *J*=8.3, 1H), 8.10 (dd, *J*=14.4, 8.9, 1H), 7.84 – 7.78 (m, 1H), 7.76 (d, *J*=9.1, 1H), 7.70 – 7.61 (m, 3H), 7.46 – 7.33 (m, 9H), 7.30 (ddd, *J*=8.1, 6.8, 1.2, 1H), 7.20 (ddd, *J*=8.3, 6.9, 1.4, 1H), 7.18 – 7.13 (m, 1H), 7.09 (d, *J*=9.2, 1H), 6.70 – 6.66 (m, 1H), 3.49 (s, 3H); ^{31}P NMR (162 MHz, Methylene Chloride-*d*2) δ = 66.4.

(2'-methoxy-[1,1'-binaphthalen]-2-yl)diphenylphosphine oxide (135 mg, 0.279 mg, 1.0 equiv.) afforded 2'-methoxy-[1,1'-binaphthalen]-2-yl)diphenylphosphane (129 mg, 0.275 mmol, 99%): ^{1}H NMR (400 MHz, Methylene Chloride-*d*2) δ = 8.05 (d, *J*=9.0, 1H), 7.97 – 7.87 (m, 3H), 7.51 (ddd, *J*=8.1, 6.6, 1.4, 1H), 7.42 (dd, *J*=8.5, 2.8, 1H), 7.38 – 7.18 (m, 12H), 7.17 – 7.10 (m, 2H), 7.01 (d, *J*=8.4, 1H), 3.38 (s, 3H); ^{31}P NMR (162 MHz, Methylene Chloride-*d*2) δ = −14.1.

3-((di-*tert*-butylphosphoryl)methyl)-1-mesityl-1*H*-imidazol-3-ium 4-methylbenzenesulfonate, 4.[9]

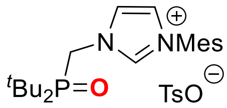

Into a round bottom flask equipped with a reflux condenser, 1-mesityl-1*H*-imidazole (1.02 g, 5.5 mmol, 1.1 equiv.) and di-tert-butyl(tosylmethyl)phosphine oxide (1.73 g, 5.0 mmol, 1.0 equiv.) were dissolved in 5 mL mesitylene with stirring under an inert atmosphere. The reaction mixture was heated at 170°C overnight before being cooled to room temperature. Hexane was added to ensure complete precipitation of salt and the suspension stirred for 1 hour. The white solid was filtered at the pump and washed with hexane before drying under high vacuum (2.54g, 4.77 mmol, 95%); ^{1}H NMR (400 MHz, Chloroform-*d*) δ = 9.85 (s, 1H), 8.44 (t, *J*=1.7, 1H), 7.79 – 7.71 (m, 2H), 7.14 – 7.05 (m, 3H), 7.07 (t, *J*=1.8, 2H), 7.00 (dd, *J*=1.3, 0.7, 2H), 5.36 (d, *J*=4.1, 2H), 2.35 (s, 3H), 2.32 (s, 3H), 2.03 (d, *J*=0.6, 6H), 1.32 (d, *J*=14.3, 18H); ^{13}C NMR (101 MHz, Chloroform-*d*) δ = 143.56, 141.52, 139.30, 138.84, 138.82, 134.31, 128.63, 126.12, 125.66, 122.05, 41.71 (d, *J*=46.2), 36.52 (d, *J*=58.4), 26.14, 21.45, 21.27, 17.60; ^{31}P NMR (162 MHz, Chloroform-*d*) δ = 57.75.

3-((di-*tert*-butyldichloro- λ^4-phosphaneyl)methyl)-1-mesityl-1*H*-imidazol-3-ium chloride, 15.

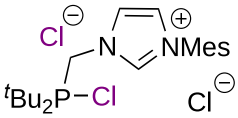

In a dry Schlenk flask, 3-((di-tert-butylphosphoryl)methyl)-1-mesityl-1H-imidazol-3-ium 4-methylbenzenesulfonate, (2.13 g, 4.0 mmol, 1 equiv.) was sealed under an atmosphere of argon with a septa before being dissolved in anhydrous degassed methylene chloride (20 mL) with stirring. A gentle flow of argon was established by piecing the septa with a needle attached to an oil bubbler. Oxalyl chloride (1.73 mL, 20.0 mmol, 5 equiv.) was added dropwise to the stirred solution, resulting in evolution of carbon dioxide and carbon monoxide. The reaction was allowed to stir for 2 hours. The needle

(connected to the bubbler) was removed and the tap of the Schlenk flask was closed before being attached to a solvent trap with rubber tubing. The trap was evacuated to $1x10^{-3}$ mbar and purged with argon, this was repeated a further two times, the trap was then placed under the active vacuum and submerged in dewar filled with nitrogen. All solvents were removed from the crude product, the Schlenk tube containing the solid chlorophosphonium salt was then sealed under vacuum and transferred to the glovebox. The crude was dissolved in the minimum volume of methylene chloride before an equal volume of hexane was added, then all solvents were removed once more. The residue was triturated with neat hexane and the resultant suspension stirred for 1 hour, filtered on a sinter and washed with fresh hexane. The powder was placed back into the Schlenk and suspended in hexane once more, then stirred for a further 30 minutes, filtered and washed with neat hexane. This process was repeated until all of *p*-toluenesulfonyl chloride was completely removed. The CPS was then immediately deprotected *vide infra*; ^{1}H NMR (400 MHz, Methylene Chloride-d_2) δ = 10.89 (s, 1H, NCHN), 9.42 (s, 1H, $N(CH)_2N$), 8.24 (s, 2H, *m*-Mes-CH), 7.36 (s, 1H, $N(CH)_2N$), 7.08 (s, 2H, PCH_2N), 2.37 (s, 3H, *p*-Mes-CH_3), 2.10 (s, 6H, *o*-Mes-CH_3), 1.76 (d, $^{3}J_{H,P}$ = 19.5 Hz, 18H, P^tBu_2-CH_3); ^{13}C NMR (101 MHz, Methylene Chloride-d_2) δ = 141.8 (s, Mes-C), 140.1 (s, NCHN), 134.2 (s, Mes-C), 130.5 (s, Mes-C), 129.8 (s, Mes-CH), 126.3 (s, $N(CH)_2N$), 123.0 (s, $N(CH)_2N$), 42.8 (d, $^{1}J_{C,P}$ = 24.4 Hz, PCH_2N), 42.2 (d, $^{1}J_{C,P}$ = 19.1 Hz, P^tBu_2-C), 26.9 (d, $^{2}J_{C,P}$ = 1.5 Hz, P^tBu_2-CH_3), 20.9 (*p*-Mes-CH_3), 17.6 (*o*-Mes-CH_3); ^{31}P NMR (162 MHz, , Methylene Chloride-d_2) δ = 108.7.

3-((di-*tert*-butylphosphaneyl)methyl)-1-mesityl-1*H*-imidazol-3-ium chloride, 5.[9]

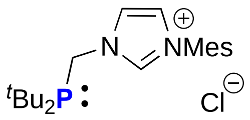

In the glovebox 3-((di-tert-butyldichloro-λ-4-phosphaneyl)methyl)-1-mesityl-1*H*-imidazol-3-ium chloride from the previous step was dissolved in methylene chloride with stirring in a Schlenk tube, hexachlorodisilane (1.03 mL, 6 mmol, 1.5 equiv.) was added dropwise from syringe and the reaction allowed to stir for 5 minutes. All solvents were removed *in vacuo*, the residue was dissolved in the minimum methylene chloride before an equal volume of hexane was added and the solvent evaporated to give solid that was dried for a further 10-30 mins. The process was repeat twice more before the solid or foam was dissolved in minimum volume of methylene chloride and filtering through a pad of celite into a tared flask. The solvent was removed *in vacuo*, to a foam which dried for 30 mins under high vacuum before it was triturated with a small volume of ether to induce precipitation. The pale brown powder was then dried under high vaccum to afford the desired phosphine(III), 3-((di-*tert*-butylphosphaneyl)methyl)-1-mesityl-1*H*-imidazol-3-ium chloride, (1.43 g, 3.75 mmol, 94%) in accordance to reference compound; ^{1}H NMR (400 MHz, Methylene Chloride-d_2) δ = 11.19 (s, 1H), 7.92 (s, 1H), 7.15 (t, *J*=1.8, 1H), 7.05 (s, 2H), 5.08 (s, 2H), 2.35 (s, 3H), 2.07 (s, 6H), 1.26 (d, *J*=11.8, 18H); ^{31}P NMR (162 MHz, Methylene Chloride-d_2) δ = 31.9.

1-((di-*tert*-butylphosphoryl)methyl)-3-(2,6-diisopropylphenyl)-1*H*-imidazol-3-ium 4-methylbenzenesulfonate

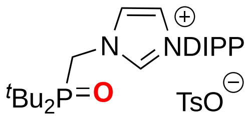

Into a round bottom flask equipped with a reflux condenser, 1-(2,6-diisopropylphenyl)-1*H*-imidazole (290 mg, 1.27 mmol, 1.1 equiv.) and di-tert-butyl(tosylmethyl)phosphine oxide (1.73 g, 5.0 mmol, 1.0 equiv.) were dissolved in 5 mL mesitylene with stirring under an inert atmosphere. The reaction mixture was heated at 170°C overnight before being cooled to room temperature. Hexane was added to ensure complete precipitation of salt and the suspension stirred for 1 hour. The white solid was filtered at the pump and washed with hexane before drying under high vacuum (650 mg, 1.13 mmol, 98%); ^{1}H NMR

(400 MHz, Chloroform-*d*) δ = 9.93 (d, *J* = 1.6 Hz, 1H, N*CH*N), 8.54 (t, *J* = 1.7 Hz, 1H, Ar), 7.80 – 7.70 (m, 2H, Ar), 7.54 (t, *J* = 7.8 Hz, 1H, Ar), 7.30 (d, *J* = 7.9 Hz, 2H, Ar), 7.13 – 7.05 (m, 3H, Ar), 5.40 (d, *J* = 4.2 Hz, 2H, CH_2) 2.31 (s, 3H, CH_3), 2.27 (hept, *J* = 6.8 Hz, 2H, CH), 1.33 (d, *J* = 14.3 Hz, 18H, $C(CH_3)_3$), 1.19 (d, *J* = 6.8 Hz, 6H, $CH(CH_3)_2$, 1.15 ppm (d, *J* = 6.8 Hz, 6H, $CH(CH_3)_2$); ^{13}C NMR (101 MHz, Chloroform-*d*) δ 145.4 (s, Ar), 139.2 (s, Ar), 139.1 (s, Ar), 139.0 (s, ArC), 132.1 (s, Ar), 130.2 (s, Ar), 128.6 (s, Ar), 126.1 (s, Ar), 125.7 (s, Ar), 124.8 (s, Ar), 123.1 (s, Ar), 41.8 (d, $^1J_{C,P}$ = 46.1 Hz, PCH_2), 36.5 (d, $^1J_{C,P}$ = 58.4 Hz, $P(C(CH_3)_3)$, 28.8 (s, $CH(CH_3)_2$), 26.1 (s, $(C(CH_3)_3)$, 24.5 (s, $CH(CH_3)_2$), 24.2 (s, $CH(CH_3)_2$), 21.4 (s, Ts-*p*-CH_3); ^{31}P NMR (162 MHz, Chloroform-*d*) δ = 58.05 ppm; HRMS (ESI) calculated for $[M-OTs]^+$: 403.2878 ($C_{24}H_{40}N_2OP$). Found 403.2851; IR (neat, cm^{-1}) ν = 3420 (br, m), 3081 (m), 2967 (vs), 2938 (s), 1543 (m), 1469 (m), 1365 (m), 1213 (s), 1190 (vs), 1122 (s), 1011 (s), 842 (w), 814 (m), 758 (w), 680 (s), 632 (m) and 567 (m); Mp 239 °C.

mono(1-((di-*tert*-butylchloro-λ^4-phosphaneyl)methyl)-3-(2,6-diisopropylphenyl)-1*H*-imidazol-3-ium) dichloride.

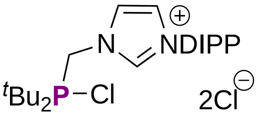

In a dry Schlenk flask, 1-((di-*tert*-butylphosphoryl)methyl)-3-(2,6-diisopropylphenyl)-1*H*-imidazol-3-ium 4-methylbenzenesulfonate, (1.01 g, 1.75 mmol, 1.0 equiv.) was sealed under an atmosphere of argon with a septa before being dissolved in anhydrous degassed methylene chloride (10 mL) with stirring. A gentle flow of argon was established by piecing the septa with a needle attached to an oil bubbler. Oxalyl chloride (0.76 mL, 8.75 mmol, 5.0 equiv.) was added dropwise to the stirred solution, resulting in evolution of carbon dioxide and carbon monoxide. The reaction was allowed to stir for 2 hours. The needle (connected to the bubbler) was removed and the tap of the Schlenk flask was closed before being attached to a solvent trap with rubber tubing. The trap was evacuated to $1x10^{-3}$ mbar and purged with argon, this was repeated a further two times, the trap was then placed under the active vacuum and submerged in dewar filled with nitrogen. All solvents were removed from the crude product, the Schlenk tube containing the solid chlorophosphonium salt was then sealed under vacuum and transferred to the glovebox. The crude was dissolved in the minimum volume of methylene chloride before an equal volume of hexane was added, then all solvents were removed once more. The residue was triturated with neat hexane and the resultant suspension stirred for 1 hour, filtered on a sinter and washed with fresh hexane. The powder was placed back into the Schlenk and suspended in hexane once more, then stirred for a further 30 minutes, filtered and washed with neat hexane. This process was repeated until all of *p*-toluenesulfonyl chloride was completely removed. The CPS was then immediately deprotected; ^{1}H NMR (400 MHz, Methylene Chloride-d_2) δ 11.07 (s, 1H, N*CH*N), 9.54 (s, 1H, ArH), 8.36 (s, 2H, CH_2), 7.60 (t, *J* = 7.9 Hz, 1H, ArH), 7.37 (d, *J* = 7.9 Hz, 2H, ArH), 7.33 (s, 1H, ArH), 2.31 (hept, *J* = 6.8 Hz, 2H. $CH(CH_3)_2$), 1.78 (d, *J* = 19.5 Hz, 18H, $C(CH_3)_3$)), 1.25 (d, *J* = 6.8 Hz, 6H, $CH(CH_3)_2$), 1.19 (d, *J* = 6.8 Hz, 6H, $CH(CH_3)_2$); ^{13}C NMR (101 MHz, Methylene Chloride-d_2) δ 145.6 (s, Ar), 140.8 (s, Ar), 132.6 (s, Ar), 130.4 (s, Ar), 126.7 (s, Ar), 125.2 (s, Ar), 124.6 (s, Ar), 43.3 (d, $^1J_{C,P}$ = 24.7 Hz, PCH_2), 42.8 (d, $^1J_{C,P}$ = 19.1 Hz, $P(C(CH_3)_3)$, 29.2 (s, $CH(CH_3)_2$), 27.4 (d, $^2J_{C,P}$ = 1.5 Hz, $P(C(CH_3)_3)$, 24.7 (s, $CH(CH_3)_2$), 24.3 (s, $(CH(CH_3)_2)$; ^{31}P NMR (162 MHz, Methylene Chloride-d_2) δ 108.54.

1-((di-*tert*-butylphosphaneyl)methyl)-3-(2,6-diisopropylphenyl)-1*H*-imidazol-3-ium chloride, 14:

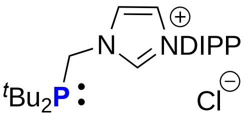

In the glovebox 1-((di-*tert*-butylphosphaneyl)methyl)-3-(2,6-diisopropylphenyl)-1*H*-imidazol-3-ium chloride from the previous step was dissolved in methylene chloride (5 mL) with stirring in a Schlenk tube, hexachlorodisilane (0.48 mL, 2.8 mmol, 1.6 equiv.) was added dropwise from a syringe and the reaction allowed to stir for 5 minutes. All solvents were removed *in vacuo*, the residue was dissolved in the minimum methylene chloride before an equal volume of hexane was added and the solvent evaporated to give solid that was dried for a further 10-30 mins. The process was repeat twice more before the solid or foam was dissolved in minimum volume of methylene chloride and filtering through a pad of celite into a tared flask. The solvent was removed *in vacuo*, to a foam which dried for 30 mins under high vacuum before it was triturated with a small volume of ether to induce precipitation. The pale brown powder was then dried under high vaccum to afford the desired phosphine(III), 3-((di-*tert*-butylphosphaneyl)methyl)-1-mesityl-1*H*-imidazol-3-ium chloride, (0.75 g, 2.8 mmol, 92%) in accordance to reference compound; ^{1}H NMR (400 MHz, Chloroform-*d*) δ 11.10 (t, *J* = 1.6 Hz, 1H, N*CH*N), 7.96 – 7.92 (m, 1H, ArH), 7.53 – 7.46 (m, 1H, ArH), 7.26 (d, *J* = 7.9 Hz, 2H, ArH), 7.13 (t, *J* = 1.8 Hz, 1H, ArH), 5.22 (s, 2H, CH_2), 2.23 (hept, *J* = 6.8 Hz, 2H, *CH*$(CH_3)_2$), 1.25 (d, *J* = 12.0 Hz, 18H, $PC(CH_3)_3$), 1.20 (d, *J* = 6.8 Hz, 6H, CH(CH_3)$_2$), 1.12 (d, *J* = 6.8 Hz, 6H, CH(CH_3)$_2$).; ^{13}C NMR (101 MHz, Chloroform-*d*) δ 145.2 (s, Ar), 139.1 (s, Ar), 131.6 (s, Ar), 130.2 (s, Ar), 124.4 (s, Ar), 123.9 (s, Ar), 123.1 (s, Ar) 123.0 (s, Ar) 45.0 (d, $^{1}J_{C,P}$ = 25.2 Hz, PCH_2), 32.3 (d, $^{1}J_{C,P}$ = 18.8 Hz, P(*C*$(CH_3)_3$)), 29.3 (d, *J* = 13.3 Hz), 28.6 (s, *CH*$(CH_3)_2$), 24.4 (s, CH(CH_3)$_2$), 24.0 (s, CH(CH_3)$_2$); ^{31}P NMR (162 MHz, Chloroform-*d*) δ 33.46; HRMS (ESI) calculated for $[M-Cl]^{+}$: 387.2929 ($C_{24}H_{40}N_2P$). Found 387.2927. IR (neat, cm^{-1}) ν = 2967 (vs), 2902 (s), 2871 (m), 1556 (w), 1540 (m), 1467 (m), 1446 (w), 1367 (w), 1274 (w), 1178 (m), 1065 (w), 812 (w), 723 (m); Mp: 242 °C.

A telescoped synthesis of metal complexes from their corresponding phosphine(V) oxides

General Procedure 2: The selected phosphine(V) oxide was converted to phosphine(III) using **general procedure 1**. In the glovebox, the phosphine(III) and the metal precursor where stirred in methylene chloride for the appropriate period of time, the reaction mixture was then filtered through a celite plug, washing with methylene chloride. The solvent was evaporated to dryness and the solid triturated and then stirred in a small volume of hexane before filtering and drying to afford the final desired metal complex, recrystallising where necessary.

Dichloro(η^6-*p*-cymene)(triphenylphosphine)ruthenium(II), 18:[10]

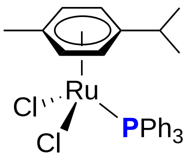

Triphenylphosphine(V) oxide (278 mg, 1.00 mmol, 1.0 equiv.) was converted to triphenylphosphine(III) as per the above method using oxalyl chloride (0.09 mL, 1.01 mmol, 1.01 equiv.) and then hexachlorodisilane (0.18 mL, 1.04 mmol, 1.04 equiv.). The 'intermediate' triphenylphosphine(**III**) was stirred with dichloro(*p*-cymene)ruthenium(II) dimer (306 mg, 0.5 mmol, 0.5 equiv.) in methylene chloride (6 mL) for 30 minutes. After workup the desired product dichloro(η6-*p*-cymene)(triphenylphosphine)ruthenium(II) (559 mg, 0.98 mmol, 98%) was afforded as a red lustrous powder: ^{1}H NMR (400 MHz, Methylene Chloride-*d*$_2$) δ = 7.83 – 7.75 (m, 6H), 7.46 – 7.34 (m, 9H), 5.19 (d, *J*=6.2, 2H), 4.99 (dd, *J*=6.3, 1.5, 2H), 2.76 (hept, *J*=6.9, 1H), 1.85 (s, 3H), 1.10 (d, *J*=7.0, 6H).; ^{31}P NMR (162 MHz, Methylene Chloride-*d*$_2$) δ = 24.56.

Umicore M2 (Grubbs Catalyst® M202) - [1,3-Bis(2,4,6-trimethylphenyl)-2-imidazolidinylidene] dichloro(3-phenyl-1*H*-inden-1-ylidene)(tricyclohexylphosphine)ruthenium(II), 19:[11]

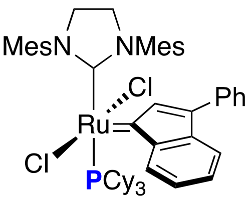

Tricyclohexylphosphine(V) oxide (74.1 mg, 0.25 mmol, 1.0 equiv.) was converted to tricyclohexylphosphine(III) as per the above method using oxalyl chloride (24 µL, 1.10 mmol, 1.10 equiv.) and then hexachlorodisilane (45 µL, 1.05 mmol, 1.05 equiv.). The 'intermediate' tricyclohexylphosphine was stirred with Grubbs Catalyst® M31 (168 mg, 0.23 mmol, 0.9 equiv.) in methylene chloride (2 mL) for 30 minutes. After workup the desired product Umicore M2 (204 mg, 0.22 mmol, 96%) was afforded as a red powder: ^{1}H NMR (400 MHz, Methylene Chloride-*d*$_2$) δ = 8.61 – 8.57 (m, 1H), 7.72 (dd, *J*=8.3, 1.4, 2H), 7.56 – 7.50 (m, 1H), 7.43 (ddt, *J*=8.2, 6.6, 1.2, 2H), 7.22 (tdd, *J*=15.0, 7.2, 5.9, 3H), 7.08 – 7.03 (m, 3H), 6.43 (s, 1H), 5.99 (s, 1H), 4.03 – 3.95 (m, 2H), 3.90 – 3.71 (m, 2H), 2.69 (s, 6H), 2.35 (s, 3H), 2.22 (s, 3H), 2.21 – 2.11 (m, 3H), 2.07 (s, 3H), 1.85 (s, 3H), 1.64 – 1.40 (m, 15H), 1.13 – 0.93 (m, 15H); ^{31}P NMR (162 MHz, Methylene Chloride-*d*$_2$) δ = 26.54.

CyJohnPhos(η3-allyl)PdCl, 20:[12]

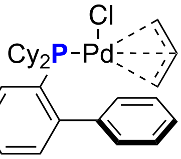

[1,1'-biphenyl]-2-yldicyclohexylphosphine oxide (183 mg, 0.5 mmol, 1.0 equiv.) was converted to [1,1'-biphenyl]-2-yldicyclohexylphosphane, CyJohnPhos as per the above method using oxalyl chloride (64.8 µL, 0.75 mmol, 1.5 equiv.) and then hexachlorodisilane (90 µL, 1.05 mmol, 0.53 equiv.). The 'intermediate' CyJohnPhos was stirred with (η^{3}-allyl)palladium(II) dichloride complexes in (3 mL) for 1 hour. After workup the solid was recrystallised from DCM/hexane at –30°C to afford the desired product CyJohnPhos(η3-allyl)PdCl (242 mg, 0.45 mmol, 91%) as a yellow crystalline solid: ^{1}H NMR (400 MHz, Methylene Chloride-*d*$_2$) δ = 7.70 – 7.24 (m, 9H), 4.95 – 4.81 (m, 1H), 4.31 (t, *J*=7.1, 1H), 3.14 (s, 1H), 3.09 (dd, *J*=13.6, 9.5, 1H), 2.27 (s, 2H), 1.96 (s, 2H), 1.86 – 1.03 (m, 18H); ^{31}P NMR (162 MHz, Methylene Chloride-*d*$_2$) δ = 28.3.

(dppe)NiCl$_2$, 21:[13]

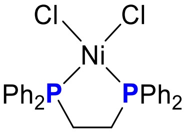

Ethane-1,2-diylbis(diphenylphosphine oxide) (215 mg, 0.50 mmol, 1.0 equiv.) was converted to 1,2-bis(diphenylphosphaneyl)ethane (dppe) as per **general method 1** using oxalyl chloride (0.13 mL, 1.5 mmol, 3.0 equiv.) and then hexachlorodisilane (0.18 mL, 1.05 mmol, 2.1 equiv.) in 5 mL dry methylene chloride. In the glovebox the filtered DPPE was then stirred with Nickel(II) chloride, dimethoxyethane adduct (110 mg, 0.5 mmol, 1.0 equiv.) in methylene chloride (10 mL) overnight. The solvent was evaporated to dryness. The solid was suspended in small volume hexane and stirred for 1 hour, before being filtered and dried under vacuum to afford (dppe)NiCl$_2$ (219 mg, 0.42, 83%) as a fine, orange powder: ^{1}H NMR (400 MHz, Methylene Chloride-*d*$_2$) δ = 8.05 – 7.95 (m, 8H), 7.62 (t, *J*=7.3, 4H), 7.55 (t,

J=7.4, 8H), 2.16 (d, *J*=18.2, 4H); ^{31}P NMR (162 MHz, Methylene Chloride-d_2) δ = 57.7; HRMS (ESI) *m/z* calcd for $[M-Cl]^+$: 491.0395 ($C_{20}H_{26}ClNiP_2$): .Found 491.0380.

[Pd(P4)Cl]Cl, 22:[14]

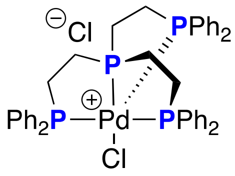

((oxo-λ^5-phosphanetriyl)tris(ethane-2,1-diyl))tris(diphenylphosphine oxide) (75 mg, 0.10 mmol, 1.0 equiv.) was converted to tris(2-(diphenylphosphaneyl)ethyl)phosphane using oxalyl chloride (52 µL, 0.60 mmol, 6.0 equiv.) and then hexachlorodisilane (0.71 µL, 0.41 mmol, 4.1 equiv.) in 3 mL dry methylene chloride. In the glovebox the filtered phosphine was then stirred with bis(benzonitrile)palladium(II) dichloride (40 mg, 0.1 mmol, 1.0 equiv.) in methylene chloride (4 mL) overnight. The solvent was evaporated to dryness before being recrystallized form methylene chloride and hexane at –30°C to afford the desired product (73 mg, 0.09 mmol, 86%); ^1H NMR (400 MHz, Methylene Chloride-d_2) δ = 7.54 – 7.40 (m, 10H), 7.40 – 7.31 (m, 5H), 7.17 (t, *J* = 7.7 Hz, 9H), 3.05 (dt, *J* = 17.8, 10.6 Hz, 5H), 2.62 (d, *J* = 27.8 Hz, 6H); ^{31}P NMR (162 MHz, Methylene Chloride-d_2) δ = 136.6, 31.2; HRMS (ES^+) calcd for $[M-Cl]^+$: 811.0960 ($C_{42}H_{42}ClPdP_4$). Found 811.0948.

Computation:

Quantum chemical calculations using the TURBOMOLE program were carried out to study the thermodynamics and kinetics of the reaction. Using the harmonic oscillator and rigid rotator approximation with a reference pressure of 1 bar, Gibbs free energies are given at the PBE0-D3/def2-TZVPP//PBE-D3/dhf-SV(P) level of theory. [17] Our calculations show that the formation of phosphines by direct liberation of CL2 is uphill in free energy by 94 kJ/mol. Formation of (unstabilized) SiCl2 by disproportionation of Si_2Cl_6 is also expected to be very unfavorable (DG = 107 kJ/mol). Formation of the free phosphine with Si2Cl6 releasing two $SiCl_4$ molecules, however is thermodynamically favorable (DG = -246 kJ/mol).

NMR Spectra:

Figure S13: ^{1}H NMR of dichlorotriphenyl-λ^4-phosphane, **2a**.[1]

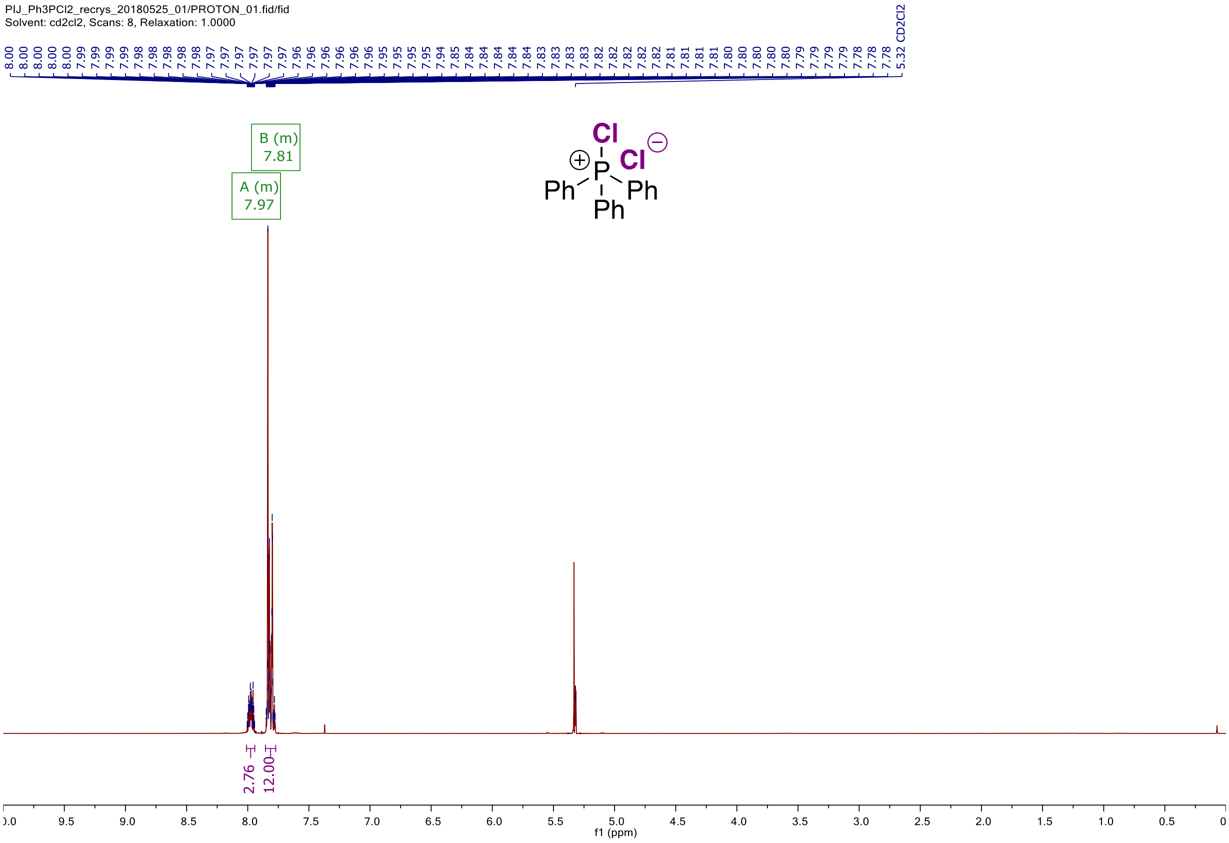

Figure S14: ^{31}P NMR of dichlorotriphenyl-λ^4-phosphane, **2a**.[1]

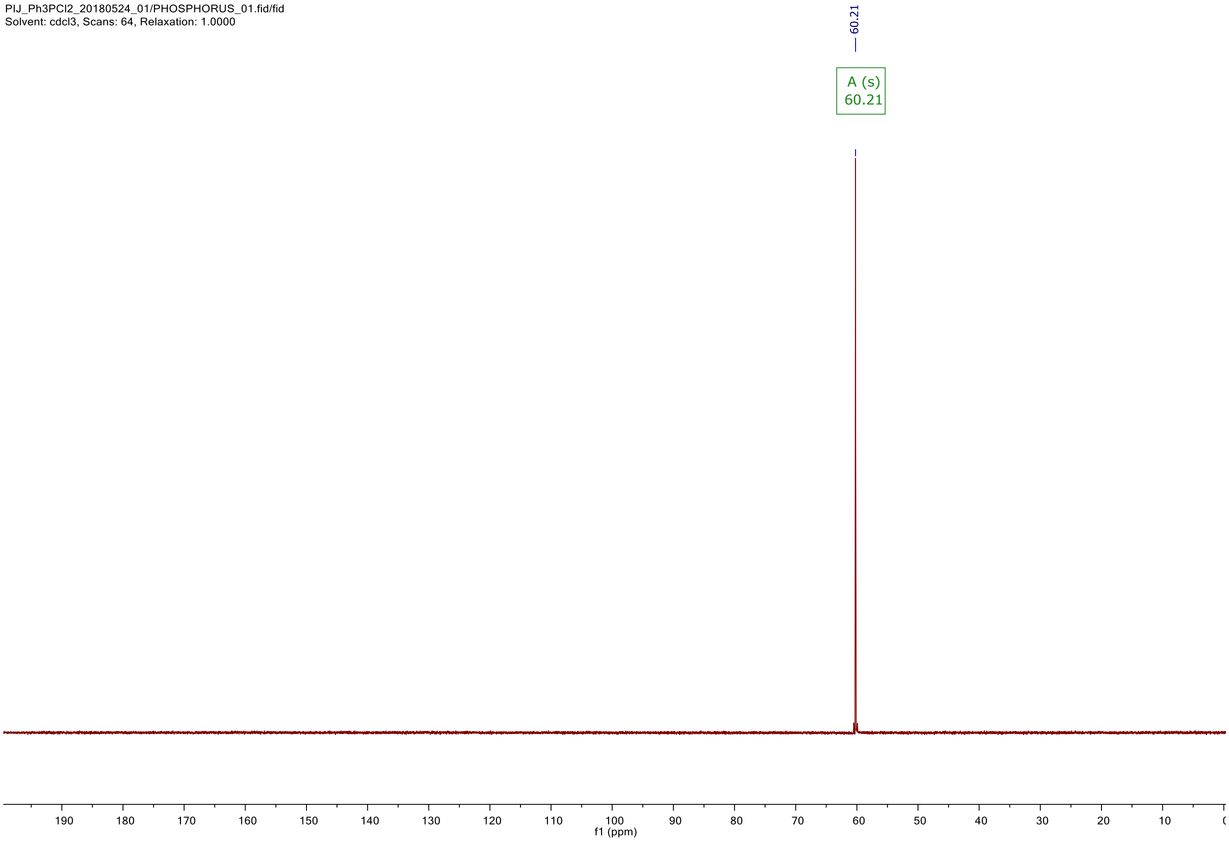

Figure S15: ^{1}H NMR of chlorotriphenyl-λ^4-phosphane trifluoromethanesulfonate, **2b**.[2]

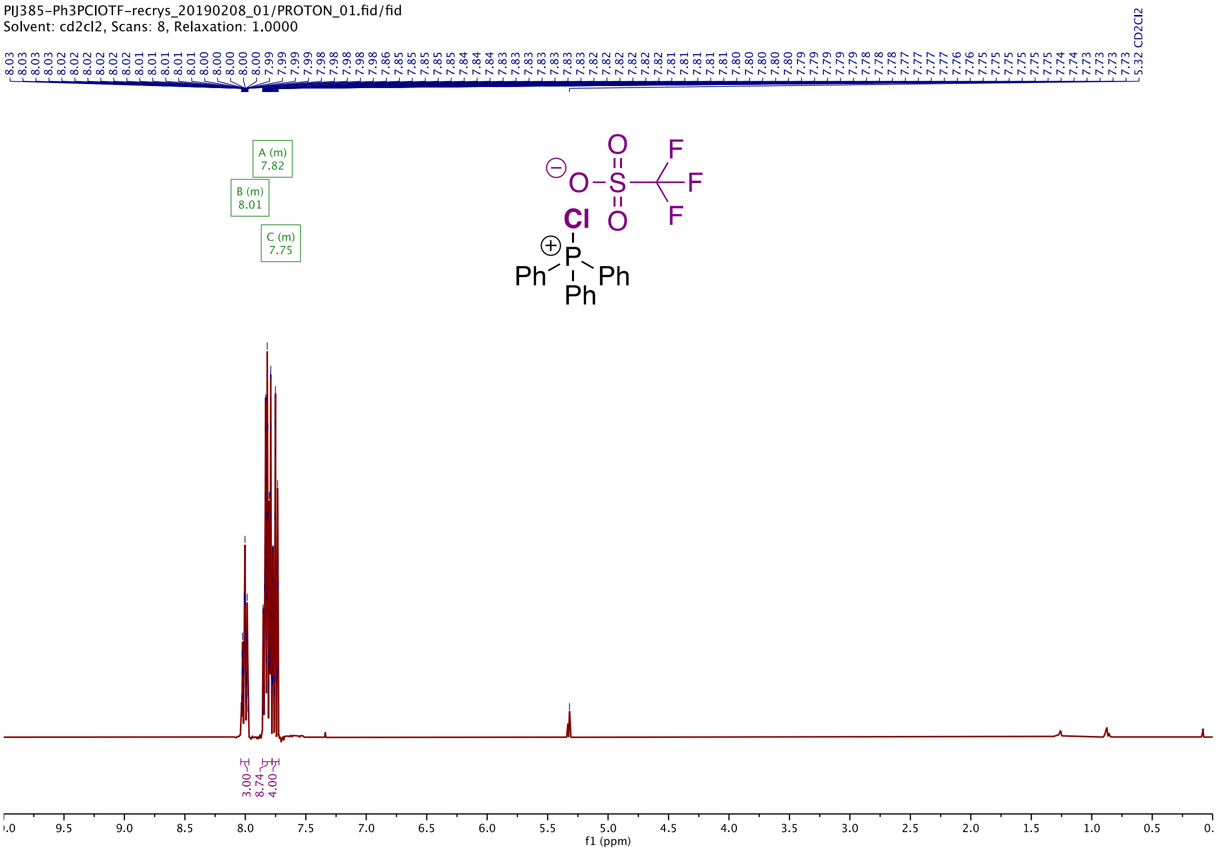

Figure S16: ^{31}P NMR Chlorotriphenyl-λ^4-phosphane trifluoromethanesulfonate, **2b**.[2]

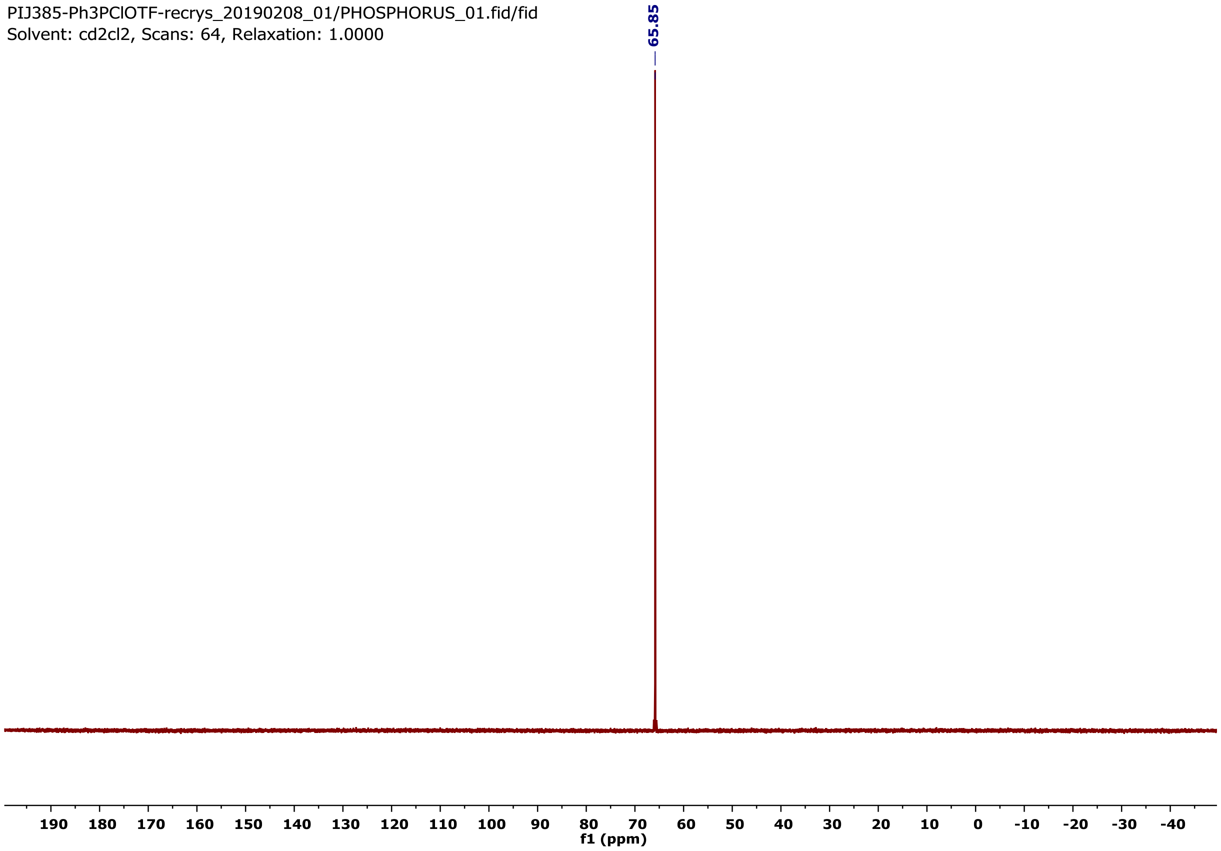

Figure S17: ^{19}F NMR of chlorotriphenyl-λ^4-phosphane trifluoromethanesulfate, **2b**.[2]

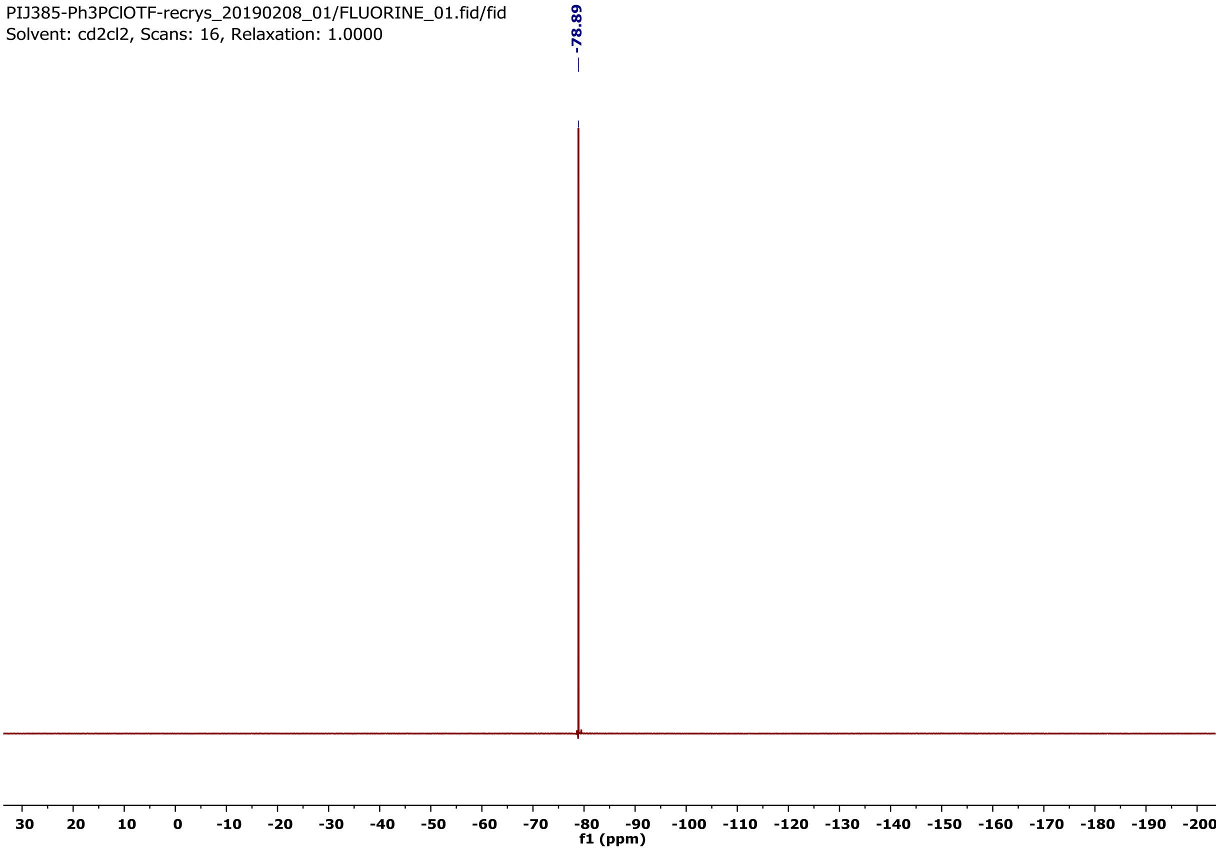

Figure S18: ^{19}H NMR of chlorotriphenyl-λ^4-phosphane tetrakis(3,5-dichlorophenyl)borate, **2c**.

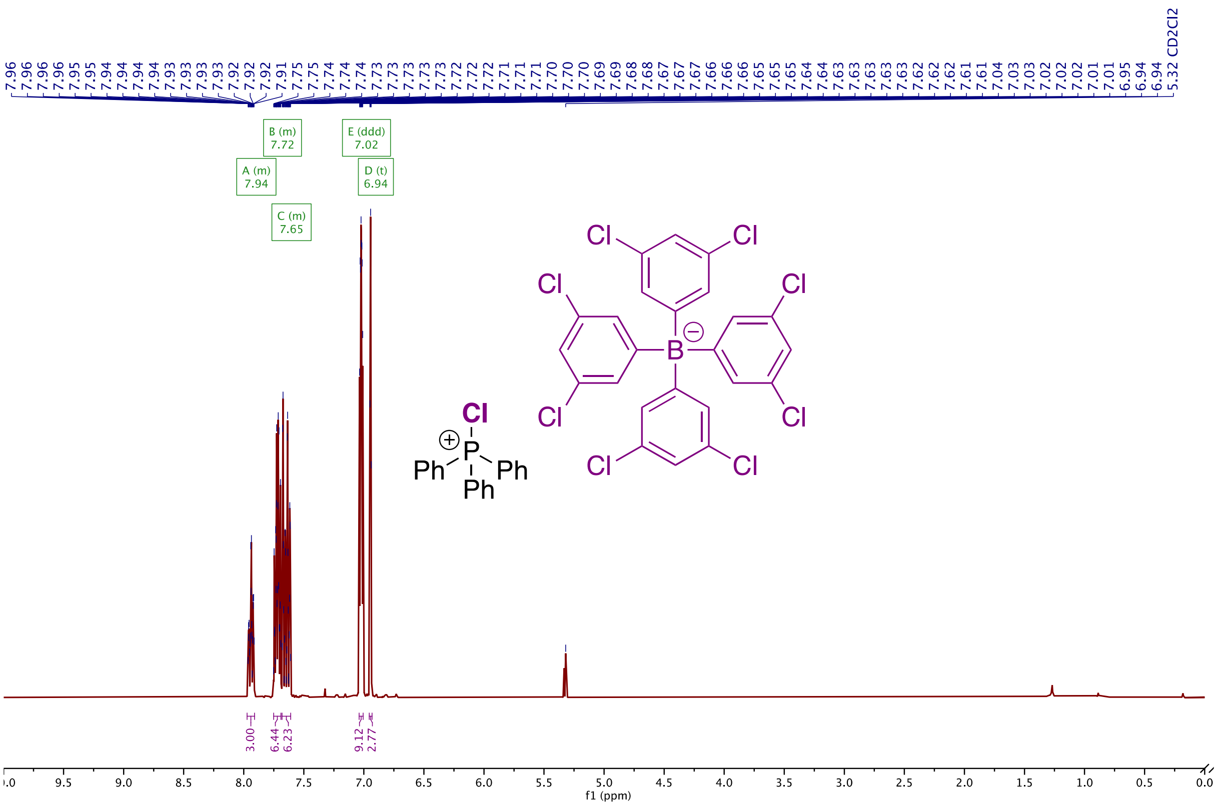

Figure S19: ^{13}C NMR of chlorotriphenyl-λ^4-phosphane tetrakis(3,5-dichlorophenyl)borate, **2c**.

PIJ401-FP_20181112_01/CARBON_01.fid/fid
Solvent: cd2cl2, Scans: 5000, Relaxation: 2.0000

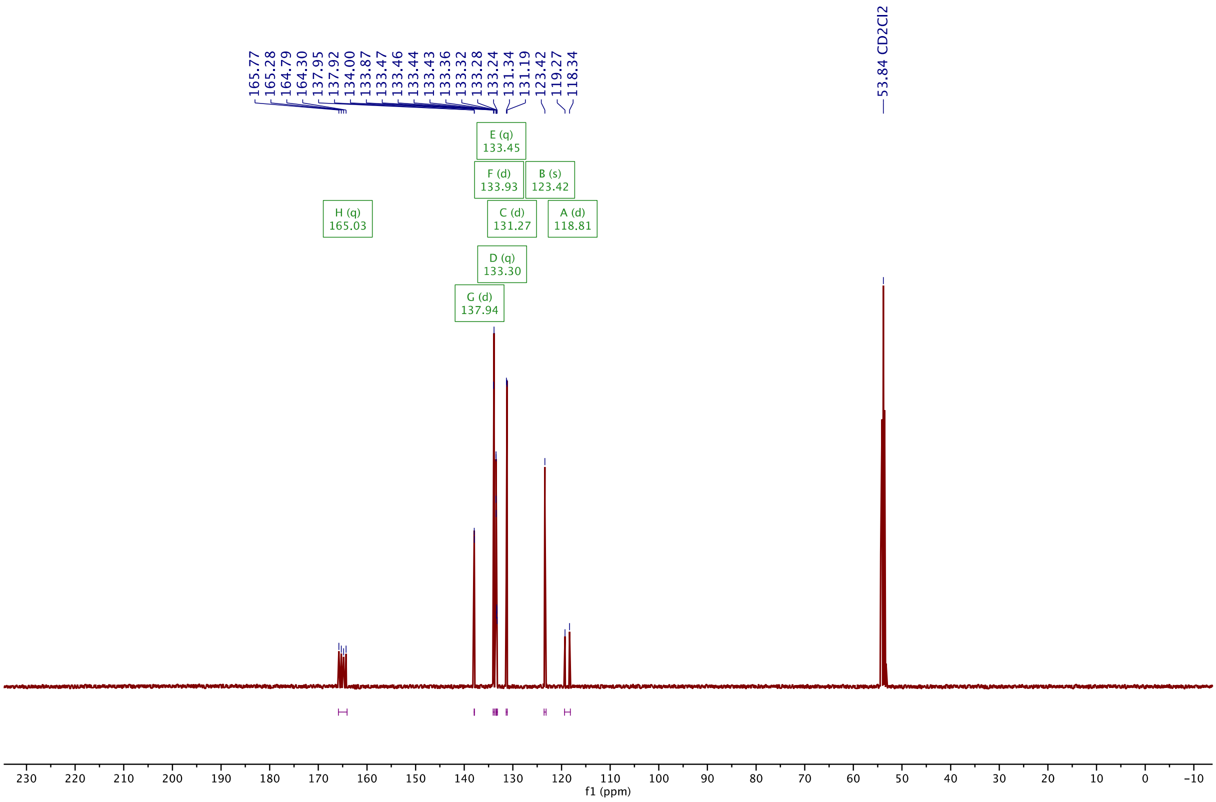

Figure S20: ^{31}P NMR of chlorotriphenyl-λ^4-phosphane tetrakis(3,5-dichlorophenyl)borate, **2c**.

PIJ401-FP_20181112_01/PHOSPHORUS_02.fid/fid
Solvent: cd2cl2, Scans: 512, Relaxation: 2.0000

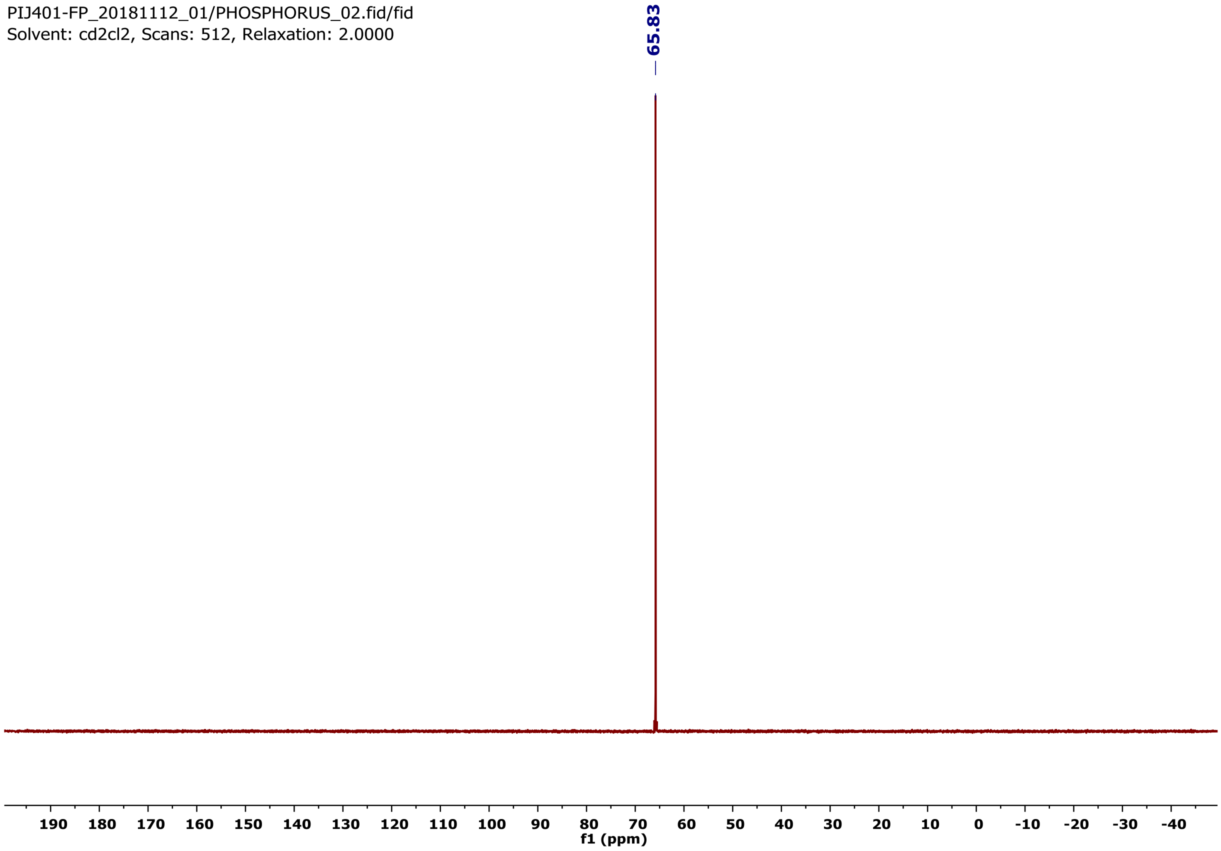

Figure S21: ^{11}B NMR of chlorotriphenyl-λ^4-phosphane tetrakis(3,5-dichlorophenyl)borate, **2c**.

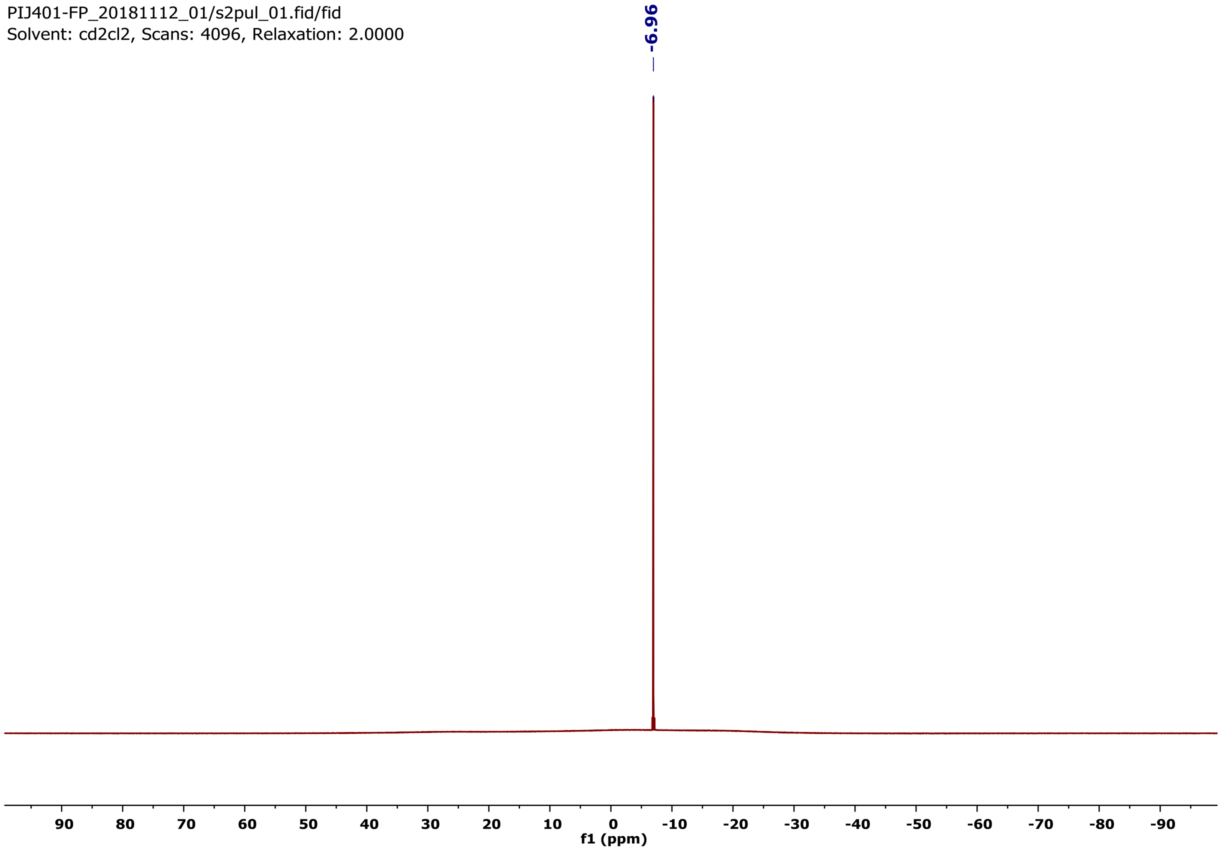

Figure S22: ^{1}H NMR of triphenylphosphine, **3**:[3]

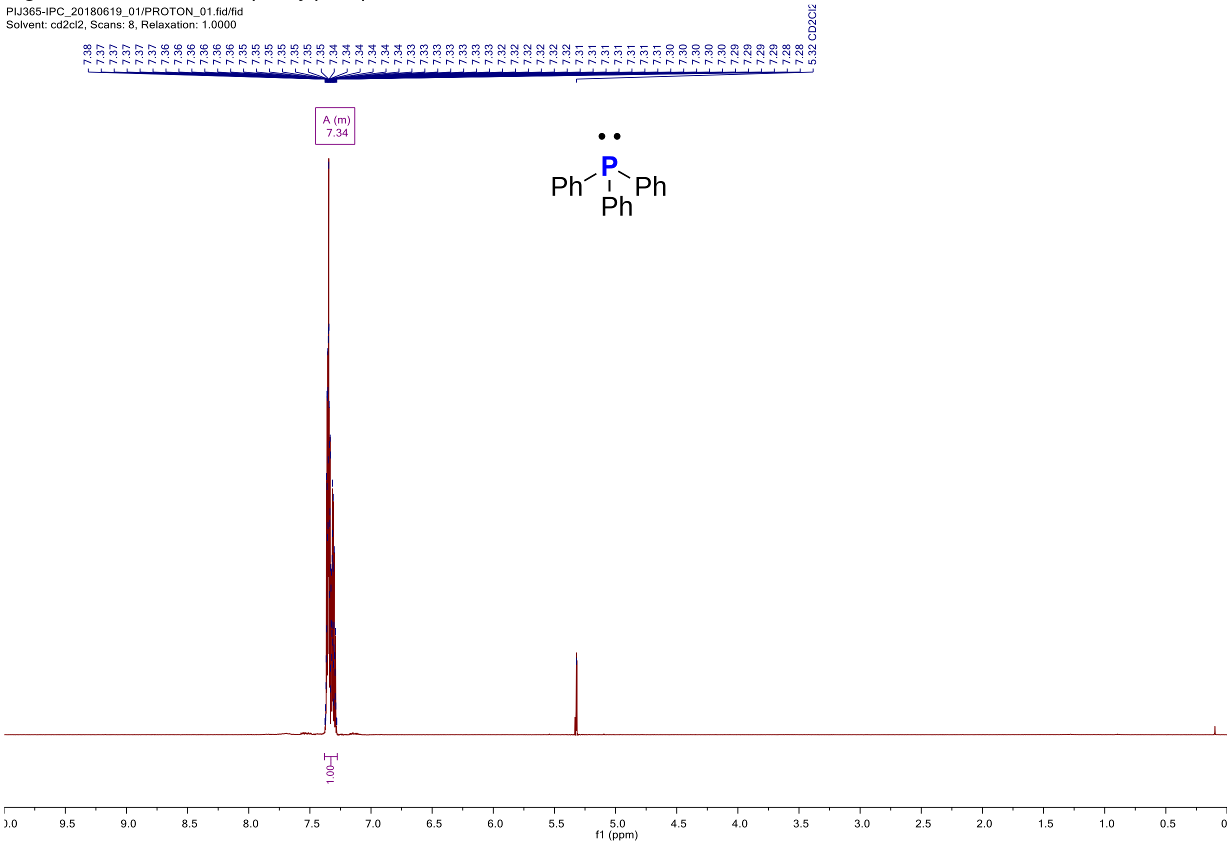

Figure S23: ^{31}P NMR of triphenylphosphine, **3**.[3]

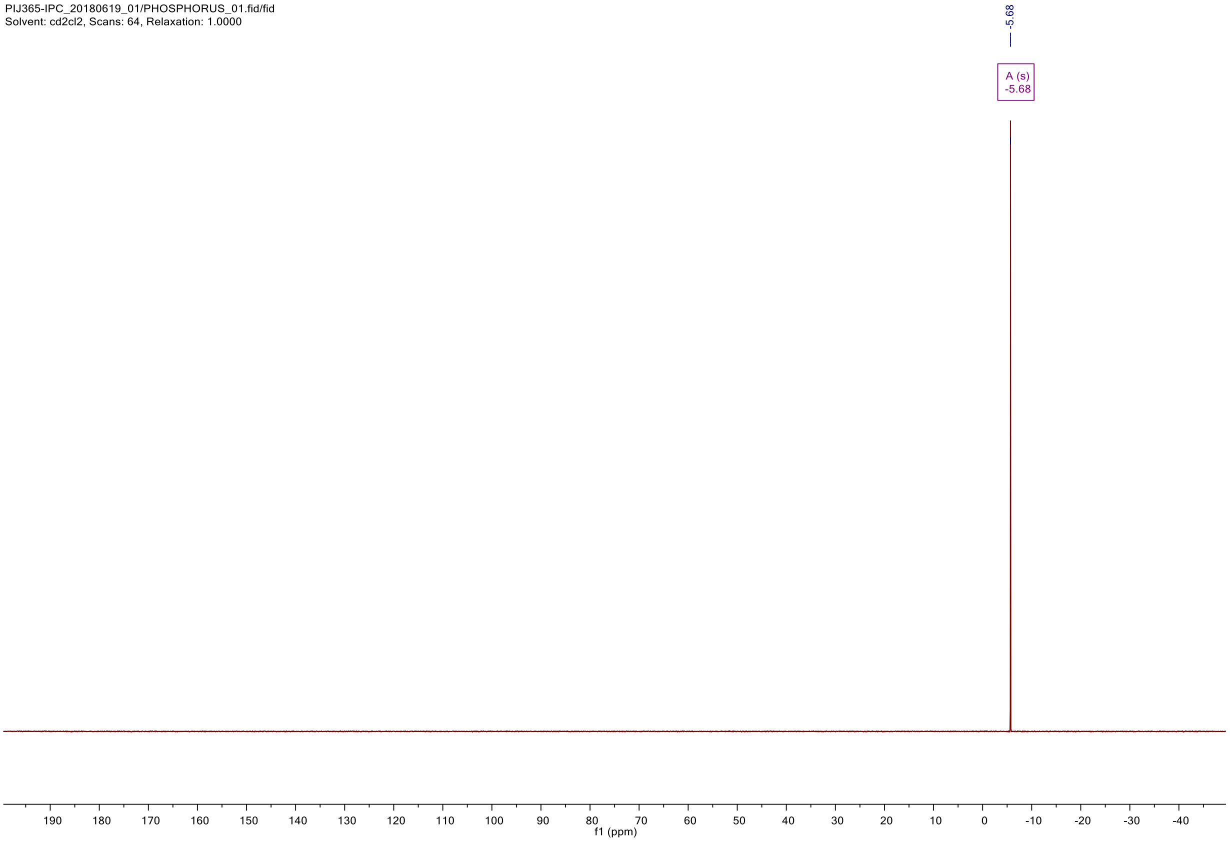

Figure S24: ^{1}H NMR of dichlorotricyclohexyl-λ^4-phosphane.

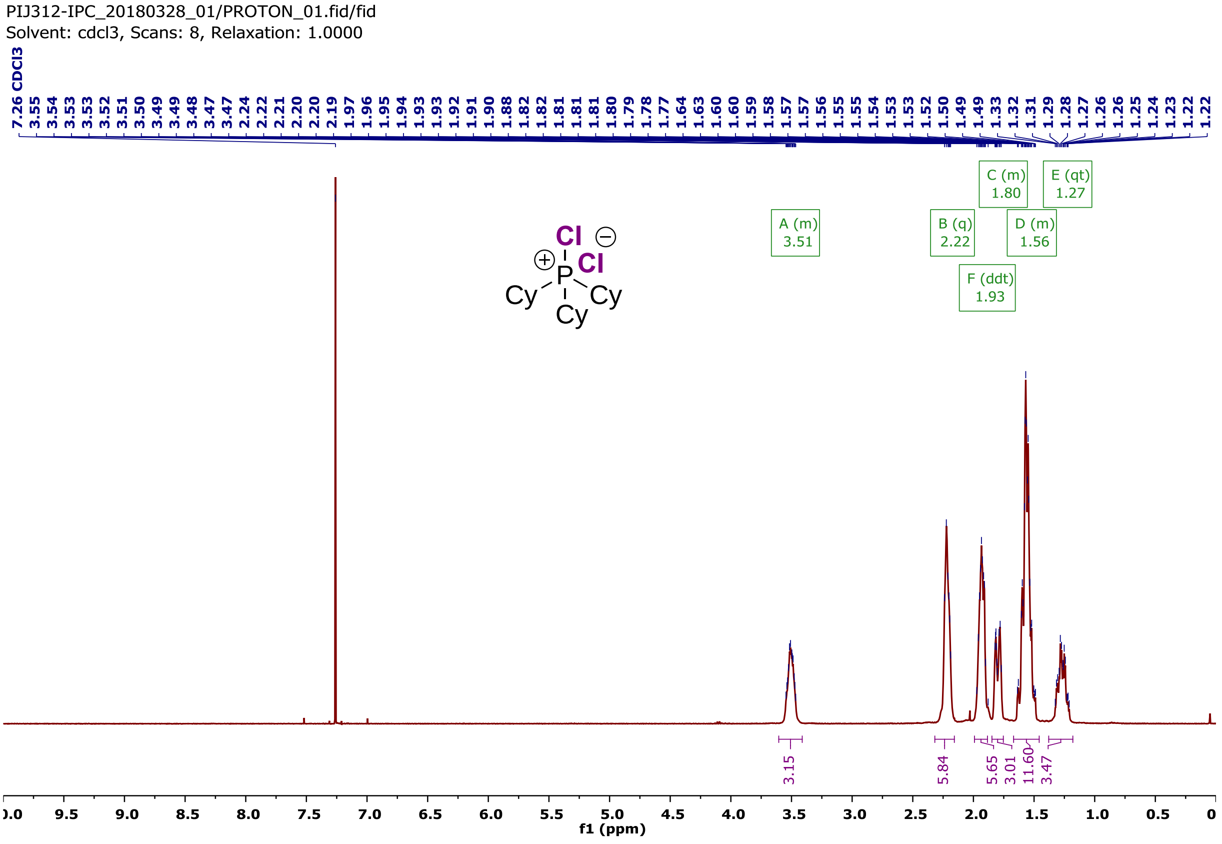

Figure S25: ^{31}P NMR of dichlorotricyclohexyl-λ^4-phosphane.

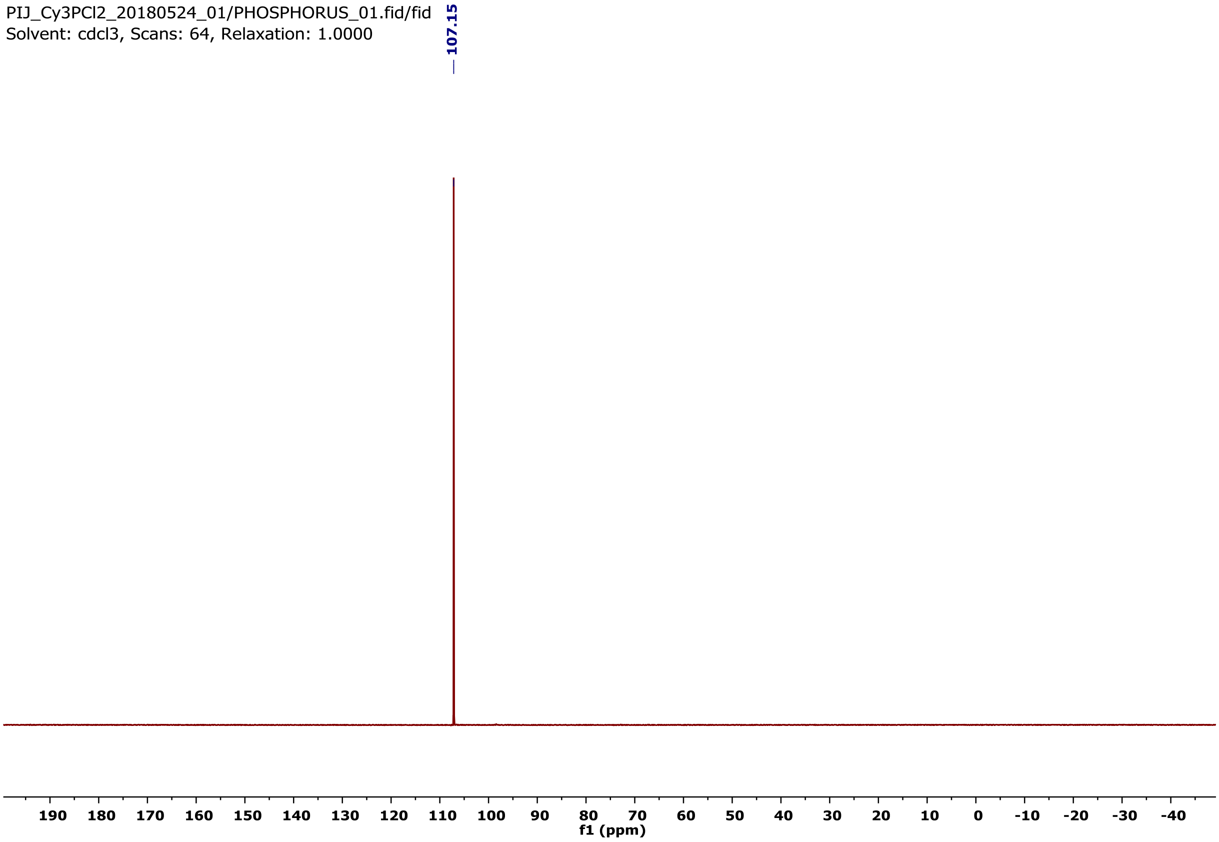

Figure S26: ^{1}H NMR of Tricyclohexylphosphane, **7**:[3]

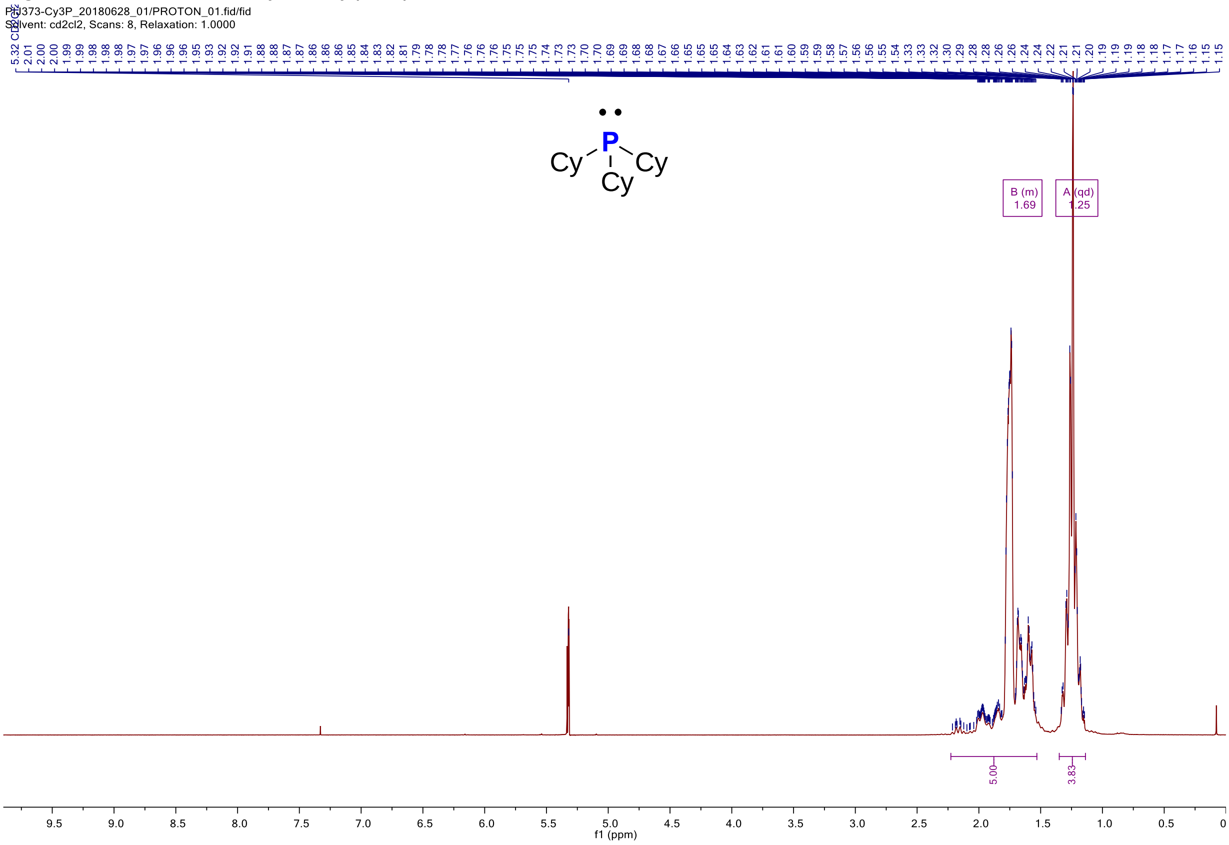

Figure S27: ^{31}P NMR of Tricyclohexylphosphane, **7**.[3]

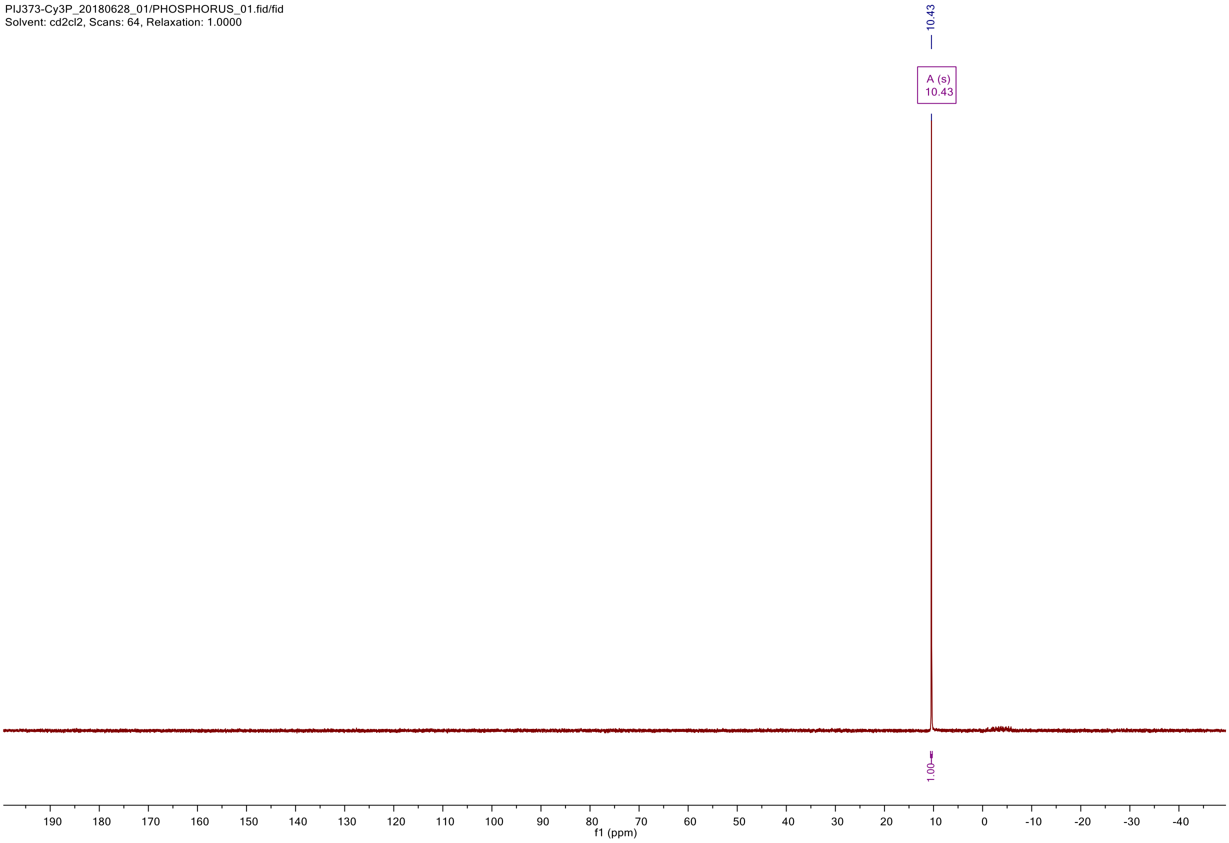

Figure S28: ^{1}H NMR of 1-chloro-4-methyl-1-phenyl-2,3-dihydro-1H-phosphol-1-ium chloride.[1]

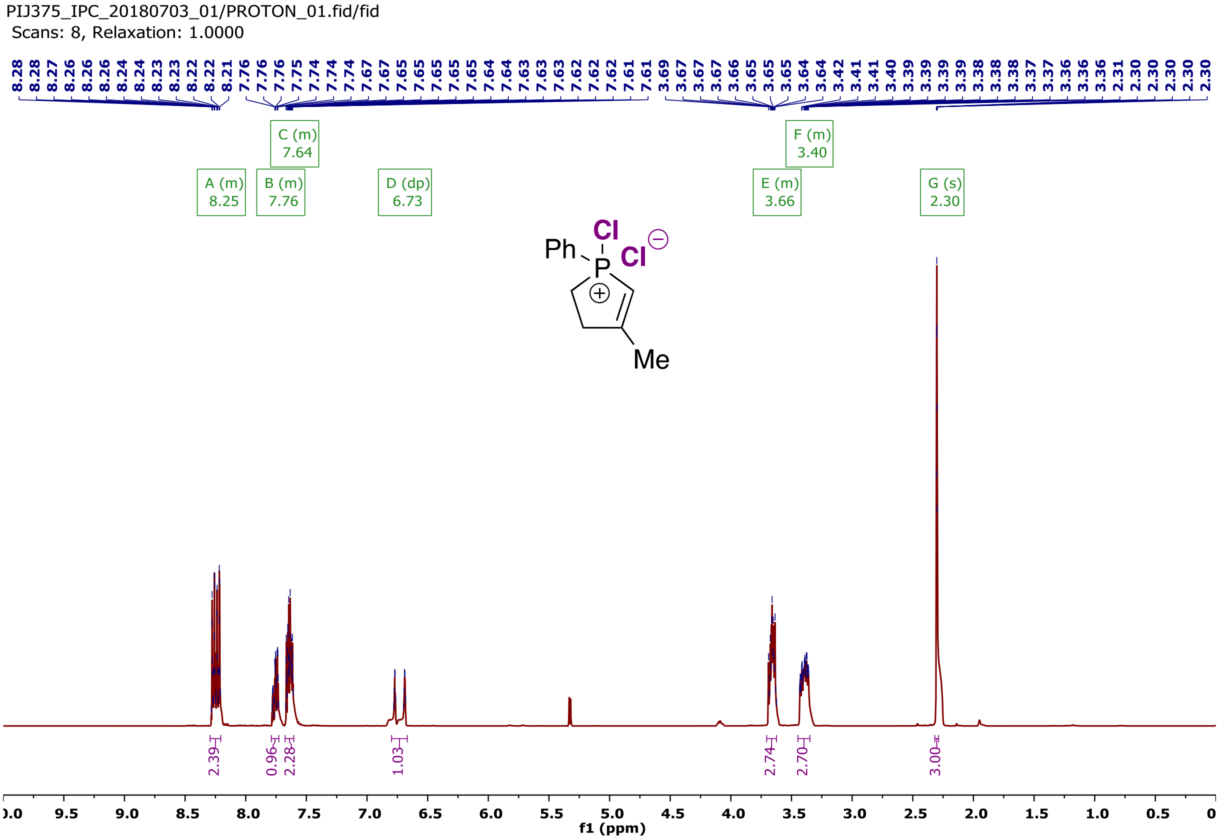

Figure S29: ^{1}H NMR of 1-chloro-4-methyl-1-phenyl-2,3-dihydro-1H-phosphol-1-ium chloride.[1]

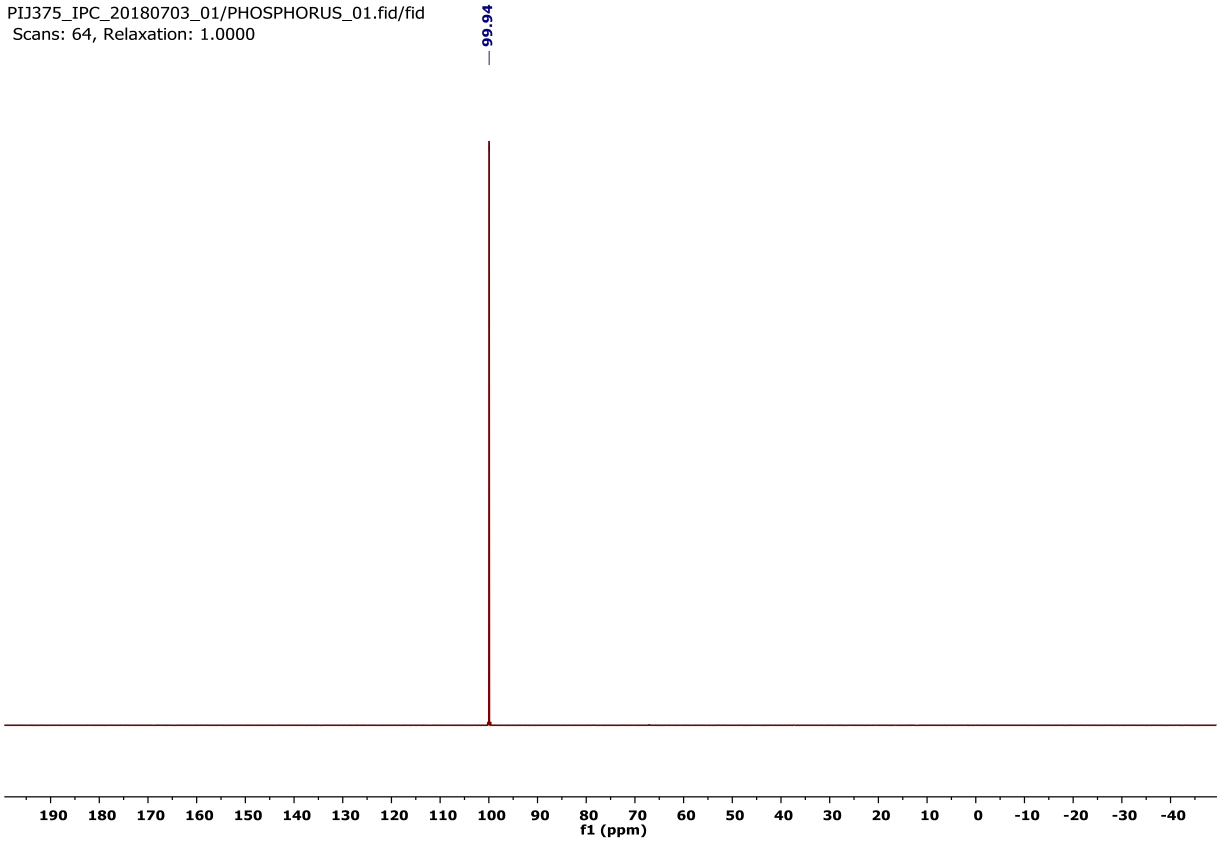

Figure S30: ^{1}H NMR of 4-methyl-1-phenyl-2,3-dihydro-1H-phosphole, **8**.[4]

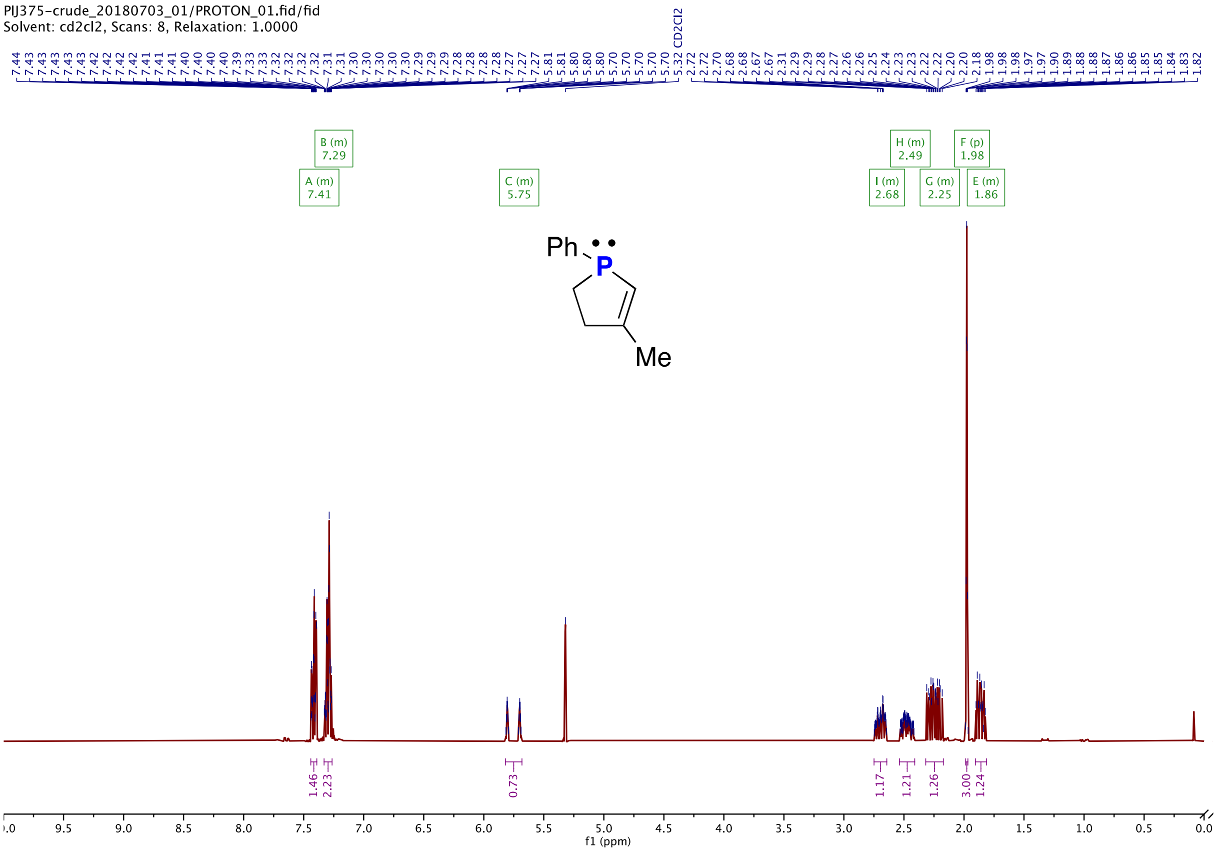

Figure S31: ^{31}P NMR of 4-methyl-1-phenyl-2,3-dihydro-1H-phosphole, **8**:[4]

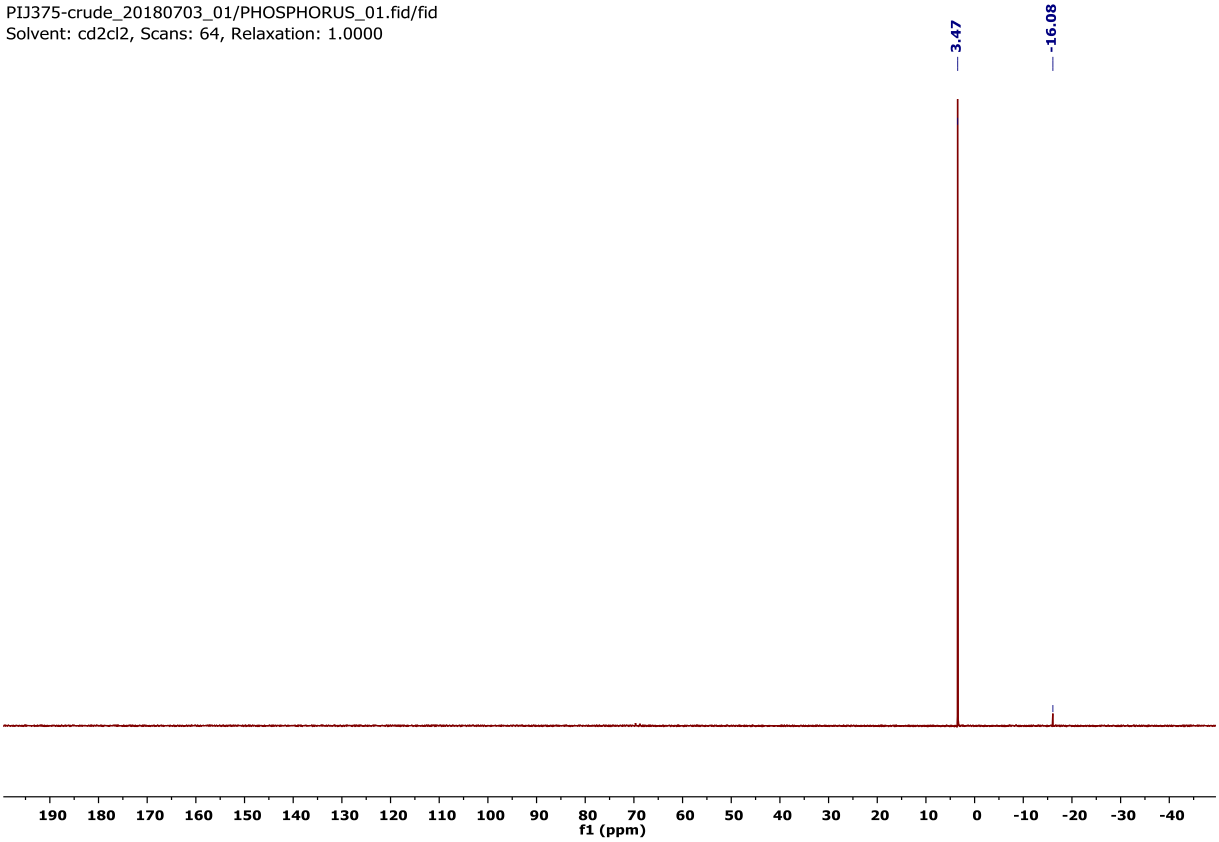

Figure S32: ^{1}H NMR of chloro(diisopropylamino)diphenylphosphonium chloride.[5]

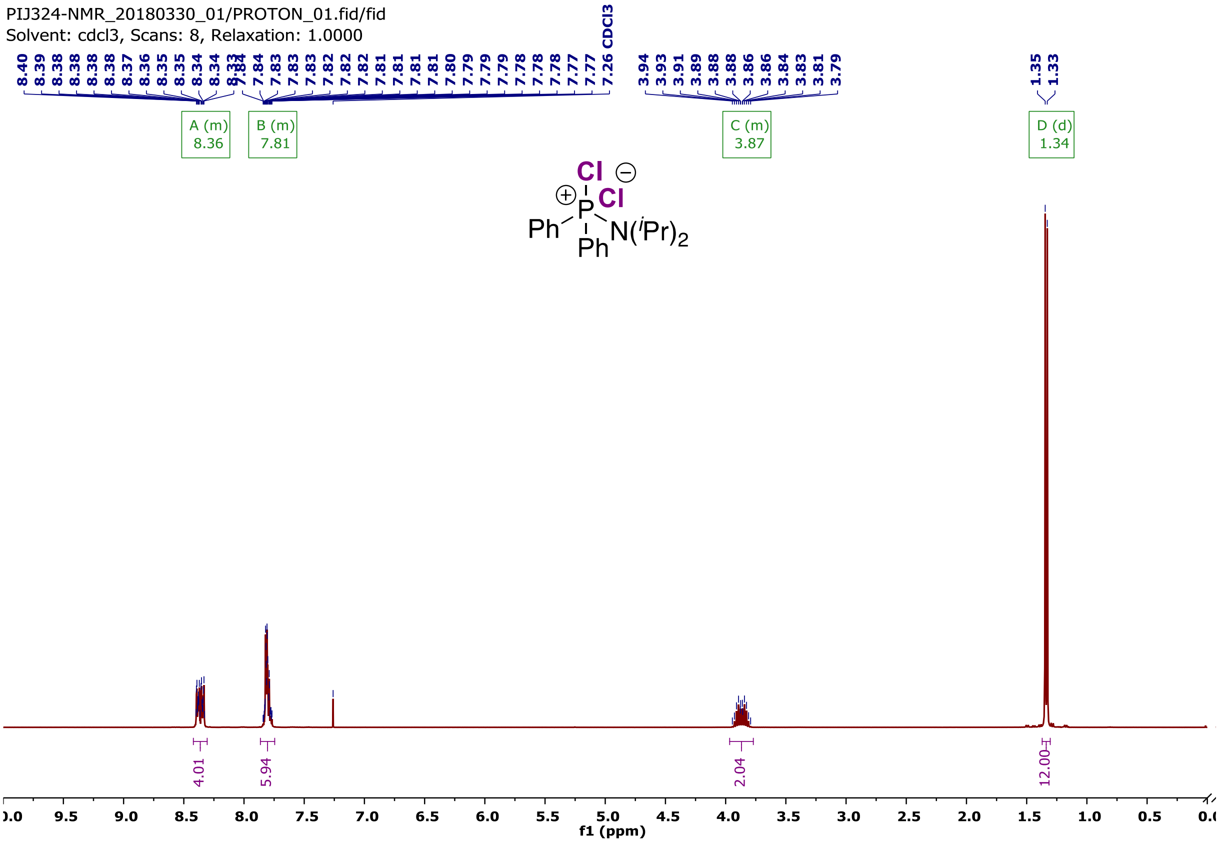

Figure S33: ^{31}P NMR of chloro(diisopropylamino)diphenylphosphonium chloride.[5]

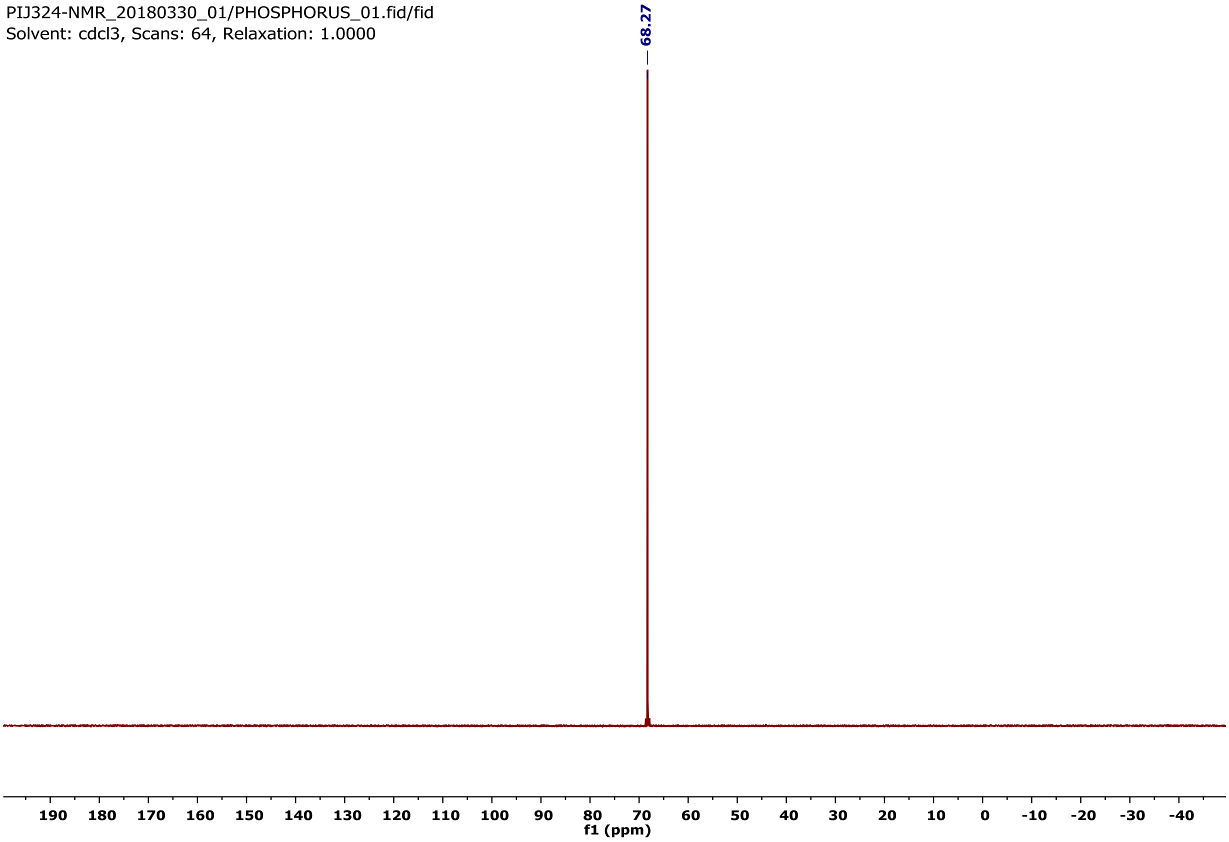

Figure S34: ^{1}H NMR of *N*,*N*-diisopropyl-1,1-diphenylphosphanamine, **9**.[5]

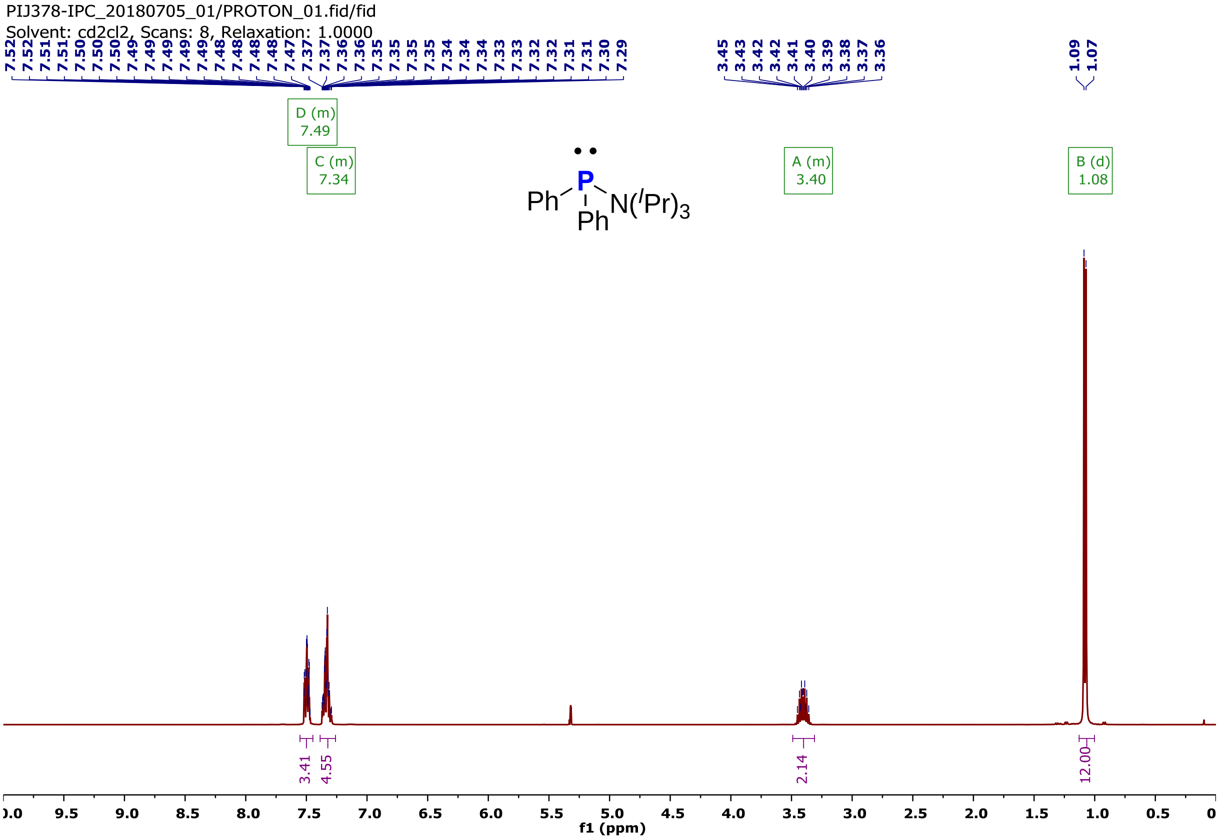

Figure S35: ^{31}P NMR of *N*,*N*-diisopropyl-1,1-diphenylphosphanamine, **9**.[5]

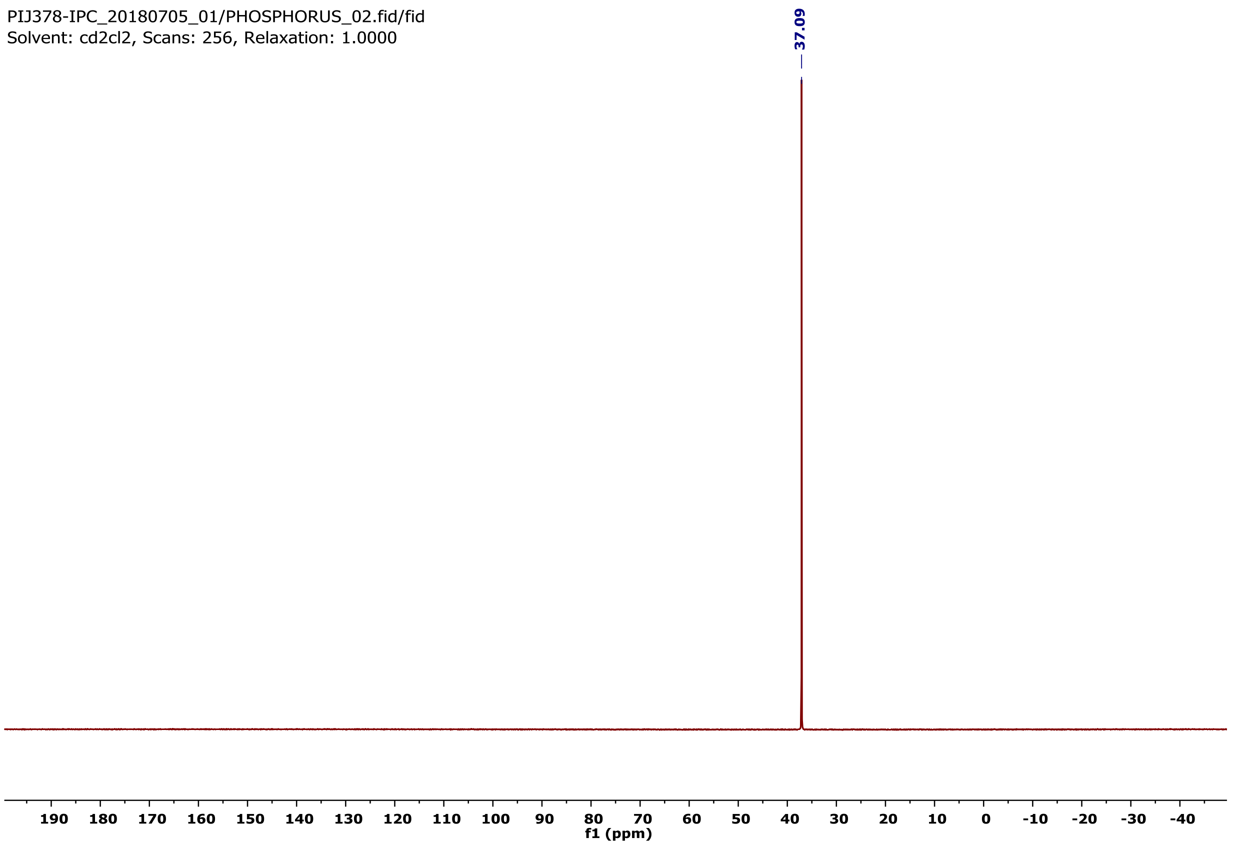

Figure S36: ^{1}H NMR of 1-Chloro-3-methyl-1-phenyl-2-phospholenium chloride.

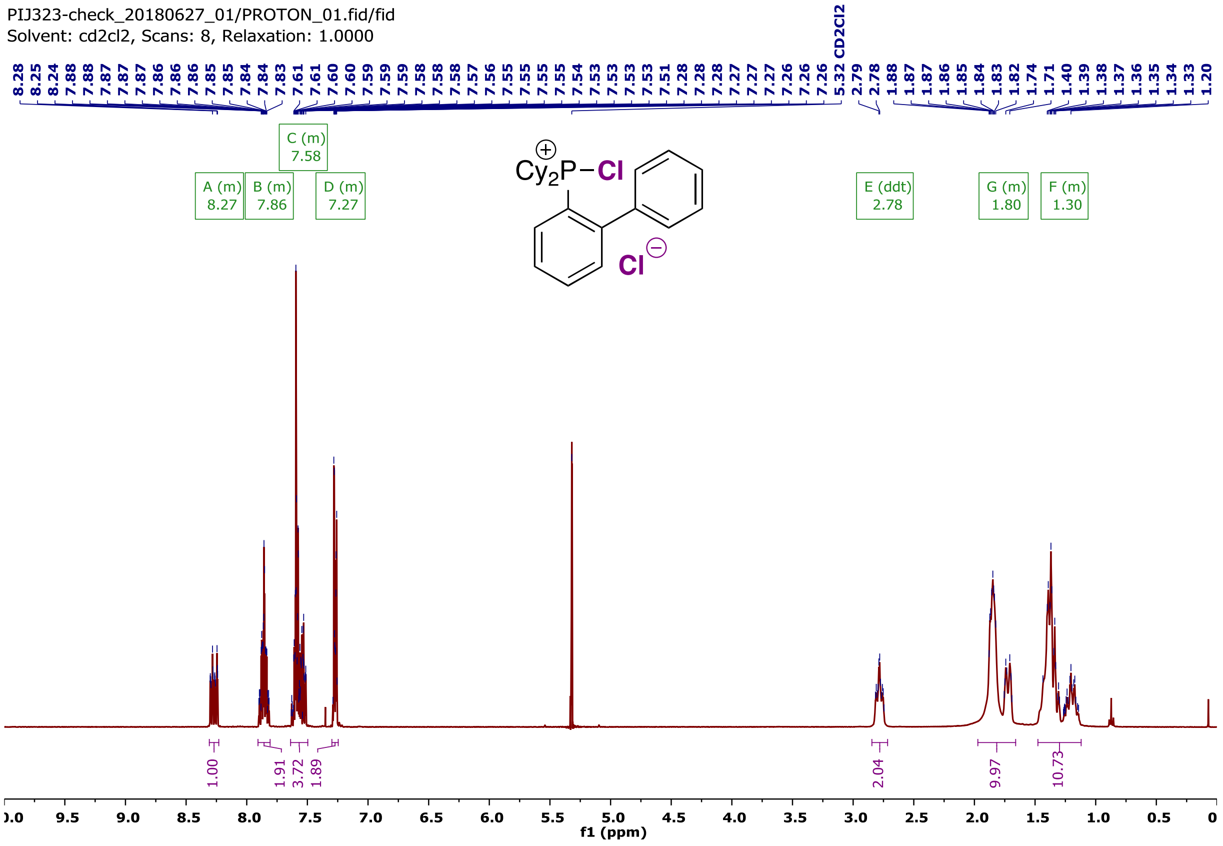

Figure S37: ^{31}P NMR of 1-Chloro-3-methyl-1-phenyl-2-phospholenium chloride.

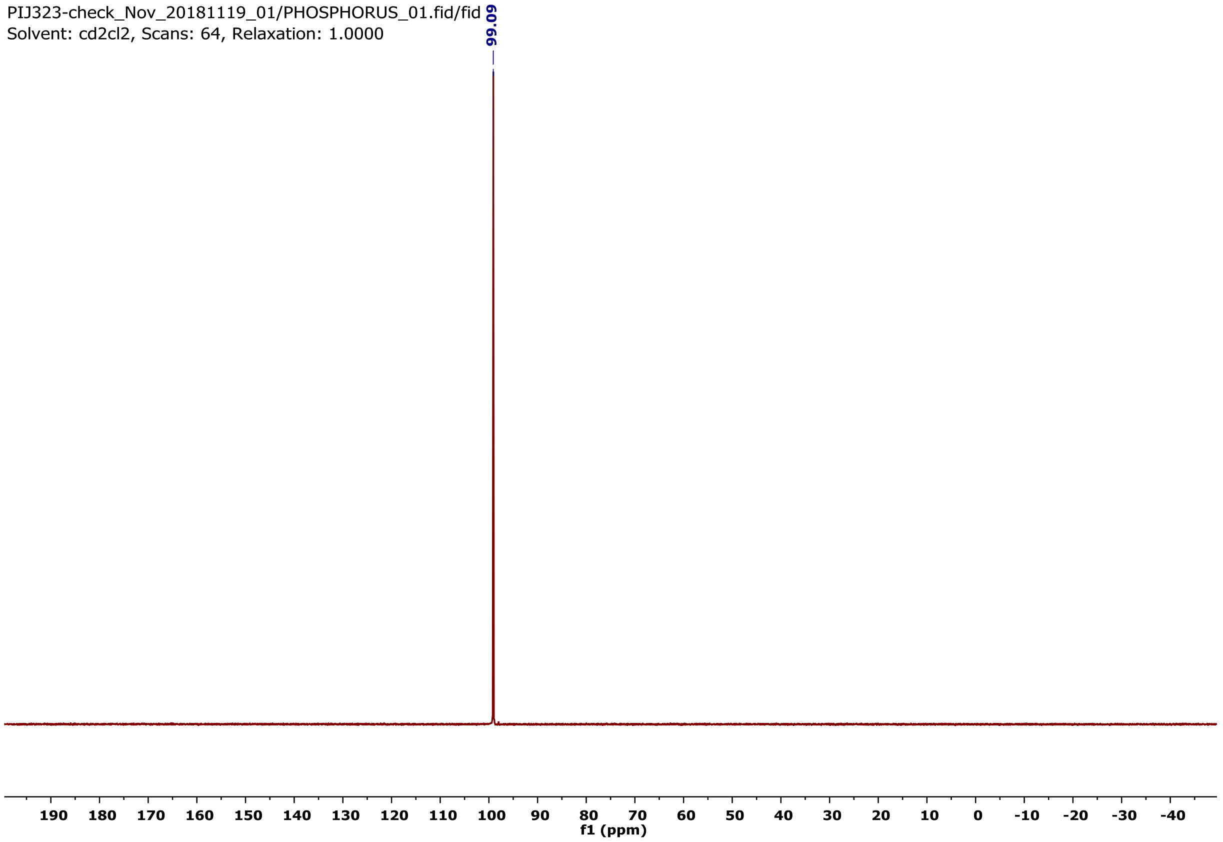

Figure S38: ^{1}H NMR of CyJohnPhos, **10**.[3a]

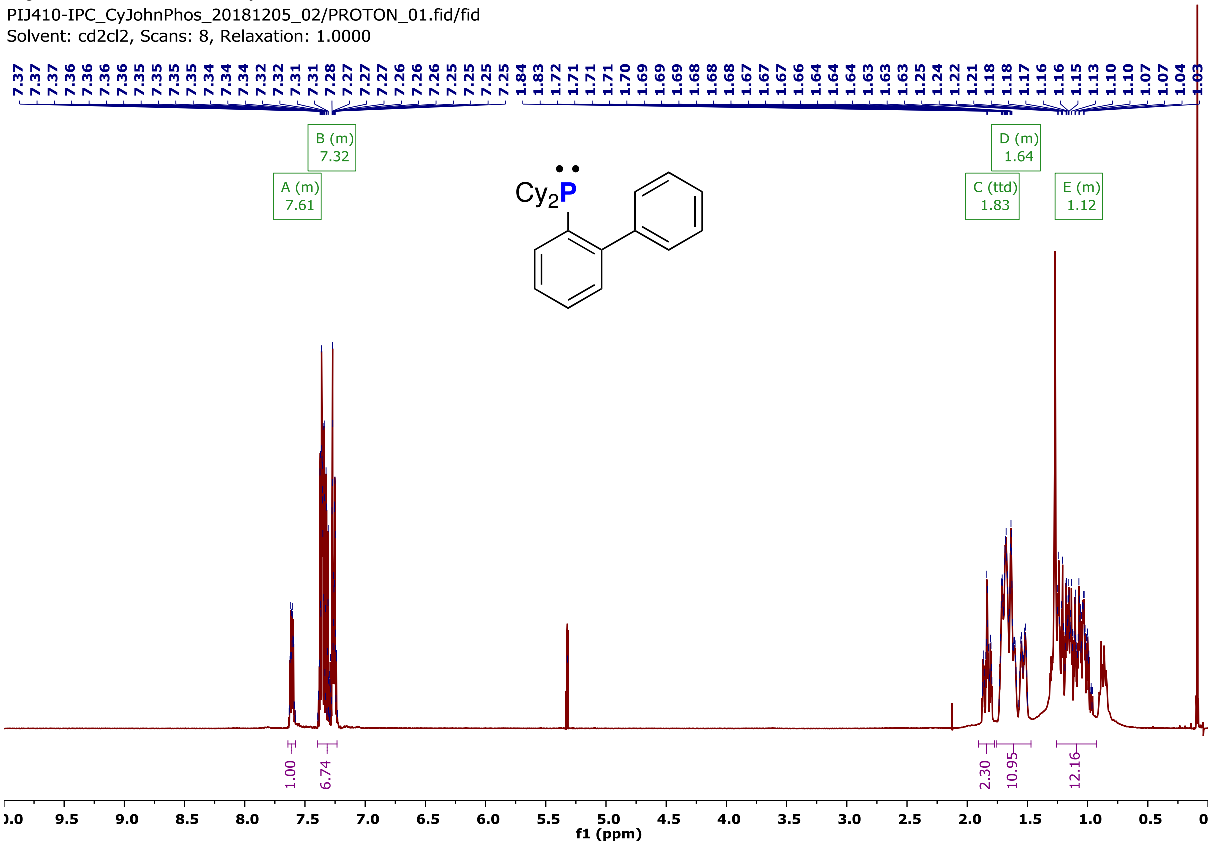

Figure S39: ^{31}P NMR of CyJohnPhos, **10**.[3a]

PIJ410-checkA_20181206_01/PHOSPHORUS_01.fid/fid
Solvent: cd2cl2, Scans: 64, Relaxation: 1.0000

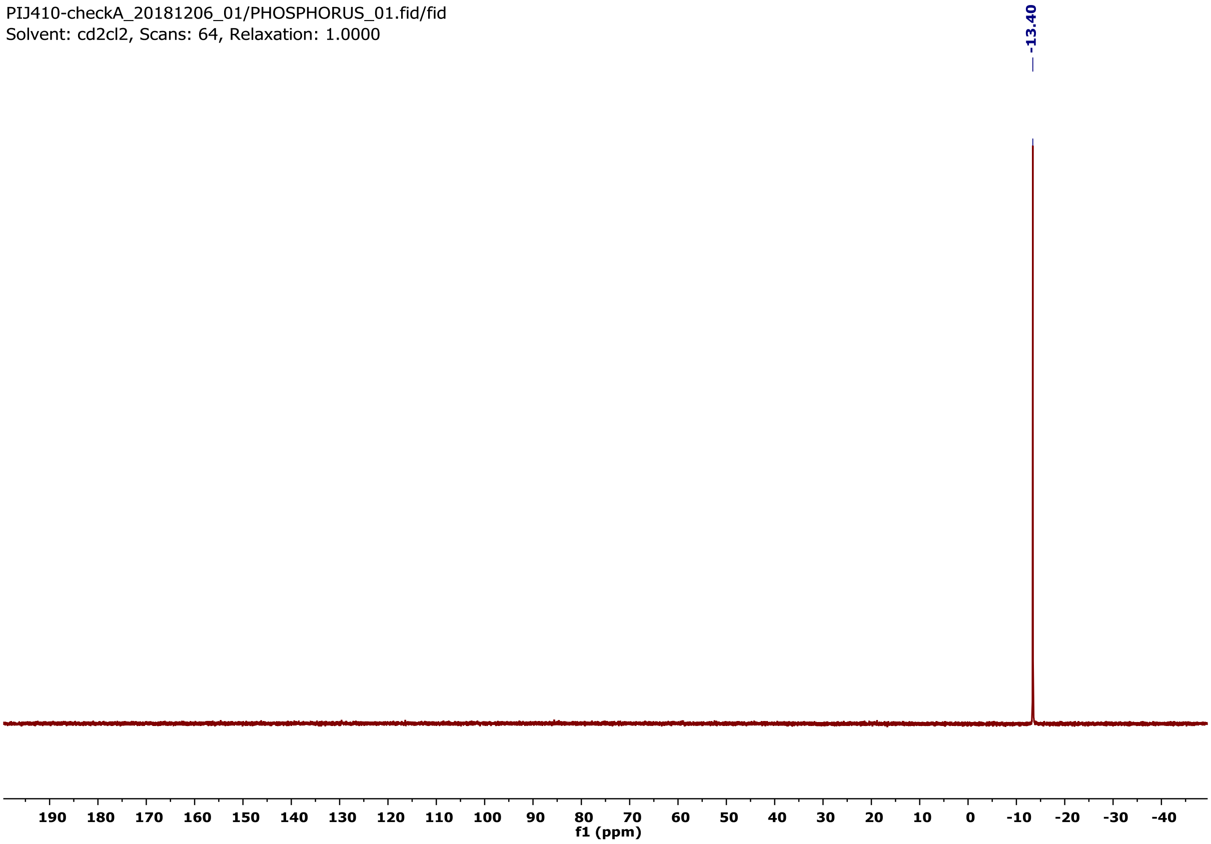

Figure S40: ^{1}H NMR of 2'-(dichlorodicyclohexyl- λ^5-phosphaneyl)-*N,N*-dimethyl-[1,1'-biphenyl]-2-amine.

PIJ321-NMR_20180330_01/PROTON_01.fid/fid
Solvent: cdcl3, Scans: 8, Relaxation: 1.0000

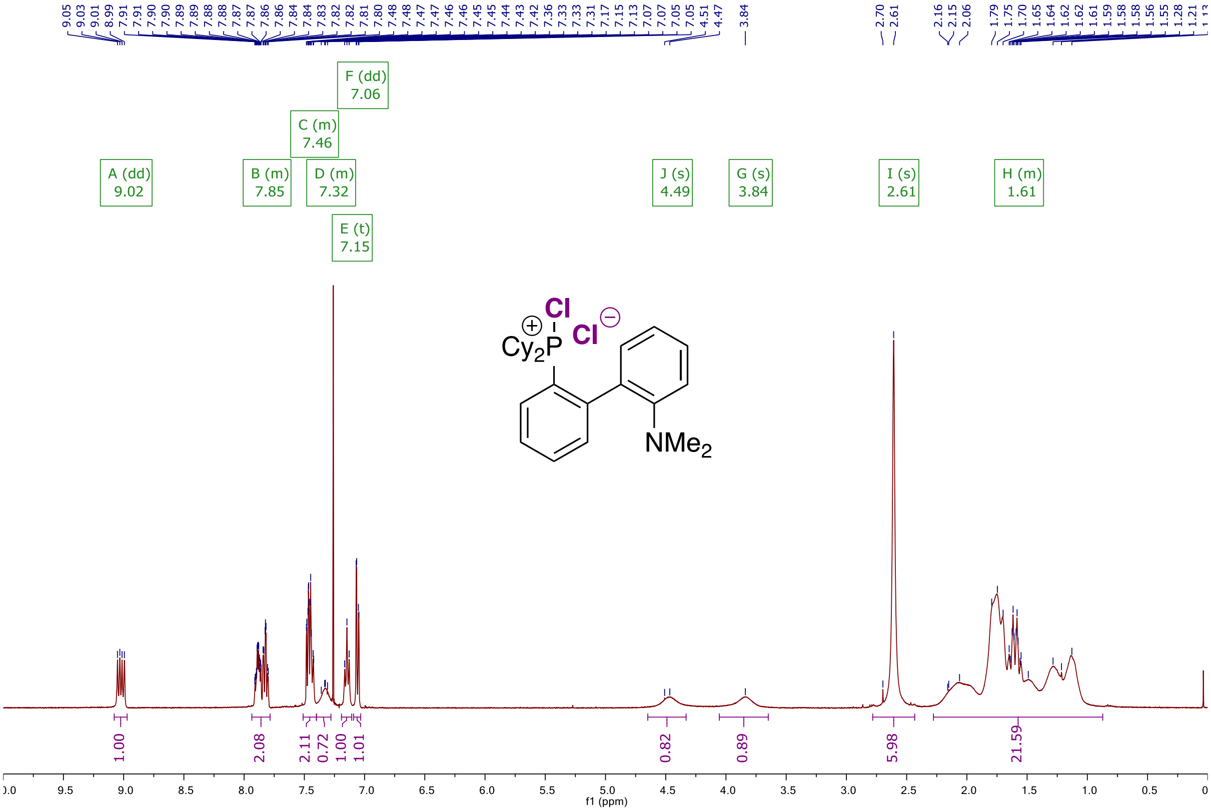

Figure S41: ^{31}P NMR of 2'-(dichlorodicyclohexyl- λ^5-phosphaneyl)-*N,N*-dimethyl-[1,1'-biphenyl]-2-amine.

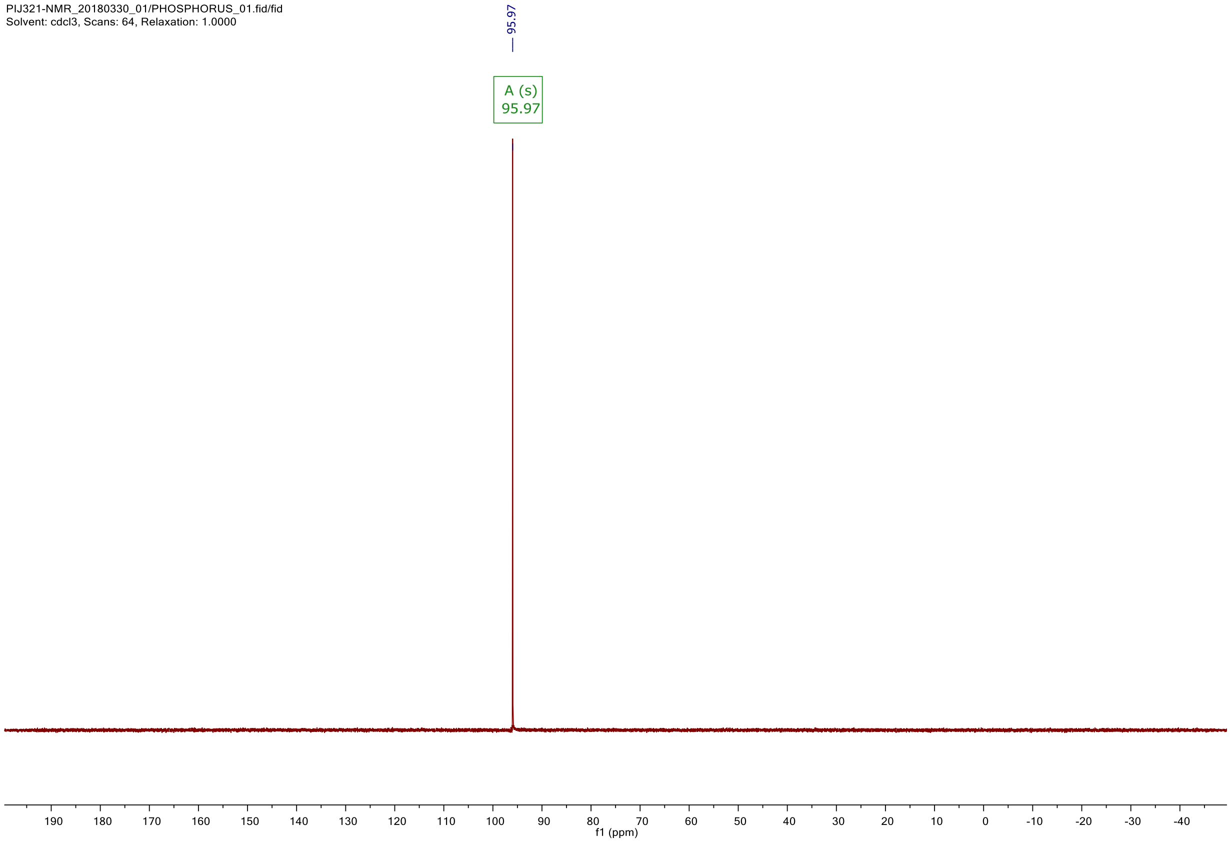

Figure S42: ^{1}H NMR of 2-Dicyclohexylphosphino-2′-(*N,N*-dimethylamino)biphenyl, (DavePhos), **11**.[6]

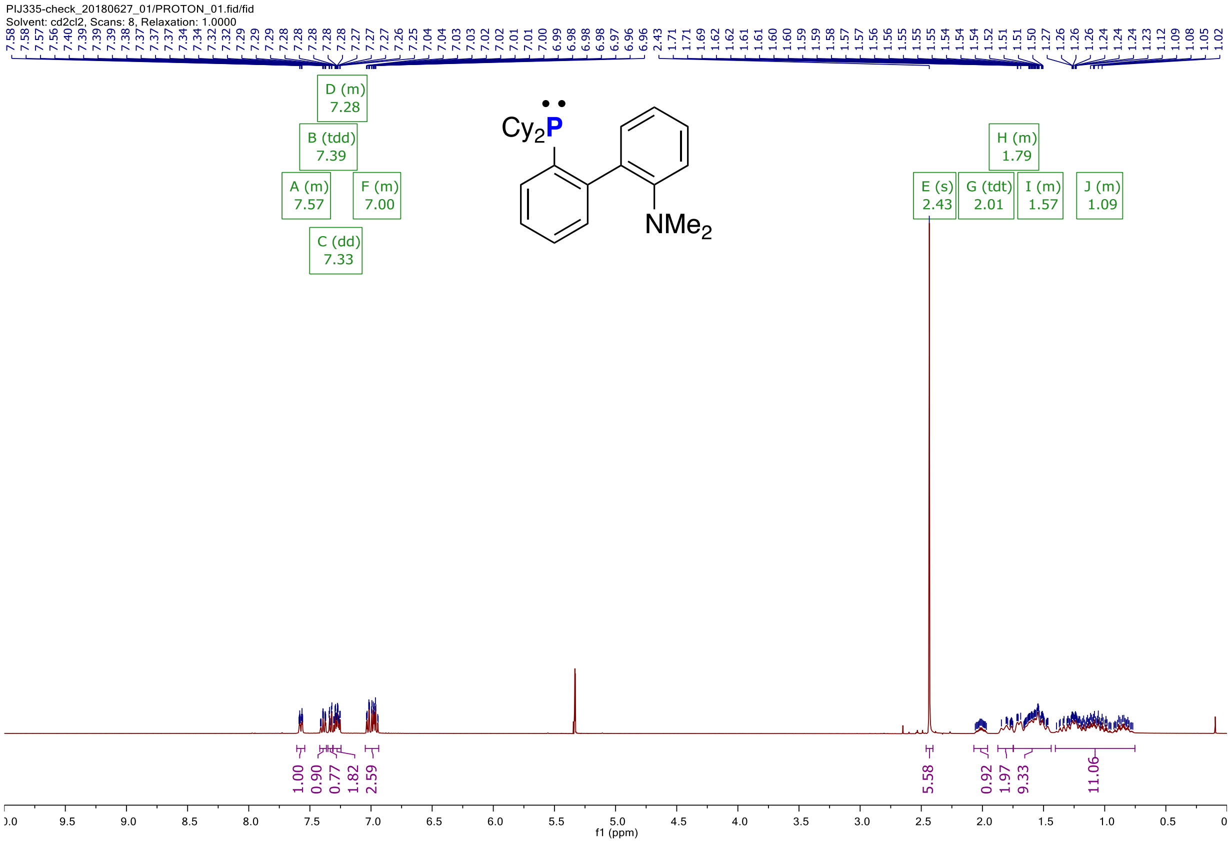

Figure S43: ^{31}P NMR of 2-Dicyclohexylphosphino-2′-(*N*,*N*-dimethylamino)biphenyl, (DavePhos), **11**.[6]

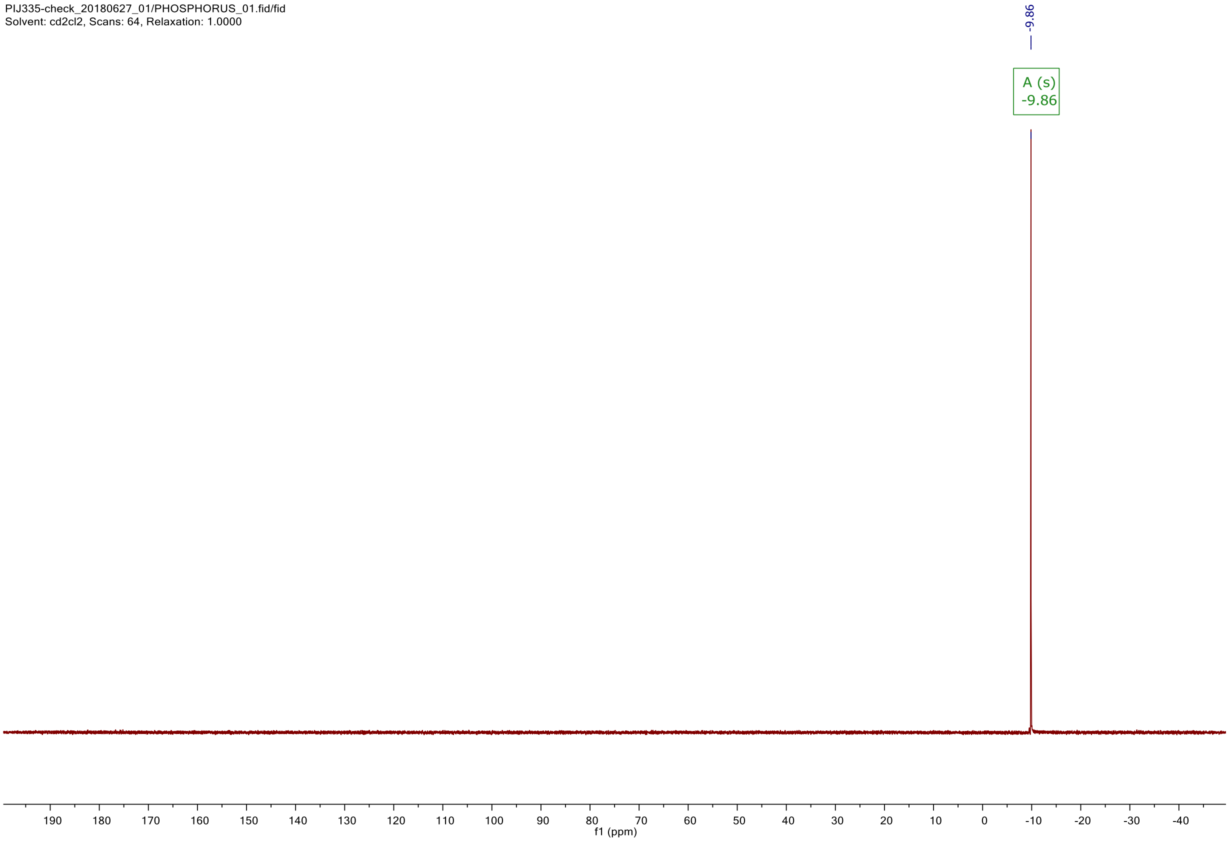

Figure S44: ^{1}H NMR of 4-chloro-4-phenyl-4,5-dihydro-3H-dinaphtho[2,1-c:1',2'-e]phosphepin-4-ium.

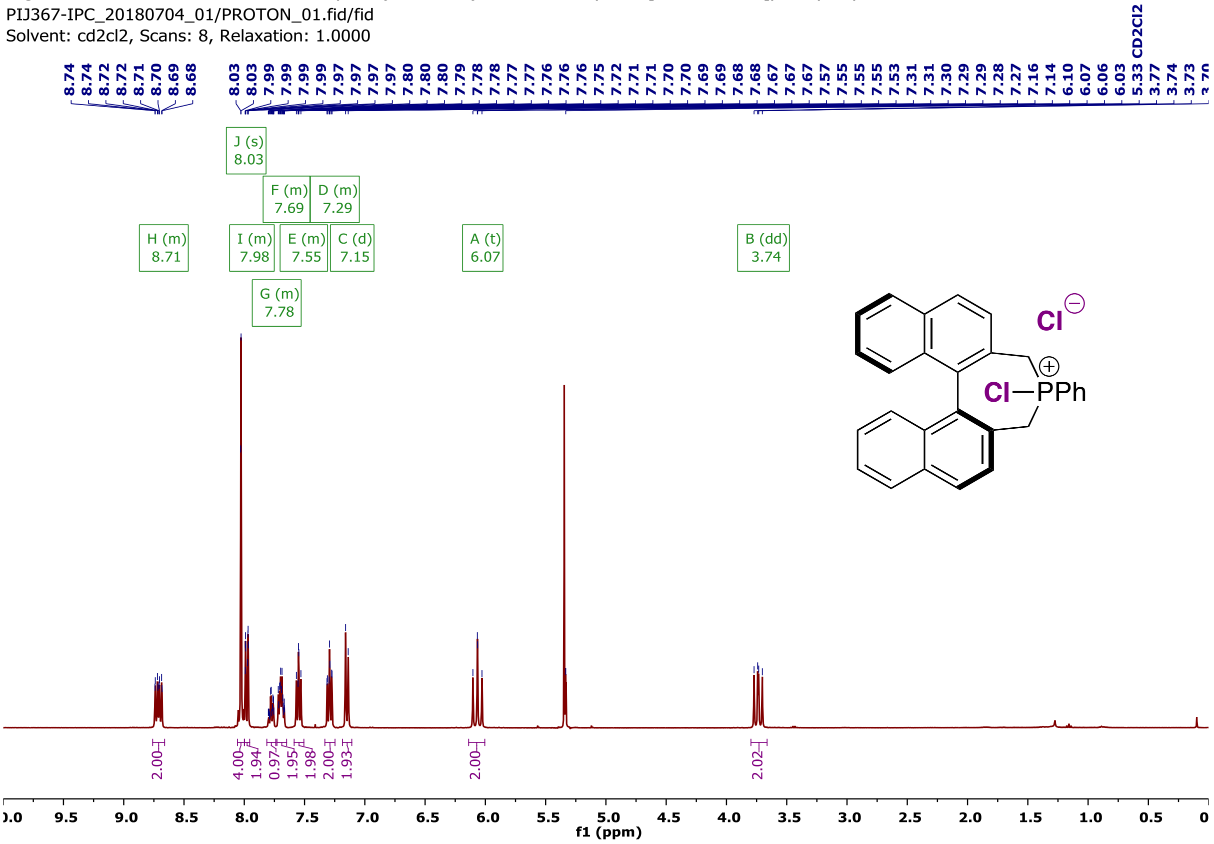

Figure S45: ^{31}P NMR of 4-chloro-4-phenyl-4,5-dihydro-3H-dinaphtho[2,1-c:1',2'-e]phosphepin-4-ium.

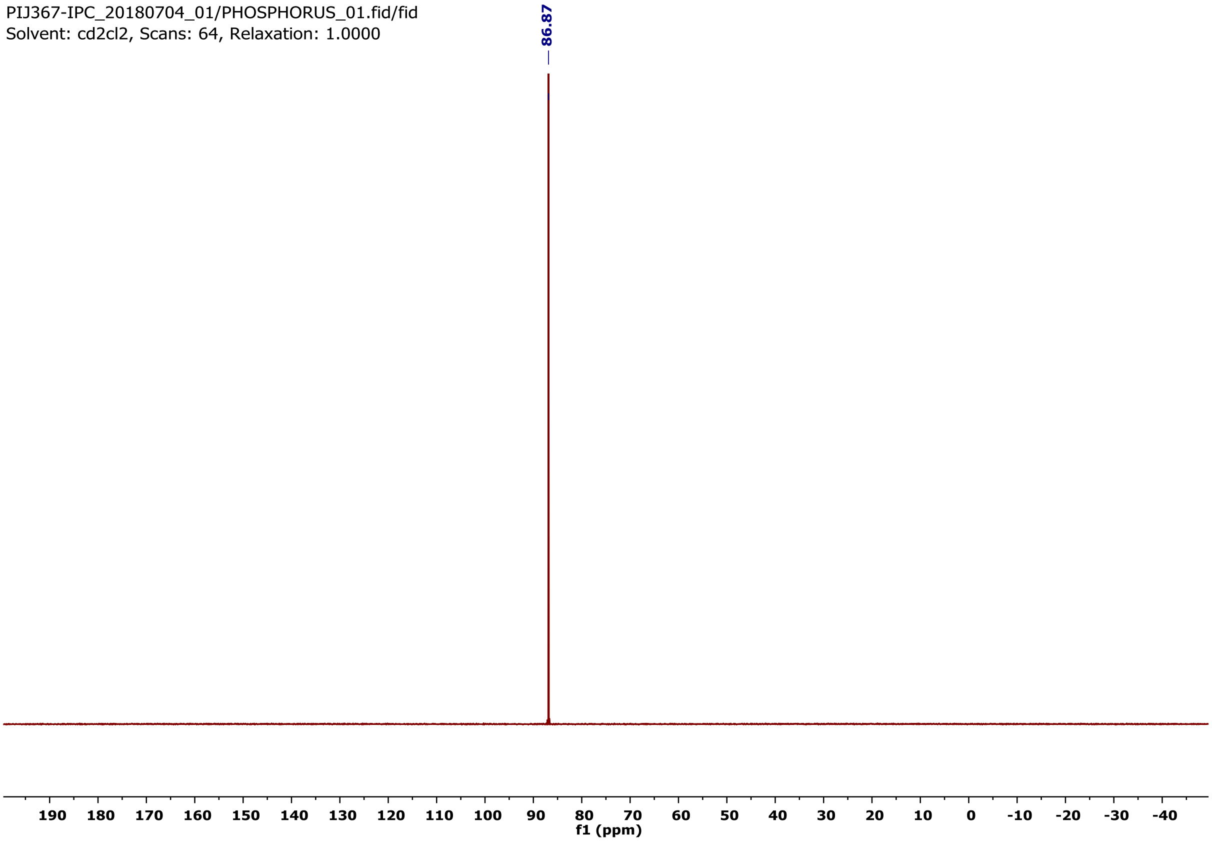

Figure S46: ^{1}H NMR of (*S*)-Ph-BINEPINE, **12**.[7]

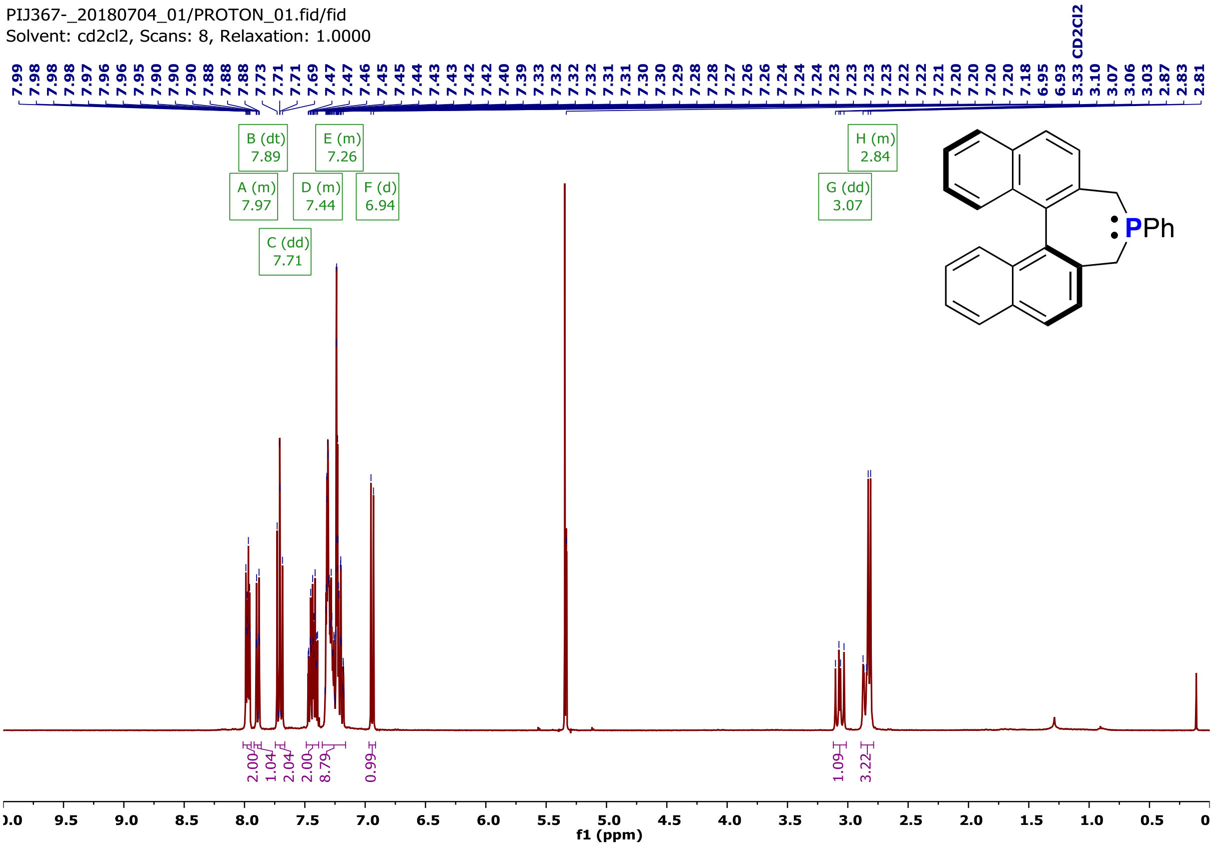

Figure S47: ^{31}P NMR of (*S*)-Ph-BINEPINE, **12**.[7]

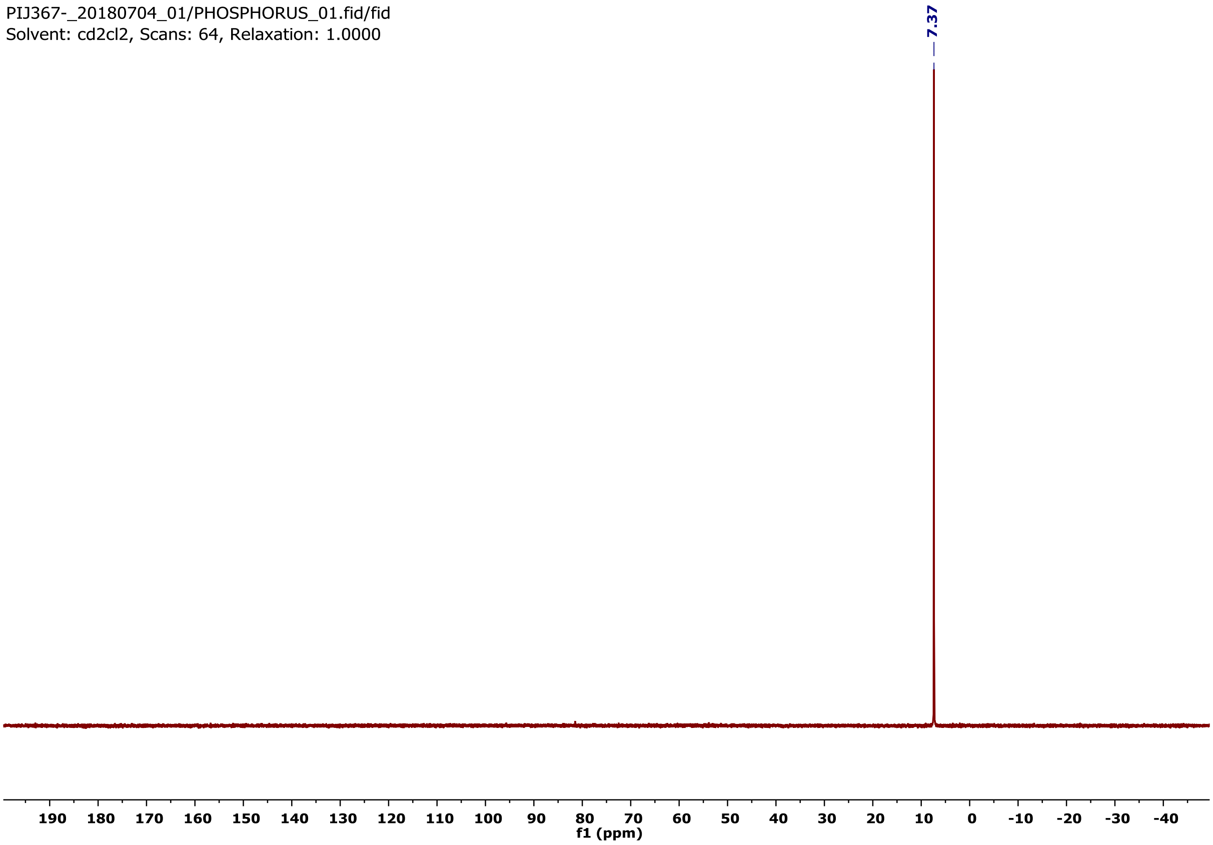

Figure S48: ^{1}H NMR of 4-chloro-4-phenyl-4,5-dihydro-3H-dinaphtho[2,1-c:1',2'-e]phosphepin-4-ium chloride.

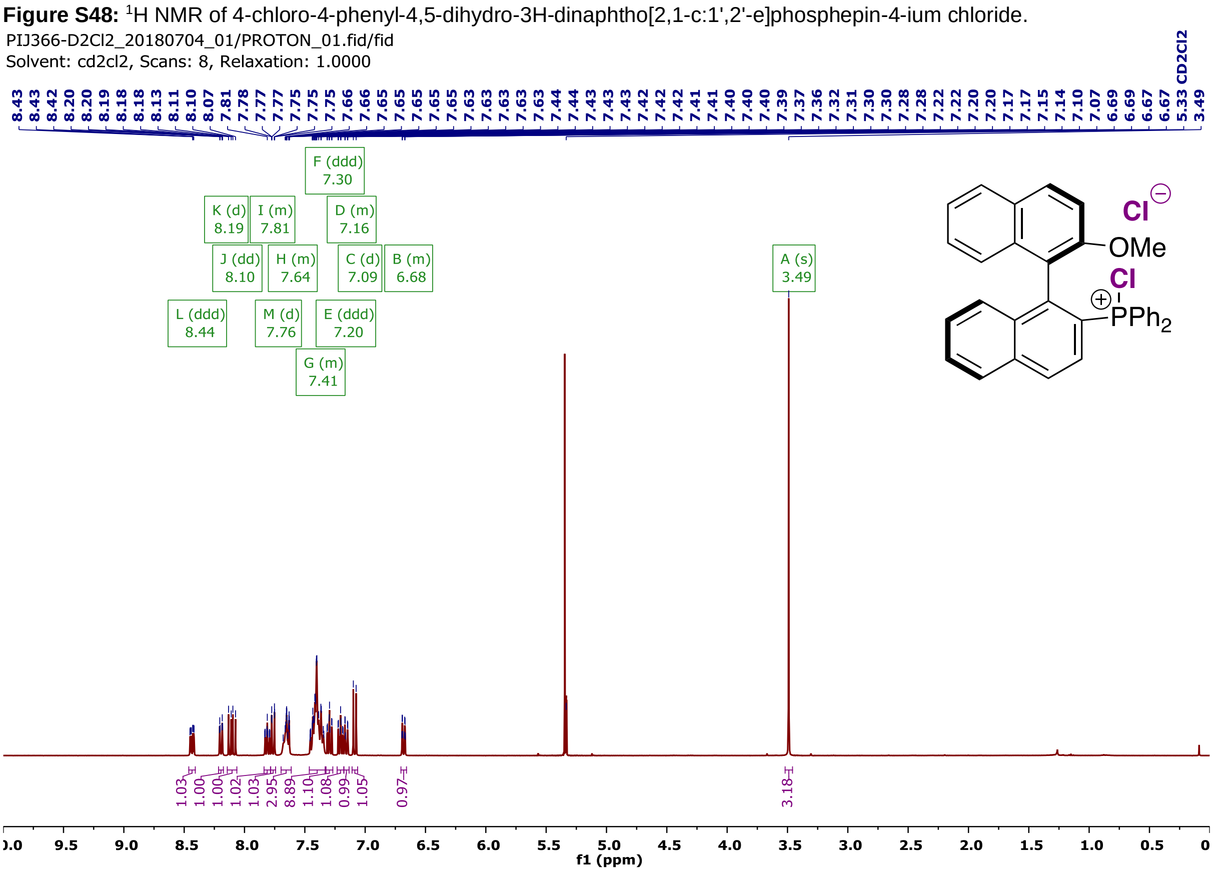

Figure S49: ^{31}P NMR of 4-chloro-4-phenyl-4,5-dihydro-3H-dinaphtho[2,1-c:1',2'-e]phosphepin-4-ium chloride.

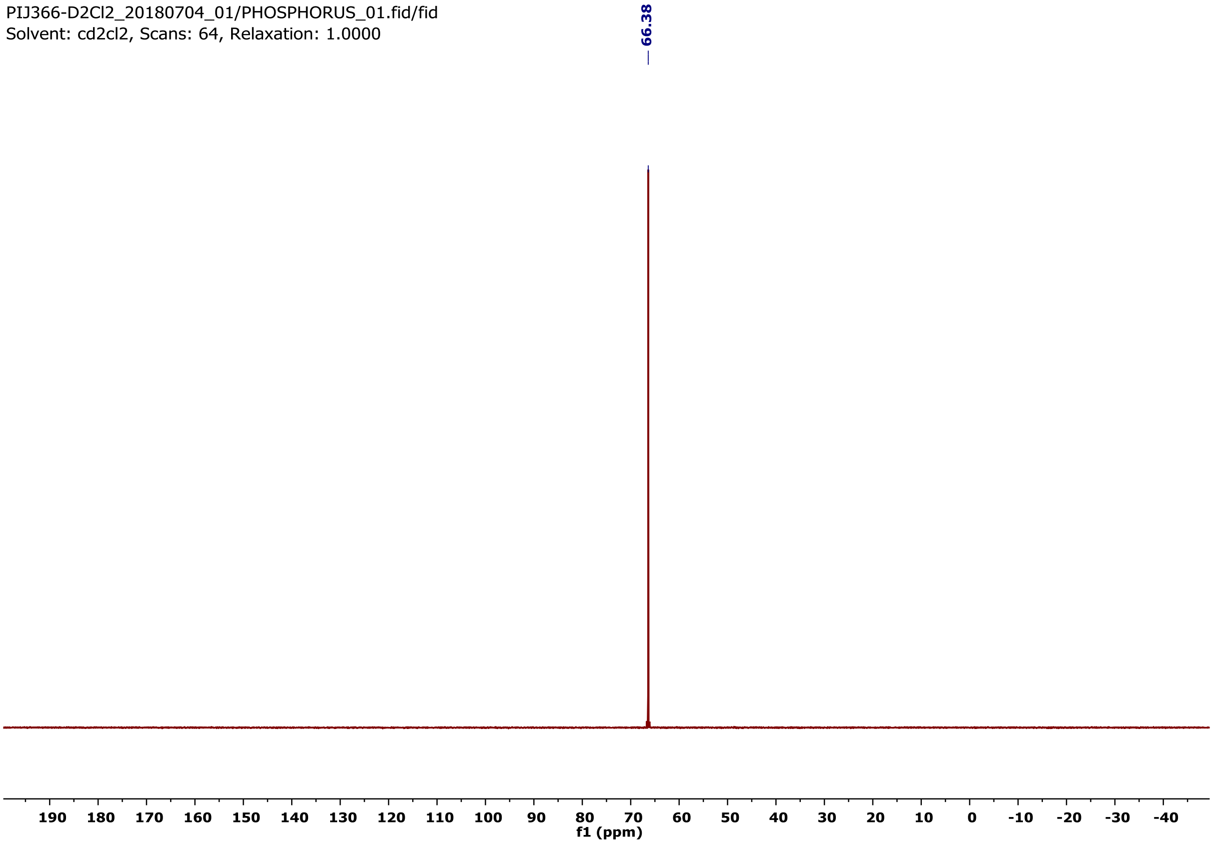

Figure S50: ^{1}H NMR of (*R*)-(+)-2-(Diphenylphosphino)-2′-methoxy-1,1′-binaphthyl, **13**.[8]

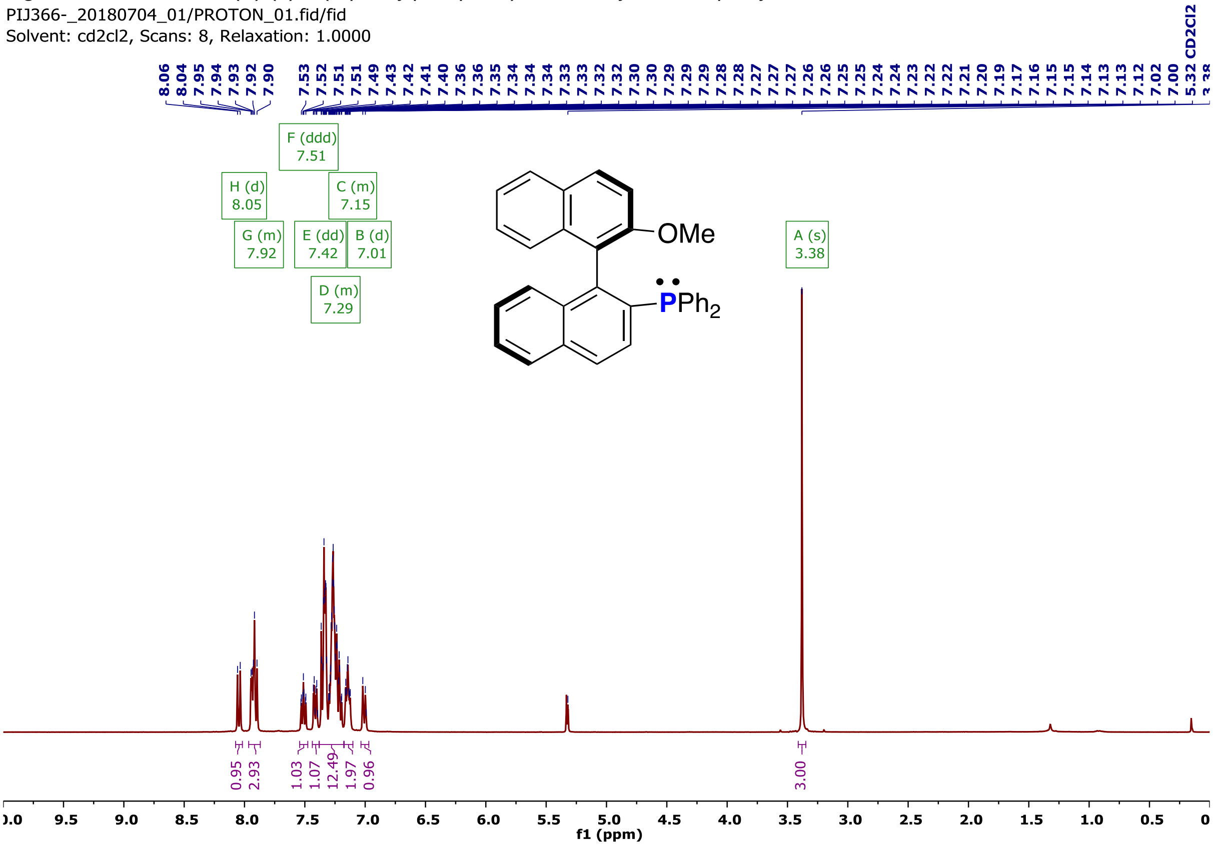

Figure S51: ^{31}P NMR of (*R*)-(+)-2-(Diphenylphosphino)-2′-methoxy-1,1′-binaphthyl, **13**.[8]

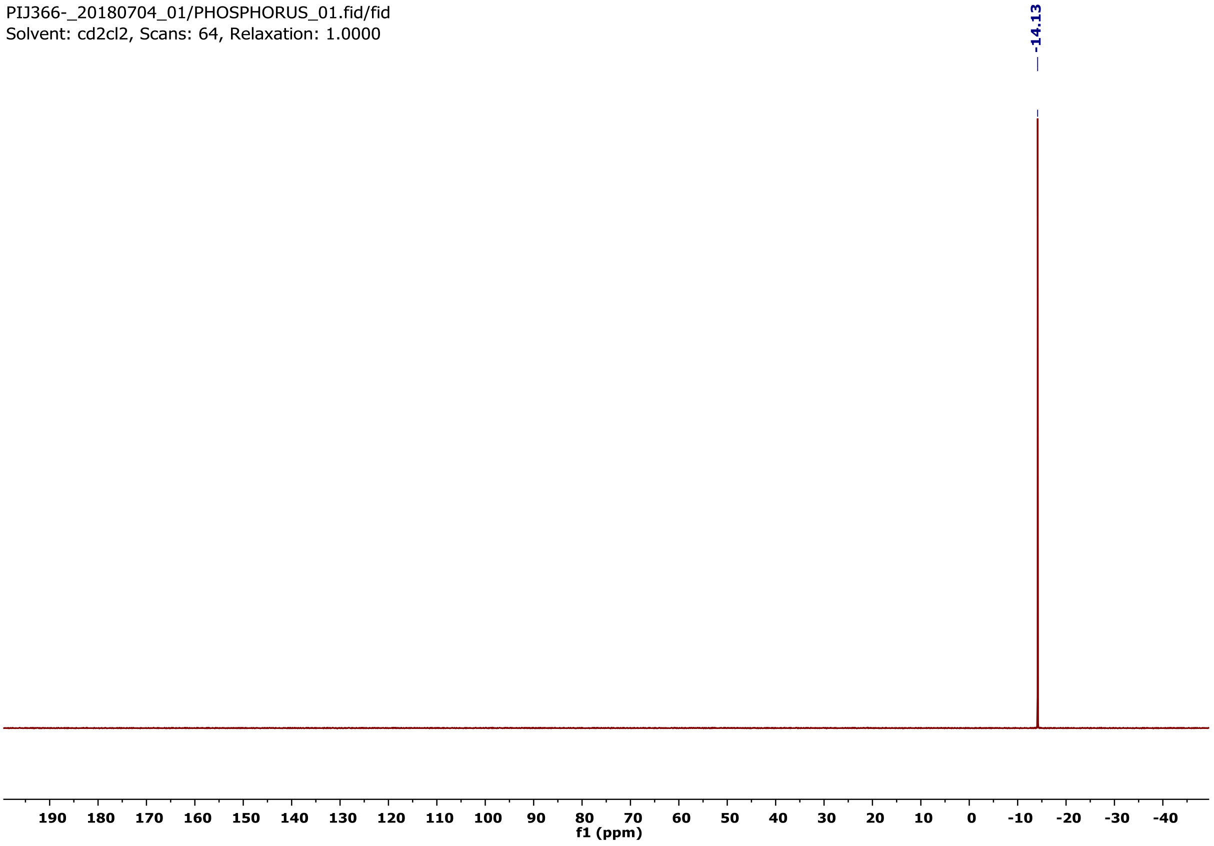

Figure S52: ^{1}H NMR of 3-((di-*tert*-butylphosphoryl)methyl)-1-mesityl-1*H*-imidazol-3-ium 4-methylbenzenesulfo-nate, **4**:[9]

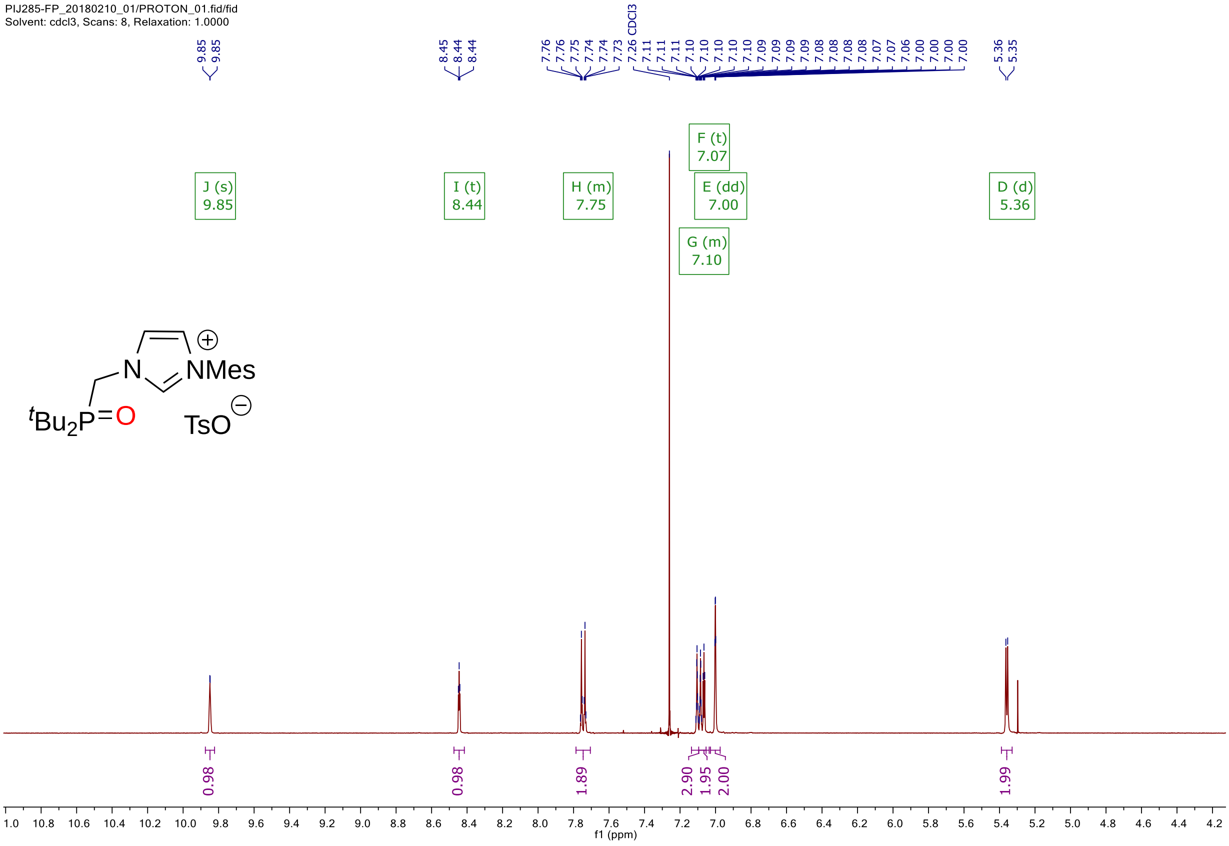

Figure S53: ^{13}C NMR of 3-((di-*tert*-butylphosphoryl)methyl)-1-mesityl-1*H*-imidazol-3-ium 4-methylbenzenesulfo-nate, **4**.[9]

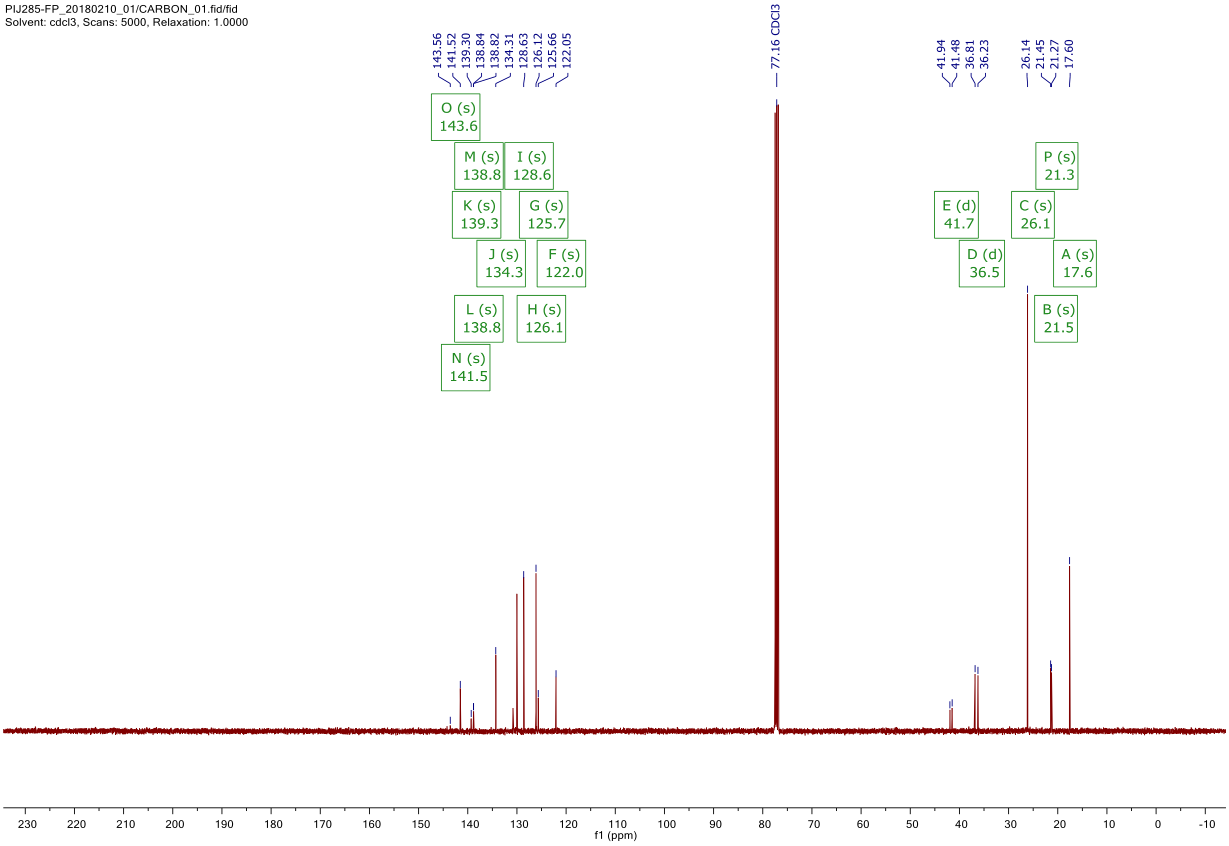

Figure S54: ^{31}P NMR of 3-((di-*tert*-butylphosphoryl)methyl)-1-mesityl-1*H*-imidazol-3-ium 4-methylbenzenesulfo-nate, **4**.[9]

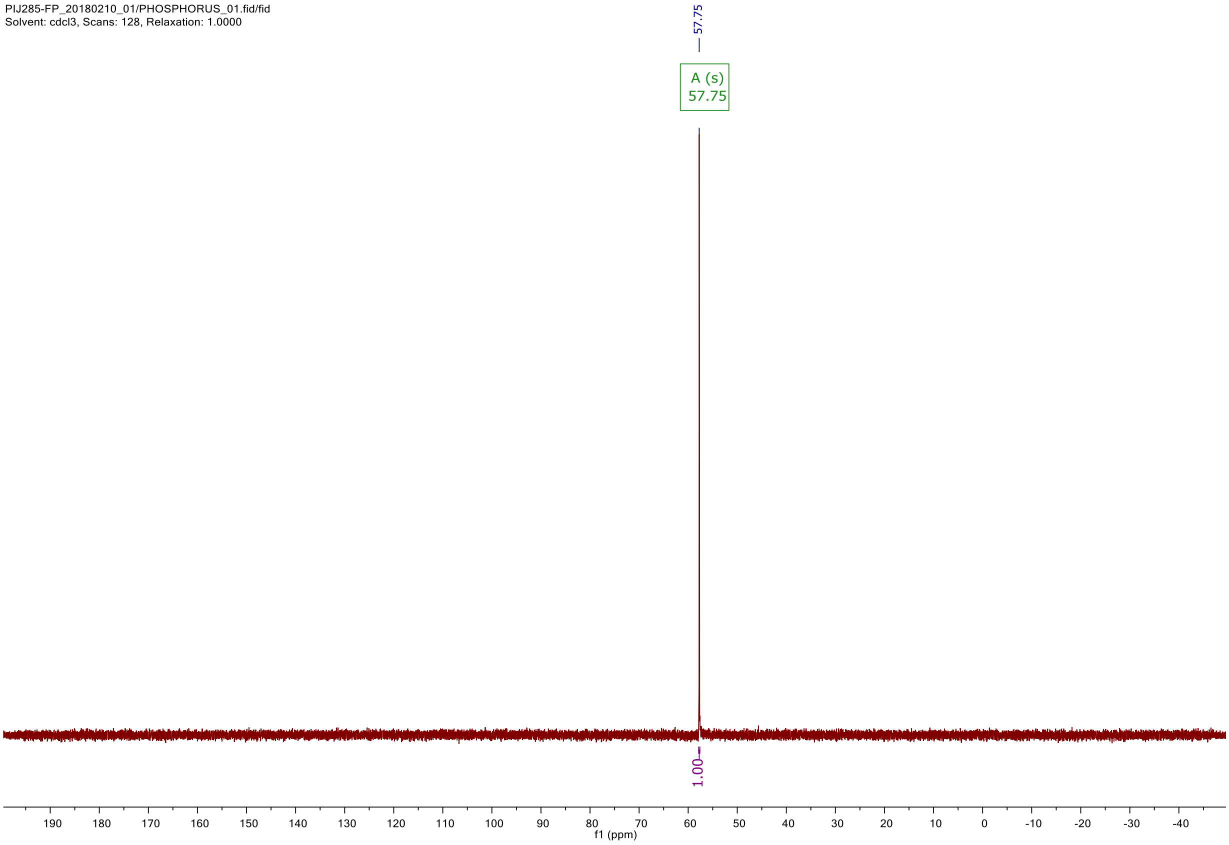

Figure S55: ^{1}H NMR of 3-((di-*tert*-butyldichloro- λ^4-phosphaneyl)methyl)-1-mesityl-1*H*-imidazol-3-ium chloride.

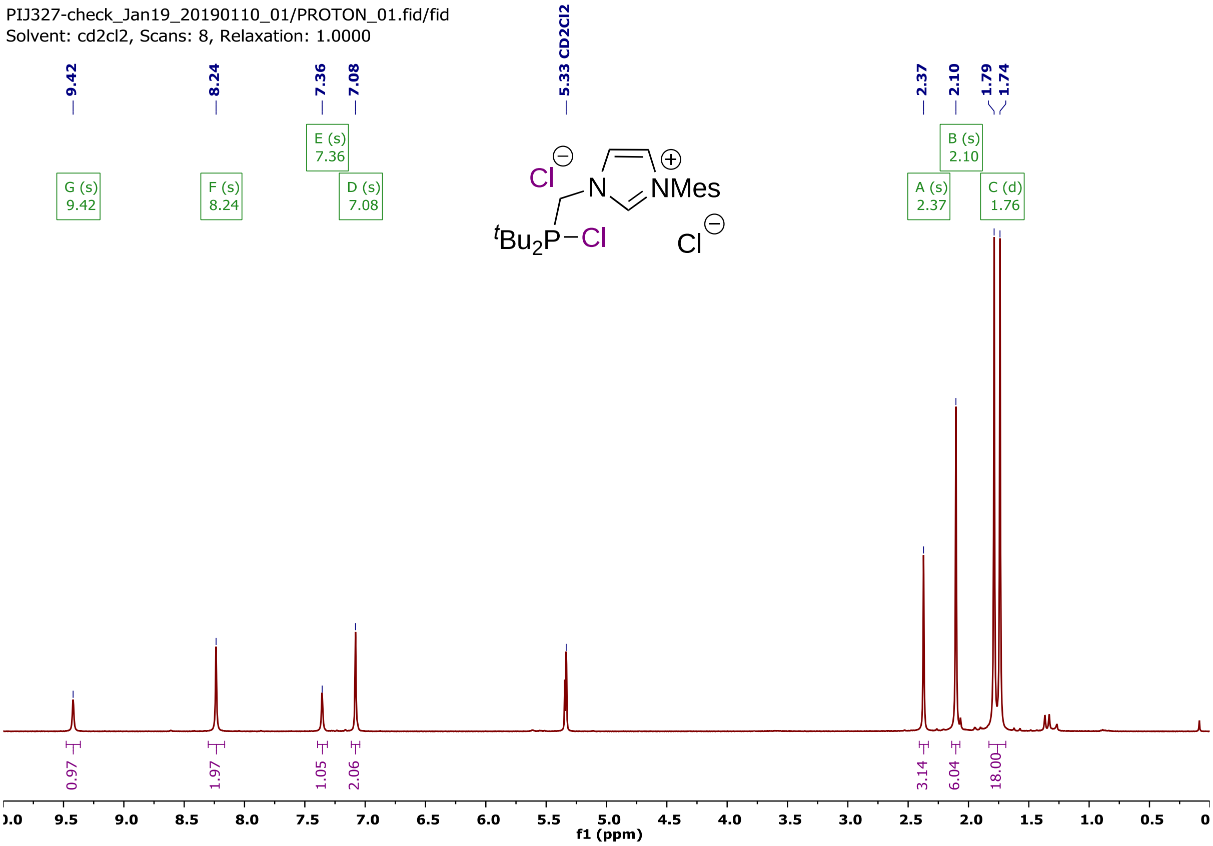

Figure S56: ^{31}P NMR of 3-((di-*tert*-butyldichloro- λ^4-phosphaneyl)methyl)-1-mesityl-1*H*-imidazol-3-ium chloride.

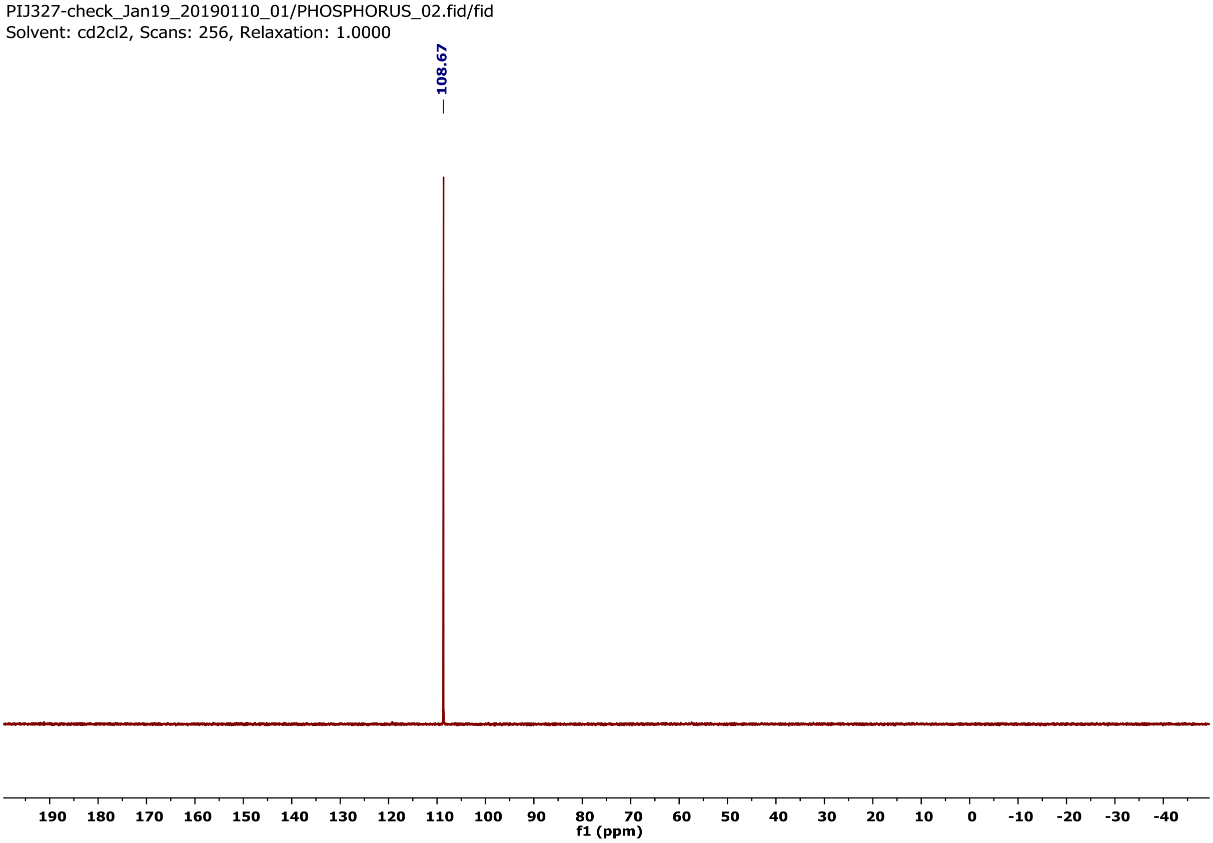

Figure S57: ^{13}C NMR of 3-((di-*tert*-butyldichloro- λ^4-phosphaneyl)methyl)-1-mesityl-1*H*-imidazol-3-ium chloride.

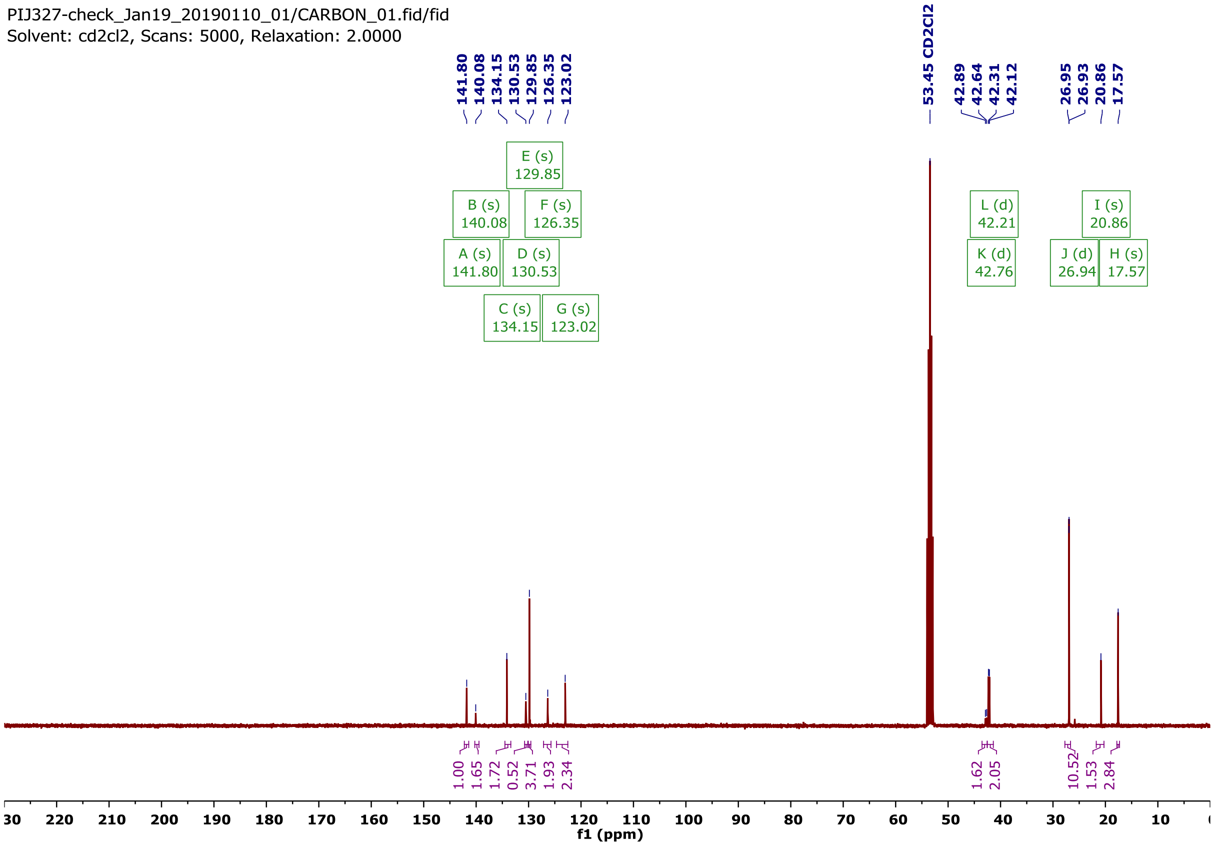

Figure S58: ^{1}H NMR of 3-((di-*tert*-butylphosphaneyl)methyl)-1-mesityl-1*H*-imidazol-3-ium chloride, **5**.[9]

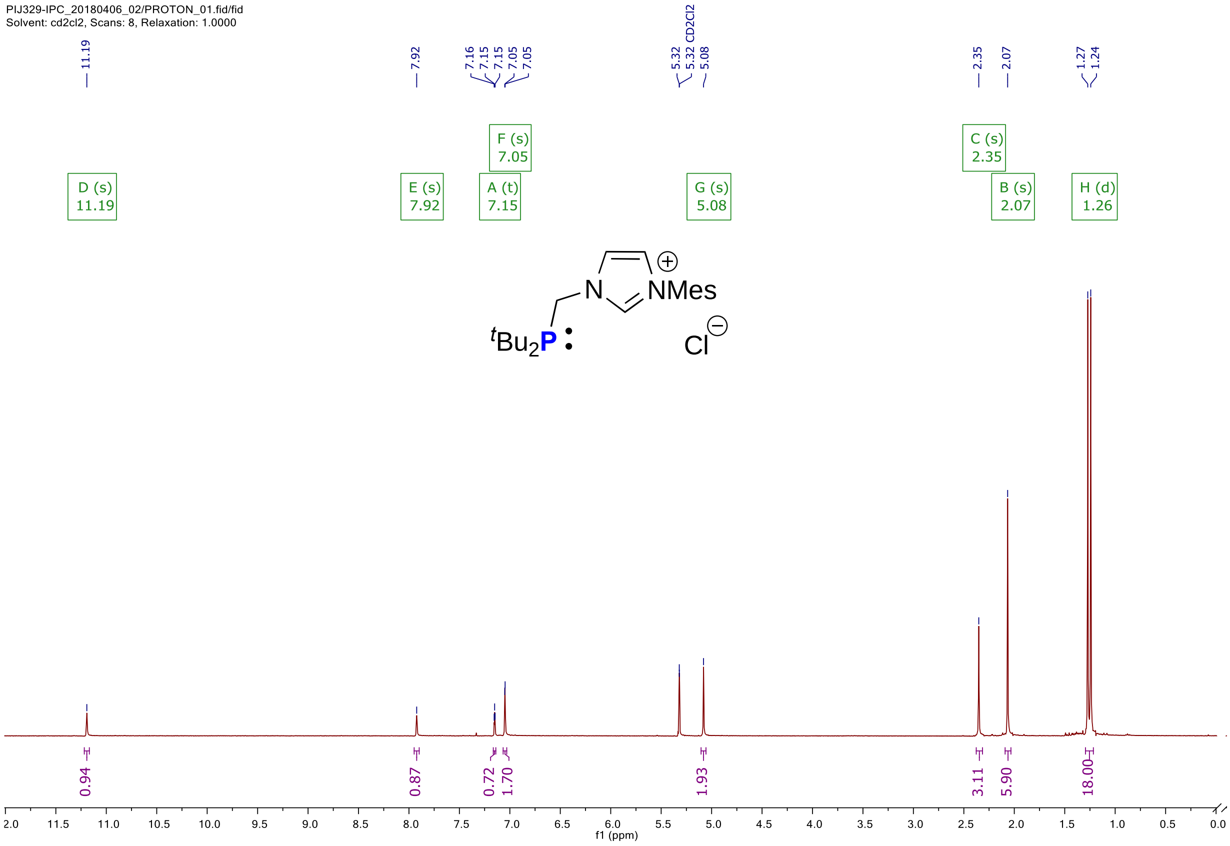

Figure S59: ^{31}P NMR of 3-((di-*tert*-butylphosphaneyl)methyl)-1-mesityl-1*H*-imidazol-3-ium chloride, **5**.[9]

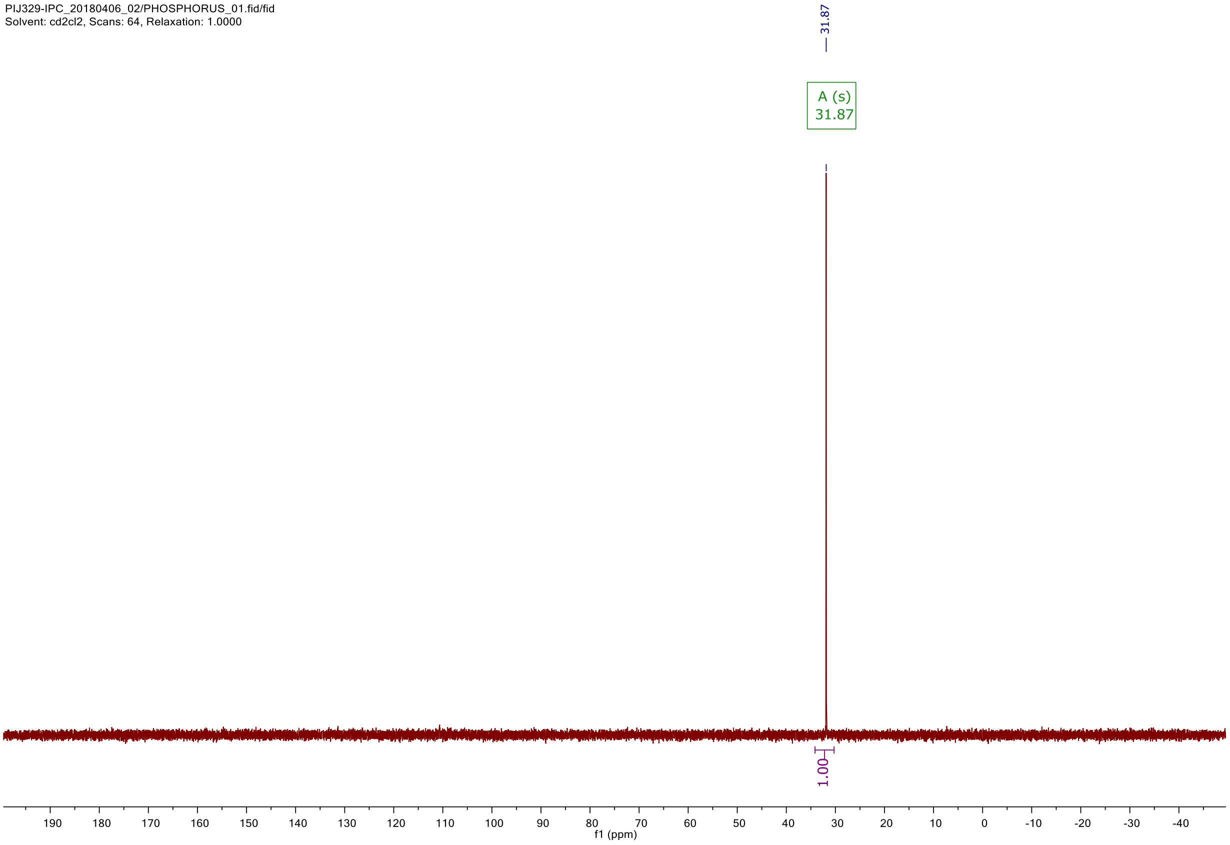

Figure S60: ^{1}H NMR of 1-((di-*tert*-butylphosphoryl)methyl)-3-(2,6-diisopropylphenyl)-1*H*-imidazol-3-ium 4-methylbenzenesulfonate.

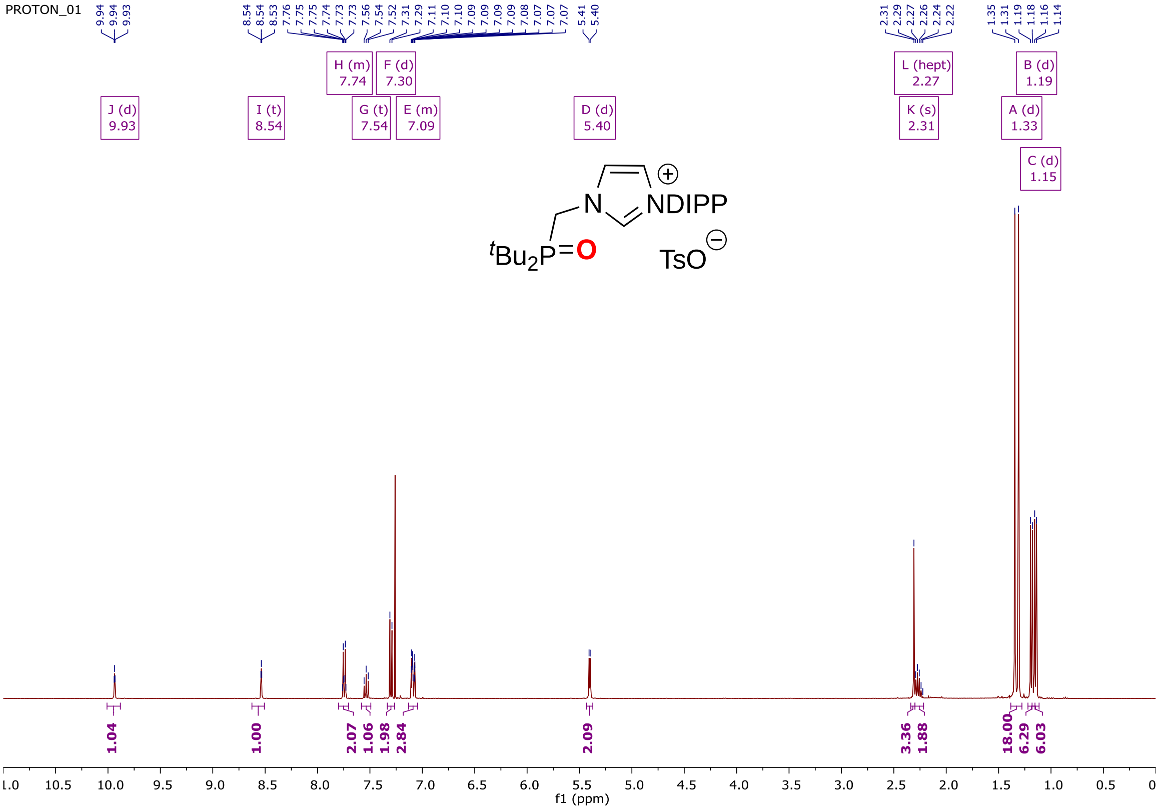

Figure S61: ^{31}P NMR of 1-((di-*tert*-butylphosphoryl)methyl)-3-(2,6-diisopropylphenyl)-1*H*-imidazol-3-ium 4-methylbenzenesulfonate.

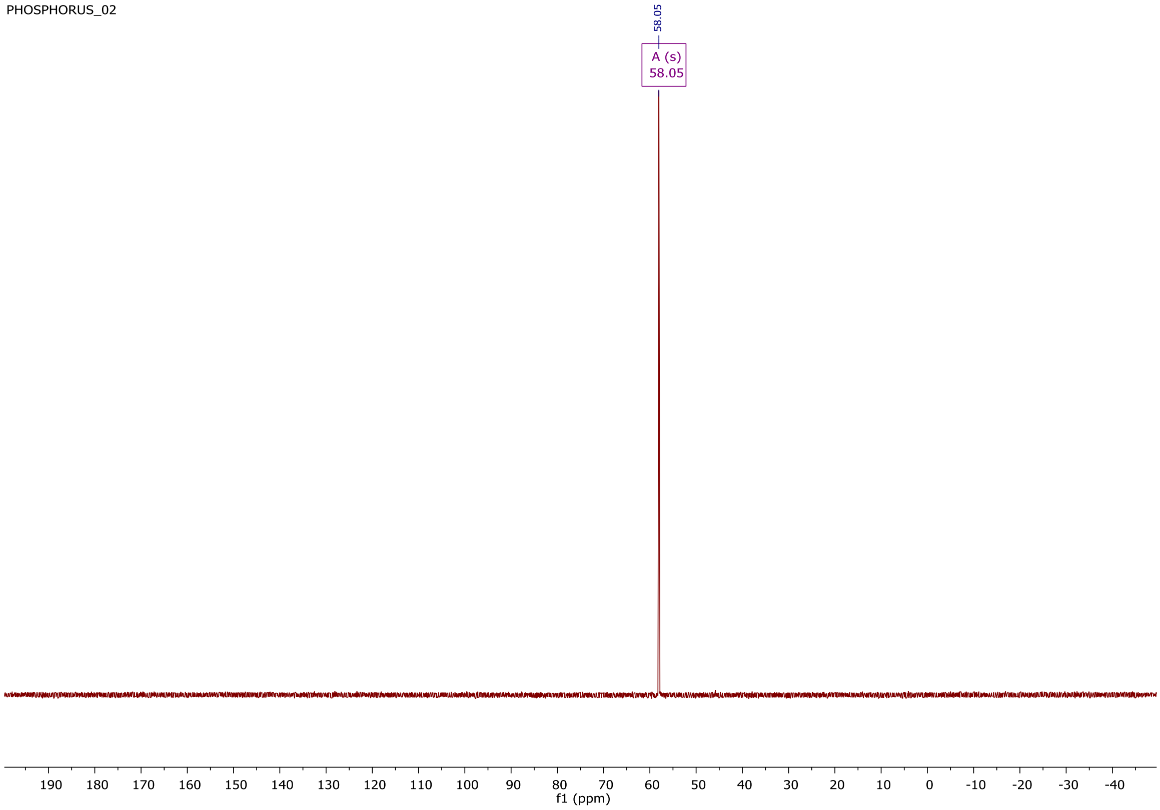

Figure S62: ^{13}C NMR of 1-((di-*tert*-butylphosphoryl)methyl)-3-(2,6-diisopropylphenyl)-1*H*-imidazol-3-ium 4-methylbenzenesulfonate.

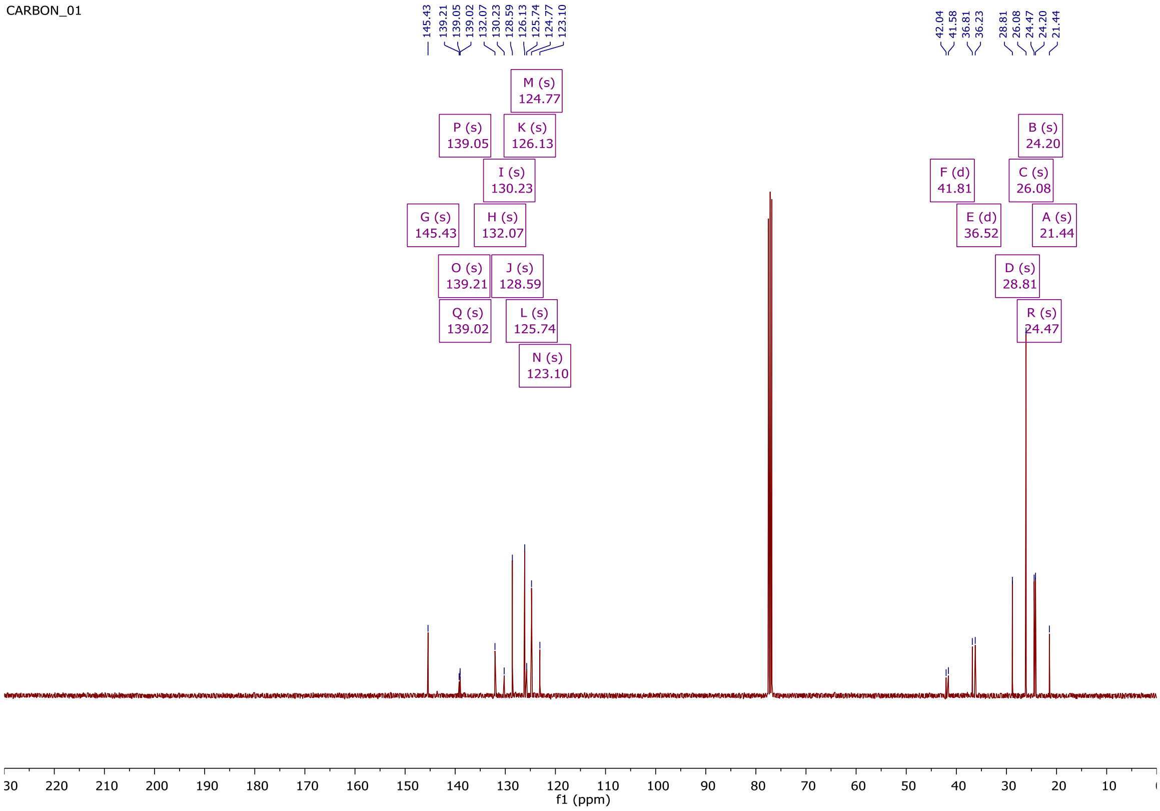

Figure S63: ^{1}H NMR of mono(1-((di-*tert*-butylchloro-λ^{4}-phosphaneyl)methyl)-3-(2,6-diisopropylphenyl)-1*H*-imidazol-3-ium) dichloride.

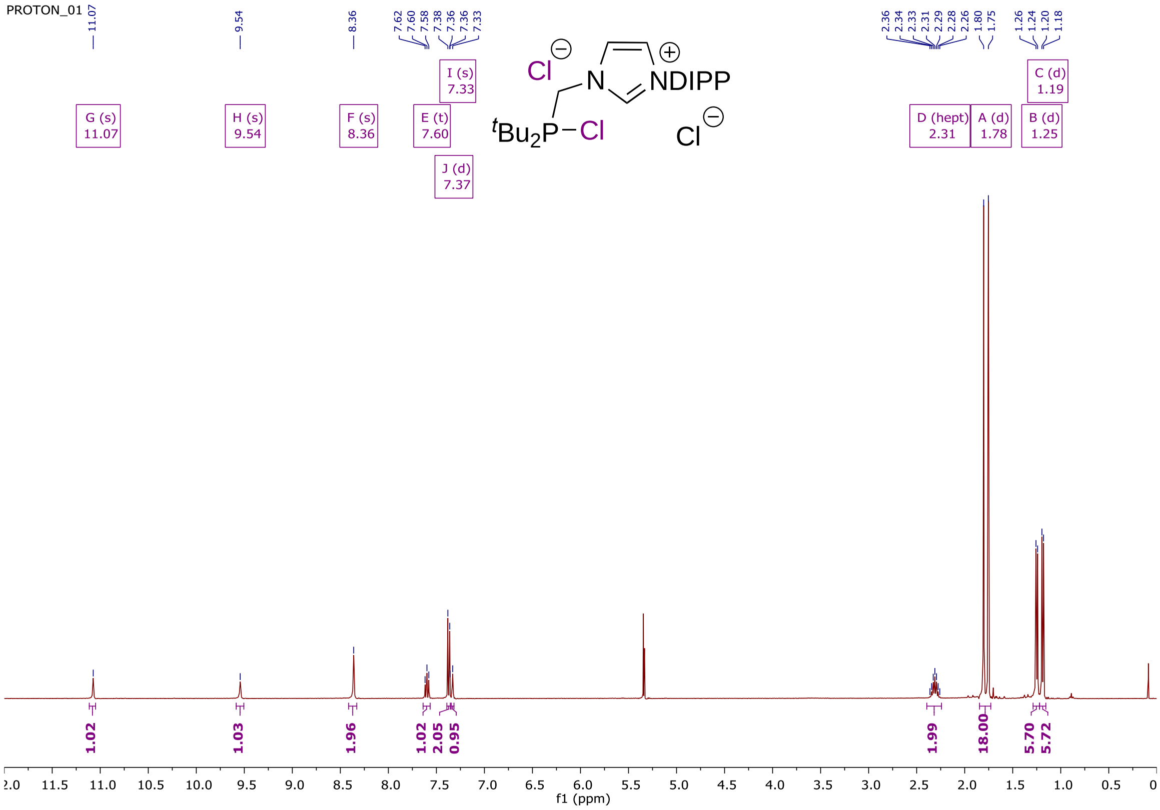

Figure S64: ^{31}P NMR of mono(1-((di-*tert*-butylchloro-λ^{4}-phosphaneyl)methyl)-3-(2,6-diisopropylphenyl)-1*H*-imidazol-3-ium) dichloride.

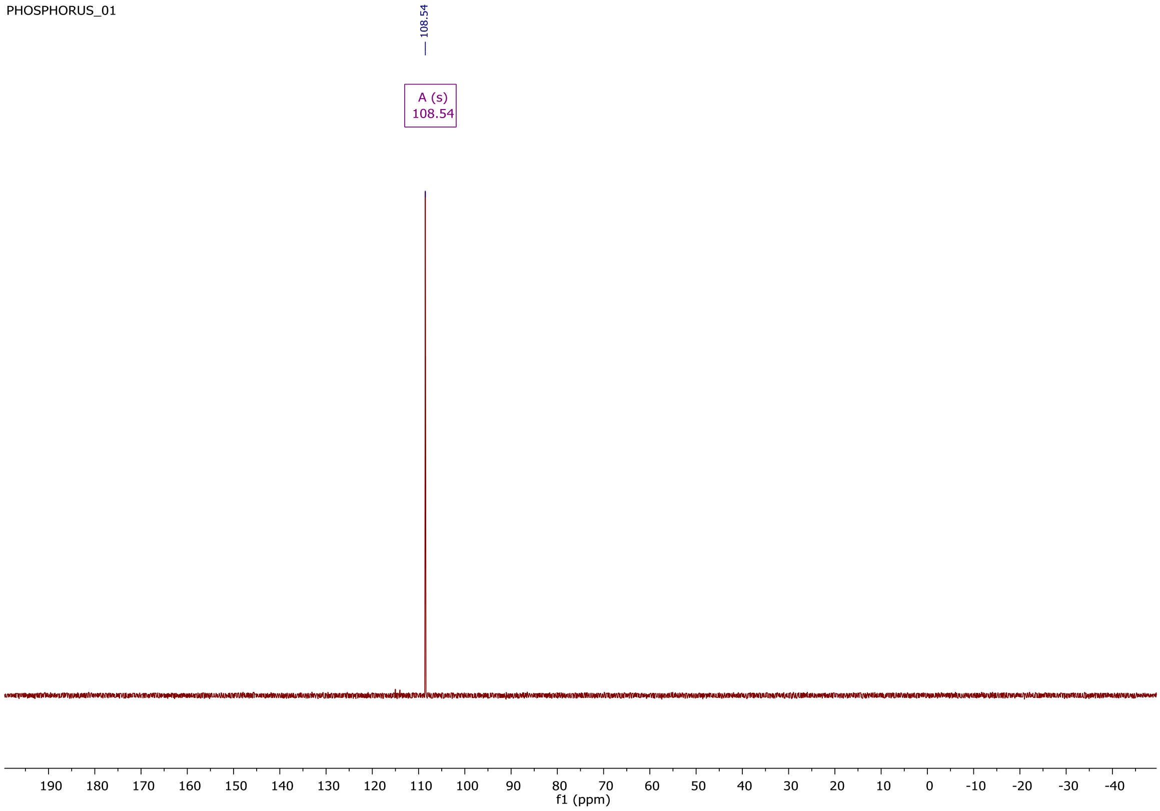

Figure S65: ^{13}C NMR of mono(1-((di-*tert*-butylchloro-λ^4-phosphaneyl)methyl)-3-(2,6-diisopropylphenyl)-1*H*-imidazol-3-ium) dichloride.

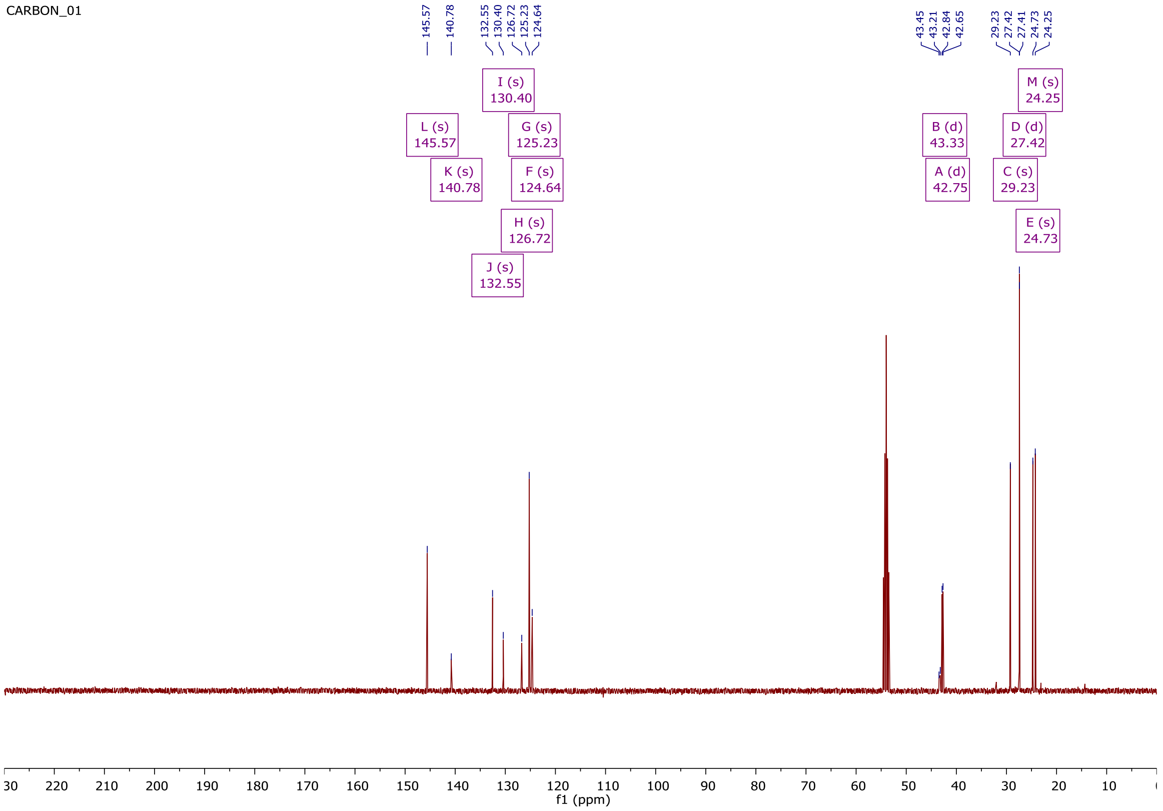

Figure S66: ^{1}H NMR of 1-((di-*tert*-butylphosphaneyl)methyl)-3-(2,6-diisopropylphenyl)-1*H*-imidazol-3-ium chloride, **14**.

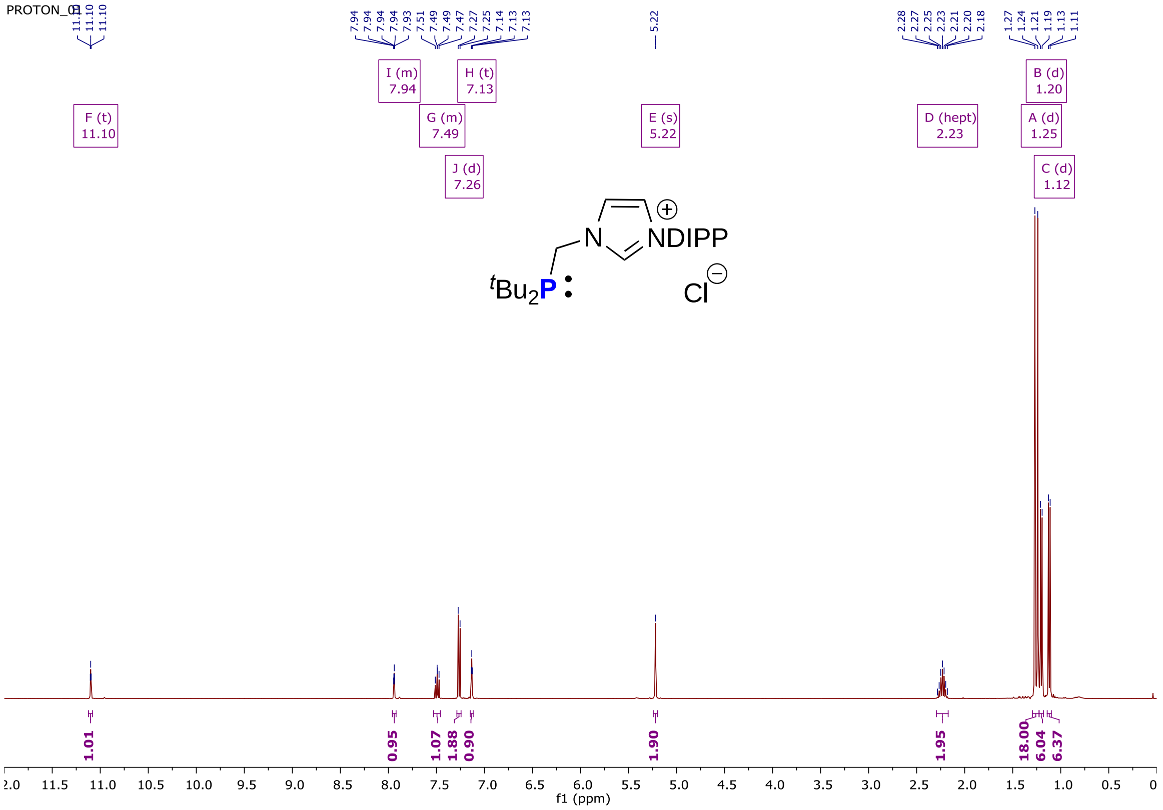

Figure S67: ^{31}P NMR of 1-((di-*tert*-butylphosphaneyl)methyl)-3-(2,6-diisopropylphenyl)-1*H*-imidazol-3-ium chloride, **14**.

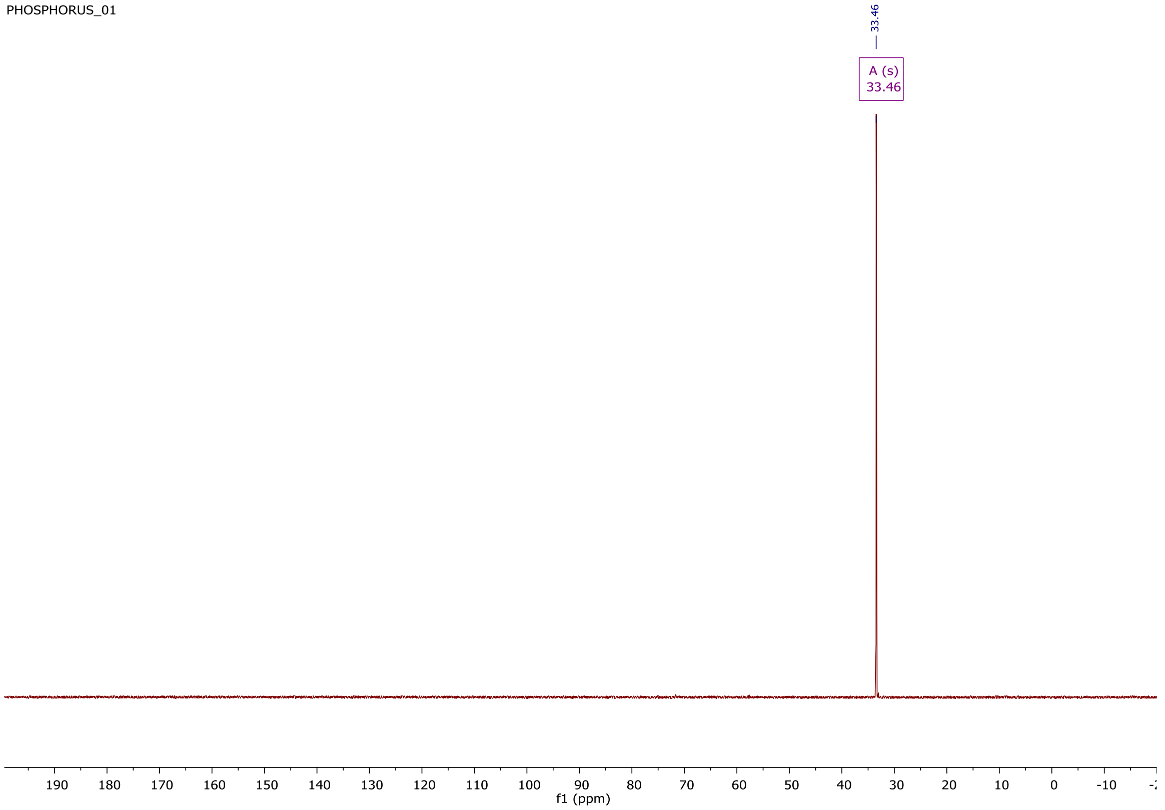

Figure S68: ^{13}C NMR of 1-((di-*tert*-butylphosphaneyl)methyl)-3-(2,6-diisopropylphenyl)-1*H*-imidazol-3-ium chloride, **14**.

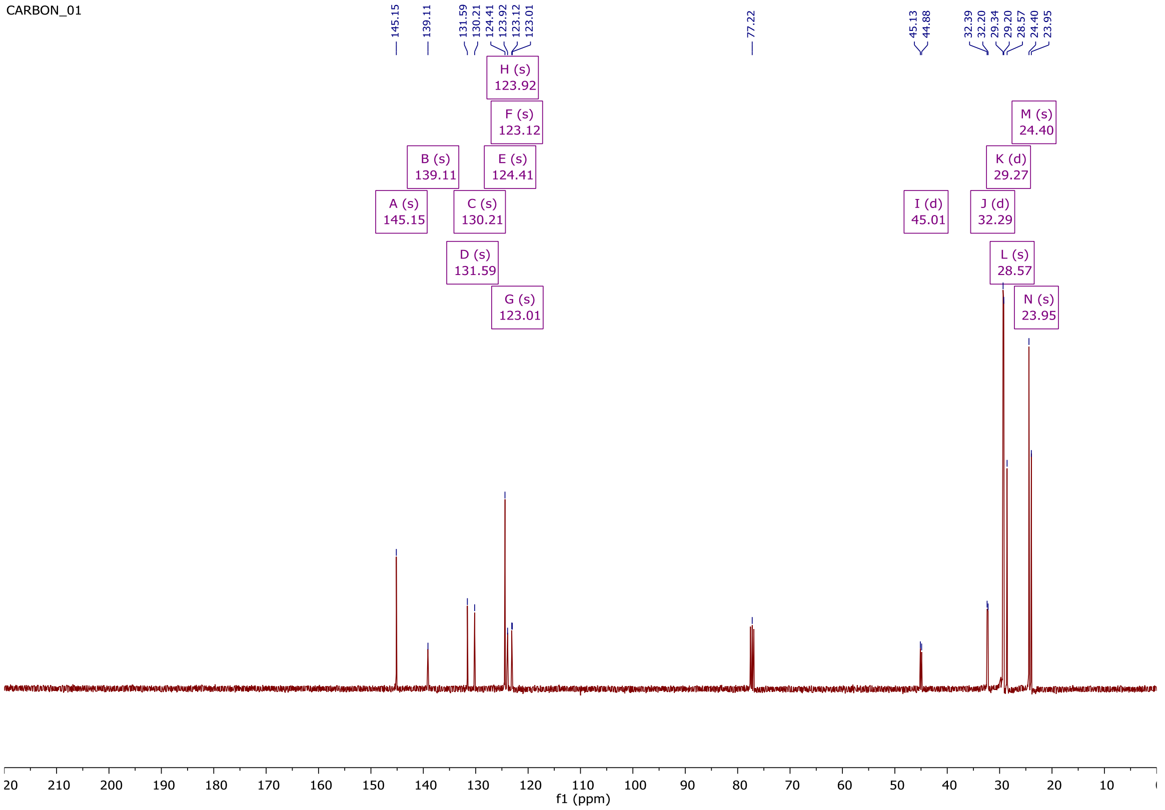

Figure S69: ^{1}H NMR of Dichloro(η^{6}-*p*-cymene)(triphenylphosphine)ruthenium(II), **18**.[10]

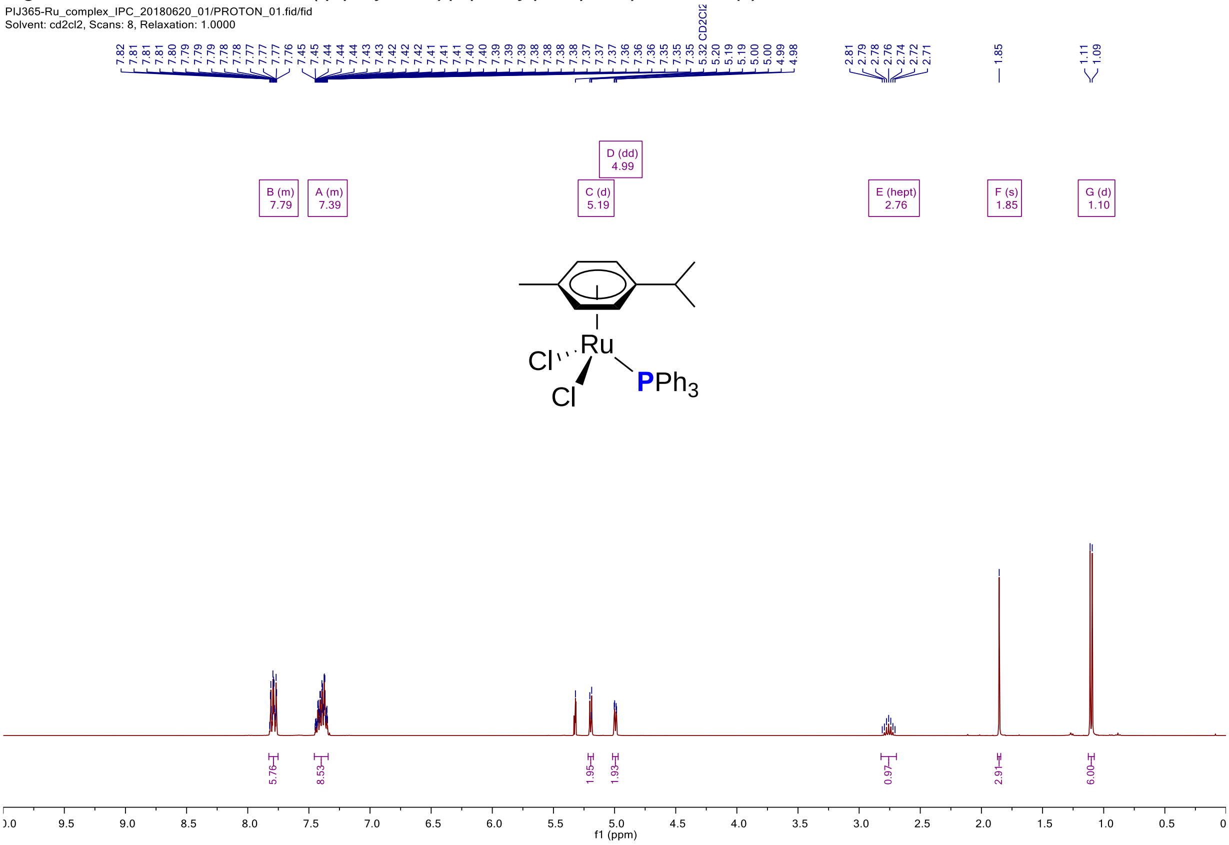

Figure, S70: ^{31}P NMR of Dichloro(η^{6}-*p*-cymene)(triphenylphosphine)ruthenium(II), **18**.[10]

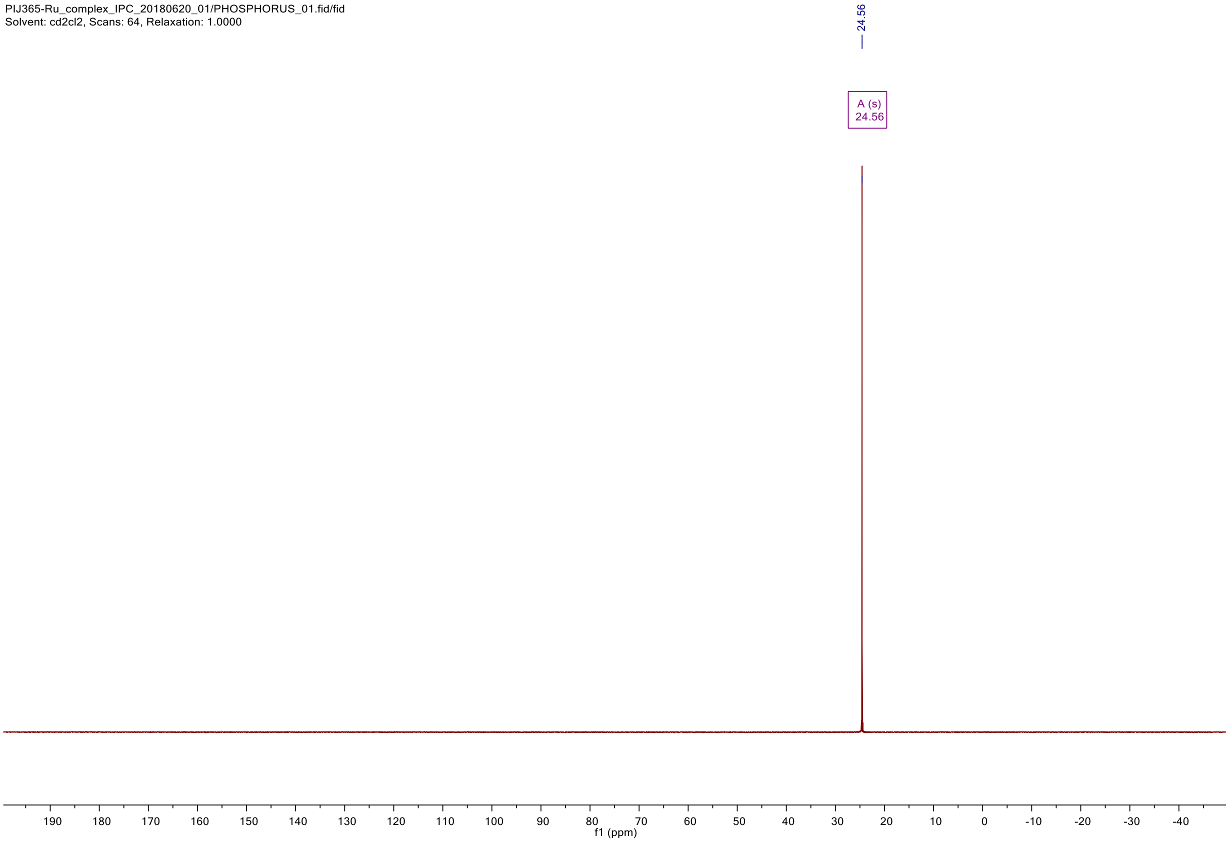

Figure S71: ^{1}H NMR of Umicore Grubbs Catalyst M2 - [1,3-Bis(2,4,6-trimethylphenyl)-2-imidazolidinylidene] dichloro(3-phenyl-1*H*-inden-1-ylidene)(tricyclohexylphosphine)ruthenium(II), **19**.[11]

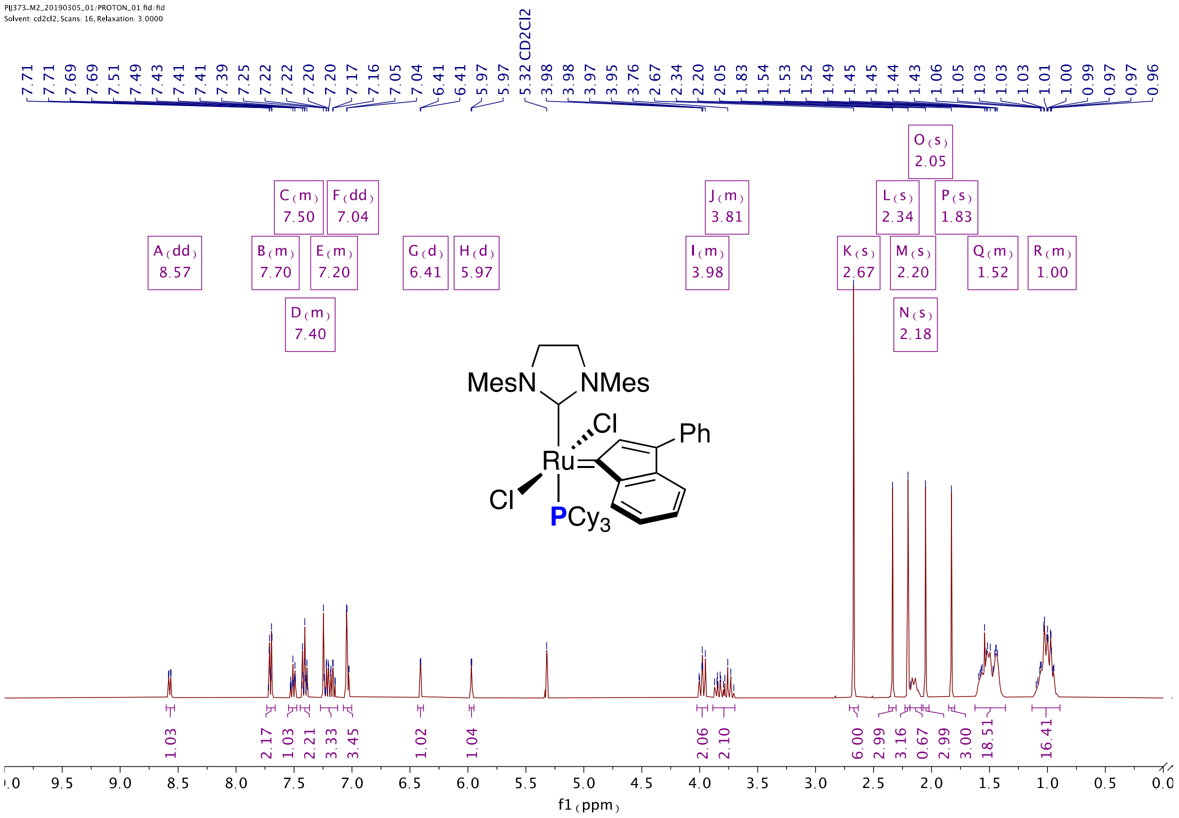

Figure, S72: ^{31}P NMR of Umicore Grubbs Catalyst M2 - [1,3-Bis(2,4,6-trimethylphenyl)-2-imidazolidinylidene] dichloro(3-phenyl-1*H*-inden-1-ylidene)(tricyclohexylphosphine)ruthenium(II), **19**.[11]

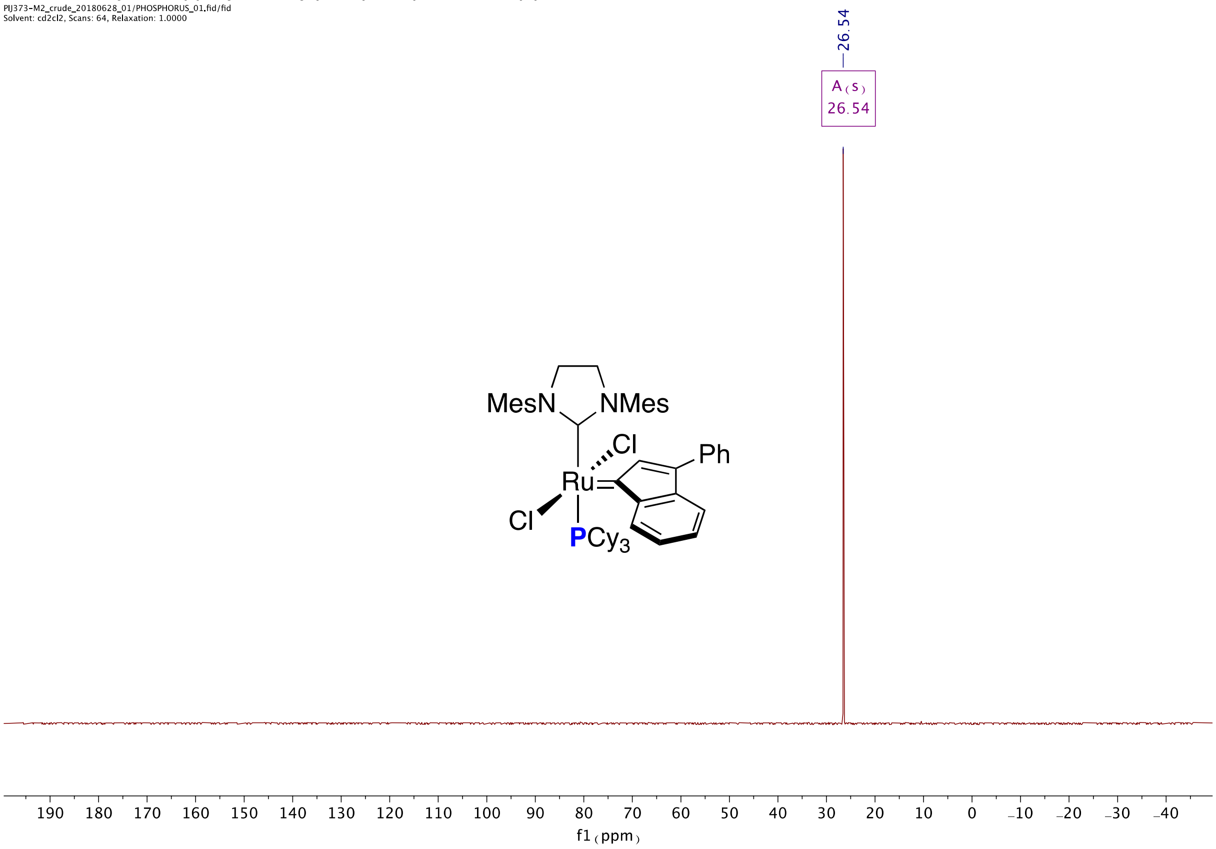

Figure S73: ^{1}H NMR of Pd(allyl)CyJohnPhos, **20**.[12]

PIJ410-recrys_20190210_01/PROTON_01.fid/fid

Solvent: cd2cl2, Scans: 8, Relaxation: 1.0000

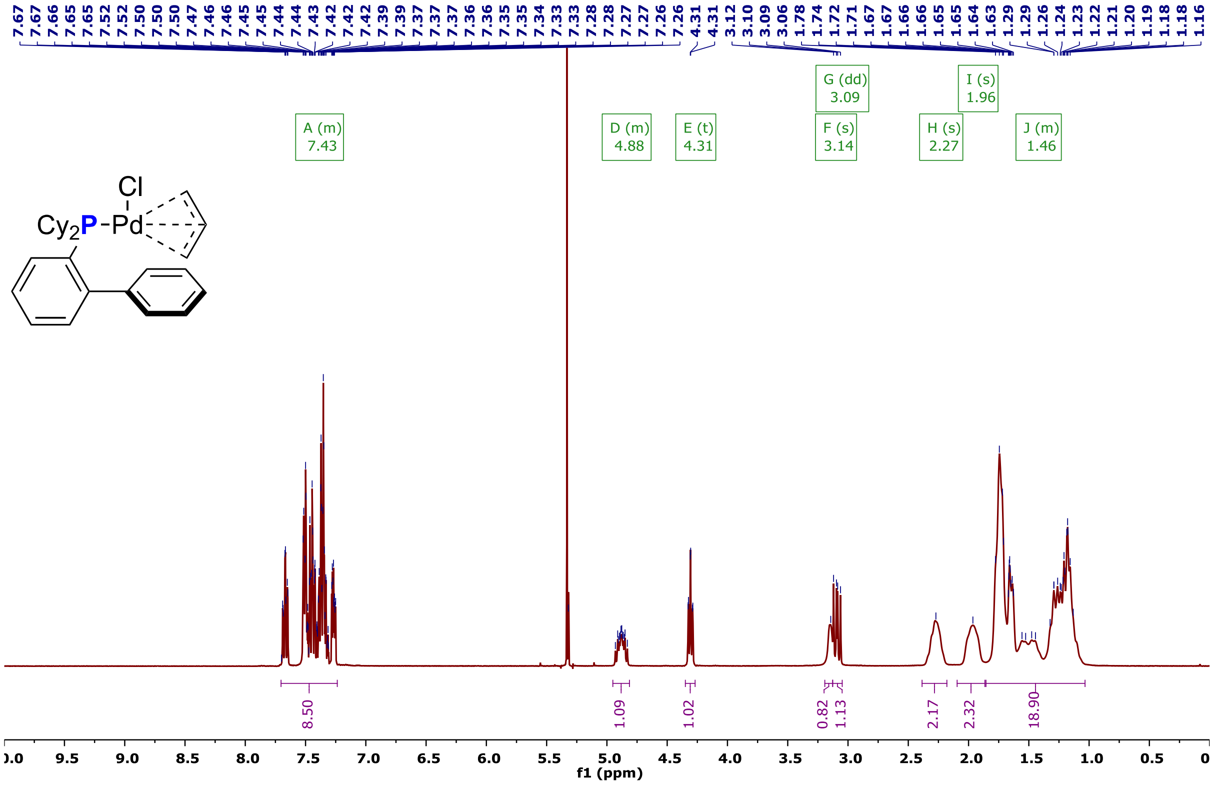

Figure, S74: ^{13}P NMR of Pd(allyl)CyJohnPhos, **20**.[12]

PIJ410-recrys_20190210_01/PHOSPHORUS_02.fid/fid

Solvent: cd2cl2, Scans: 256, Relaxation: 1.0000

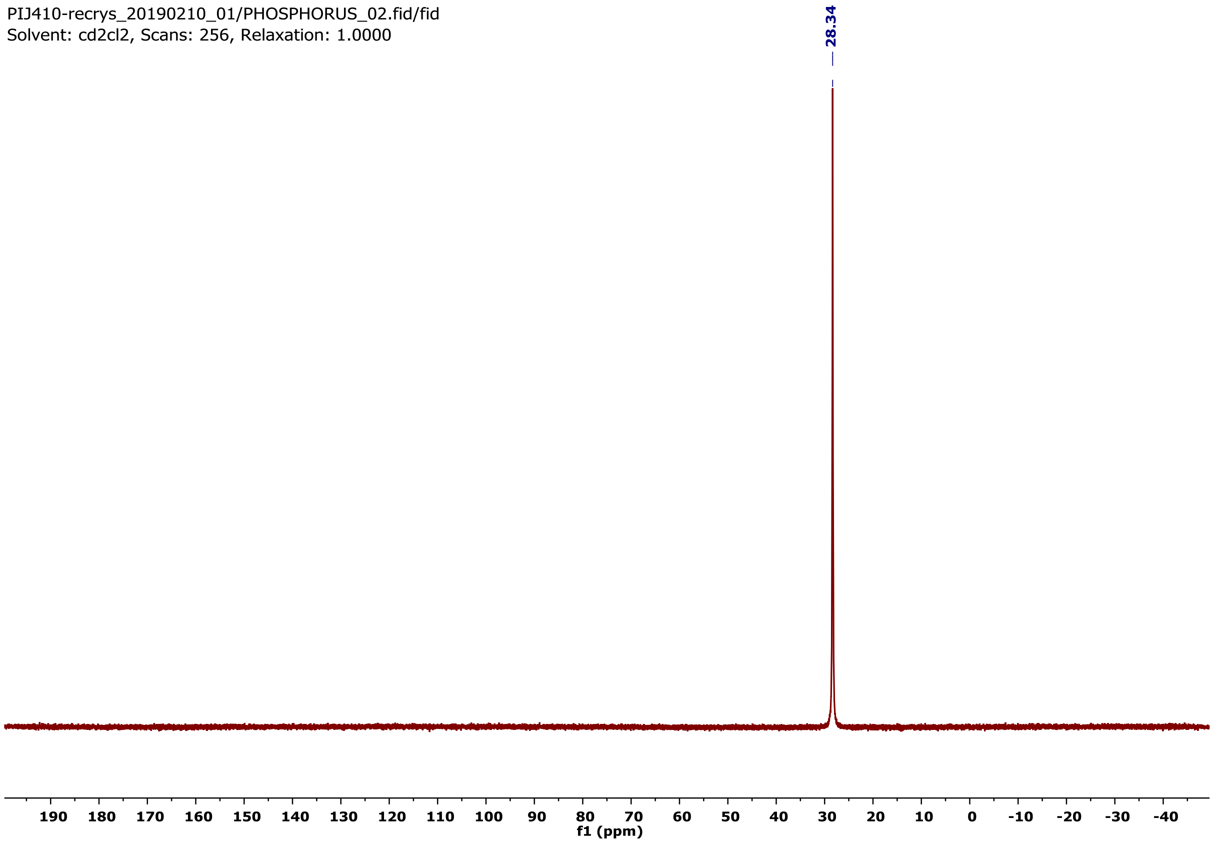

Figure S75: ^{1}H NMR of Ni(DPPE)Cl_2, **21**.[13]

PIJ418-Ni_retake_20190208_01/PROTON_02.fid/fid

Solvent: cd2cl2, Scans: 8, Relaxation: 1.0000

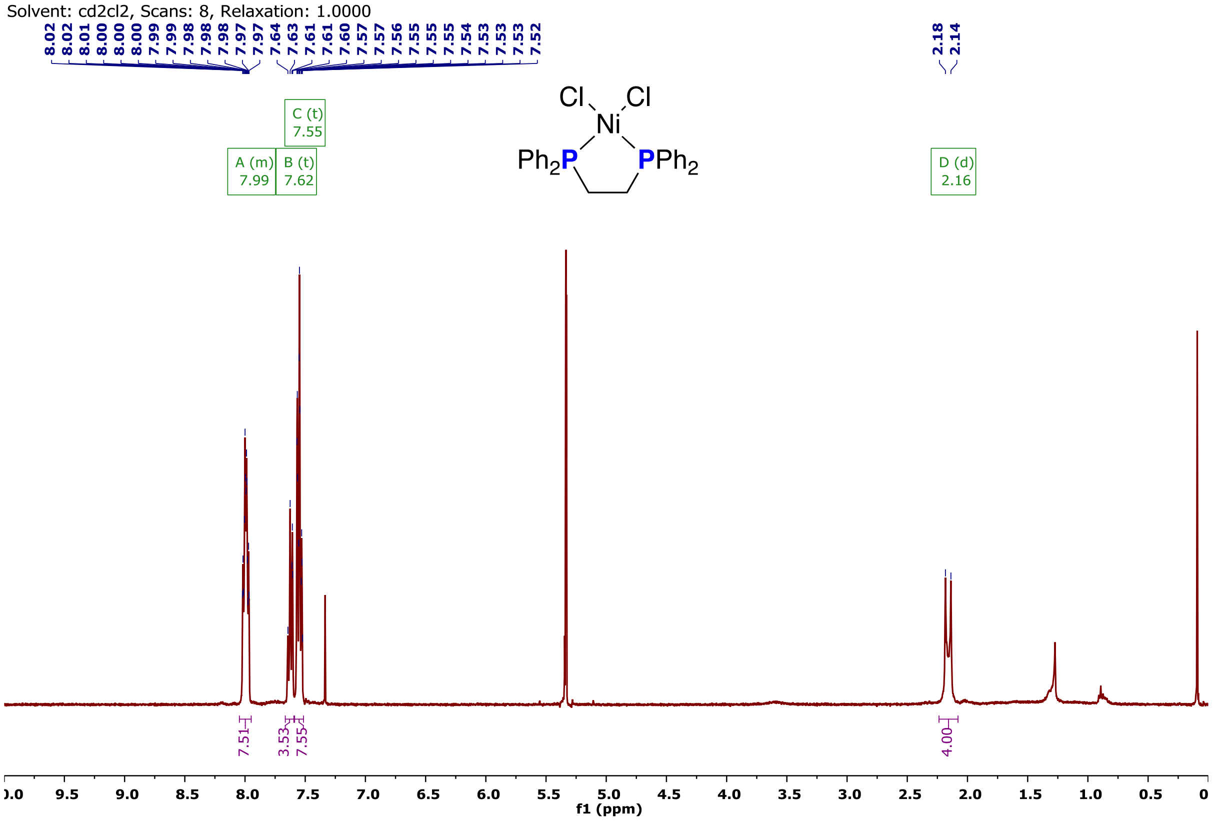

Figure S75: ^{13}P NMR of Ni(DPPE)Cl_2, **21**.[13]

PIJ418-Ni_20190116_01/PHOSPHORUS_02.fid/fid

Solvent: cd2cl2, Scans: 256, Relaxation: 1.0000

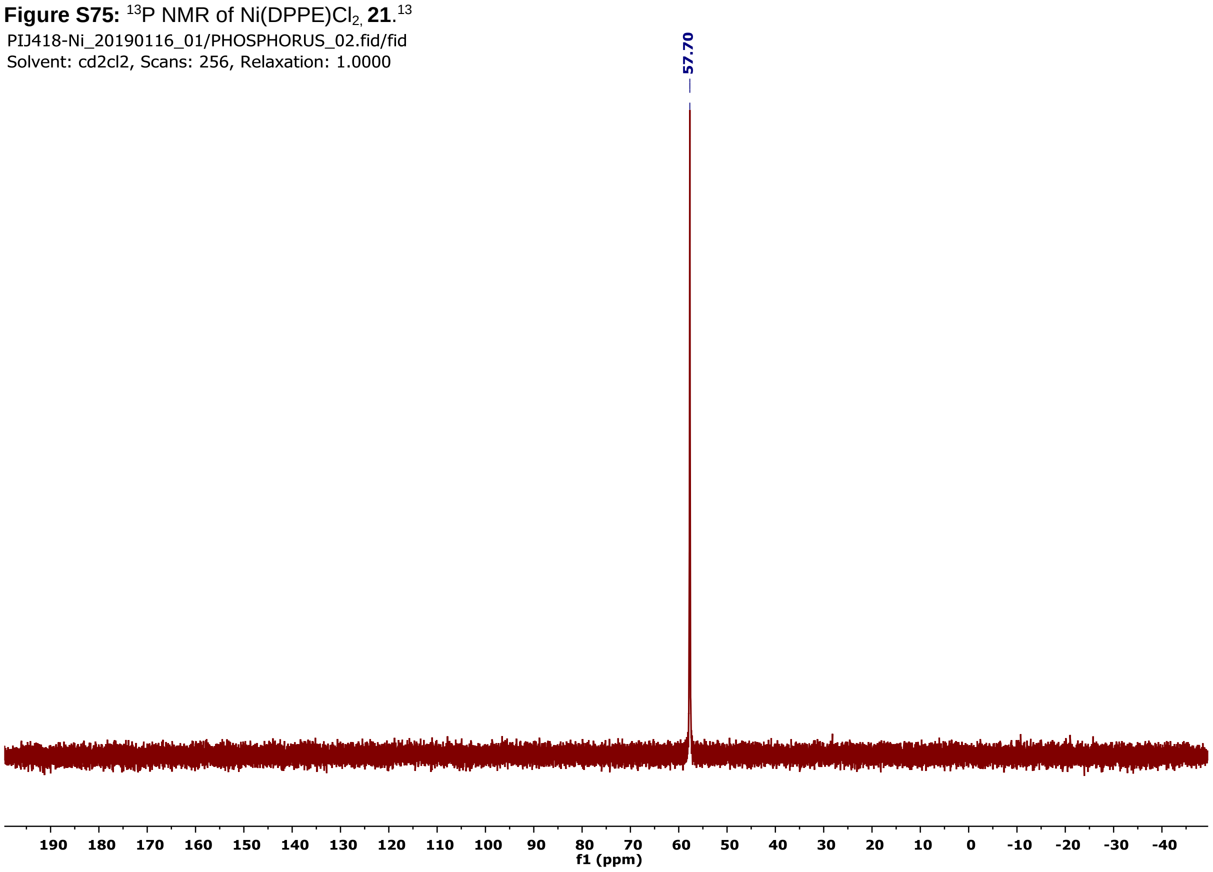

Figure S76: ^{1}H NMR of ([Pd(P4)Cl]Cl), **22**.[14]

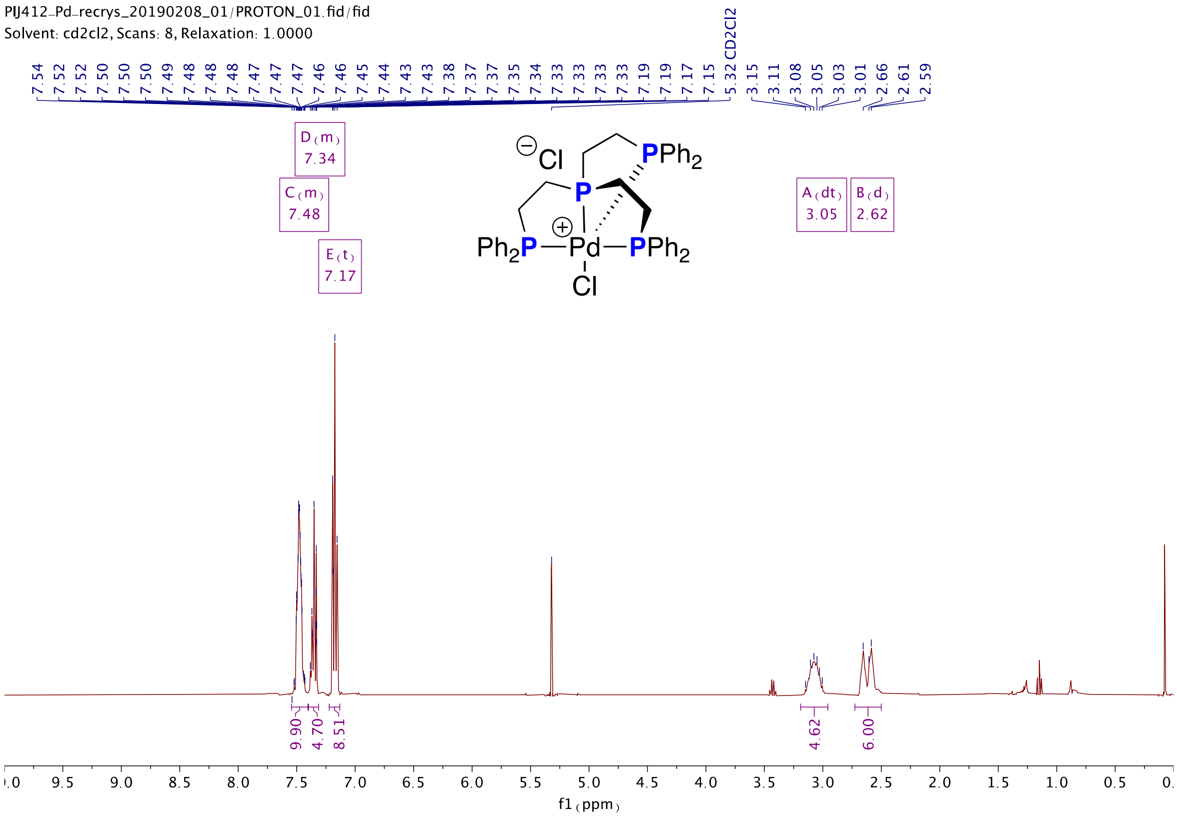

Figure S76: ^{1}H NMR of ([Pd(P4)Cl]Cl), **22**.[14]

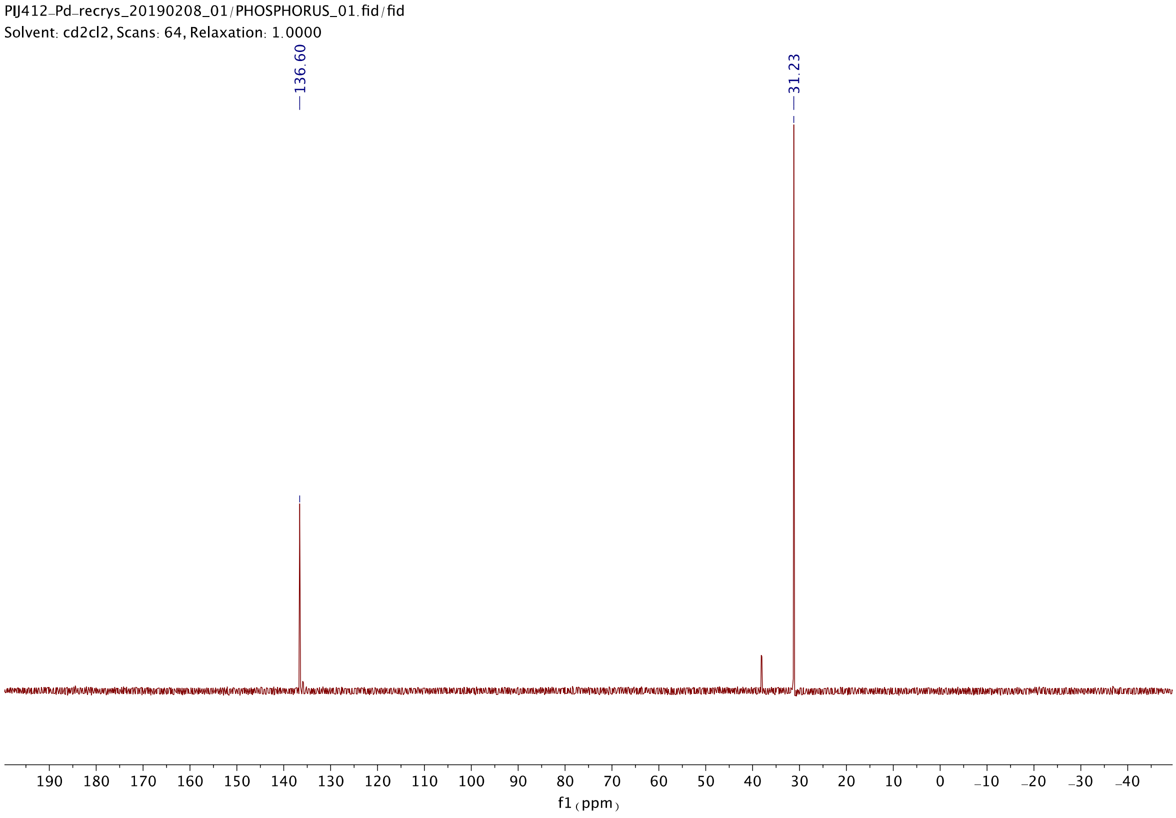

Synthetic Reference:

1. R. M. Denton, J. An, B. Adeniran, A. J. Blake, W. Lewis and A. M. Poulton, *J. Org. Chem.*, 2011, **76**, 6749-6767.
2. S. J. Hwang, D. C. Powers, A. G. Maher and D. G. Nocera, *Chem. Sci.*, 2015, **6**, 917-922.
3. [a] J. A. Buonomo, C. G. Eiden and C. C. Aldrich, *Chem. Eur. J.*, 2017, **23**, 14434-14438; [b] T. Allman and R. G. Goel, *Can. J. Chem.*, 1982, **60**, 716-722.
4. M.-L. Schirmer, S. Jopp, J. Holz, A. Spannenberg and T. Werner, *Adv. Synth. Catal.*, 2015, **358**, 26-29.
5. N. P. Kenny, K. V. Rajendran, E. V. Jennings and D. G. Gilheany, *Chem. Eur. J.*, 2013, **19**, 14210-14214.
6. H. Tomori, J. M. Fox and S. L. Buchwald, *J. Org. Chem.*, 2000, **65**, 5334-5341.
7. [a] K. Junge, B. Hagemann, S. Enthaler, A. Spannenberg, M. Michalik, G. Oehme, A. Monsees, T. Riermeier and M. Beller, *Tetrahedron: Asymmetry*, 2004, **15**, 2621-2631; [b] K. Junge, G. Oehme, A. Monsees, T. Riermeier, U. Dingerdissen and M. Beller, *Tetrahedron Lett.*, 2002, **43**, 4977-4980.
8. H. Zhang, R.-B. Hu, X.-Y. Zhang, S.-X. Li and S.-D. Yang, *Chem. Commun.*, 2014, **50**, 4686-4689.
9. H. Salem, M. Schmitt, U. Herrlich, E. Kühnel, M. Brill, P. Nägele, A. L. Bogado, F. Rominger and P. Hofmann, *Organometallics*, 2013, **32**, 29-46.
10. E. Hodson and S. J. Simpson, *Polyhedron*, 2004, **23**, 2695-2707.
11. S. Monsaert, R. Drozdzak, V. Dragutan, I. Dragutan and F. Verpoort, *Eur. J. Inorg. Chem.*, 2008, **2008**, 432-440.
12. N. Svensen, P. Fristrup, D. Tanner and P.-O. Norrby, *Adv. Synth. Catal.*, 2007, **349**, 2631-2640.
13. [a] P. S. Jarrett and P. J. Sadler, *Inorg. Chem.*, 1991, **30**, 2098-2104; [b] J. A. S. Bomfim, F. P. de Souza, C. A. L. Filgueiras, A. G. de Sousa and M. T. P. Gambardella, *Polyhedron*, 2003, **22**, 1567-1573.
14. [a] D. Fernández, M. I. García-Seijo, T. Kégl, G. Petőcz, L. Kollár and M. E. García-Fernández, *Inorg. Chem.*, 2002, **41**, 4435-4443; [b] S.-i. Aizawa, T. Iida and S. Funahashi, *Inorg. Chem.*, 1996, **35**, 5163-5167.
15. Y. Unoh, T. Satoh, K. Hirano and M. Miura, *ACS Catal.*, 2015, **5**, 6634-6639.
16. R. Anulewiczostrowska, T. Klis, D. Krajewski, B. Lewandowski and J. Serwatowski, *Tetrahedron Lett.*, 2003, **44**, 7329-7331.
17. [a] TURBOMOLE V7.1 2016, a development of University of Karlsruhe and Forschungszentrum Karlsruhe GmbH, 1989-2007, TURBOMOLE GmbH, since 2007; available from http://www.turbomole.com, http://www.turbomole.com, (accessed 05.05.2017); [b] K. Eichkorn, O. Treutler, H. Öhm, M. Häser and R. Ahlrichs, *Chem. Phys. Lett.*, 1995, **240**, 283-290; [c] F. Weigend, M. Haser, H. Patzelt and R. Ahlrichs, *Chem. Phys. Lett.*, 1998, **294**, 143-152; [d] J. P. Perdew, K. Burke and M. Ernzerhof, *Phys. Rev. Lett.*, 1997, **78**, 1396-1396; [e] S. Grimme, J. Antony, S. Ehrlich and H. Krieg, *J. Chem. Phys.*, 2010, **132**, 154104; [f] A. Schäfer, H. Horn and R. Ahlrichs, *J. Chem. Phys.*, 1992, **97**, 2571-2577; [g] A. Schäfer, C. Huber and R. Ahlrichs, *J. Chem. Phys.*, 1994, **100**, 5829-5835; [h] F. Weigend and R. Ahlrichs, *Phys. Chem. Chem. Phys.*, 2005, **7**, 3297-3305; [i] F. Weigend, *Phys. Chem. Chem. Phys.*, 2006, **8**, 1057-1065.

Single-crystal X-ray diffraction analysis report (CPS 15 and azolium 5)

Good-quality single-crystals of investigated compounds (CPS **15** and azolium **5**) were selected for the X-ray diffraction experiments at T = 100(2) K. The crystals were mounted with paratone-N oil to the MiTeGen micro-mounts (Fig. 1S). Diffraction data were collected on the Agilent Technologies SuperNova Dual Source with the Cu*K*α radiation (λ = 1.54184 Å). The lattice parameters were obtained by least-squares fit to the optimized setting angles of the reflections collected by using the CrysAlis CCD software[1]. Data were reduced using the CrysAlis RED program[1]. The analytical numeric absorption correction using a multifaceted crystal model based on expressions derived by R.C. Clark & J.S. Reid was applied[1,2]. The structural determination procedure was carried out using the SHELX package[3].

Figure S77: Single crystals of investigated compounds selected for the analysis, where (a) CPS **15** and azolium **5** (b).

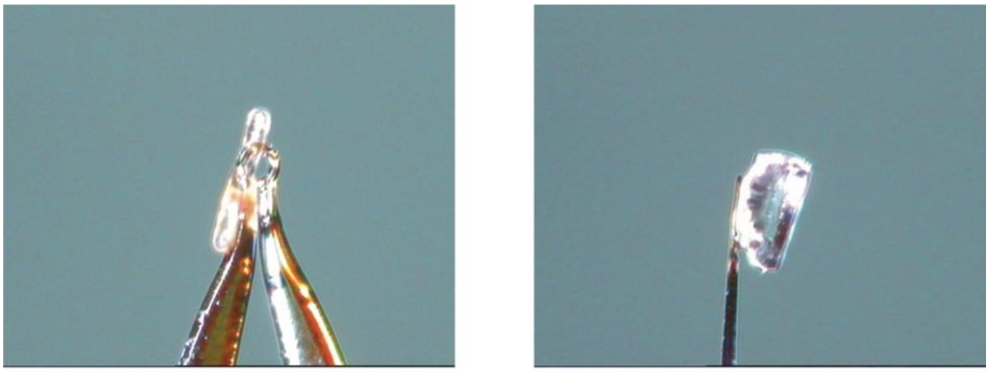

a) b)

The structure was solved with direct methods, and then successive least-squares refinements were carried out based on full-matrix least-squares on F^2 using the SHELXL program[2]. The H-atom

linked to the Cl-atom in case of CPS **15** was located on a Fourier difference map and refined as riding with $U_{iso}(H) = 1.2U_{eq}(Cl)$. Remaining H-atoms were positioned geometrically with the C−H bond length equal to 0.93, 0.96 and 0.97 Å for the aromatic, methyl and methylene H-atoms, respectively and constrained to ride on their parent atoms with $U_{iso}(H) = xU_{eq}(C)$, where x = 1.2 for the aromatic and methylene H-atoms, and 1.5 for the methyl H-atoms, respectively. One of the Cl^{-} anions in case of azolium **5** was disordered in two positions with the occupancy of 0.74 : 0.26. In case of CPS **15** the atoms C9, C10, C11 and C12 were subject of ISOR restraints. The figures for this report were prepared using Olex2[4] and ORTEP-3[5] programs.

Investigated compounds are crystallizing in the monoclinic $P2_1/c$ (CPS **15**) and $P2_1/n$ (Azolium **5**) space group. In case of CPS **15** the asymmetric unit of the crystal lattice contains one cation, two chloride anions and the molecule of hydrochloride (Fig. 2S), whereas in azolium **5** independent part of the unit cell consists two ionic pairs of compound (Fig. 3S). The crystallographic data are summarized in the in Table 1S. The values of bond lengths, valence and torsion angles are given in Tables 2S−7S. The crystal packing of investigated compounds is presented on Figs 4S and 5S.

Figure S78. The asymmetric unit of the crystal lattice of CPS **15** showing the atom labelling scheme. Displacement ellipsoids are drawn at the 50% probability level, and H-atoms are shown as small spheres of arbitrary radius. Hydrogen bonds are represented by the dashed lines.

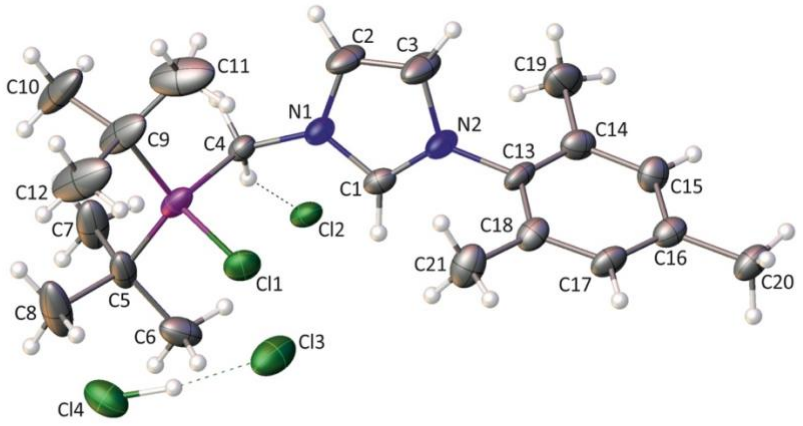

Figure S79: The asymmetric unit of the crystal lattice of azolium **5** showing the atom labelling scheme. Displacement ellipsoids are drawn at the 50% probability level, and H-atoms are shown as small spheres of arbitrary radius. Hydrogen bonds are represented by the dashed lines.

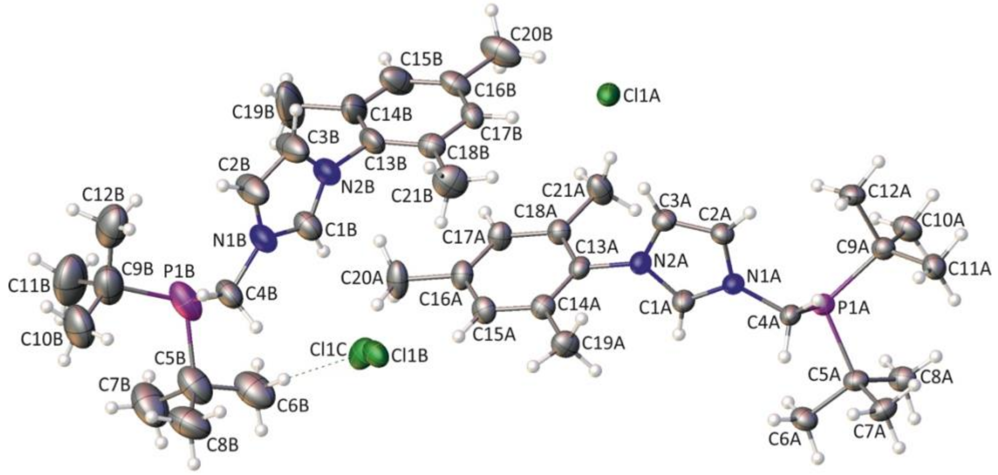

Figure S80: Supramolecular architecture of CPS **15** in the crystal, viewed along *a* -direction. Hydrogen bonds are represented by the dashed lines.The H-atoms not involved in the intermolecular interactions have been omitted for clarity.

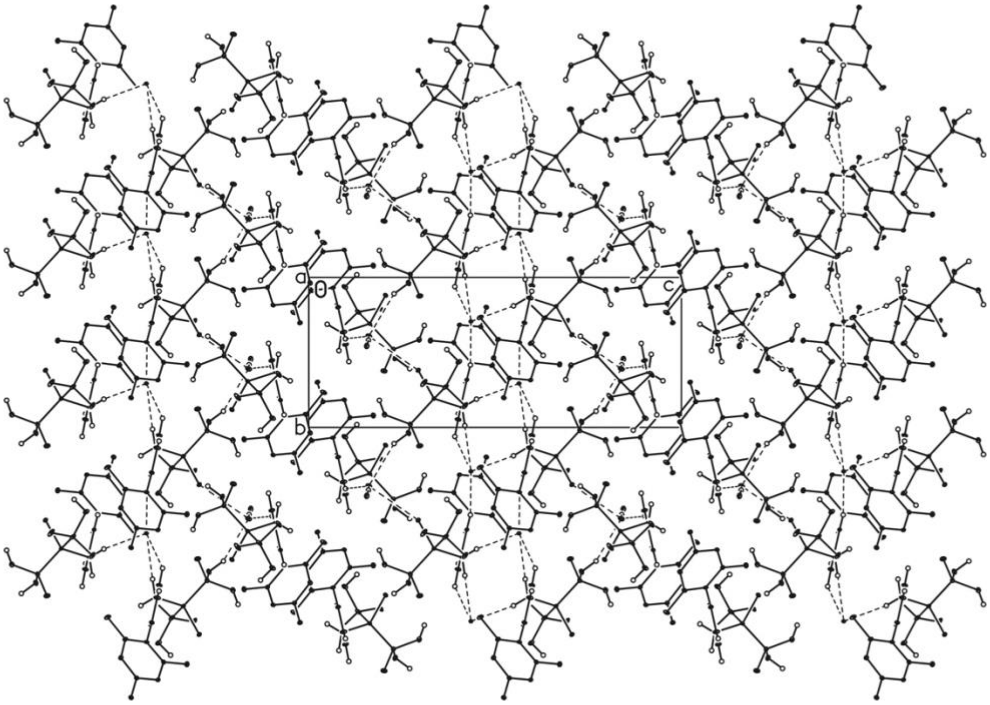

Figure S81: Supramolecular architecture of azolium **2** in the crystal, viewed along *a*, *b* and *c*-direction. Hydrogen bonds are represented by the dashed lines. The H-atoms not involved in the intermolecular interactions have been omitted for clarity.

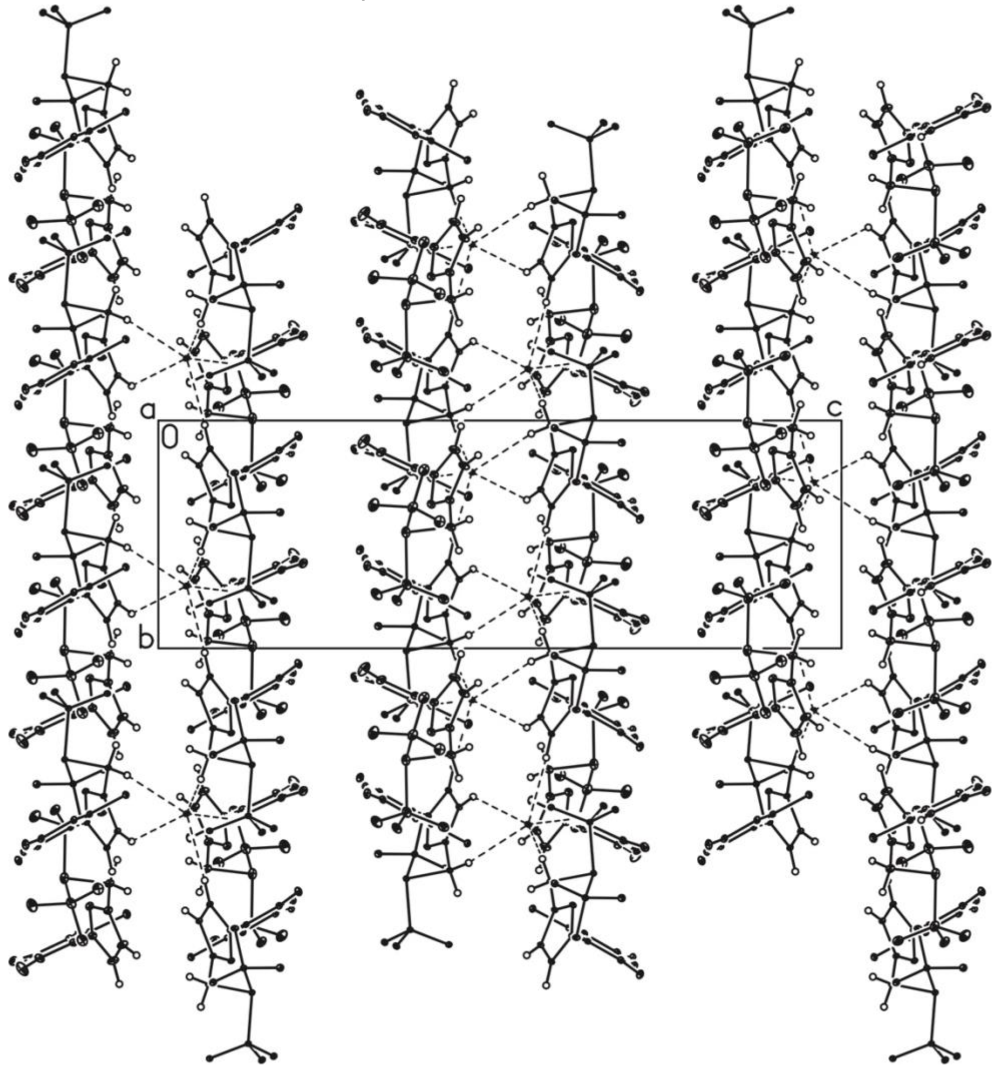

Table S4: Crystal data and structure refinement for investigated compounds.

**Identification code**	**CPS 15**	**Azolium 2**
Empirical formula	$C_{21}H_{35}Cl_4N_2P$	$C_{21}H_{34}ClN_2P$
Formula weight	488.28	380.92
Temperature/K	100(2)	100(2)
Crystal system	monoclinic	monoclinic
Space group	$P2_1/n$	$P2_1/c$
a/Å	16.0440(9)	21.6479(5)
b/Å	7.9639(4)	8.33065(14)
c/Å	19.8443(10)	25.7440(6)
α/°	90	90
β/°	92.988(5)	103.756(2)
γ/°	90	90
Volume/Å^3	2532.1(2)	4509.54(16)
Z	4	8

ρ_{calc}g/cm^3	1.281	1.122
μ/mm^{-1}	4.912	2.196
F(000)	1032.0	1648.0
Crystal size/mm^3	0.40 × 0.08 × 0.06	0.33 × 0.21 × 0.18
Radiation	Cu$K\alpha$ (λ = 1.54184)	Cu$K\alpha$ (λ = 1.54184)
2Θ range for data collection/°	6.912 to 134.154	4.202 to 134.152
Index ranges	$-19 \le h \le 19$, $-5 \le k \le 9$, $-23 \le l \le 19$	$-25 \le h \le 25$, $-5 \le k \le 9$, $-30 \le l \le 30$
Reflections collected	8946	28864
Independent reflections	4527 [R_{int} = 0.0465, R_{sigma} = 0.0761]	8034 [R_{int} = 0.0324, R_{sigma} = 0.0267]
Data/restraints/parameters	4527/24/265	8034/0/479
Goodness-of-fit on F^2	1.029	1.031
Final R indexes [I>=2σ (I)]	R_1 = 0.0539, wR_2 = 0.1203	R_1 = 0.0908, wR_2 = 0.2495
Final R indexes [all data]	R_1 = 0.0889, wR_2 = 0.1413	R_1 = 0.0985, wR_2 = 0.2582
Largest diff. peak/hole / e Å^{-3}	0.49/-0.64	2.58/-1.28

Table S5: Bond lengths for CPS **15**.

Atom	Atom	Length/Å	Atom	Atom	Length/Å
C(1)	N(1)	1.340(5)	C(9)	C(12)	1.552(6)
C(1)	N(2)	1.332(5)	C(9)	P(1)	1.855(5)
C(2)	C(3)	1.339(6)	C(13)	C(14)	1.388(6)
C(2)	N(1)	1.387(5)	C(13)	C(18)	1.396(5)
C(3)	N(2)	1.382(5)	C(13)	N(2)	1.454(5)
C(4)	N(1)	1.462(4)	C(14)	C(15)	1.392(6)
C(4)	P(1)	1.830(4)	C(14)	C(19)	1.511(5)
C(5)	C(6)	1.528(6)	C(15)	C(16)	1.375(5)
C(5)	C(7)	1.544(5)	C(16)	C(17)	1.400(6)
C(5)	C(8)	1.530(5)	C(16)	C(20)	1.501(5)
C(5)	P(1)	1.833(5)	C(17)	C(18)	1.392(5)
C(9)	C(10)	1.560(7)	C(18)	C(21)	1.514(6)
C(9)	C(11)	1.518(7)	Cl(1)	P(1)	1.9758(14)

Table S6: Valence angles for CPS **15**.

Atom	Atom	Atom	Angle/˚	Atom	Atom	Atom	Angle/˚
N(2)	C(1)	N(1)	108.3(3)	C(15)	C(14)	C(19)	120.4(4)
C(3)	C(2)	N(1)	107.4(4)	C(16)	C(15)	C(14)	122.5(4)
C(2)	C(3)	N(2)	107.3(4)	C(15)	C(16)	C(17)	118.4(4)
N(1)	C(4)	P(1)	115.9(2)	C(15)	C(16)	C(20)	120.6(4)
C(6)	C(5)	C(7)	108.3(4)	C(17)	C(16)	C(20)	121.0(4)
C(6)	C(5)	C(8)	109.8(4)	C(18)	C(17)	C(16)	122.0(4)
C(6)	C(5)	P(1)	107.8(3)	C(13)	C(18)	C(21)	122.4(4)
C(7)	C(5)	P(1)	109.1(3)	C(17)	C(18)	C(13)	116.6(4)
C(8)	C(5)	C(7)	111.0(4)	C(17)	C(18)	C(21)	121.0(4)
C(8)	C(5)	P(1)	110.8(4)	C(1)	N(1)	C(2)	108.3(3)
C(10)	C(9)	P(1)	107.8(4)	C(1)	N(1)	C(4)	125.2(3)
C(11)	C(9)	C(10)	111.3(5)	C(2)	N(1)	C(4)	126.4(3)
C(11)	C(9)	C(12)	111.1(5)	C(1)	N(2)	C(3)	108.8(3)

C(11)	C(9)	P(1)	107.2(4)	C(1)	N(2)	C(13)	126.5(3)
C(12)	C(9)	C(10)	109.2(5)	C(3)	N(2)	C(13)	124.6(3)
C(12)	C(9)	P(1)	110.3(4)	C(4)	P(1)	C(5)	105.24(18)
C(14)	C(13)	C(18)	123.6(4)	C(4)	P(1)	C(9)	110.4(2)
C(14)	C(13)	N(2)	117.6(4)	C(4)	P(1)	Cl(1)	107.61(13)
C(18)	C(13)	N(2)	118.8(4)	C(5)	P(1)	C(9)	120.3(3)
C(13)	C(14)	C(15)	116.9(4)	C(5)	P(1)	Cl(1)	107.90(15)
C(13)	C(14)	C(19)	122.7(4)	C(9)	P(1)	Cl(1)	104.89(18)

Table S7.: Torsion angles for CPS **15**.

A	B	C	D	Angle/˚	A	B	C	D	Angle/˚
C(2)	C(3)	N(2)	C(1)	0.0(5)	C(14)	C(13)	N(2)	C(3)	-63.8(5)
C(2)	C(3)	N(2)	C(13)	176.1(4)	C(14)	C(15)	C(16)	C(17)	-0.4(6)
C(3)	C(2)	N(1)	C(1)	-1.8(5)	C(14)	C(15)	C(16)	C(20)	179.7(4)
C(3)	C(2)	N(1)	C(4)	-177.5(4)	C(15)	C(16)	C(17)	C(18)	0.2(6)
C(6)	C(5)	P(1)	C(4)	69.8(3)	C(16)	C(17)	C(18)	C(13)	0.5(6)
C(6)	C(5)	P(1)	C(9)	-164.9(3)	C(16)	C(17)	C(18)	C(21)	-178.9(4)
C(6)	C(5)	P(1)	Cl(1)	-44.8(3)	C(18)	C(13)	C(14)	C(15)	0.8(6)
C(7)	C(5)	P(1)	C(4)	-47.6(4)	C(18)	C(13)	C(14)	C(19)	-178.7(4)
C(7)	C(5)	P(1)	C(9)	77.7(4)	C(18)	C(13)	N(2)	C(1)	-70.5(5)
C(7)	C(5)	P(1)	Cl(1)	-162.3(3)	C(18)	C(13)	N(2)	C(3)	114.0(5)
C(8)	C(5)	P(1)	C(4)	-170.1(3)	C(19)	C(14)	C(15)	C(16)	179.5(4)
C(8)	C(5)	P(1)	C(9)	-44.8(4)	C(20)	C(16)	C(17)	C(18)	-179.9(4)
C(8)	C(5)	P(1)	Cl(1)	75.3(3)	N(1)	C(1)	N(2)	C(3)	-1.1(5)
C(10)	C(9)	P(1)	C(4)	73.3(4)	N(1)	C(1)	N(2)	C(13)	-177.1(3)
C(10)	C(9)	P(1)	C(5)	-49.4(4)	N(1)	C(2)	C(3)	N(2)	1.1(5)
C(10)	C(9)	P(1)	Cl(1)	-171.0(3)	N(1)	C(4)	P(1)	C(5)	-141.9(3)
C(11)	C(9)	P(1)	C(4)	-46.6(4)	N(1)	C(4)	P(1)	C(9)	87.0(3)
C(11)	C(9)	P(1)	C(5)	-169.3(3)	N(1)	C(4)	P(1)	Cl(1)	-27.0(3)
C(11)	C(9)	P(1)	Cl(1)	69.1(3)	N(2)	C(1)	N(1)	C(2)	1.8(4)
C(12)	C(9)	P(1)	C(4)	-167.6(4)	N(2)	C(1)	N(1)	C(4)	177.6(3)
C(12)	C(9)	P(1)	C(5)	69.6(5)	N(2)	C(13)	C(14)	C(15)	178.5(3)
C(12)	C(9)	P(1)	Cl(1)	-51.9(5)	N(2)	C(13)	C(14)	C(19)	-1.1(6)
C(13)	C(14)	C(15)	C(16)	0.0(6)	N(2)	C(13)	C(18)	C(17)	-178.7(3)
C(14)	C(13)	C(18)	C(17)	-1.1(6)	N(2)	C(13)	C(18)	C(21)	0.7(6)
C(14)	C(13)	C(18)	C(21)	178.3(4)	P(1)	C(4)	N(1)	C(1)	81.0(4)
C(14)	C(13)	N(2)	C(1)	111.7(5)	P(1)	C(4)	N(1)	C(2)	-103.9(4)

Table S8: Bond lengths for azolium **5**.

Atom	Atom	Length/Å	Atom	Atom	Length/Å
C(1B)	N(1B)	1.319(6)	C(1A)	N(1A)	1.327(5)
C(1B)	N(2B)	1.319(6)	C(1A)	N(2A)	1.339(5)
C(2B)	C(3B)	1.354(7)	C(2A)	C(3A)	1.360(5)
C(2B)	N(1B)	1.365(7)	C(2A)	N(1A)	1.377(5)
C(3B)	N(2B)	1.365(7)	C(3A)	N(2A)	1.385(5)
C(4B)	N(1B)	1.474(6)	C(4A)	N(1A)	1.477(5)
C(4B)	P(1B)	1.866(5)	C(4A)	P(1A)	1.873(4)

C(5B)	C(6B)	1.483(9)	C(5A)	C(6A)	1.538(5)
C(5B)	C(7B)	1.527(9)	C(5A)	C(7A)	1.539(5)
C(5B)	C(8B)	1.524(10)	C(5A)	C(8A)	1.529(5)
C(5B)	P(1B)	1.938(7)	C(5A)	P(1A)	1.887(4)
C(9B)	C(10B)	1.501(9)	C(9A)	C(10A)	1.545(5)
C(9B)	C(11B)	1.577(10)	C(9A)	C(11A)	1.522(6)
C(9B)	C(12B)	1.476(11)	C(9A)	C(12A)	1.538(5)
C(9B)	P(1B)	1.874(7)	C(9A)	P(1A)	1.887(4)
C(13B)	C(14B)	1.376(7)	C(13A)	C(14A)	1.392(6)
C(13B)	C(18B)	1.405(7)	C(13A)	C(18A)	1.395(6)
C(13B)	N(2B)	1.448(6)	C(13A)	N(2A)	1.445(5)
C(14B)	C(15B)	1.369(8)	C(14A)	C(15A)	1.398(6)
C(14B)	C(19B)	1.511(7)	C(14A)	C(19A)	1.509(6)
C(15B)	C(16B)	1.368(7)	C(15A)	C(16A)	1.386(6)
C(16B)	C(17B)	1.367(6)	C(16A)	C(17A)	1.387(6)
C(16B)	C(20B)	1.520(6)	C(16A)	C(20A)	1.514(6)
C(17B)	C(18B)	1.398(6)	C(17A)	C(18A)	1.386(6)
C(18B)	C(21B)	1.499(7)	C(18A)	C(21A)	1.510(6)

Table S9: Valence angles for azolium **5**.

Atom	Atom	Atom	Angle/˚	Atom	Atom	Atom	Angle/˚
N(1B)	C(1B)	N(2B)	110.0(4)	N(1A)	C(1A)	N(2A)	108.7(3)
C(3B)	C(2B)	N(1B)	107.7(5)	C(3A)	C(2A)	N(1A)	107.5(3)
C(2B)	C(3B)	N(2B)	106.6(5)	C(2A)	C(3A)	N(2A)	106.5(3)
N(1B)	C(4B)	P(1B)	111.6(4)	N(1A)	C(4A)	P(1A)	112.2(2)
C(6B)	C(5B)	C(7B)	107.9(6)	C(6A)	C(5A)	C(7A)	108.6(3)
C(6B)	C(5B)	C(8B)	111.2(7)	C(6A)	C(5A)	P(1A)	104.8(3)
C(6B)	C(5B)	P(1B)	103.8(4)	C(7A)	C(5A)	P(1A)	116.3(3)
C(7B)	C(5B)	P(1B)	108.7(6)	C(8A)	C(5A)	C(6A)	107.9(3)
C(8B)	C(5B)	C(7B)	110.5(6)	C(8A)	C(5A)	C(7A)	109.0(3)
C(8B)	C(5B)	P(1B)	114.3(5)	C(8A)	C(5A)	P(1A)	110.0(3)
C(10B)	C(9B)	C(11B)	109.6(6)	C(10A)	C(9A)	P(1A)	107.5(3)
C(10B)	C(9B)	P(1B)	119.3(5)	C(11A)	C(9A)	C(10A)	110.1(3)
C(11B)	C(9B)	P(1B)	105.4(5)	C(11A)	C(9A)	C(12A)	108.3(3)
C(12B)	C(9B)	C(10B)	108.6(7)	C(11A)	C(9A)	P(1A)	117.4(3)
C(12B)	C(9B)	C(11B)	108.5(7)	C(12A)	C(9A)	C(10A)	107.6(3)
C(12B)	C(9B)	P(1B)	104.9(4)	C(12A)	C(9A)	P(1A)	105.5(3)
C(14B)	C(13B)	C(18B)	122.9(4)	C(14A)	C(13A)	C(18A)	122.8(4)
C(14B)	C(13B)	N(2B)	117.1(4)	C(14A)	C(13A)	N(2A)	118.4(3)
C(18B)	C(13B)	N(2B)	120.0(4)	C(18A)	C(13A)	N(2A)	118.7(3)
C(13B)	C(14B)	C(19B)	122.4(5)	C(13A)	C(14A)	C(15A)	117.5(4)
C(15B)	C(14B)	C(13B)	117.7(4)	C(13A)	C(14A)	C(19A)	122.2(4)
C(15B)	C(14B)	C(19B)	119.9(5)	C(15A)	C(14A)	C(19A)	120.3(4)
C(16B)	C(15B)	C(14B)	122.2(5)	C(16A)	C(15A)	C(14A)	121.3(4)
C(15B)	C(16B)	C(20B)	120.3(5)	C(15A)	C(16A)	C(17A)	119.1(4)
C(17B)	C(16B)	C(15B)	119.1(4)	C(15A)	C(16A)	C(20A)	119.4(4)
C(17B)	C(16B)	C(20B)	120.6(5)	C(17A)	C(16A)	C(20A)	121.5(4)

C(16B)	C(17B)	C(18B)	122.2(4)	C(18A)	C(17A)	C(16A)	121.9(4)
C(13B)	C(18B)	C(21B)	121.7(4)	C(13A)	C(18A)	C(21A)	121.1(4)
C(17B)	C(18B)	C(13B)	115.8(4)	C(17A)	C(18A)	C(13A)	117.3(4)
C(17B)	C(18B)	C(21B)	122.4(4)	C(17A)	C(18A)	C(21A)	121.6(4)
C(1B)	N(1B)	C(2B)	107.5(4)	C(1A)	N(1A)	C(2A)	108.7(3)
C(1B)	N(1B)	C(4B)	124.9(4)	C(1A)	N(1A)	C(4A)	124.1(3)
C(2B)	N(1B)	C(4B)	127.4(4)	C(2A)	N(1A)	C(4A)	127.1(3)
C(1B)	N(2B)	C(3B)	108.1(4)	C(1A)	N(2A)	C(3A)	108.6(3)
C(1B)	N(2B)	C(13B)	125.9(4)	C(1A)	N(2A)	C(13A)	125.2(3)
C(3B)	N(2B)	C(13B)	125.7(4)	C(3A)	N(2A)	C(13A)	126.2(3)
C(4B)	P(1B)	C(5B)	99.2(3)	C(4A)	P(1A)	C(5A)	99.08(17)
C(4B)	P(1B)	C(9B)	102.6(3)	C(4A)	P(1A)	C(9A)	102.84(17)
C(9B)	P(1B)	C(5B)	110.0(3)	C(5A)	P(1A)	C(9A)	110.53(18)

Table S10: Torsion angles for azolium **5**.

A	B	C	D	Angle/˚	A	B	C	D	Angle/˚
C(2B)	C(3B)	N(2B)	C(1B)	0.4(7)	C(3A)	C(2A)	N(1A)	C(4A)	179.7(3)
C(2B)	C(3B)	N(2B)	C(13B)	174.0(5)	C(6A)	C(5A)	P(1A)	C(4A)	82.2(3)
C(3B)	C(2B)	N(1B)	C(1B)	-1.5(7)	C(6A)	C(5A)	P(1A)	C(9A)	-170.4(2)
C(3B)	C(2B)	N(1B)	C(4B)	-176.6(6)	C(7A)	C(5A)	P(1A)	C(4A)	-37.7(3)
C(10B)	C(9B)	P(1B)	C(4B)	-65.2(7)	C(7A)	C(5A)	P(1A)	C(9A)	69.8(3)
C(10B)	C(9B)	P(1B)	C(5B)	39.6(7)	C(8A)	C(5A)	P(1A)	C(4A)	-162.1(3)
C(11B)	C(9B)	P(1B)	C(4B)	171.2(5)	C(8A)	C(5A)	P(1A)	C(9A)	-54.6(3)
C(11B)	C(9B)	P(1B)	C(5B)	-84.0(6)	C(10A)	C(9A)	P(1A)	C(4A)	-171.1(3)
C(12B)	C(9B)	P(1B)	C(4B)	56.7(5)	C(10A)	C(9A)	P(1A)	C(5A)	83.9(3)
C(12B)	C(9B)	P(1B)	C(5B)	161.4(5)	C(11A)	C(9A)	P(1A)	C(4A)	64.2(3)
C(13B)	C(14B)	C(15B)	C(16B)	0.7(9)	C(11A)	C(9A)	P(1A)	C(5A)	-40.8(3)
C(14B)	C(13B)	C(18B)	C(17B)	0.3(7)	C(12A)	C(9A)	P(1A)	C(4A)	-56.6(3)
C(14B)	C(13B)	C(18B)	C(21B)	-179.0(5)	C(12A)	C(9A)	P(1A)	C(5A)	-161.5(3)
C(14B)	C(13B)	N(2B)	C(1B)	88.2(7)	C(13A)	C(14A)	C(15A)	C(16A)	-0.9(6)
C(14B)	C(13B)	N(2B)	C(3B)	-84.3(7)	C(14A)	C(13A)	C(18A)	C(17A)	0.4(6)
C(14B)	C(15B)	C(16B)	C(17B)	-0.6(8)	C(14A)	C(13A)	C(18A)	C(21A)	179.3(4)
C(14B)	C(15B)	C(16B)	C(20B)	179.6(5)	C(14A)	C(13A)	N(2A)	C(1A)	79.2(5)
C(15B)	C(16B)	C(17B)	C(18B)	0.3(7)	C(14A)	C(13A)	N(2A)	C(3A)	-97.7(5)
C(16B)	C(17B)	C(18B)	C(13B)	-0.1(6)	C(14A)	C(15A)	C(16A)	C(17A)	0.1(6)
C(16B)	C(17B)	C(18B)	C(21B)	179.2(5)	C(14A)	C(15A)	C(16A)	C(20A)	-179.2(4)
C(18B)	C(13B)	C(14B)	C(15B)	-0.6(8)	C(15A)	C(16A)	C(17A)	C(18A)	1.0(7)
C(18B)	C(13B)	C(14B)	C(19B)	179.0(7)	C(16A)	C(17A)	C(18A)	C(13A)	-1.2(6)
C(18B)	C(13B)	N(2B)	C(1B)	-89.9(6)	C(16A)	C(17A)	C(18A)	C(21A)	179.9(4)
C(18B)	C(13B)	N(2B)	C(3B)	97.6(7)	C(18A)	C(13A)	C(14A)	C(15A)	0.6(6)
C(19B)	C(14B)	C(15B)	C(16B)	-178.9(7)	C(18A)	C(13A)	C(14A)	C(19A)	-178.3(4)
C(20B)	C(16B)	C(17B)	C(18B)	-179.9(4)	C(18A)	C(13A)	N(2A)	C(1A)	-102.3(5)
N(1B)	C(1B)	N(2B)	C(3B)	-1.4(6)	C(18A)	C(13A)	N(2A)	C(3A)	80.8(5)
N(1B)	C(1B)	N(2B)	C(13B)	-174.9(5)	C(19A)	C(14A)	C(15A)	C(16A)	178.0(4)
N(1B)	C(2B)	C(3B)	N(2B)	0.6(8)	C(20A)	C(16A)	C(17A)	C(18A)	-179.8(4)
N(1B)	C(4B)	P(1B)	C(5B)	142.0(4)	N(1A)	C(1A)	N(2A)	C(3A)	-0.7(4)
N(1B)	C(4B)	P(1B)	C(9B)	-105.0(4)	N(1A)	C(1A)	N(2A)	C(13A)	-178.1(3)

N(2B)	C(1B)	N(1B)	C(2B)	1.7(6)	N(1A)	C(2A)	C(3A)	N(2A)	0.1(4)
N(2B)	C(1B)	N(1B)	C(4B)	177.0(5)	N(1A)	C(4A)	P(1A)	C(5A)	-143.0(3)
N(2B)	C(13B)	C(14B)	C(15B)	-178.7(5)	N(1A)	C(4A)	P(1A)	C(9A)	103.4(3)
N(2B)	C(13B)	C(14B)	C(19B)	0.9(9)	N(2A)	C(1A)	N(1A)	C(2A)	0.8(4)
N(2B)	C(13B)	C(18B)	C(17B)	178.3(4)	N(2A)	C(1A)	N(1A)	C(4A)	-179.5(3)
N(2B)	C(13B)	C(18B)	C(21B)	-1.0(7)	N(2A)	C(13A)	C(14A)	C(15A)	179.0(3)
P(1B)	C(4B)	N(1B)	C(1B)	-67.1(6)	N(2A)	C(13A)	C(14A)	C(19A)	0.2(6)
P(1B)	C(4B)	N(1B)	C(2B)	107.3(6)	N(2A)	C(13A)	C(18A)	C(17A)	-178.0(3)
C(2A)	C(3A)	N(2A)	C(1A)	0.3(4)	N(2A)	C(13A)	C(18A)	C(21A)	0.8(6)
C(2A)	C(3A)	N(2A)	C(13A)	177.7(4)	P(1A)	C(4A)	N(1A)	C(1A)	72.5(4)
C(3A)	C(2A)	N(1A)	C(1A)	-0.6(4)	P(1A)	C(4A)	N(1A)	C(2A)	-107.9(4)

X-ray Reference:

1. *CrysAlis CCD and CrysAlis RE*D; Oxford Diffraction Ltd: Yarnton, 2008.
2. *R*.C. Clark & J.S. Reid, *Acta Crystallogr., Sect A: Found. Crystallogr.* 1995, **51**, 887−897.
3. G.M. Sheldrick, *Acta Crystallogr., Sect. A: Found. Crystallogr*. 2008, **64**, 112−122.
4. O.V. Dolomanov, L.J. Bourhis, R.J. Gildea, J.A.K. Howard, H. Puschmann, *J. Appl. Cryst.* 2009, 42, 339–341.
5. L.J. Farrugia, *J. Appl. Cryst.* 1997, **30**, 565.
